# Supplementary material for: Enantioconvergent construction of stereogenic silicon via Lewis base-catalyzed dynamic kinetic silyletherification of racemic chlorosilanes
Source: Nat Commun. 2023 Aug 14;14:4900. doi: 10.1038/s41467-023-40558-6 (PMC10425371; doi:10.1038/s41467-023-40558-6)
Supplement: Supplementary file 1 — Supplementary Information [file 41467_2023_40558_MOESM1_ESM.pdf]

Supplementary Information

Enantioconvergent Construction of Stereogenic Silicon *via* Lewis Base-Catalyzed Dynamic Kinetic Silyletherification of Racemic Chlorosilanes

Tianbao Hu,[1] Chen Zhao,[1] Yan Zhang,[2] Yuzhong Kuang,[3] Lu Gao,[1] Wanshu Wang,[1] Zhishan Su[2]* and Zhenlei Song[1]**

[1]Key Laboratory of Drug-Targeting and Drug Delivery System of the Education Ministry and Sichuan Province, Sichuan Engineering Laboratory for Plant-Sourced Drug and Sichuan Research Center for Drug Precision Industrial Technology, West China School of Pharmacy, Sichuan University, Chengdu, 610041, China.
[2]Key Laboratory of Green Chemistry and Technology, Ministry of Education, College of Chemistry, Sichuan University, Chengdu, 610064, China.
[3]School of Pharmacy, China Pharmaceutical University, 639 Longmian Avenue, Nanjing, Jiangsu, 211198, China.

Content

1. Supplementary Methods 2
1.1 General Information 2
1.2 Experimental Procedures 3
1.3 Crystallographic Data of **6g** 51
2. Supplementary Discussion 60
2.1 Optimization of Reaction Conditions 60
2.2 ^{29}Si NMR Studies 62
2.3 DFT studies 63
3. Supplementary Figures 11-210 66
4. Supplementary Figures 211-217 166
5. Supplementary References 173

1. Supplementary Methods

1.1 General Information

Commercial reagents were used without any purification. Reagents were used as purchased from J&K, Alfa Aesar, TCI, or Energy Chemicals reagent suppliers. **1i**, **2a-2g**, **3a-3d** and **3f** were purchased for those are commercially available, or prepared according to literatures for those are known compounds. CCl_4, CH_2Cl_2 and Et_3N were distilled from CaH_2. toluene, Et_2O and THF were distilled from sodium. Inert atmosphere techniques were carried out through Schlenk system. TLC which was performed on glass-backed silica plates (purchased from Yantai Jiangyou Silica Gel Development Co. Ltd.) and visualized using UV at 254 nm, $KMnO_4$ stains. Column chromatography was performed using silica gel (200-300, purchased from Yantai Jiangyou Silica Gel Development Co. Ltd.). Melting point were recorded at WRX-4 Melting-point Apparatus (purchased from Shanghai Yice Apparatus & Equipments Co. Lit.). ^{1}H NMR spectra were recorded at 400 MHz (Varian and Bruker) or 600 MHz (Agilent) using $CDCl_3$ (except where noted), ^{13}C NMR spectra were recorded at 100 MHz (Bruker) or 150 MHz (Agilent) using $CDCl_3$ (except where noted), ^{19}F NMR spectra were recorded at 376 MHz (Agilent) using $CDCl_3$ (except where noted), ^{29}Si NMR spectra were recorded at 80 MHz (Bruker) or 120 MHz (Bruker) using $CDCl_3$ as solvent (except where noted), High-resolution mass spectral analyses performed on Waters Q-TOF in positive mode. Infrared spectra were obtained using PerkinElmer Spectrum Two FTIR Spectrometer. X-ray diffraction experiment was carried out on Xcalibur E and the data obtained was deposited at the Cambridge Crystallographic Data Centre. Enantiomeric excess was determined by HPLC (Agilent Technologies: 1260 Infinity II) analysis on a Daicel Chiralcel OD-H column or Phenomenex Lux Cellulose-3 column. Specific optical rotation was measured on PL341 Polarimeter (PerkinElmer). All spectral data obtained for new compounds are reported here.

1.2 Experimental Procedures

Synthesis of Chlorosilanes 1

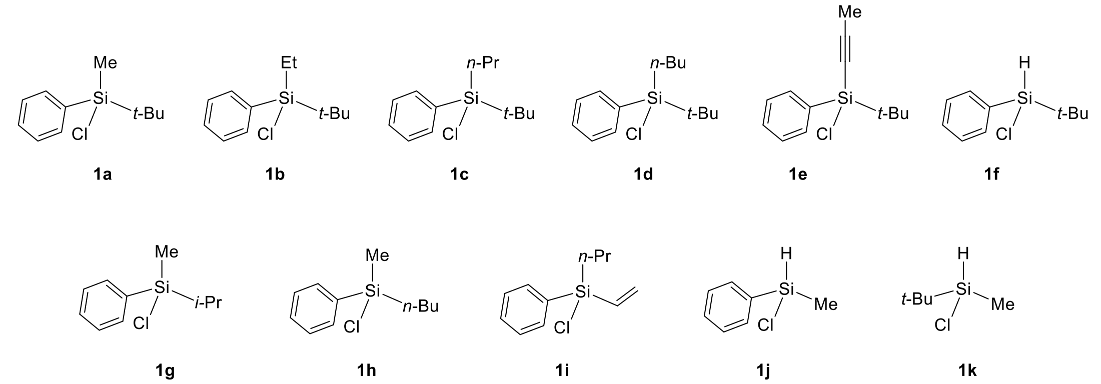

1i is commercially available. **1a**[1], **1b**[2], **1c**[2], **1d**[1], **1e**[3], **1f**[4], **1g**[1], **1h**[1], **1j**[5], **1k**[6] was prepared according to the literature, and the NMR spectral data are in accordance with the reported data.

Synthesis of 1a[1]

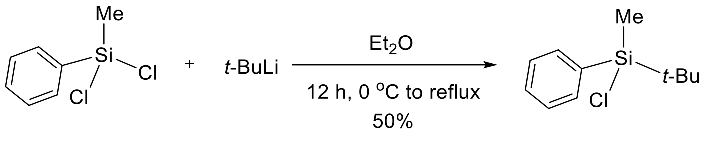

To a 250 mL flask under an argon atmosphere equipped with a magnetic stirrer and a dropping funnel was charged with dichloromethylphenylsilane (18 mL, 110 mmol, 1.0 equiv.) and Et_2O (90 mL) and was cooled to 0 °C. Freshly titrated *t*-BuLi (79 mL, 110 mmol, 1.4 M in pentane, 1.0 equiv.) was added dropwise *via* the dropping funnel over 3 h. After complete addition, the reaction mixture was subsequently heated to 40 °C and refluxed overnight. The white slurry was filtered under an argon atmosphere and the white solid was thoroughly washed with Et_2O (50 mL × 3). Fractional distillation of the filtrate afforded the desired product **1a** as a colorless oil (11.6 g, 50%).

Synthesis of Hydrosilane S1: The Precursor of Chlorosilanes 1

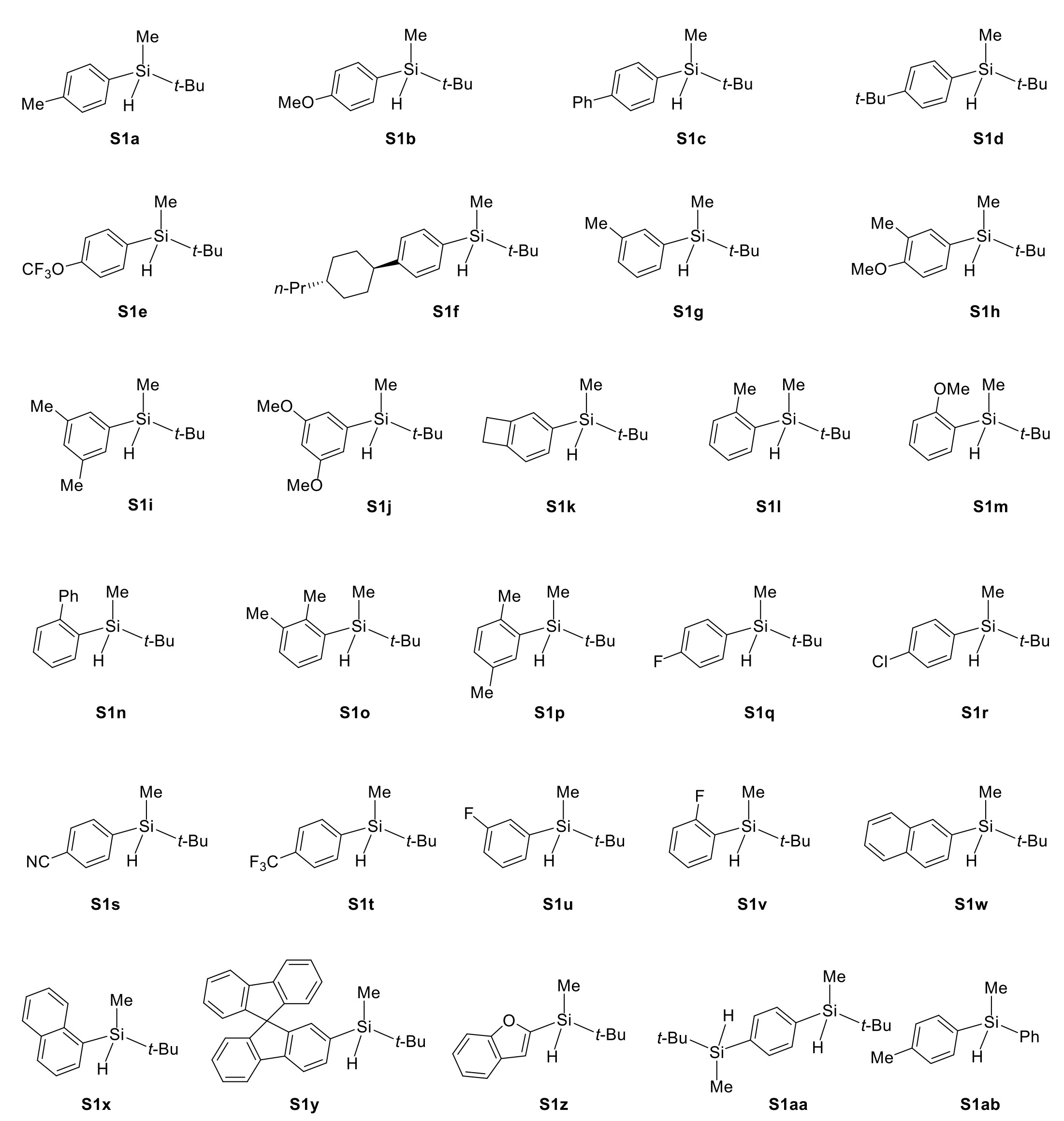

S1x[6-8] was prepared according to the literature, and the NMR spectral data are in accordance with the reported data. **S1a-S1w** and **S1y-S1ab** are new compounds and were prepared according to **Method 3A** or **3B**.

Method 3A:

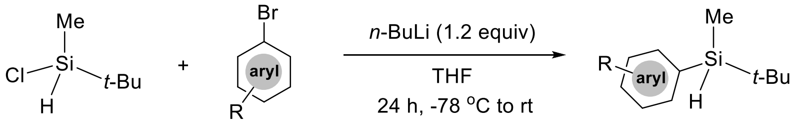

1k **S1**

To a solution of arylbromide (1.0 equiv.) in dry THF was added *n*-BuLi (2.4 M in *n*-hexane, 1.2 equiv.) dropwise at -78 °C under argon. The resulting mixture was stirred for 3 h at -78 °C before

adding **1k** (1.5 equiv.). The reaction was slowly warmed up to room temperature and stirred for 12 h. Finally, the reaction was quenched with aq. NH_4Cl (30 mL). The organic layers were separated and the aqueous layer was extracted with EtOAc (10 mL × 3). The combined organic layers were washed with sat. aq. NaCl, dried over Na_2SO_4 and concentrated under reduced pressure. The residue was purified by silica gel column chromatography (gradient eluent: Petroleum Ether to Petroleum Ether /EtOAc = 50:1) to afford **S1**.

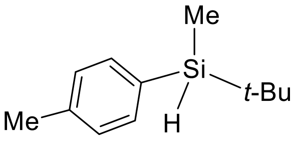

S1a

tert-butyl(methyl)(p-tolyl)silane (**S1a**)

S1a was prepared according to Method **3A**.

4-bromotoluene (1.71 g, 10 mmol), *n*-BuLi (5 mL, 12 mmol, 2.4 M in hexane) and **1k** (2 mL, 15 mmol) in dry THF (30 mL) afforded **S1a** as a colorless oil (1.72 g, 90%).

- R_f = 0.9 (Petroleum Ether).
- ^{1}H NMR (400 MHz, $CDCl_3$) δ 7.43 (d, *J* = 8.0 Hz, 2H), 7.23 (d, *J* = 8.0 Hz, 2H), 4.11 (q, *J* = 4 Hz, 1H), 2.49 (s, 3H), 0.92 (s, 9H), 0.32 (d, *J* = 4.0 Hz, 3H).
- ^{13}C NMR (100 MHz, $CDCl_3$) δ 140.0, 135.4, 131.4, 125.3, 26.8, 16.6, 15.2, -8.5.
- IR (neat) cm $^{-1}$ 2856, 2108, 1603, 1664, 1413, 1107, 832.
- HRMS calcd for $C_{12}H_{20}Si$ $(M+H)^+$ 193.1407 found 193.1411.

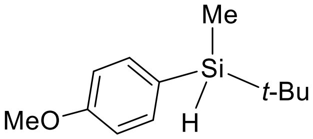

S1b

tert-butyl(4-methoxyphenyl)(methyl)silane (**S1b**)

S1b was prepared according to Method **3A**.

4-Bromoanisole (1.87g, 10 mmol), *n*-BuLi (5 mL, 12 mmol, 2.4 M in hexane) and **1k** (2 mL, 15 mmol) in dry THF (30 mL) afforded **S1b** as a colorless oil (1.62 g, 78%).

- R_f = 0.4 (Petroleum Ether).
- ^{1}H NMR (400 MHz, $CDCl_3$) δ 7.47 (d, *J* = 8 Hz, 2H), 6.92 (d, *J* = 8 Hz, 2H), 4.12 (q, *J* = 4 Hz, 1H), 3.83 (s, 3H), 0.94 (s, 9H), 0.32 (d, *J* = 4 Hz, 3H).
- ^{13}C NMR (100 MHz, $CDCl_3$) δ 160.0, 136.4, 126.2, 113.4, 55.0, 26.8, 16.7, -8.3.
- IR (neat) cm $^{-1}$ 2929, 2107, 1593, 1500, 1246, 1111, 816.
- HRMS calcd for $C_{12}H_{20}OSi$ $(M+H)^+$ 209.1356 found 209.1355.

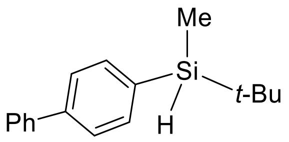

S1c

[1,1'-biphenyl]-4-yl(tert-butyl)(methyl)silane (**S1c**)

S1c was prepared according to Method **3A**.

4-Bromobiphenyl (2.33g, 10 mmol), *n*-BuLi (5 mL, 12 mmol, 2.4 M in hexane) and **1k** (2 mL, 15 mmol) in dry THF (30 mL) afforded **S1c** as a white solid (2.08 g, 82%).

- mp: 62.1 - 63.0 °C.
- R_f = 0.8 (Petroleum Ether).
- ^{1}H NMR (400 MHz, $CDCl_3$) δ 7.62 - 7.54 (m, 6H), 7.42 - 7.38 (m, 2H), 7.32 - 7.28 (m, 1H), 4.19 (q, J = 4 Hz, 1H), 0.96 (s, 9H), 0.35 (d, J = 4 Hz, 3H).
- ^{13}C NMR (100 MHz, $CDCl_3$) δ 141.9, 141.0, 135.6, 134.2, 128.8, 127.4, 127.1, 126.3, 26.9 16.7, -8.4.
- IR (neat) cm $^{-1}$ 3057, 2926, 2111, 1465, 1112, 822.
- HRMS calcd for $C_{17}H_{22}Si$ $(M+H)^+$ 255.1564 found 255.1563.

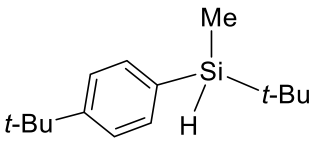

S1d

tert-butyl(4-(tert-butyl)phenyl)(methyl)silane (**S1d**)

S1d was prepared according to Method **3A**.

1-Bromo-4-*tert*-butylbenzene (2.13g, 10 mmol), *n*-BuLi (5 mL, 12 mmol, 2.4 M in hexane) and **1k** (2 mL, 15 mmol) in dry THF (30 mL) afforded **S1d** as a colorless oil (1.63 g, 70%).

- R_f = 0.8 (Petroleum Ether).
- ^{1}H NMR (400 MHz, $CDCl_3$) δ 7.35 (d, J = 8 Hz, 2H), 7.25 (d, J = 8 Hz, 2H), 4.00 (q, J = 4 Hz, 1H), 1.20 (s, 9H), 0.82 (s, 9H), 0.20 (d, J = 4 Hz, 3H)
- ^{13}C NMR (150 MHz, $CDCl_3$) δ 152.1, 135.0, 131.8, 124.6, 34.7, 31.3, 26.9, 16.7, -8.4.
- IR (neat) cm $^{-1}$ 3057, 2926, 2111, 1465, 1112, 822.
- HRMS calcd for $C_{15}H_{26}Si$ $(M+H)^+$ 235.1877 found 235.1875.

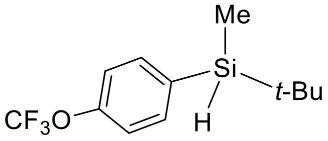

S1e

tert-butyl(methyl)(4-(trifluoromethoxy)phenyl)silane (**S1e**)

S1e was prepared according to Method **3A**.

1-Bromo-4-(trifluoromethoxy)benzene (2.41 g, 10 mmol), *n*-BuLi (5 mL, 12 mmol, 2.4 M in hexane) and **1k** (2 mL, 15 mmol) in dry THF (30 mL) afforded **S1e** as a colorless oil (1.54 g, 59%).

- R_f = 0.8 (Petroleum Ether).
- ^{1}H NMR (400 MHz, $CDCl_3$) δ 7.62 (d, J = 8 Hz, 2H), 7.27 (d, J = 8 Hz, 2H), 4.22 (q, J = 4 Hz, 1H), 1.01 (s, 9H), 0.42 (q, J = 4 Hz, 3H).
- ^{13}C NMR (150 MHz, $CDCl_3$) δ 150.4, 136.6, 134.4, 120.7 (q, J = 256 Hz), 119.9, 34.7, 31.3,

26.7, 16.6, -8.4.

- IR (neat) cm $^{-1}$ 2931, 2858, 2116, 1363, 1252, 826.
- HRMS calcd for $C_{12}H_{17}F_3OSi$ $(M+H)^+$ 263.1074 found 263.1076.

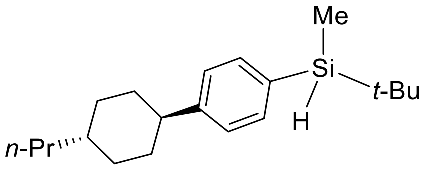

S1f

tert-butyl(methyl)(4-((1s,4r)-4-propylcyclohexyl)phenyl)-silane (**S1f**)

S1f was prepared according to Method **3A**.

1-Bromo-4-(*trans*-4-propylcyclohexyl)benzene (2.81g, 10 mmol), *n*-BuLi (5 mL, 12 mmol, 2.4 M in hexane) and **1k** (2 mL, 15 mmol) in dry THF (30 mL) afforded **S1f** as a colorless oil (2.63 g, 87%).

- R_f = 0.8 (Petroleum Ether).
- ^{1}H NMR (400 MHz, $CDCl_3$) δ 7.28 (d, *J* = 8 Hz, 2H), 7.04 (d, *J* = 8 Hz, 2H), 3.95 (q, *J* = 4 Hz, 1H), 2.32 - 2.25 (m, 1H), 1.75 - 1.68 (m, 4H), 1.34 - 1.02 (m,5H), 0.93 - 0.83 (m, 2H), 0.77 (s, 9H), 0.74 (t, *J* = 4 Hz, 3H), 0.15 (t, *J* = 4 Hz, 3H).
- ^{13}C NMR (100 MHz, $CDCl_3$) δ 148.9, 135.1, 132.2, 126.2, 44.6, 39.8, 37.1, 34.2, 33.6, 26.9, 20.1, 16.7, 14.4, -8.4.
- IR (neat) cm $^{-1}$ 2957, 2921, 2108, 1600, 1461, 1109, 833.
- HRMS calcd for $C_{20}H_{34}Si$ $(M+H)^+$ 303.2503 found 303.2502.

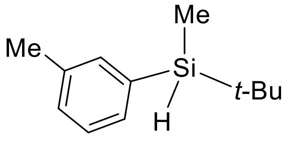

S1g

tert-butyl(methyl)(m-tolyl)silane (**S1g**)

S1g was prepared according to Method **3A**.

3-Bromotoluene (1.71g, 10 mmol), *n*-BuLi (5 mL, 12 mmol, 2.4 M in hexane) and **1k** (2 mL, 15 mmol) in dry THF (30 mL) afforded **S1g** as a colorless oil (1.68 g, 88%).

- R_f = 0.9 (Petroleum Ether).
- ^{1}H NMR (400 MHz, $CDCl_3$) δ 7.20 (d, *J* = 8.0 Hz, 2H), 7.13 - 7.09 (m, 1H), 7.06 - 7.04 (m, 1H), 4.00 (q, *J* = 4 Hz, 1H), 2.22 (s, 3H), 0.81 (s, 9H), 0.20 (d, *J* = 4 Hz, 3H).
- ^{13}C NMR (100 MHz, $CDCl_3$) δ 136.9, 135.7, 135.3, 132.1, 130.0, 127.5, 26.9, 21.5, 16.6, -8.4.
- IR (neat) cm $^{-1}$ 2927, 2109, 1464, 1118, 872, 822.
- HRMS calcd for $C_{12}H_{20}Si$ $(M+H)^+$ 193.1407 found 193.1410.

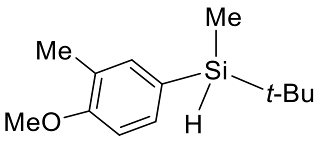

S1h

tert-butyl(4-methoxy-3-methylphenyl)(methyl)silane (**S1h**)

S1h was prepared according to Method **3A**.

4-Bromo-2-methylanisole (2.01 g, 10 mmol), *n*-BuLi (5 mL, 12 mmol, 2.4 M in hexane) and **1k** (2 mL, 15 mmol) in dry THF (30 mL) afforded **S1h** as a colorless oil (1.95 g, 88%).

- R_f = 0.2 (Petroleum Ether).
- ^{1}H NMR (400 MHz, $CDCl_3$) δ 7.35 (d, J = 8.0 Hz, 1H), 7.29 (s, 1H), 6.85 (d, J = 8.0 Hz, 1H), 4.11 (q, J = 4 Hz, 1H), 3.85 (s, 3H), 2.24 (s, 3H), 0.95 (s, 9H), 0.32 (d, J = 4 Hz, 3H).
- ^{13}C NMR (100 MHz, $CDCl_3$) δ 158.8, 137.3, 134.1, 125.9, 125.8 109.3, 55.0, 26.9, 16.7, 16.2, -8.2.
- IR (neat) cm $^{-1}$ 3055, 2854, 2103, 1592, 1260, 1109, 1034, 839.
- HRMS calcd for $C_{13}H_{22}OSi$ $(M+Na)^+$ 245.1332 found 245.1335.

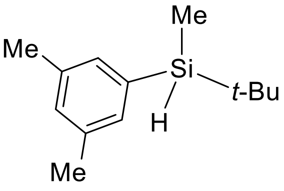

S1i

tert-butyl(3,5-dimethylphenyl)(methyl)silane (**S1i**)

S1i was prepared according to Method **3A**.

5-Bromo-*m*-xylene (1.85g, 10 mmol), *n*-BuLi (5 mL, 12 mmol, 2.4 M in hexane) and **1k** (2 mL, 15 mmol) in dry THF (30 mL) afforded **S1i** as a colorless oil (1.58 g, 77%).

- R_f = 0.9 (Petroleum Ether).
- ^{1}H NMR (400 MHz, $CDCl_3$) δ 7.02 (s, 2H), 6.89 (s, 1H), 3.98 (q, J = 4 Hz, 1H), 2.19 (s, 6H), 0.82 (s, 9H), 0.19 (d, J = 4 Hz, 3H).
- ^{13}C NMR (100 MHz, $CDCl_3$) δ 136.8, 135.2, 132.8, 131.0, 26.9, 21.4, 16.6, -8.3.
- IR (neat) cm $^{-1}$ 2926, 2856, 2108, 1464, 855.
- HRMS calcd for $C_{13}H_{22}Si$ $(M+H)^+$ 207.1564 found 207.1569.

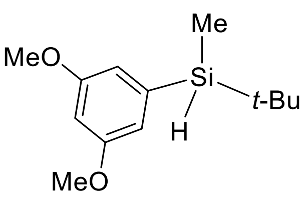

S1j

tert-butyl(3,5-dimethoxyphenyl)(methyl)silane (**S1j**)

S1j was prepared according to Method **3A**.

1-Bromo-3, 5-dimethoxybenzene (2.17g, 10 mmol), *n*-BuLi (5 mL, 12 mmol, 2.4 M in hexane) and **1k** (2 mL, 15 mmol) in dry THF (30 mL) afforded **S1j** as a colorless oil (1.04 g, 44%).

- R_f = 0.3 (Petroleum Ether: Ethyl Acetate = 50 : 1).
- ^{1}H NMR (400 MHz, $CDCl_3$) δ 6.67 (d, J = 2.4 Hz, 2H), 6.49 (t, J = 2.4 Hz, 1H), 4.10 (q, J = 4Hz, 1H), 3.81 (s, 6H), 0.95 (s, 9H), 0.33 (d, J = 4 Hz, 3H).

- ^{13}C NMR (100 MHz, $CDCl_3$) δ 160.2, 137.8, 112.6, 101.1, 55.2, 26.9, 16.6, -8.4.
- IR (neat) cm $^{-1}$ 2930, 2109, 1581, 1402, 1152, 814.
- HRMS calcd for $C_{13}H_{22}O_2Si$ $(M+H)^+$ 239.1462 found 239.1460.

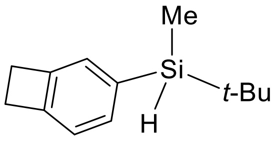

S1k

bicyclo[4.2.0]octa-1(6),2,4-trien-3-yl(tert-butyl)(methyl)silane (**S1k**)

S1k was prepared according to Method **3A**.

4-Bromobenzocyclobutene (1.83g, 10 mmol), *n*-BuLi (5 mL, 12 mmol, 2.4 M in hexane) and **1k** (2 mL, 15 mmol) in dry THF (30 mL) at -78 °C, slowly warmed up to room temperature and stirred for 12 h afforded **S1l** as a colorless oil (1.61 g, 79%).

- R_f = 0.7 (Petroleum Ether).
- ^1H NMR (400 MHz, $CDCl_3$) δ 7.26 (d, J = 8 Hz, 1H), 7.10 (s, 1H), 6.93 (d, J = 8Hz, 1H), 3.99 (q, J = 4 Hz, 1H), 3.07 (s, 4H), 0.81 (s, 9H), 0.19 (d, J = 4 Hz, 3H).
- ^{13}C NMR (100 MHz, $CDCl_3$) δ 147.3, 145.4, 133.4, 128.8, 121.8, 29.9, 29.8, 26.9, 16.6, -8.2.
- IR (neat) cm $^{-1}$ 2927, 2854, 2106, 1464, 1050, 815.
- HRMS calcd for $C_{12}H_{20}Si$ $(M+H)^+$ 205.1407 found 205.1410.

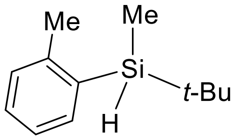

S1l

tert-butyl(methyl)(o-tolyl)silane (**S1l**)

S1l was prepared according to Method **3A**.

2-Bromotoluene (1.71g, 10 mmol), *n*-BuLi (5 mL, 12 mmol, 2.4 M in hexane) and **1k** (2 mL, 15 mmol) in dry THF (30 mL) at -78 °C, slowly warmed up to room temperature and stirred for 12 h afforded **S1l** as a colorless oil (1.11 g, 58%).

- R_f = 0.9 (Petroleum Ether).
- ^1H NMR (400 MHz, $CDCl_3$) δ 7.44 (d, J = 8 Hz, 1H), 7.28 (dd, J = 11.2, 4 Hz, 1H), 7.16 (t, J = 8 Hz, 2H), 4.35 (q, J = 4 Hz, 1H), 2.47 (s, 3H), 0.96 (s, 9H), 0.35 (d, J = 4 Hz, 3H).
- ^{13}C NMR (150 MHz, $CDCl_3$) δ 144.0, 135.7, 134.4, 129.7, 129.3, 124.6, 27.3, 23.4, 17.5, -7.4.
- IR (neat) cm $^{-1}$ 3056, 2928, 2855, 2119, 1463, 1250, 829.
- HRMS calcd for $C_{12}H_{20}Si$ $(M+H)^+$ 193.1407 found 193.1412.

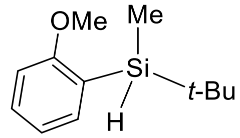

S1m

tert-butyl(2-methoxyphenyl)(methyl)silane (**S1m**)

S1m was prepared according to Method **3A**.

2-Bromoanisole (1.87g, 10 mmol), *n*-BuLi (5 mL, 12 mmol, 2.4 M in hexane) and **1k** (2 mL, 15 mmol) in dry THF (30 mL) at -78 °C, slowly warmed up to room temperature and stirred for 12 h afforded **S1m** as a colorless oil (0.79 g, 38%).

- R_f = 0.3 (Petroleum Ether).
- ^{1}H NMR (400 MHz, $CDCl_3$) δ 7.42 (dd, *J* = 8, 4 Hz, 1H), 7.32 (ddd, *J* = 8, 8, 4 Hz, 1H), 6.91 (td, *J* = 8, 4 Hz, 1H), 6.79 (d, *J* = 8 Hz, 4H), 4.12 (q, *J* = 4 Hz, 1H), 3.75 (s, 3H), 0.93 (s, 9H), 0.32 (d, *J* = 4 Hz, 3H).
- ^{13}C NMR (100 MHz, $CDCl_3$) δ 164.2, 137.4, 131.1, 123.8, 120.3, 109.5, 54.8, 27.5, 17.1, -7.7.
- IR (neat) cm $^{-1}$ 2929, 2101, 1585, 1463, 1237, 828.
- HRMS calcd for $C_{12}H_{20}OSi$ $(M+H)^+$ 209.1356 found 209.1360.

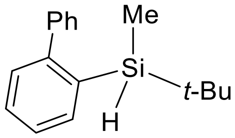

S1n

[1,1'-biphenyl]-2-yl(tert-butyl)(methyl)silane (**S1n**)

S1n was prepared according to Method **3A**.

2-Bromobiphenyl (2.33g, 10 mmol), *n*-BuLi (5 mL, 12 mmol, 2.4 M in hexane) and **1k** (2 mL, 15 mmol) in dry THF (30 mL) at -78 °C, slowly warmed up to room temperature and stirred for 12 h afforded **S1n** as a colorless oil (1.77 g, 70%).

- R_f = 0.7 (Petroleum Ether).
- ^{1}H NMR (400 MHz, $CDCl_3$) δ 7.53 (d, *J* = 8 Hz, 1H), 7.31 - 7.19 (m, 8H), 3.91 (d, *J* = 4 Hz, 1H), 0.72 (s, 9H), 0.11 (d, *J* = 4 Hz, 3H).
- ^{13}C NMR (100 MHz, $CDCl_3$) δ 149.7, 143.8, 135.6, 134.1, 129.9, 129.7, 128.8, 127.4, 126.9, 126.0, 27.4, 17.1, -6.9.
- IR (neat) cm $^{-1}$ 3054, 2927, 2147, 1463, 1084, 830.
- HRMS calcd for $C_{17}H_{22}Si$ $(M+H)^+$ 255.1564 found 255.1563.

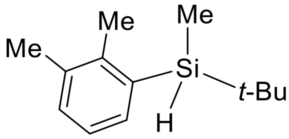

S1o

tert-butyl(2,3-dimethylphenyl)(methyl)silane (**S1o**)

S1o was prepared according to Method **3A**.

2, 3-Dimethylbromobenzene (1.85g, 10 mmol), *n*-BuLi (5 mL, 12 mmol, 2.4 M in hexane) and **1k** (2 mL, 15 mmol) in dry THF (30 mL) at -78 °C, slowly warmed up to room temperature and stirred for 12 h afforded **S1o** as a colorless oil (1.85 g, 90%).

- R_f = 0.8 (Petroleum Ether).
- ^{1}H NMR (400 MHz, $CDCl_3$) δ 7.30 (dd, *J* = 8, 4 Hz, 1H), 7.15 (d, *J* = 8 Hz, 1H), 7.08 (t, *J* = 8 Hz, 1H), 4.39 (q, *J* = 4 Hz, 1H), 2.38 (s, 3H), 2.26 (s, 3H), 0.95 (s, 9H), 0.35 (d, *J* = 4 Hz, 3H).
- ^{13}C NMR (100 MHz, $CDCl_3$) δ 142.5, 136.4, 134.5, 133.7, 131.0, 124.8, 27.4, 20.7, 20.3, 17.5, -7.0.
- IR (neat) cm $^{-1}$ 3005 2926, 2115, 1462, 1275, 1260, 891, 764.
- HRMS calcd for $C_{13}H_{22}Si$ $(M+H)^+$ 207.1564 found 207.1566.

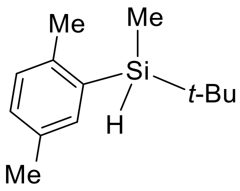

S1p

tert-butyl(2,5-dimethylphenyl)(methyl)silane (**S1p**)

S1p was prepared according to Method **3A**.

2, 5-Dimethylbromobenzene (1.85g, 10 mmol), *n*-BuLi (5 mL, 12 mmol, 2.4 M in hexane) and **1k** (2 mL, 15 mmol) in dry THF (30 mL) at -78 °C, slowly warmed up to room temperature and stirred for 12 h afforded **S1p** as a colorless oil (1.90 g, 92%).

- R_f = 0.8 (Petroleum Ether).
- ^{1}H NMR (400 MHz, $CDCl_3$) δ 7.23 (s, 9H),7.07 (d, *J* = 8 Hz, 2H), 4.33 (d, *J* = 4 Hz, 1H), 2.42 (s, 3H), 2.31 (s, 3H), 0.95 (s, 9H), 0.35 (d, *J* = 4 Hz, 3H).
- ^{13}C NMR (100 MHz, $CDCl_3$) δ 140.9, 136.4, 134.2, 133.7, 130.1, 129,7, 27.4, 22.9, 21.1, -7.3.
- IR (neat) cm $^{-1}$ 3004, 2926, 2118, 1470, 1275, 888, 764.
- HRMS calcd for $C_{13}H_{22}Si$ $(M+H)^+$ 207.1564 found 207.1560.

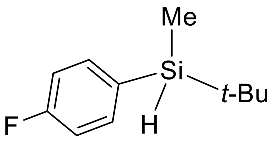

S1q

tert-butyl(4-fluorophenyl)(methyl)silane (**S1q**)

S1q was prepared according to Method **3A**.

4-Bromofluorobenzene (1.75g, 10 mmol), *n*-BuLi (5 mL, 12 mmol, 2.4 M in hexane) and **1k** (2 mL, 15 mmol) in dry THF (30 mL) at -78 °C, slowly warmed up to room temperature and stirred for 12 h afforded **S1q** as a colorless oil (1.07 g, 55%).

- R_f = 0.8 (Petroleum Ether).
- ^{1}H NMR (400 MHz, $CDCl_3$) δ 7.51 - 7.47 (m, 2H), 7.07 - 7.02 (m, 2H), 4.14 (q, *J* = 4 Hz, 1H), 0.92 (s, 9H), 0.33 (d, *J* = 4 Hz, 3H).
- ^{13}C NMR (100 MHz, $CDCl_3$) δ 163.9 (d, *J* = 240 Hz), 136.9 (d, *J* = 10 Hz), 130.9 (d, *J* = 10 Hz), 114.8 (d, *J* = 20 Hz), 26.7, 16.6, -8.3.
- IR (neat) cm $^{-1}$ 2927, 2856, 2112, 1587, 1260, 813.
- HRMS calcd for $C_{11}H_{17}FSi$ $(M+H)^+$ 219.0976 found 219.0981.

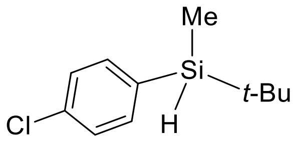

S1r

tert-butyl(4-chlorophenyl)(methyl)silane (**S1r**)

S1r was prepared according to Method **3A**.

4-Bromochlorobenzene (1.91g, 10 mmol), *n*-BuLi (5 mL, 12 mmol, 2.4 M in hexane) and **1k** (2 mL, 15 mmol) in dry THF (30 mL) at -78 °C, slowly warmed up to room temperature and stirred for 12 h afforded **S1r** as a colorless oil (1.31 g, 62%).

- R_f = 0.8 (Petroleum Ether).
- ^{1}H NMR (400 MHz, $CDCl_3$) δ 7.45 (d, *J* = 8 Hz, 2H), 7.33 (d, *J* = 8 Hz, 2H), 4.12 (q, *J* = 4 Hz, 1H), 0.92 (s, 9H), 0.32 (d, *J* = 4 Hz, 3H).
- ^{13}C NMR (100 MHz, $CDCl_3$) δ 136.3, 135.6, 133.8, 127.9, 26.7, 16.5, -8.5.
- IR (neat) cm $^{-1}$ 2927, 2113, 1576, 1466, 1252,1082, 832.
- HRMS calcd for $C_{11}H_{17}ClSi$ $(M+H)^+$ 213.0681 found 213.0677.

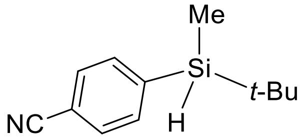

S1s

4-(tert-butyl(methyl)silyl)benzonitrile (**S1s**)

S1s was prepared according to Method **3A**.

4-Bromobenzonitrile (1.82g, 10 mmol), *n*-BuLi (5 mL, 12 mmol, 2.4 M in hexane) and **1k** (2 mL, 15 mmol) in dry THF (30 mL) at -78 °C, slowly warmed up to room temperature and stirred for 12 h afforded **S1s** as a colorless oil (1.72 g, 85%).

- R_f = 0.2 (Petroleum Ether: Ethyl Acetate = 30 : 1).
- ^{1}H NMR (400 MHz, $CDCl_3$) δ 7.77 - 7.52 (m, 4H), 4.16 (q, *J* = 4 Hz, 1H), 0.93 (s, 9H), 0.36 (d, *J* = 4 Hz, 3H).
- ^{13}C NMR (100 MHz, $CDCl_3$) δ 142.4, 135.4, 130.8, 118.9, 112.9, 26.7, 16.6, -8.7.
- IR (neat) cm $^{-1}$ 2929, 2228, 2117, 1465, 1386, 1099, 814.
- HRMS calcd for $C_{12}H_{17}NSi$ $(M+H)^+$ 204.1203 found 204.1206.

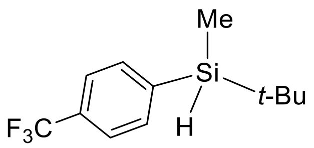

S1t

tert-butyl(methyl)(4-(trifluoromethyl)phenyl)silane (**S1t**)

S1t was prepared according to Method **3A**

4-Bromobenzotrifluoride (2.25 g, 10 mmol), *n*-BuLi (5 mL, 12 mmol, 2.4 M in hexane) and **1k** (2 mL, 15 mmol), in dry THF (30 mL) at -78 °C, slowly warmed up to room temperature and stirred for 12 h afforded **S1t** as a colorless oil (1.24 g, 51%).

- R_f = 0.9 (Petroleum Ether)

➢ ^{1}H NMR (400 MHz, $CDCl_3$) δ 7.66 (d, *J* = 8.0 Hz, 2H), 7.61 (d, *J* = 8.0 Hz, 2H), 4.19 (q, *J* = 4 Hz, 1H), 0.96 (s, 9H), 0.38 (d, *J* = 3.7 Hz, 3H).

➢ ^{13}C NMR (150 MHz, $CDCl_3$) δ 140.6, 135.3, 131.2 (q, *J* = 32 Hz), 124.2 (q, *J* = 272 Hz), 124.1 (q, *J* = 5 Hz), 124.14, 124.12, 123.33, 121.53, 26.7, 16.6, -8.6.

➢ IR (neat) cm $^{-1}$ 2929, 2858, 2115, 1610, 1466, 1322, 1128, 1059, 820, 764.

➢ HRMS calcd for $C_{12}H_{17}F_3Si$ $(M+Na)^+$ 269.0944 found 269.0941.

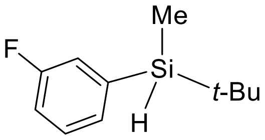

S1u

tert-butyl(3-fluorophenyl)(methyl)silane (**S1u**)

S1u was prepared according to Method **3A**.

3-Bromofluorobenzene (1.75g, 10 mmol), *n*-BuLi (5 mL, 12 mmol, 2.4 M in hexane) and **1k** (2 mL, 15 mmol) in dry THF (30 mL) at -78 °C, slowly warmed up to room temperature and stirred for 12 h afforded **S1u** as a colorless oil (1.23 g, 63%).

➢ R*f* = 0.8 (Petroleum Ether).

➢ ^{1}H NMR (400 MHz, $CDCl_3$) δ 7.34 - 7.25 (m, 2H), 7.20 - 7.18 (m, 1H), 7.06 - 7.01 (m, 1H), 4.12 (q, *J* = 4 Hz, 1H), 0.92 (s, 9H), 0.32 (d, *J* = 4 Hz, 3H).

➢ ^{13}C NMR (150 MHz, $CDCl_3$) δ 162.4 (d, *J* = 248 Hz), 138.5 (d, *J* = 4.0 Hz), 130.6 (d, *J* = 3 Hz), 129.37 (d, *J* = 7 Hz), 121.3 (d, *J* = 18 Hz), 116.2 (d, *J* = 21 Hz), 26.8, 16.6, -8.5.

➢ IR (neat) cm $^{-1}$ 2929, 2857, 2115, 1573, 1216, 866, 823.

➢ HRMS calcd for $C_{11}H_{17}FSi$ $(M+H)^+$ 197.1156 found 197.1156.

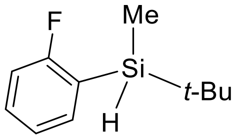

S1v

tert-butyl(2-fluorophenyl)(methyl)silane (**S1v**)

S1v was prepared according to Method **3A**.

2-Bromofluorobenzene (1.75g, 10 mmol), *n*-BuLi (5 mL, 12 mmol, 2.4 M in hexane) and **1k** (2 mL, 15 mmol) in dry THF (30 mL) at -78 °C, slowly warmed up to room temperature and stirred for 12 h afforded **S1v** as a colorless oil (1.31 g, 67%)

➢ R*f* = 0.8 (Petroleum Ether).

➢ ^{1}H NMR (400 MHz, $CDCl_3$) δ 7.51 - 7.47 (m, 1H), 7.42 - 7.36 (m, 1H), 7.16 (t, *J* = 8 Hz, 1H), 7.03 (t, *J* = 8 Hz, 1H), 4.60 - 4.00 (m, 1H), 1.00 (s, 9H), 0.42 (dd, *J* = 4, 1.0 Hz, 1H).

➢ ^{13}C NMR (150 MHz, $CDCl_3$) δ 167.1 (d, *J* = 240 Hz), 137.2 (d, *J* = 11 Hz), 131.6 (d, *J* = 8 Hz), 123.7 (d, *J* = 3 Hz), 121.70 (d, *J* = 30.9 Hz), 114.78 (d, *J* = 25.9 Hz), 26.95, 16.85, -8.14 (d, *J* = 2.8 Hz).

➢ IR (neat) cm $^{-1}$ 2929, 2857, 2116, 1435, 1254, 1078, 878, 829.

- HRMS calcd for $C_{11}H_{17}FSi$ $(M+H)^+$ 197.1156 found 197.1155.

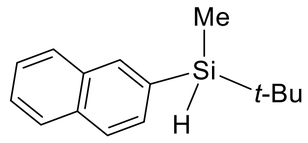

S1w

tert-butyl(methyl)(naphthalen-2-yl)silane (**S1w**)

S1w was prepared according to Method **3A**.

2-Bromonaphthalene (2.07g, 10 mmol), *n*-BuLi (5 mL, 12 mmol, 2.4 M in hexane) and **1k** (2 mL, 15 mmo) in dry THF (30 mL) at -78 °C, slowly warmed up to room temperature and stirred for 12 h afforded **S1w** as a white solid (2.09 g, 92%).

- mp: 49.8 - 51.3 °C.
- R_f = 0.9 (Petroleum Ether).
- ^{1}H NMR (400 MHz, $CDCl_3$) δ 8.12 (s, 1H), 7.95 - 7.86 (m, 3H), 7.68 (d, J = 8.0 Hz, 1H), 7.55 (dd, J = 6, 4 Hz, 2H), 4.36 (q, J = 4 Hz, 1H), 1.06 (s, 9H), 0.50 (d, J = 4 Hz, 3H).
- ^{13}C NMR (100 MHz, $CDCl_3$) δ 135.9, 133.8, 133.1, 132.9, 131.2, 128.1, 127.7, 126.7, 126.4, 125.9, 26.9, 16.8, -8.3.
- IR (neat) cm $^{-1}$ 3046, 2923, 2852, 2106, 1457, 1085, 874, 774.
- HRMS calcd for $C_{15}H_{20}Si$ $(M+H)^+$ 251.1226 found 251.1225.

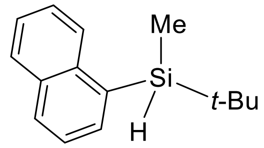

S1x

tert-butyl(methyl)(naphthalen-1-yl)silane (**S1x**)

S1x was prepared according to Method **3A**.

1-Bromonaphthalene (2.07g, 10 mmol), *n*-BuLi (5 mL, 12 mmol, 2.4 M in hexane) and **1k** (2 mL, 15 mmol) in dry THF (30 mL) at -78 °C, slowly warmed up to room temperature and stirred for 12 h afforded **S1x** as a colorless oil (2.14 g, 94%)

- R_f = 0.8 (Petroleum Ether).
- ^{1}H NMR (400 MHz, $CDCl_3$) δ 8.13 (d, J = 8 Hz, 1H), 7.76 - 7.69 (m, 2H), 7.60 - 7.58 (m, 1H), 7.40 - 7.31 (m, 3H), 4.60 (q, J = 4 Hz, 1H), 0.87 (s, 9H), 0.37 (d, J = 4 Hz, 3H).
- ^{13}C NMR (100 MHz, $CDCl_3$) δ 137.6, 135.0, 134.1, 123.3, 129.9 128.8, 128.6, 125.6, 125.4, 124.9, 27.5, 17.6, -7.2.
- IR (neat) cm $^{-1}$ 3051, 2927, 2113, 1464, 1251, 885, 791.
- HRMS calcd for $C_{15}H_{20}Si$ $(M+H)^+$ 229.1407 found 229.1409.

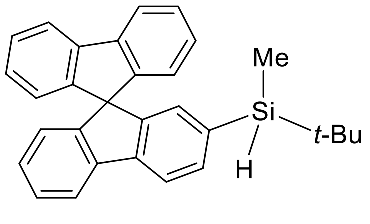

S1y

9,9'-spirobi[fluoren]-3-yl(tert-butyl)(methyl)silane (**S1y**)

S1y was prepared according to Method **3A**.

2-Bromo-9,9'-spirobi[9H-fluorene] (3.95g, 10 mmol), *n*-BuLi (5 mL, 12 mmol, 2.4 M in hexane) and **1k** (2 mL, 15 mmol) in dry THF (30 mL) at -78 °C, slowly warmed up to room temperature and stirred for 12 h afforded **S1y** as a white solid (3.57g, 86%).

- mp: 51.3 - 51.9 °C.
- R_f = 0.5 (Petroleum Ether).
- ^{1}H NMR (400 MHz, $CDCl_3$) δ 7.89 - 7.82 (m, 4H), 7.55 (dd, *J* = 8, 1 Hz, 1H), 7.40 - 7.33 (m, 3H), 7.11 (td, *J* = 8, 1 Hz, 3H), 6.89 (s, 1H), 6.72 (t, *J* = 8 Hz, 3H), 3.98 (q, *J* = 4 Hz, 1H), 0.78 (s, 9H), 0.21 (d, *J* = 4 Hz, 3H).
- ^{13}C NMR (100 MHz, $CDCl_3$) δ 149.1, 148.8, 147.8, 142.9, 141.7, 141.7, 141.5, 135.1, 134.2, 130.7, 128.0, 127.7, 127.6, 124.0, 120.1, 119.9, 119.2, 65.9, 26.7, 16.5, -8.5.
- IR (neat) cm $^{-1}$ 2950, 2853, 2110, 1446, 1260, 750.
- HRMS calcd for $C_{30}H_{28}Si$ $(M+H)^+$ 417.2033 found 417.2037.

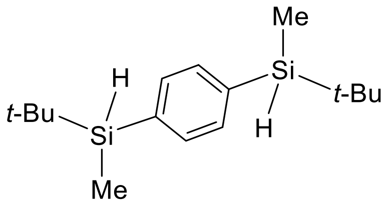

S1aa

1,4-bis(tert-butyl(methyl)silyl)benzene (**S1aa**)

S1aa was prepared according to Method **3A**.

1,4-Dibromobenzene (2.36g, 10 mmol), *n*-BuLi (10 mL, 24 mmol, 2.4 M in hexane) and **1k** (4 mL, 30 mmol) in dry THF (50 mL) at -78 °C, slowly warmed up to room temperature and stirred for 12 h afforded **S1aa** as a white solid (1.36g, 49%).

- mp: 46.1 - 58.0 °C.
- R_f = 0.8 (Petroleum Ether).
- ^{1}H NMR (400 MHz, $CDCl_3$) δ 7.50 (s, 2H), 4.13 (q, *J* = 4 Hz, 1H), 0.93 (s, 9H), 0.33 (d, *J* = 4 Hz, 3H).
- ^{13}C NMR (100 MHz, $CDCl_3$) δ 136.5, 134.2, 26.9, 16.7, -8.5.
- IR (neat) cm $^{-1}$ 2927, 2109, 1464, 1380, 1255, 1133, 1008, 821.
- HRMS calcd for $C_{16}H_{30}Si_2$ $(M+H)^+$ 279.1959 found 279.1955.

Method 3B:

benzofuran-2-yl(tert-butyl)(methyl)silane (**S1z**)

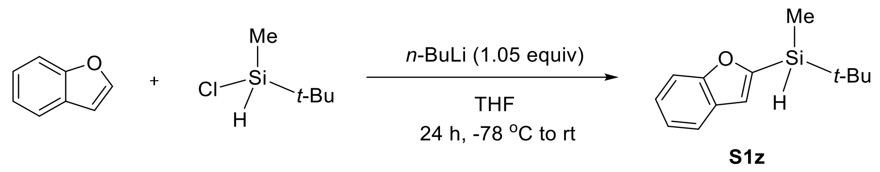

To a 100 mL round-bottom flask charged with benzofuran (1.18 g, 10 mmol, 1.0 equiv.) in dry THF (30 mL) under an inert atmosphere of argon at -78 °C, freshly titrated *n*-BuLi (5 mL, 12 mmol, 2.4 M, 1.2 equiv.) was added carefully and the mixture was stirred for 3 h at -78 °C. Then **1k** (2 mL, 15 mmol, 1.5 equiv.) was added at -78 °C and the resulting mixture was slowly warmed up to room temperature and stirred for 12 h. Finally, the reaction was quenched with aq. NH_4Cl (30 mL). The organic layers were separated and the aqueous layer was extracted with EtOAc (100 mL × 3). The combined organic layers were washed with sat. aq. NaCl, dried over Na_2SO_4 and concentrated under reduced pressure. Purification by column chromatography on silica gel (Petroleum Ether: 100%) afforded the desired product **S1z** as a colorless oil (1.73g, 80%).

- R_f = 0.9 (Petroleum Ether).
- ^{1}H NMR (400 MHz, $CDCl_3$) δ 7.59 (d, *J* = 8 Hz, 1H), 7.52 (d, *J* = 8 Hz, 1H), 7.28 (t, *J* = 8 Hz, 1H), 7.21 (t, *J* = 8 Hz, 1H), 7.06 (s, 1H), 4.24 (q, *J* = 4 Hz, 1H), 1.04 (s, 9H), 0.40 (d, *J* = 4 Hz, 3H).
- ^{13}C NMR (100 MHz, $CDCl_3$) δ 159.2, 158.2, 127.8, 124.5, 122.4, 121.0, 118.6, 111.3, 26.7, 16.5, -9.0.
- IR (neat) cm $^{-1}$ 3006, 2951, 2857, 2125, 1525, 1470, 1275, 1253, 920, 810, 764.
- HRMS calcd for $C_{13}H_{18}OSi$ $(M+H)^+$ 219.1200 found 219.1203.

Method 3C:

Methyl(phenyl)(p-tolyl)silane (**S1ab**)

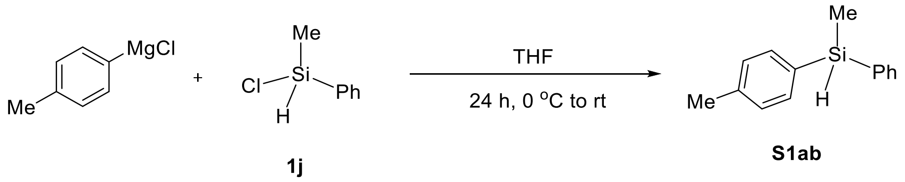

To a 100 mL round-bottom flask charged with **1j** (1.56 g, 10 mmol, 1.0 equiv.) in dry THF (30 mL) under an inert atmosphere of argon at 0 °C, then 4-Methylphenylmagnesium chloride (7.5 mL, 15 mmol, 1.5 equiv. 2 M in THF) was added at 0 °C and the resulting mixture was slowly warmed up to room temperature and stirred for 12 h. Finally, the reaction was quenched with aq. NH_4Cl (30 mL). The organic layers were separated and the aqueous layer was extracted with EtOAc (100 mL × 3). The combined organic layers were washed with sat. aq. NaCl, dried over Na_2SO_4 and

concentrated under reduced pressure. Purification by column chromatography on silica gel (Petroleum Ether: 100%) afforded the desired product **S1ab** as a colorless oil (1.76g, 83%).

- R_f = 0.8 (Petroleum Ether).
- ^{1}H NMR (400 MHz, $CDCl_3$) δ 7.58 - 7.55 (m, 2H), 7.48 - 7.46 (m, 2H), 7.40 - 7.34 (m, 3H), 7.20 (d, *J* = 8 Hz, 2H), 4.94 (q, *J* = 4 Hz, 1H), 2.37 (s, 3H), 0.63 (d, *J* = 4 Hz, 3H).
- ^{13}C NMR (150 MHz, $CDCl_3$) δ 139.5, 135.6, 134.9, 134.8, 131.6, 129.4, 128.8, 127.9, 21.5, -4.9.
- IR (neat) cm $^{-1}$ 3067, 2919, 2116, 1908, 1601, 1427, 1109, 873, 831.
- HRMS calcd for $C_{14}H_{16}Si$ $(M+H)^+$ 213.1094 found 213.1098.

Synthesis of 3e [9-11]

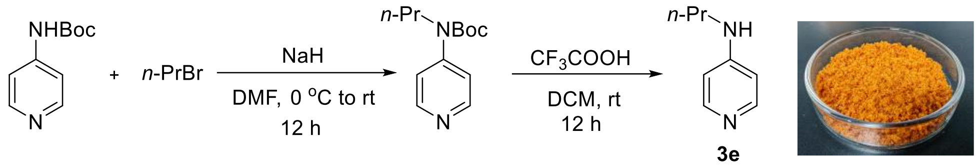

To a suspension of NaH (5.8 g, 145 mmol, 1.5 equiv., 60% in mineral oil) in DMF (400 mL) under argon atmosphere was added *tert*-butyl pyridin-4-ylcarbamate (18.8 g, 97 mmol, 1.0 equiv.) slowly at 0 °C. The mixture was stirred for 30 min before adding 1-bromopropane (17.9 g, 145 mmol, 1.5 equiv.). After 12 h, the reaction was quenched at 0 °C by careful addition of H_2O (100 mL). The mixture was extracted with EtOAc (3 × 100 mL). The combined organic layers were dried over Na_2SO_4 and concentrated under reduced pressure. The crude product was used without purification in the next step.

To a solution of the crude product in CH_2Cl_2 (100 mL) was added trifluoroacetic acid (100 mL) slowly. The mixture was stirred at room temperature for 12 h. The reaction was cooled down to 0 °C, quenched with sat. aq. $NaHCO_3$ and adjusted pH to 9-10 with aq. NaOH (1 M). The organic layers were separated and the aqueous layer was extracted with EtOAc (100 mL × 3). The combined organic layers were washed with sat. aq. NaCl, dried over Na_2SO_4 and concentrated under reduced pressure to afford **3e** as an orange solid (10.2 g, 85%).

Synthesis of Silylethers 4

Method 6A:

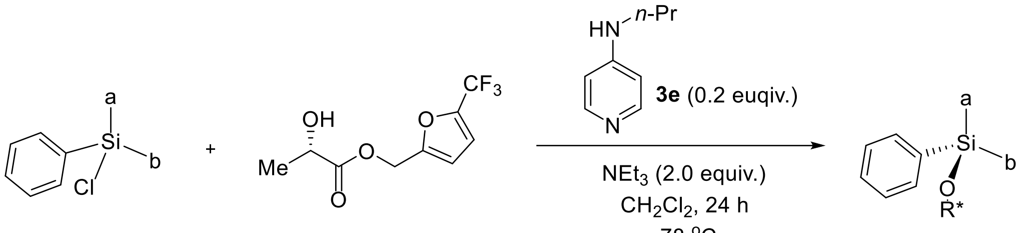

1a-j **2i** **4j-q** and **4s**

To a 10 mL round-bottom flask charged with **2i** (48 mg, 0.2 mmol, 1.0 equiv.) in dry CH_2Cl_2 (2 mL) were added chlorosilanes (0.24 mmol, 1.2 equiv.), **3e** (6 mg, 0.04 mmol, 20 mol%) and NEt_3 (56 μL, 0.4 mmol, 2.0 equiv.) under an inert atmosphere of argon at -78 °C. The mixture was stirred for 24 h at -78 °C before removing the solvent under reduced pressure. Purification by column chromatography on silica gel (gradient eluent: Petroleum Ether to Petroleum Ether /EtOAc = 30:1) afforded the desired product.

Method 6B:

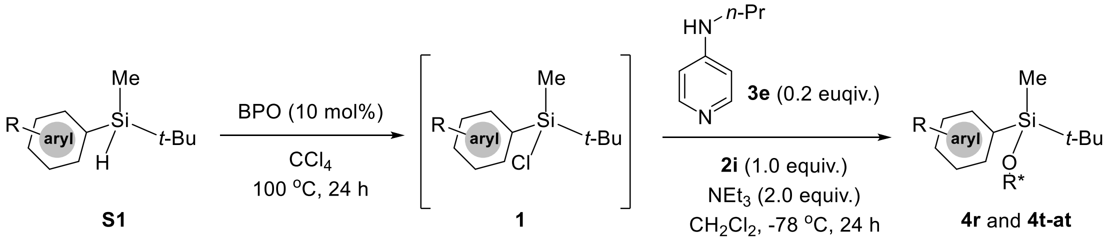

S1 **1** **4r** and **4t-at**

To a 10 mL round-bottom flask charged with silanes (0.3 mmol) and CCl_4 (3 mL) was added benzoyl peroxide (8 mg, 0.03 mmol). The mixture was refluxed for 21 h followed by stirring for 3 h at room temperature before removing the solvent under reduced pressure. The crude product was used without purification in the next step.

To a 10 mL round-bottom flask charged with **2i** (48 mg, 0.2 mmol, 1.0 equiv.) in dry CH_2Cl_2 (2 mL) were added the crude chlorosilane **1** (1.2 equiv.), **3e** (6 mg, 0.04 mmol, 20 mol%) and NEt_3 (56 μL, 0.4 mmol, 2.0 equiv.) under an inert atmosphere of argon at -78 °C. The mixture was stirred for 24 h at -78 °C before removing the solvent under reduced pressure. Purification by column chromatography on silica gel (gradient eluent: Petroleum Ether to Petroleum Ether /EtOAc = 3:1) afforded the desired product.

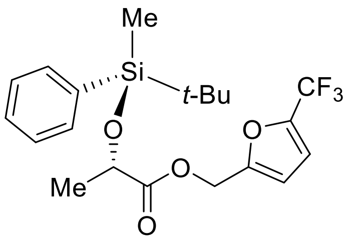

4i

(5-(trifluoromethyl)furan-2-yl)methyl(2S)-2-((tert-butyl(methyl)(phenyl)silyl)oxy)propanoate (**4i**)

4i was prepared according to Method **6A**.

2i (48 mg, 0.2 mmol), **3e** (6 mg, 0.04 mmol), NEt_3 (56 μL, 0.4 mmol) and **1a** (51 mg, 0.24 mmol) in dry CH_2Cl_2 (2 mL) at -78 °C for 24 h afforded **4i** as a colorless oil (76 mg, 95%, *dr* = 93:7).

- R_f = 0.6 (Petroleum Ether: Ethyl Acetate = 8 : 1).
- ^{1}H NMR (400 MHz, $CDCl_3$) δ 7.53 (dd, *J* = 8, 4 Hz, 2H), 7.52 - 7.28 (m, 3H), 6.75 (d, *J* = 4 Hz, 1H), 6.47 (d, *J* = 4 Hz, 1H), 5.11 (9, *J* = 12 Hz, 2H), 4.34 (q, *J* = 8 Hz, 1H), 1.44 (d, *J* = 8 Hz, 3H), 0.89 (s, 9H), 0.31 (s, 3H).
- ^{13}C NMR (100 MHz, $CDCl_3$) δ 173.4, 152.0, 142.4 (q, *J* = 50 Hz), 135.4, 134.4, 129.6, 127.6, 118.9 (q, *J* = 270 Hz), 112.3 (q, *J* = 10 Hz), 111.3, 68.3, 57.6, 25.6, 21.2, 18.2, -7.3.
- ^{29}Si NMR (80 MHz, $CDCl_3$) δ 9.94.
- ^{19}F NMR (376 MHz, $CDCl_3$) δ -64.24.
- IR (neat) cm $^{-1}$ 2957, 2858, 1759, 1741, 1317, 1275, 1130, 764.
- HRMS calcd for $C_{20}H_{25}F_3O_4Si$ $(M+Na)^+$ 437.1366 found 437.1362.
- $[\alpha]^{25}_D$ = -60 (*c* = 1.0, $CHCl_3$).

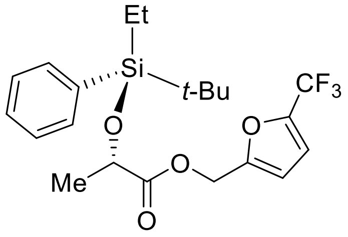

4j

(5-(trifluoromethyl)furan-2-yl)methyl(2S)-2-((tert-butyl(ethyl)(phenyl)silyl)oxy)propanoate (**4j**)

4j was prepared according to Method **6A**.

2i (48 mg, 0.2 mmol), **3e** (6 mg, 0.04 mmol), NEt_3 (56 μL, 0.4 mmol) and **1b** (55 mg, 0.24 mmol) in dry CH_2Cl_2 (2 mL) at -78 °C for 24 h afforded **4j** as a colorless oil (78 mg, 92%, *dr* = 87:13).

- R_f = 0.7 (Petroleum Ether: Ethyl Acetate = 8 : 1).
- ^{1}H NMR (400 MHz, $CDCl_3$) δ 7.55 - 7.53 (m, 2H), 7.41 - 7.31 (m, 3H), 6.75 (d, *J* = 4 Hz, 1H), 6.48 (d, *J* = 4 Hz, 1H), 5.17 - 5.09 (m, 2H), 4.47 (q, *J* = 8 Hz, 1H), 1.48 (d, *J* = 8 Hz, 3H), 0.99 - 0.93 (m, 5H), 0.89 (s, 9H).
- ^{13}C NMR (101 MHz, $CDCl_3$) δ 173.5, 152.0, 142.3 (q, *J* = 43 Hz), 134.7, 134.3, 129.4, 127.6, 118.8 (q, *J* = 266 Hz) 112.3 (q, *J* = 3 Hz), 111.3, 68.6, 57.6, 26.1, 21.6, 18.7, 7.2, 2.4.
- ^{29}Si NMR (80 MHz, $CDCl_3$) δ 8.65.
- IR (neat) cm $^{-1}$ 2930, 2857, 1760, 1616, 1317, 1131, 1107, 975.
- HRMS calcd for $C_{21}H_{27}F_3O_4Si$ $(M+Na)^+$ 451.1523 found 451.1526.

- $[\alpha]^{25}_D$ = -16 (c = 1.0, $CHCl_3$).

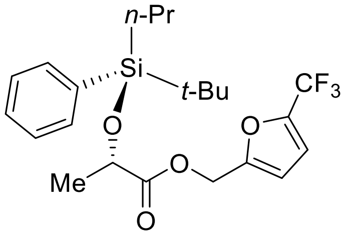

4k

(5-(trifluoromethyl)furan-2-yl)methyl(2S)-2-((tert-butyl(phenyl)(propyl)silyl)oxy)propanoate (**4k**)

4k was prepared according to Method **6A**.

2i (48 mg, 0.2 mmol), **3e** (6 mg, 0.04 mmol), NEt_3 (56 μL, 0.4 mmol) and **1c** (58 mg, 0.24 mmol) in dry CH_2Cl_2 (2 mL) at -78 °C for 24 h afforded **4k** as a colorless oil (83 mg, 94%, *dr* = 86:14).

- R_f = 0.7 (Petroleum Ether: Ethyl Acetate = 8 : 1).
- ^1H NMR (400 MHz, $CDCl_3$) δ 7.55 - 7.52 (m, 2H), 7.36 (d, J = 8 Hz, 3H), 6.76 (d, J = 4 Hz, 1H), 6.48 (d, J = 4 Hz, 1H), 5.17 - 5.08 (m, 2H), 4.46 (q, J = 8 Hz, 1H), 1.48 (d, J = 8 Hz, 3H), 1.35 - 1.28 (m, 2H), 1.01 - 0.92 (m, 5H), 0.89 (s, 9H).
- ^{13}C NMR (100 MHz, $CDCl_3$) δ 173.5, 152.0, 142.3 (q, J = 43 Hz), 134.7, 129.4, 127.6, 118.8 (q, J = 265 Hz), 112.3 (q, J = 3 Hz), 111.3, 68.7, 57.6, 26.5, 26.1, 25.7, 21.6, 18.7, 17.2, 13.6.
- ^{29}Si NMR (80 MHz, $CDCl_3$) δ 8.15.
- IR (neat) cm^{-1} 3005, 2958, 2859, 2707, 2318, 1760, 1318, 1275, 1134, 752.
- HRMS calcd for $C_{22}H_{29}F_3O_4Si$ $(M+Na)^+$ 465.1679 found 465.1676.
- $[\alpha]^{25}_D$ = -22.4 (c = 0.5, $CHCl_3$).

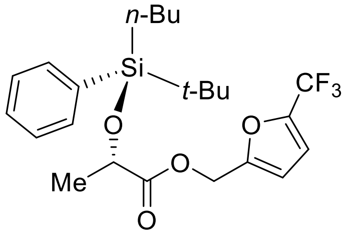

4l

(5-(trifluoromethyl)furan-2-yl)methyl(2S)-2-((tert-butyl(butyl)(phenyl)silyl)oxy)propanoate (**4l**)

4l was prepared according to Method **6A**.

2i (48 mg, 0.2 mmol), **3e** (6 mg, 0.04 mmol), NEt_3 (56 μL, 0.4 mmol) and **1d** (62 mg, 0.24 mmol) in dry CH_2Cl_2 (2 mL) at -78 °C for 24 h afforded **4l** as a colorless oil (87 mg, 95%, *dr* = 84:16).

- R_f = 0.6 (Petroleum Ether: Ethyl Acetate = 8 : 1).
- ^1H NMR (400 MHz, $CDCl_3$) δ 7.55 - 7.53 (m, 2H), 7.41 - 7.31 (m, 3H), 6.76 - 6.75 (m, 1H), 6.49 (d, J = 4 Hz, 1H), 5.12 (q, J = 12 Hz, 2H), 4.48 (d, J = 4 Hz, 1H), 1.48 (d, J = 8 Hz, 3H), 1.39 - 1.27 (m, 4H), 0.97 - 0.84 (m, 14H).
- ^{13}C NMR (100 MHz, $CDCl_3$) δ 173.5, 152.0, 151.9, 142.3 (q, J = 30 Hz), 134.7, 129.4,127.5, 118.8 (q, J = 260 Hz), 112.3 (q, J = 10 Hz), 111.3, 68.7, 57.6, 26.9, 26.1, 25.7, 21.6, 18.8, 13.6, 10.5.
- ^{29}Si NMR (80 MHz, $CDCl_3$) δ 7.66.
- IR (neat) cm^{-1} 2930, 2859, 1741, 1760, 1317, 1275, 1268, 1132, 764.

- HRMS calcd for $C_{23}H_{31}F_3O_4Si$ $(M+Na)^+$ 479.1836 found 479.1838.
- $[\alpha]^{25}_D$ = -35.2 (*c* = 0.25, $CHCl_3$).

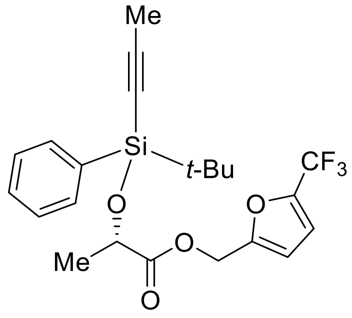

4m

(5-(trifluoromethyl)furan-2-yl)methyl(2S)-2-((tert-butyl(phenyl)(prop-1-yn-1-yl)silyl)oxy)propanoate (**4m**)

4m was prepared according to Method **6A**.

2i (48 mg, 0.2 mmol), **3e** (6 mg, 0.04 mmol), NEt_3 (56 μL, 0.4 mmol) and **1e** (57 mg, 0.24 mmol) in dry CH_2Cl_2 (2 mL) at -78 °C for 24 h afforded **4m** as a colorless oil (78 mg, 90%, *dr* = 50:50).

- R_f = 0.5 (Petroleum Ether: Ethyl Acetate = 8 : 1).
- ^1H NMR (400 MHz, $CDCl_3$) δ 7.68 - 7.76 (m, 2H), 7.41 - 7.29 (m, 3H), 6.76 (d, *J* = 4 Hz, 1H), 6.49 (d, *J* = 4 Hz, 1H), 5.13 (q, *J* = 12 Hz, 2H), 4.58 (q, *J* = 8 Hz, 1H), 1.98 (s, 3H), 1.50 (d, *J* = 8 Hz, 3H), 0.95 (s, 9H).
- ^{13}C NMR (100 MHz, $CDCl_3$) δ 173.2, 152.1, 142.3 (q, *J* = 42 Hz), 134.9, 133.5, 132.8, 129.9, 118.8 (q, *J* = 266 Hz), 111.3 (q, *J* = 3 Hz), 111.0, 106.5, 77.8, 69.1, 57.6, 25.30, 21.0, 18.4, 4.9.
- IR (neat) cm^{-1} 2919, 2850, 2181, 1761, 1566, 1179, 1132, 799.
- HRMS calcd for $C_{22}H_{25}F_3O_4Si$ $(M+Na)^+$ 461.1366 found 461.1370.

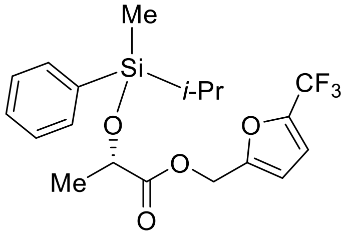

4o

(5-(trifluoromethyl)furan-2-yl)methyl(2S)-2-((isopropyl(methyl)(phenyl)silyl)oxy)propanoate (**4o**)

4o was prepared according to Method **6A**.

2i (48 mg, 0.2 mmol), **3e** (6 mg, 0.04 mmol), NEt_3 (56 μL, 0.4 mmol) and **1g** (48 mg, 0.24 mmol) in dry CH_2Cl_2 (2 mL) at -78 °C for 24 h afforded **4o** as a colorless oil (76 mg, 95%, *dr* = 50:50).

- R_f = 0.6 (Petroleum Ether: Ethyl Acetate = 8 : 1).
- ^1H NMR (400 MHz, $CDCl_3$) δ 7.55 - 7.54 (m, 2H), 7.41 - 7.37 (m, 3H), 6.76 (d, *J* = 4 Hz, 1H), 6.45 (d, *J* = 4 Hz, 1H), 5.12 - 4.99 (m, 2H), 4.33 (q, *J* = 8 Hz, 1H), 1.40 (d, *J* = 8 Hz, 3H), 1.18 -1.07 (m, 1H), 1.01 (d, *J* = 8 Hz, 3H), 0.92 (d, *J* = 8 Hz, 3H), 0.34 (s, 3H).
- ^{13}C NMR (100 MHz, $CDCl_3$) δ 173.3, 151.9, 142.3 (q, *J* = 42 Hz), 134.1, 129.7, 127.8, 118.8 (q, *J* = 266 Hz), 112.3 (q, *J* = 3 Hz), 111.2, 68.3, 57.6, 21.1, 16.8, 16.6, 13.9, -6.1.
- IR (neat) cm^{-1} 2956, 2867, 1757, 1616, 1566, 1317, 1275, 1106, 791, 763.

- HRMS calcd for $C_{19}H_{23}F_3O_4Si$ $(M+Na)^+$ 423.1210 found 423.1207.

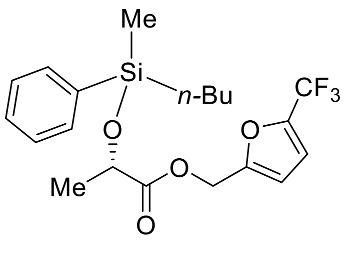

4p

<u>(5-(trifluoromethyl)furan-2-yl)methyl(2S)-2-((butyl(methyl)(phenyl)silyl)oxy)propanoate</u> (**4p**)

4p was prepared according to Method **6A**.

2i (48 mg, 0.2 mmol), **3e** (6 mg, 0.04 mmol), NEt_3 (56 μL, 0.4 mmol) and **1h** (51 mg, 0.24 mmol) in dry CH_2Cl_2 (2 mL) at -78 °C for 24 h afforded **4p** as a colorless oil (74 mg, 90%, *dr* = 50:50).

- R_f = 0.6 (Petroleum Ether: Ethyl Acetate = 8 : 1).
- ^{1}H NMR (400 MHz, $CDCl_3$) δ 7.55 - 7.53 (m, 2H), 7.39 - 7.34 (m, 3H), 6.75 (d, *J* = 4 Hz, 1H), 6.44 (d, *J* = 4 Hz, 1H), 5.10 - 5.0 (m, 2H), 4.31 (q, *J* = 8 Hz, 1H), 1.38 (d, *J* = 8 Hz, 3H), 1.35 - 1.31 (m, 4H), 0.89 - 0.84 (m, 5H), 0.37 (s, 3H).
- ^{13}C NMR (100 MHz, $CDCl_3$) δ 173.3, 152.0, 142.3 (q, *J* = 42 Hz), 136.4, 133.8, 129.8, 127.8, 118.8 (q, *J* = 266 Hz), 112.3 (q, *J* = 3 Hz), 111.2, 68.2, 57.6, 26.3, 25.1, 21.2, 15.0, 13.7, -3.5.
- IR (neat) cm $^{-1}$ 2926, 1959, 2873, 1756, 1318, 1260, 1107, 800, 764.
- HRMS calcd for $C_{20}H_{25}F_3O_4Si$ $(M+Na)^+$ 437.1366 found 437.1363.

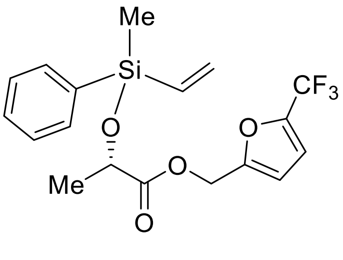

4q

<u>(5-(trifluoromethyl)furan-2-yl)methyl(2S)-2-((methyl(phenyl)(vinyl)silyl)oxy)propanoate</u> (**4q**)

4q was prepared according to Method **6A**.

2i (48 mg, 0.2 mmol), **3e** (6 mg, 0.04 mmol), NEt_3 (56 μL, 0.4 mmol) and **1i** (42 μL, 0.24 mmol) in dry CH_2Cl_2 (2 mL) at -78 °C for 24 h afforded **4q** as a colorless oil (67 mg, 88%, *dr* = 50:50).

- R_f = 0.6 (Petroleum Ether: Ethyl Acetate = 8 : 1).
- ^{1}H NMR (400 MHz, $CDCl_3$) δ 7.51 - 7.50 (m, 2H), 7.35 - 7.30 (m, 3H), 6.69 - 6.67 (m, 1H), 6.38 - 6.37 (m, 1H), 6.23 - 6.18 (m, 1H), 6.09 - 6.08 (m, 1H), 5.83 - 5.81 (m, 1H), 5.05 - 4.95 (m, 2H), 4.31 (q, *J* = 8 Hz, 1H), 1.35 (d, *J* = 8 Hz, 3H), 0.38 (s, 3H).
- ^{13}C NMR (100 MHz, $CDCl_3$) δ 173.1, 151.9, 142.3 (q, *J* = 42 Hz), 135.8, 135.4, 134.9, 134.0, 130.0, 127.9, 118.8 (q, *J* = 265 Hz), 112.3 (q, *J* = 3 Hz), 111.4, 68.4, 57.7, 21.1, -3.1.
- IR (neat) cm $^{-1}$ 3005, 2989, 1753, 1616, 1317, 1275, 1129, 1106, 934, 751.
- HRMS calcd for $C_{18}H_{19}F_3O_4Si$ $(M+Na)^+$ 407.0879 found 407.0882.

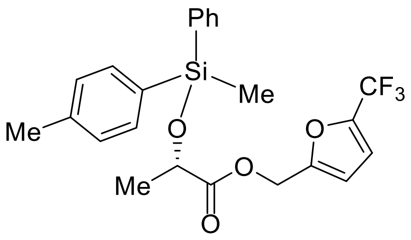

4r

(5-(trifluoromethyl)furan-2-yl)methyl (2S)-2-((methyl(phenyl)(p-tolyl)silyl)oxy)propanoate (**4r**)

4r was prepared according to Method **6B**.

S1ab (64 mg, 0.3 mmol), BPO (8 mg, 0.03 mmol), CCl_4 (3 mL), 100 °C refluxed for 21 h; then concentration followed by **2i** (48 mg, 0.2 mmol), **3e** (6 mg, 0.04 mmol) and NEt_3 (56 μL, 0.4 mmol) in dry CH_2Cl_2 (2 mL) at -78 °C for 24 h afforded **4r** as a yellow oil (72 mg, 80%, *dr* = 50:50).

- R_f = 0.6 (Petroleum Ether: Ethyl Acetate = 8 : 1).
- ^{1}H NMR (400 MHz, $CDCl_3$) δ 7.59 - 7.56 (m, 2H), 7.47 (d, J = 8 Hz, 2H), 7.41 - 7.33 (m, 3H), 7.19 - 7.17 (m, 2H), 6.76 - 6.75 (m, 1H), 6.43 - 6.42 (m, 1H), 5.07 - 4.98 (m, 2H), 4.38 (q, J = 8 Hz, 1H), 2.36 (s, 3H), 1.43 (d, J = 8 Hz, 3H), 0.63 (s, 3H).
- ^{13}C NMR (100 MHz, $CDCl_3$) δ 173.1, 151.9, 142.3 (q, J = 43 Hz), 140.1, 135.7, 134.6, 134.4, 131.7, 130.0, 128.7, 127.8, 118.8 (q, J = 266 Hz), 112.3 (q, J = 3 Hz), 111.2, 68.5, 57.7, 21.5, 21.1, -2.6.
- IR (neat) cm $^{-1}$ 2923, 1754, 1615, 1448, 1317, 1177, 1105, 975, 796.
- HRMS calcd for $C_{23}H_{23}F_3O_4Si$ $(M+Na)^+$ 471.1210 found 471.1203.

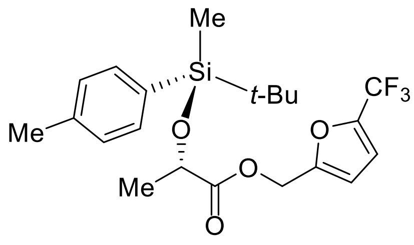

4t

(5-(trifluoromethyl)furan-2-yl)methyl(2S)-2-((tert-butyl(methyl)(p-tolyl)silyl)oxy)propanoate (**4t**)

4t was prepared according to Method **6B**.

S1a (58 mg, 0.3 mmol), BPO (8 mg, 0.03 mmol), CCl_4 (3 mL), 100 °C refluxed for 21 h; then concentration followed by **2i** (48 mg, 0.2 mmol), **3e** (6 mg, 0.04 mmol) and NEt_3 (56 μL, 0.4 mmol) in dry CH_2Cl_2 (2 mL) at -78 °C for 24 h afforded **4t** as a colorless oil (80 mg, 94%, *dr* = 92:8).

- R_f = 0.7 (Petroleum Ether: Ethyl Acetate = 8 : 1).
- ^{1}H NMR (400 MHz, $CDCl_3$) δ 7.43 (d, J = 8 Hz, 2H), 7.18 (d, J = 8 Hz, 2H), 6.75 (d, J = 4 Hz, 1H), 6.47 (d, J = 4 Hz, 1H), 5.10 (q, J = 12 Hz, 2H), 4.32 (q, J = 8 Hz, 1H), 2.36 (s, 3H), 1.43 (d, J = 8 Hz, 3H), 0.89 (s, 9H), 0.29 (s, 3H).
- ^{13}C NMR (150 MHz, $CDCl_3$) δ 173.4, 152.0, 142.3 (q, J = 42 Hz), 139.5, 134.5, 131.7, 128.5, 118.8 (q, J = 266 Hz), 112.3 (q, J = 3 Hz), 111.2, 68.2, 57.6, 25.6, 21.5, 21.2, 18.2, -7.3.
- ^{29}Si NMR (80 MHz, $CDCl_3$) δ 10.21.
- IR (neat) cm $^{-1}$ 3005, 2989, 2859, 1759, 1275, 1260, 1131, 764.

- HRMS calcd for $C_{21}H_{27}F_3O_4Si$ $(M+Na)^+$ 451.1523 found 451.1525.
- $[\alpha]^{25}_D$ = -83.4 (*c* = 1.0, $CHCl_3$).

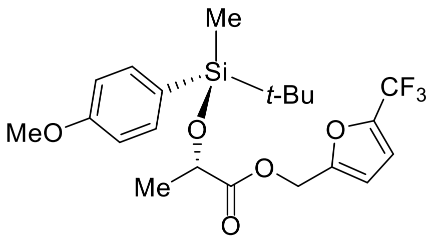

4u

<u>(5-(trifluoromethyl)furan-2-yl)methyl(2S)-2-((tert-butyl(4-methoxyphenyl)(methyl)silyl)oxy)-propanoate</u> (**4u**)

4u was prepared according to Method **6B**.

S1b (64 mg, 0.3 mmol), BPO (8 mg, 0.03 mmol), CCl_4 (3 mL), 100 °C refluxed for 21 h; then concentration followed by **2i** (48 mg, 0.2 mmol), **3e** (6 mg, 0.04 mmol) and NEt_3 (56 μL, 0.4 mmol) in dry CH_2Cl_2 (2 mL) at -78 °C stirred for 24 h afforded **4u** as a colorless oil (82 mg, 88%, *dr* = 92:8).

- R_f = 0.3 (Petroleum Ether: Ethyl Acetate = 8 : 1).
- ^{1}H NMR (400 MHz, $CDCl_3$) δ 7.46 (d, *J* = 8 Hz, 2H), 6.91 (d, *J* = 8 Hz, 2H), 6.46 (d, *J* = 4 Hz, 1H), 5.10 (q, *J* = 12 Hz, 2H), 4.31 (q, *J* = 8 Hz, 1H), 3.82 (s, 3H), 1.42 (d, *J* = 8 Hz, 3H), 0.88 (s, 9H), 0.28 (s, 3H).
- ^{13}C NMR (100 MHz, $CDCl_3$) δ 173.4, 160.8, 152.0, 142.3 (q, *J* = 42 Hz), 136.0, 126.0, 120.1, 118.8 (q, *J* = 270 Hz), 112.3, (q, *J* = 3 Hz), 111.2, 68.2, 57.6, 55.0, 25.6, 21.2, 18.3, -7.3.
- ^{29}Si NMR (80 MHz, $CDCl_3$) δ 10.14.
- IR (neat) cm $^{-1}$ 2930, 2859, 1741, 1473, 1317, 1275, 1132, 764.
- HRMS calcd for $C_{21}H_{27}F_3O_5Si$ $(M+Na)^+$ 467.1472 found 467.1474.
- $[\alpha]^{25}_D$ = -35.6 (*c* = 0.5, $CHCl_3$).

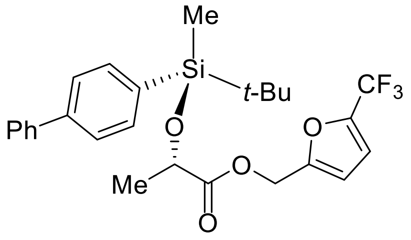

4v

<u>(5-(trifluoromethyl)furan-2-yl)methyl(2S)-2-(([1,1'-biphenyl]-4-yl(tert-butyl)(methyl)silyl)oxy)propanoate</u> (**4v**)

4v was prepared according to Method **6B**.

S1c (77 mg, 0.3 mmol), BPO (8 mg, 0.03 mmol), CCl_4 (3 mL), 100 °C refluxed for 21 h; then concentration followed by **2i** (48 mg, 0.2 mmol), **3e** (6 mg, 0.04 mmol) and NEt_3 (56 μL, 0.4 mmol) in dry CH_2Cl_2 (2 mL) at -78 °C for 24 h afforded **4v** as a colorless oil (92 mg, 91%, *dr* = 90:10).

- R_f = 0.6 (Petroleum Ether: Ethyl Acetate = 8 : 1).
- ^{1}H NMR (400 MHz, $CDCl_3$) δ 7.62 - 7.55 (m, 6H), 7.45 (t, *J* = 8 Hz, 2H), 7.36 (t, *J* = 8 Hz, 1H), 6.75 (d, *J* = 4 Hz, 1H), 6.47 (d, *J* = 4 Hz, 1H), 5.12 (q, *J* = 12 Hz, 2H), 4.38 (q, *J* = 8 Hz, 1H), 1.47 (d, *J* = 8 Hz, 3H), 0.93 (s, 9H), 0.35 (s, 3H).

- ^{13}C NMR (100 MHz, $CDCl_3$) δ 173.4, 152.0, 142.2, 141.9 (q, *J* = 42 Hz), 140.9, 134.9, 134.1, 128.8, 127.5, 127.1, 126.3, 118.8 (q, *J* = 266 Hz), 112.3 (q, *J* = 3 Hz), 111.2, 68.3, 57.6, 25.6, 21.2, 18.3, -7.2.
- ^{29}Si NMR (80 MHz, $CDCl_3$) δ 10.11.
- IR (neat) cm $^{-1}$ 2939, 1757, 1386, 1318, 1264, 1108, 825.
- HRMS calcd for $C_{26}H_{29}F_3O_4Si$ $(M+Na)^+$ 513.1679 found 513.1681.
- $[\alpha]^{25}_D$ = -43.2 (*c* = 1.0, $CHCl_3$).

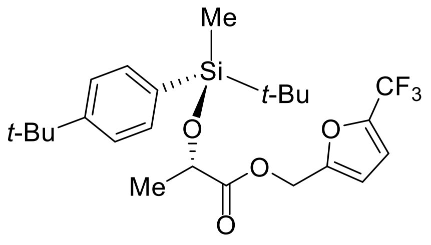

4w

(5-(trifluoromethyl)furan-2-yl)methyl(2S)-2-((tert-butyl(4-(tert-butyl)phenyl)(methyl)silyl)oxy)-propanoate (**4w**)

4w was prepared according to Method **6B**.

S1d (71 mg, 0.3 mmol), BPO (8 mg, 0.03 mmol), CCl_4 (3 mL), 100 °C refluxed for 21 h; then concentration followed by **2i** (48 mg, 0.2 mmol), **3e** (6 mg, 0.04 mmol) and NEt_3 (56 μL, 0.4 mmol) in dry CH_2Cl_2 (2 mL) at -78 °C for 24 h afforded **4w** as a colorless oil (92 mg, 98%, *dr* = 89:11).

- R_f = 0.7 (Petroleum Ether: Ethyl Acetate = 8 : 1).
- ^1H NMR (400 MHz, $CDCl_3$) δ 7.45 (d, *J* = 8 Hz, 3H), 7.37 (d, *J* = 8 Hz, 2H), 6.75 (d, *J* = 4 Hz, 1H), 6.47 (d, *J* = 4 Hz, 1H), 5.10 (q, *J* = 12 Hz, 2H), 4.33 (q, *J* = 8 Hz, 1H), 1.44 (d, *J* = 8 Hz, 3H), 1.32 (s, 9H), 0.89 (s, 9H), 0.29 (s, 3H).
- ^{13}C NMR (100 MHz, $CDCl_3$) δ 173.5, 152.5, 152.0, 142.3 (q, *J* = 42 Hz), 134.3, 131.7, 124.5, 118.8 (q, *J* = 266 Hz), 112.3 (q, *J* = 3 Hz), 111.2, 68.2, 57.6, 34.7, 31.2, 25.6, 21.2, 18.3, -7.2.
- ^{29}Si NMR (80 MHz, $CDCl_3$) δ 10.17.
- IR (neat) cm $^{-1}$ 2961, 2863, 1760, 1387, 1317, 1260, 1133, 784, 763.
- HRMS calcd for $C_{24}H_{33}F_3O_4Si$ $(M+Na)^+$ 493.1992 found 493.1989.
- $[\alpha]^{25}_D$ = -62 (*c* = 0.5, $CHCl_3$).

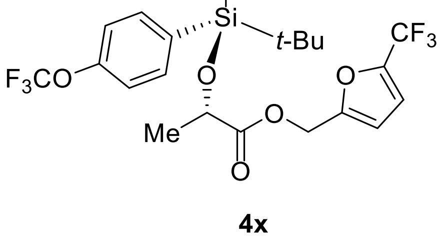

4x

(5-(trifluoromethyl)furan-2-yl)methyl(2S)-2-((tert-butyl(methyl)(4-(trifluoromethoxy)phenyl)silyl)oxy)-propanoate (**4x**)

4x was prepared according to Method **6B**.

S1e (79 mg, 0.3 mmol), BPO (8 mg, 0.03 mmol), CCl_4 (3 mL), 100 °C refluxed for 21 h; then concentration

followed by **2i** (48 mg, 0.2 mmol), **3e** (6 mg, 0.04 mmol) and NEt_3 (56 μL, 0.4 mmol) in dry CH_2Cl_2 (2 mL) at -78 °C for 24 h afforded **4x** as a colorless oil (77 mg, 77%, *dr* = 88:11).

- R_f = 0.5 (Petroleum Ether: Ethyl Acetate = 8 : 1).
- ^{1}H NMR (400 MHz, $CDCl_3$) δ 7.56 (d, J = 8 Hz, 2H), 7.20 (d, J = 8 Hz, 2H), 6.76 (d, J = 4 Hz, 1H), 6.47 (d, J = 4 Hz, 1H), 5.11 (q, J = 12 Hz, 2H), 4.33 (q, J = 8 Hz, 1H), 1.44 (d, J = 8 Hz, 3H), 0.88 (s, 9H), 0.32 (s, 3H).
- ^{13}C NMR (150 MHz, $CDCl_3$) δ 173.1, 151.9, 150.5, 142.4 (q, J = 44 Hz), 136.0, 134.3, 119.8, 120.4 (q, J = 255 Hz), 118.8 (q, J = 266 Hz), 112.3 (q, J = 3 Hz), 111.3, 68.4, 57.6, 25.5, 21.2, 18.2, -7.3.
- ^{29}Si NMR (80 MHz, $CDCl_3$) δ 9.53.
- ^{19}F NMR (376 MHz, $CDCl_3$) δ -57.60, -64.29.
- IR (neat) cm $^{-1}$ 3008, 2989, 2859, 1759, 1473, 1275, 1131, 802, 763.
- HRMS calcd for $C_{21}H_{24}F_6O_5Si$ $(M+Na)^+$ 521.1189 found 521.1185.
- $[\alpha]^{25}_D$ = -44 (c = 1.0, $CHCl_3$).

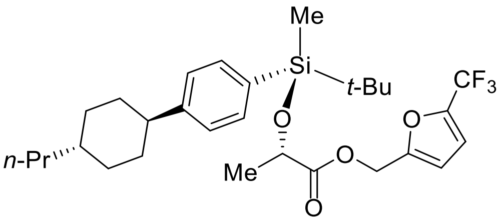

4y

(5-(trifluoromethyl)furan-2-yl)methyl-(2S)-2-((tert-butyl(methyl)(4-((1s,4S)-4-propylcyclohexyl)phenyl)silyl)oxy)propanoate (**4y**)

4y was prepared according to Method **6B**.

S1f (91 mg, 0.3 mmol), BPO (8 mg, 0.03 mmol), CCl_4 (3 mL), 100 °C refluxed for 21 h; then concentration followed by **2i** (48 mg, 0.2 mmol), **3e** (6 mg, 0.04 mmol) and NEt_3 (56 μL, 0.4 mmo) in dry CH_2Cl_2 (2 mL) at -78 °C for 24 h afforded **4y** as a colorless oil (84 mg, 78%, *dr* = 90:10).

- R_f = 0.7 (Petroleum Ether: Ethyl Acetate = 8 : 1).
- ^{1}H NMR (400 MHz, $CDCl_3$) δ 7.44 (d, J = 8 Hz, 2H), 7.20 (d, J = 8 Hz, 2H), 6.75 (d, J = 4 Hz, 1H), 6.47 (d, J = 4 Hz, 1H), 5.10 (q, J = 12 Hz, 2H), 4.34 (q, J = 8 Hz, 1H), 2.46 (ddd, J = 12, 8, 4 Hz, 1H), 1.89 (t, J = 12 Hz, 4H), 1.44 (d, J = 8 Hz, 3H), 1.39-1.29 (m, 6H), 1.11-1.00 (m, 2H), 0.91 (t, J = 8 Hz, 3H), 0.89 (s, 9H), 0.29 (s, 3H).
- ^{13}C NMR (150 MHz, $CDCl_3$) δ 173.5, 152.0, 149.3, 142.3 (q, J = 44 Hz), 134.5, 132.1, 126.2, 118.8 (q, J = 266 Hz), 112.3 (q, J = 3 Hz), 111.2, 68.2, 57.6, 44.6, 39.7, 37.0, 34.1, 33.6, 25.6, 21.2, 20.0, 18.2, 14.4, -7.3.
- ^{29}Si NMR (80 MHz, $CDCl_3$) δ 10.16.
- IR (neat) cm $^{-1}$ 2925, 2856, 1759, 1260, 1108, 764.

➢ HRMS calcd for $C_{29}H_{41}F_3O_4Si$ $(M+Na)^+$ 561.2618 found 561.2619.

➢ $[\alpha]^{25}_D$ = -50.4 (*c* = 1.0, $CHCl_3$).

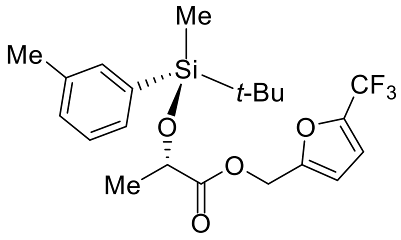

4z

(5-(trifluoromethyl)furan-2-yl)methyl(2S)-2-((tert-butyl(methyl)(m-tolyl)silyl)oxy)propanoate (**4z**)

4z was prepared according to Method **6B**.

S1g (58 mg, 0.3 mmol), BPO (8 mg, 0.03 mmol), CCl_4 (3 mL), 100 °C refluxed for 21 h; then concentration followed by **2i** (48 mg, 0.2 mmol,), **3e** (6 mg, 0.04 mmol) and NEt_3 (56 μL, 0.4 mmol) in dry CH_2Cl_2 (2 mL) at -78 °C for 24 h afforded **4z** as a colorless oil (80 mg, 93%, *dr* = 94:6).

➢ R_f = 0.6 (Petroleum Ether: Ethyl Acetate = 8 : 1).

➢ ^{1}H NMR (400 MHz, $CDCl_3$) δ 7.33 (d, *J* = 8 Hz, 2H), 7.27 - 7.19 (m, 2H), 6.75 (d, *J* = 4 Hz, 1H), 6.47 (d, *J* = 4 Hz, 1H), 5.10 (q, *J* = 12 Hz, 2H), 4.33 (q, *J* = 8 Hz, 1H), 2.35 (s, 3H), 1.44 (d, *J* = 8 Hz, 3H), 0.89 (s, 9H), 0.30 (s, 3H).

➢ ^{13}C NMR (150 MHz, $CDCl_3$) δ 173.4, 152.0, 142.3 (q, *J* = 44 Hz), 136.9, 135.3, 135.0, 131.5, 130.4, 127.5, 118.8 (q, *J* = 267 Hz), 112.3 (q, *J* = 3 Hz), 111.2, 68.3, 57.6, 34.7, 25.6, 21.6, 21.2, 18.2, -7.3.

➢ ^{29}Si NMR (80 MHz, $CDCl_3$) δ 10.07.

➢ IR (neat) cm $^{-1}$ 2931, 2859, 1759, 1741, 1317, 1107, 800, 770.

➢ HRMS calcd for $C_{21}H_{27}F_3O_4Si$ $(M+Na)^+$ 451.1523 found 451.1521.

➢ $[\alpha]^{25}_D$ = -59.2 (*c* = 1.0, $CHCl_3$).

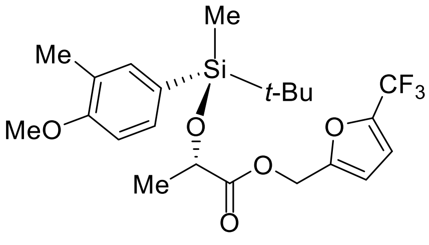

4aa

(5-(trifluoromethyl)furan-2-yl)methyl(2S)-2-((tert-butyl(4-methoxy-3-methylphenyl)(methyl)silyl)oxy)-propanoate (**4aa**)

4aa was prepared according to Method **6B**.

S1h (67 mg, 0.3 mmol), BPO (8 mg, 0.03 mmol), CCl_4 (3 mL), 100 °C refluxed for 21 h; then concentration followed by **2i** (48 mg, 0.2 mmol), **3e** (6 mg, 0.04 mmol) and NEt_3 (56 μL, 0.4 mmol) in dry CH_2Cl_2 (2 mL) at -78 °C for 24 h afforded **4aa** as a colorless oil (82 mg, 90%, *dr* = 94:6).

➢ R_f = 0.5 (Petroleum Ether: Ethyl Acetate = 5 : 1).

➢ ^{1}H NMR (400 MHz, $CDCl_3$) δ 7.34 (d, *J* = 8.0 Hz, 1H), 7.26 (s, 1H), 6.83 (d, *J* = 8 Hz, 1H), 6.75 (d, *J* = 4 Hz, 1H), 6.46 (d, *J* = 4 Hz, 1H), 5.10 (q, *J* = 12 Hz, 2H), 4.32 (q, *J* = 8 Hz, 1H),

3.84 (s, 3H), 2.22 (s, 3H), 1.43 (d, *J* = 8 Hz, 3H), 0.89 (s, 9H), 0.28 (s, 3H).

- ^{13}C NMR (150 MHz, $CDCl_3$) δ 173.5, 159.1, 152.0, 142.3 (q, *J* = 42 Hz), 136.7, 133.7, 125.8, 125.7, 118.8 (q, *J* = 266 Hz), 112.3 (q, *J* = 3 Hz), 111.2, 109.2, 68.2, 57.6, 55.0, 25.7, 21.2, 18.3, 16.3, -7.2.
- ^{29}Si NMR (80 MHz, $CDCl_3$) δ 10.23.
- IR (neat) cm $^{-1}$ 3008, 2989, 2859, 1741, 1463, 1275, 1105, 764.
- HRMS calcd for $C_{22}H_{29}F_3O_5Si$ $(M+Na)^+$ 481.1629 found 481.1633.
- $[\alpha]^{25}_D$ = -56.4 (*c* = 0.5, $CHCl_3$).

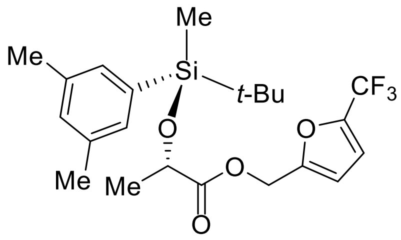

4ab

(5-(trifluoromethyl)furan-2-yl)methyl(2S)-2-((tert-butyl(3,5-dimethylphenyl)(methyl)silyl)oxy)-propanoate (**4ab**)

4ab was prepared according to Method **6B**.

S1i (62 mg, 0.3 mmol), BPO (8 mg, 0.03 mmol), CCl_4 (3 mL), 100 °C refluxed for 21 h; then concentration followed by **2i** (48 mg, 0.2 mmol), **3e** (6 mg, 0.04 mmol) and NEt_3 (56 μL, 0.4 mmol) in dry CH_2Cl_2 (2 mL) at -78 °C for 24 h afforded **4ab** as a colorless oil (80 mg, 91%, *dr* = 95:5).

- R_f = 0.7 (Petroleum Ether: Ethyl Acetate = 8 : 1).
- ^{1}H NMR (400 MHz, $CDCl_3$) δ 7.13 (s, 2H), 7.03 (s, 1H), 6.75 (d, *J* = 4 Hz, 1H), 6.47 (d, *J* = 4 Hz, 1H), 5.10 (dd, *J* = 20, 12 Hz, 2H), 4.33 (q, *J* = 4 Hz, 1H), 2.32 (s, 6H), 1.44 (d, *J* = 8 Hz, 3H), 0.89 (s, 9H), 0.29 (s, 3H).
- ^{13}C NMR (150 MHz, $CDCl_3$) δ 173.4, 152.0, 142.3 (q, *J* = 42 Hz), 136.8, 135.2, 132.2, 131.3, 118.8 (q, *J* = 266 Hz), 112.3 (q, *J* = 3 Hz), 111.2, 68.3, 57.6, 25.7, 21.4, 21.2, 18.2, -7.2.
- ^{29}Si NMR (80 MHz, $CDCl_3$) δ 10.02.
- IR (neat) cm $^{-1}$ 3006, 2989, 1759, 1742, 1275, 1107, 764.
- HRMS calcd for $C_{22}H_{29}F_3O_4Si$ $(M+Na)^+$ 465.1679 found 465.1680.
- $[\alpha]^{25}_D$ = -65.6 (*c* = 0.125, $CHCl_3$).

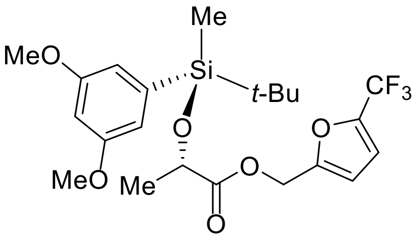

4ac

(5-(trifluoromethyl)furan-2-yl)methyl(2S)-2-((tert-butyl(3,5-dimeth oxyphenyl)(methyl)silyl)oxy)propanoate (**4ac**)

4ac was prepared according to Method **6B**.

S1j (72 mg, 0.3 mmol), BPO (8 mg, 0.03 mmol), CCl_4 (3 mL), 100 °C refluxed for 21 h; then concentration followed

by **2i** (48 mg, 0.2 mmol,), **3e** (6 mg, 0.04 mmol) and NEt_3 (56 μL, 0.4 mmol) in dry CH_2Cl_2 (2 mL) at -78 °C for 24 h afforded **4ac** as a colorless oil (65 mg, 69%, *dr* = 92:8).

- R_f = 0.4 (Petroleum Ether: Ethyl Acetate = 5 : 1).
- ^{1}H NMR (400 MHz, $CDCl_3$) δ 6.75 (d, *J* = 4 Hz, 1H), 6.65 (d, *J* = 4 Hz, 2H), 6.49 (t, *J* = 4 Hz, 1H), 6.47 (d, *J* = 4 Hz, 1H), 5.10 (q, *J* = 12 Hz, 2H), 4.34 (q, *J* = 8 Hz, 1H), 3.80 (s, 6H), 1.45 (d, *J* = 8Hz, 3H), 0.89 (s, 9H), 0.29 (s, 3H).
- ^{13}C NMR (150 MHz, $CDCl_3$) δ 173.5, 159.1, 152.0, 142.3 (q, *J* = 42 Hz), 136.7, 133.7, 125.8, 125.7, 118.8 (q, *J* = 266 Hz), 112.3 (q, *J* = 3 Hz), 111.2, 109.2, 68.2, 57.6, 55.0, 25.7, 21.2, 18.3, 16.3, -7.2.
- ^{29}Si NMR (80 MHz, $CDCl_3$) δ 10.27.
- IR (neat) cm $^{-1}$ 2933, 1583, 1264, 1183, 1061, 896, 763.
- HRMS calcd for $C_{22}H_{29}F_3O_6Si$ $(M+Na)^+$ 497.1578 found 497.1580.
- $[\alpha]^{25}_D$ = -35.2 (*c* = 0.5, $CHCl_3$).

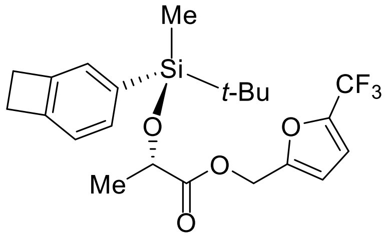

4ad

<u>(5-(trifluoromethyl)furan-2-yl)methyl(2S)-2-((bicyclo[4.2.0]octa-1(6),2,4-trien-3-yl(tert-butyl)(methyl)silyl)oxy)propanoate</u> (**4ad**)

4ad was prepared according to Method **6B**.

S1k (62 mg, 0.3 mmol), BPO (8 mg, 0.03 mmol), CCl_4 (3 mL), 100 °C refluxed for 21 h; then concentration followed by **2i** (48 mg, 0.2 mmol), **3e** (6 mg, 0.04 mmol) and NEt_3 (56 μL, 0.4 mmol) in dry CH_2Cl_2 (2 mL) at -78 °C for 24 h afforded **4ad** as a colorless oil (84 mg, 95%, *dr* = 90:10).

- R_f = 0.7 (Petroleum Ether: Ethyl Acetate = 6 : 1).
- ^{1}H NMR (400 MHz, $CDCl_3$) δ 7.38 (d, *J* = 8 Hz, 1H), 7.22 (s, 1H), 7.07 (d, *J* = 8 Hz, 1H), 6.76 (d, *J* = 4 Hz, 1H), 6.47 (d, *J* = 4 Hz, 1H), 5.11 (q, *J* = 12 Hz, 2H), 4.33 (q, *J* = 8 Hz, 1H), 3.20 (s, 4H), 1.44 (d, *J* = 8 Hz, 3H), 0.89 (s, 9H), 0.29 (s, 3H).
- ^{13}C NMR (150 MHz, $CDCl_3$) δ 173.5, 152.0, 147.8, 145.4, 142.3 (q, *J* = 42 Hz), 133.3, 132.8, 128.2, 121.7, 118.8 (q, *J* = 260 Hz), 112.3 (q, *J* = 3 Hz), 111.2, 68.2, 57.6, 29.9, 29.8, 25.7, 21.2, 18.2, -7.2.
- ^{29}Si NMR (80 MHz, $CDCl_3$) δ 10.55.
- IR (neat) cm $^{-1}$ 2931, 2863, 1759, 1741, 1317, 1260, 1275, 1131, 1050, 802, 764.
- HRMS calcd for $C_{22}H_{27}F_3O_4Si$ $(M+Na)^+$ 463.1523 found 463.1524.
- $[\alpha]^{25}_D$ = -36 (*c* = 0.25, $CHCl_3$).

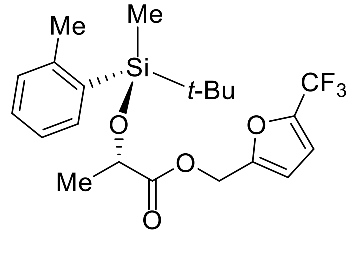

4ae

(5-(trifluoromethyl)furan-2-yl)methyl(2S)-2-((tert-butyl(methyl)(o-tolyl)silyl)oxy)propanoate (**4ae**)

4ae was prepared according to Method **6B**.

S1l (58 mg, 0.3 mmol), BPO (8 mg, 0.03 mmol), CCl_4 (3 mL), 100 °C refluxed for 21 h; then concentration followed by **2i** (48 mg, 0.2 mmol), **3e** (6 mg, 0.04 mmol) and NEt_3 (56 μL, 0.4 mmol) in dry CH_2Cl_2 (2 mL) at -78 °C for 24 h afforded **4ae** as a colorless oil (77 mg, 90%, *dr* = 94:6).

- R_f = 0.7 (Petroleum Ether: Ethyl Acetate = 8 : 1).
- ^{1}H NMR (400 MHz, $CDCl_3$) δ 7.52 (d, *J* = 8 Hz, 1H), 7.31 (d, *J* = 8 Hz, 1H), 7.19 - 7.18 (m, 3H), 6.78 (d, *J* = 4Hz, 1H), 6.51 (d, *J* = 4 Hz, 1H), 5.16 (q, *J* = 12 Hz, 2H), 4.42 (q, *J* = 8 Hz, 1H), 2.49 (s, 3H), 1.47 (d, *J* = 8 Hz, 3H), 0.91 (s, 9H), 0.41 (s, 3H).
- ^{13}C NMR (100 MHz, $CDCl_3$) δ 173.4, 151.9, 144.0, 142.4 (q, *J* = 42 Hz), 135.7, 134.2, 130.3, 129.5, 124.4, 118.8 (q, *J* = 266 Hz), 112.3 (q, *J* = 3 Hz), 111.3, 68.5, 57.6, 34.7, 26.0, 23.7, 21.2, 19.3, -5.1.
- ^{29}Si NMR (80 MHz, $CDCl_3$) δ 11.37.
- IR (neat) cm $^{-1}$ 2933, 2859, 1759, 1317, 1107, 783, 764.
- HRMS calcd for $C_{21}H_{27}F_3O_4Si$ $(M+Na)^+$ 451.1523 found 451.1525.
- $[\alpha]^{25}_D$ = -36.8 (*c* = 0.25, $CHCl_3$).

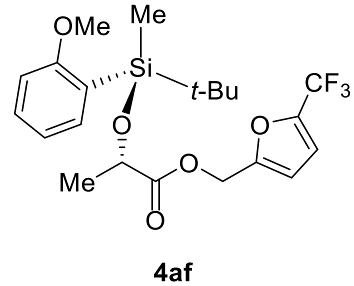

4af

(5-(trifluoromethyl)furan-2-yl)methyl(2S)-2-((tert-butyl(2-methoxyphenyl)(methyl)silyl)oxy)propanoate (**4af**)

4af was prepared according to Method **6B**.

S1m (63 mg, 0.3 mmol), BPO (8 mg, 0.03 mmol), CCl_4 (3 mL), 100 °C refluxed for 21 h; then concentration followed by **2i** (48 mg, 0.2 mmol), **3e** (6 mg, 0.04 mmol) and NEt_3 (56 μL, 0.4 mmol) in dry CH_2Cl_2 (2 mL) at -78 °C for 24 h afforded **4af** as a colorless oil (74 mg, 83%, *dr* = 94:6).

- R_f = 0.5 (Petroleum Ether: Ethyl Acetate = 5 : 1).
- ^{1}H NMR (400 MHz, $CDCl_3$) δ 7.49 (dd, *J* = 8, 2 Hz, 1H), 7.36 (ddd, *J* = 8, 8, 2 Hz, 1H), 6.96 (td, *J* = 8, 2 Hz, 1H), 6.82 (d, *J* = 8 Hz, 1H), 6.75 (dd, *J* = 4, 1 Hz, 1H), 6.46 (d, *J* = 4 Hz, 1H), 5.10 (q, *J* = 12 Hz, 2H), 4.40 (q, *J* = 8Hz, 1H), 3.74 (s, 3H), 1.45 (d, *J* = 8 Hz, 3H), 0.88 (s, 9H), 0.32 (s, 3H).
- ^{13}C NMR (100 MHz, $CDCl_3$) δ 173.7, 164.0, 152.0, 142.3 (q, *J* = 42 Hz), 136.5, 131.3, 123.4, 120.3, 118.8 (q, *J* = 266 Hz), 112.3 (q, *J* = 3 Hz), 111.2, 109.5, 76.8, 68.4, 57.5, 54.5, 26.0,

21.3, 18.8, -5.7.

- ^{29}Si NMR (80 MHz, $CDCl_3$) δ 11.08.
- IR (neat) cm $^{-1}$ 2859, 1587, 1427, 1275, 1107, 853, 764.
- HRMS calcd for $C_{21}H_{27}F_3O_5Si$ $(M+Na)^+$ 467.1472 found 467.1477.
- $[\alpha]^{25}_D$ = -39.4 (*c* = 1.0, $CHCl_3$).

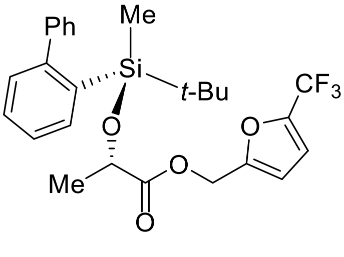

4ag

(5-(trifluoromethyl)furan-2-yl)methyl(2S)-2-(([1,1'-biphenyl]-2-yl(tert-butyl)(methyl)silyl)oxy)propanoate (**4ag**)

4ag was prepared according to Method **6B**.

S1n (77 mg, 0.3 mmol), BPO (8 mg, 0.03 mmol), CCl_4 (3 mL), 100 °C refluxed for 21 h; then concentration followed by **2i** (48 mg, 0.2 mmol), **3e** (6 mg, 0.04 mmol) and NEt_3 (56 μL, 0.4 mmol) in dry CH_2Cl_2 (2 mL) at -78 °C for 24 h afforded **4ag** as a colorless oil (89 mg, 91%, *dr* = 72:28).

- R_f = 0.6 (Petroleum Ether: Ethyl Acetate = 6 : 1).
- ^{1}H NMR (400 MHz, $CDCl_3$) δ 7.78 - 7.75 (m, 1H), 7.40 - 7.28 (m, 8H), 6.71 (d, *J* = 4 Hz, 1H), 6.42 (d, *J* = 4 Hz, 1H), 5.05 (q, *J* = 12 Hz, 2H), 4.18 (q, *J* = 8 Hz, 1H), 1.33 (d, *J* = 8 Hz, 3H), 0.86 (s, 9H), 0.08 (s, 3H).
- ^{13}C NMR (100 MHz, $CDCl_3$) δ 173.5, 152.0, 149.4, 144.2, 142.3 (q, *J* = 42 Hz), 136.0, 129.9, 129.5, 129.2, 128.8, 127.3, 127.0, 125.7, 118.8 (q, *J* = 266 Hz), 112.2 (q, *J* = 3 Hz), 111.1, 68.1, 57.5, 26.4, 21.1, 19.0, -5.4.
- ^{29}Si NMR (80 MHz, $CDCl_3$) δ 10.26.
- IR (neat) cm $^{-1}$ 2966, 2929, 2859, 1758, 1474, 1317, 1130, 788.
- HRMS calcd for $C_{26}H_{29}F_3O_4Si$ $(M+Na)^+$ 513.1679 found 513.1680.
- $[\alpha]^{25}_D$ = -38.8 (*c* = 0.5, $CHCl_3$).

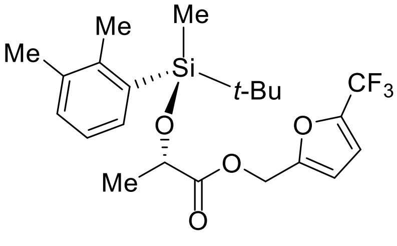

4ah

(5-(trifluoromethyl)furan-2-yl)methyl(2S)-2-((tert-butyl-(2,3-dimethylphenyl)(methyl)silyl)oxy)propanoate (**4ah**)

4ah was prepared according to Method **6B**.

S1o (62 mg, 0.3 mmol), BPO (8 mg, 0.03 mmol), CCl_4 (3 mL), 100 °C refluxed for 21 h; then concentration followed by **2i** (48 mg, 0.2 mmol), **3e** (6 mg, 0.04 mmol) and NEt_3 (56 μL, 0.4 mmol) in dry CH_2Cl_2 (2 mL) at -78 °C for 24 h afforded **4ah** as a colorless oil (82 mg, 93%, *dr* = 88:12).

- R_f = 0.7 (Petroleum Ether: Ethyl Acetate = 8 : 1).

- ^{1}H NMR (400 MHz, $CDCl_3$) δ 7.34 (d, *J* = 8 Hz, 1H), 7.19 (d, *J* = 8 Hz, 1H), 7.11 (t, *J* = 8 Hz, 1H), 6.77 (d, *J* = 4 Hz, 1H), 6.49 (d, *J* = 4 Hz, 1H), 5.14 (q, *J* = 12 Hz, 2H), 4.40 (q, *J* = 8 Hz, 1H), 2.40 (s, 3H), 2.27 (s, 3H), 1.45 (d, *J* = 8 Hz, 3H), 0.90 (s, 9H), 0.39 (s, 3H).
- ^{13}C NMR (150 MHz, $CDCl_3$) δ 173.5, 152.0, 142.7, 142.3 (q, *J* = 42 Hz), 136.9, 134.3, 133.7, 131.3, 124.7, 118.8 (q, *J* = 266 Hz), 112.3, (q, *J* = 3 Hz), 111.3, 68.5, 57.6, 26.2, 21.1, 20.8, 20.6, 19.3, -5.0.
- ^{29}Si NMR (80 MHz, $CDCl_3$) δ 11.97.
- IR (neat) cm $^{-1}$ 3005, 2989, 1462, 1275, 1261, 766.
- HRMS calcd for $C_{22}H_{29}F_3O_4Si$ $(M+Na)^+$ 465.1679 found 465.1680.
- $[\alpha]^{25}_D$ = -25.6 (*c* = 1.0, $CHCl_3$).

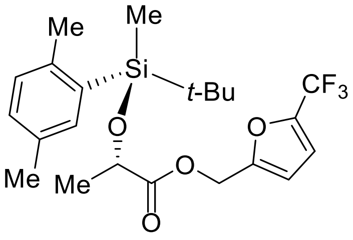

4ai

(5-(trifluoromethyl)furan-2-yl)methyl(2S)-2-((tert-butyl(2,5-dimethylphenyl)(methyl)silyl)oxy)propanoate (**4ai**)

4ai was prepared according to Method **6B**.

S1p (62 mg, 0.3 mmol), BPO (8 mg, 0.03 mmol), CCl_4 (3 mL), 100 °C refluxed for 21 h; then concentration followed by **2i** (48 mg, 0.2 mmol), **3e** (6 mg, 0.04 mmol) and NEt_3 (56 μL, 0.4 mmol) in dry CH_2Cl_2 (2 mL) at -78 °C for 24 h afforded **4ai** as a colorless oil (83 mg, 94%, *dr* = 88:12).

- R_f = 0.7 (Petroleum Ether: Ethyl Acetate = 8 : 1).
- ^{1}H NMR (400 MHz, $CDCl_3$) δ 7.29 (s, 1H), 7.11 - 7.04 (m, 2H), 6.76 (d, *J* = 4 Hz, 1H), 6.49 (d, *J* = 4 Hz, 1H), 5.14 (q, *J* = 12 Hz, 2H), 4.39 (q, *J* = 8 Hz, 1H), 2.42 (s, 3H), 2.31 (s, 3H), 1.45 (d, *J* = 8 Hz, 3H), 0.90 (s, 9H), 0.38 (s, 3H).
- ^{13}C NMR (100 MHz, $CDCl_3$) δ 173.5, 152.0, 142.4 (q, *J* = 42 Hz), 140.8, 136.4, 133.9, 133.5, 130.3, 130.2, 118.8 (q, *J* = 266 Hz), 112.3 (q, *J* = 3 Hz), 111.3, 68.5, 57.6, 26.0, 23.1, 21.2, 21.1, 19.3, -5.1.
- ^{29}Si NMR (80 MHz, $CDCl_3$) δ 11.48.
- IR (neat) cm $^{-1}$ 2933, 2863, 1760, 1742, 1317, 1259, 1107, 764.
- HRMS calcd for $C_{22}H_{29}F_3O_4Si$ $(M+Na)^+$ 465.1679 found 465.1682.
- $[\alpha]^{25}_D$ = -32.4 (*c* = 1.0, $CHCl_3$).

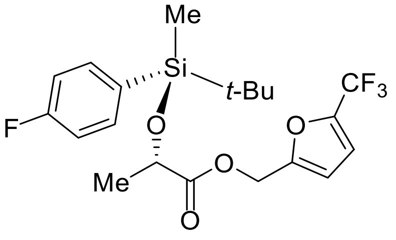

4aj

(5-(trifluoromethyl)furan-2-yl)methyl(2S)-2-((tert-butyl(4-fluorophenyl)(methyl)silyl)oxy)propanoate (**4aj**)

4aj was prepared according to Method **6B**.

S1q (59 mg, 0.3 mmol), BPO (8 mg, 0.03 mmol), CCl_4 (3 mL), 100 °C refluxed for 21 h; then concentration followed by **2i** (48 mg, 0.2 mmol), **3e** (6 mg, 0.04 mmol) and NEt_3 (56 μL, 0.4 mmol) in dry CH_2Cl_2 (2 mL) at -78 °C for 24 h afforded **4aj** as a colorless oil (80 mg, 93%, *dr* = 93:7).

- R_f = 0.7 (Petroleum Ether: Ethyl Acetate = 8 : 1).
- ^{1}H NMR (400 MHz, $CDCl_3$) δ 7.53 - 7.49 (m, 2H), 7.06 (t, *J* = 8 Hz, 2H), 6.76 (d, *J* = 4 Hz, 1H), 6.47 (d, *J* = 4 Hz, 1H), 5.11 (q, *J* = 12 Hz, 2H), 4.32 (q, *J* = 8 Hz, 1H), 1.43 (d, *J* = 8 Hz, 3H), 0.88 (s, 9H), 0.31 (s, 3H).
- ^{13}C NMR (100 MHz, $CDCl_3$) δ 173.2, 164.0 (d, *J* = 247 Hz), 151.9, 142.4 (q, *J* = 43 Hz), 136.3 (d, *J* = 7 Hz), 131.0 (d, *J* = 4 Hz), 118.8 (q, *J* = 265 Hz), 114.8 (d, *J* = 19 Hz), 112.3 (q, *J* = 3 Hz), 111.3, 68.3, 57.6, 25.1, 21.2, 18.2, -7.3.
- ^{29}Si NMR (80 MHz, $CDCl_3$) δ 9.27.
- ^{19}F NMR (376 MHz, $CDCl_3$) δ -64.25, -111.08 (ddd, *J* = 15.4, 9.3, 6.1 Hz).
- IR (neat) cm $^{-1}$ 2859, 1759, 1589, 1275, 1260, 1104, 934, 764.
- HRMS calcd for $C_{20}H_{24}F_4O_4Si$ $(M+Na)^+$ 455.1272 found 455.1275.
- $[\alpha]^{25}_D$ = -27.2 (*c* = 1.0, $CHCl_3$).

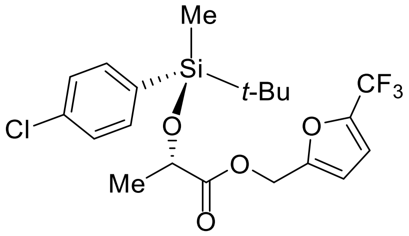

4ak

(5-(trifluoromethyl)furan-2-yl)methyl(2S)-2-((tert-butyl(4-chlorophenyl)(methyl)silyl)oxy)propanoate (**4ak**)

4ak was prepared according to Method **6B**.

S1r (64 mg, 0.3 mmol), BPO (8 mg, 0.03 mmol), CCl_4 (3 mL), 100 °C refluxed for 21 h; then concentration followed by **2i** (48 mg, 0.2 mmol), **3e** (6 mg, 0.04 mmol) and NEt_3 (56 μL, 0.4 mmol) in dry CH_2Cl_2 (2 mL) at -78 °C for 24 h afforded **4ak** as a colorless oil (83 mg, 92%, *dr* = 89:11).

- R_f = 0.7 (Petroleum Ether: Ethyl Acetate = 8 : 1).
- ^{1}H NMR (400 MHz, $CDCl_3$) δ 7.46 (d, *J* = 8 Hz, 2H), 7.34 (d, *J* = 8 Hz, 2H), 6.76 (d, *J* = 4 Hz, 1H), 6.47 (d, *J* = 4 Hz, 1H), 5.11 (q, *J* = 12 Hz, 2H), 4.32 (q, *J* = 8 Hz, 1H), 1.43 (d, *J* = 8 Hz, 3H), 0.88 (s, 9H), 0.31 (s, 3H).
- ^{13}C NMR (100 MHz, $CDCl_3$) δ 173.2, 151.9, 142.4 (q, *J* = 42 Hz), 136.0, 135.7, 133.8, 127.9,

118.8 (q, J = 266 Hz), 112.3 (q, J = 3 Hz), 111.3, 68.3, 57.6, 25.5, 21.2, 18.1, -7.3.

- ^{29}Si NMR (80 MHz, $CDCl_3$) δ 9.71.
- IR (neat) cm $^{-1}$ 2989, 2933, 1742, 1589, 1260, 1104, 782, 764.
- HRMS calcd for $C_{20}H_{24}ClF_3O_4Si$ $(M+Na)^+$ 471.0977 found 471.0980.
- $[\alpha]^{25}_D$ = -47.6 (c = 0.5, $CHCl_3$).

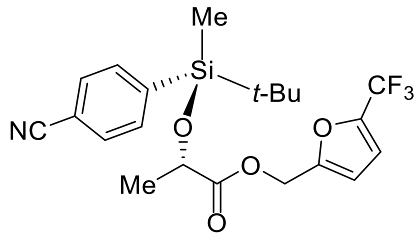

4al

(5-(trifluoromethyl)furan-2-yl)methyl(2S)-2-((tert-butyl(4-cyanophenyl)(methyl)silyl)oxy)propanoate (**4al**)

4al was prepared according to Method **6B**.

S1s (61 mg, 0.3 mmol), BPO (8 mg, 0.03 mmol), CCl_4 (3 mL), 100 °C refluxed for 21 h; then concentration followed by **2i** (48 mg, 0.2 mmol), **3e** (6 mg, 0.04 mmol) and NEt_3 (56 μL, 0.4 mmol) in dry CH_2Cl_2 (2 mL) at -78 °C for 24 h afforded **4al** as a colorless oil (71 mg, 81%, dr = 84:16).

- R_f = 0.4 (Petroleum Ether: Ethyl Acetate = 5 : 1).
- ^{1}H NMR (400 MHz, $CDCl_3$) δ 7.63 (s, 4H), 6.76 (d, J = 4 Hz, 1H), 6.47 (d, J = 4Hz, 1H), 5.12 (q, J = 12 Hz, 2H)), 4.34 (q, J = 8 Hz, 1H), 1.43 (d, J = 8 Hz, 3H), 0.87 (s, 9H), 0.34 (s, 3H).
- ^{13}C NMR (150 MHz, $CDCl_3$) δ 172.9, 151.7, 142.6, 142.4 (q, J = 42 Hz), 134.7, 130.8, 118.8, 118.8 (q, J = 270 Hz), 113.1, 112.3 (q, J = 3 Hz), 111.4, 68.5, 57.6, 25.4, 21.2, 18.2, -7.4.
- ^{29}Si NMR (80 MHz, $CDCl_3$) δ 9.88.
- IR (neat) cm $^{-1}$ 3060, 2853, 2926, 2108, 1446, 1394, 906, 823.
- HRMS calcd for $C_{21}H_{24}F_3NO_4Si$ $(M+Na)^+$ 462.1319 found 462.1321.
- $[\alpha]^{25}_D$ = -30.2 (c = 0.25, $CHCl_3$).

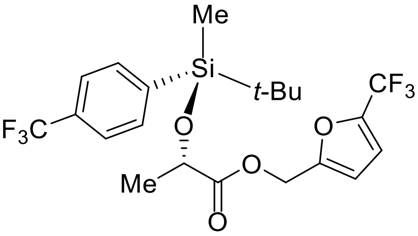

4am

(5-(trifluoromethyl)furan-2-yl)methyl(2S)-2-((tert-butyl(methyl)(4-(trifluoromethyl)phenyl)silyl)oxy)-propanoate (**4am**)

4am was prepared according to Method **6B**.

S1t (74 mg, 0.3 mmol), BPO (8 mg, 0.03 mmol), CCl_4 (3 mL), 100 °C refluxed for 21 h; then concentration followed by **2i** (48 mg, 0.2 mmol), **3e** (6 mg, 0.04 mmol) and NEt_3 (56 μL, 0.4 mmol) in dry CH_2Cl_2 (2 mL) at -78 °C for 24 h afforded **4am** as a colorless oil (92 mg, 96%, dr = 82:18).

- R_f = 0.6 (Petroleum Ether: Ethyl Acetate = 8 : 1).
- ^{1}H NMR (400 MHz, $CDCl_3$) δ 7.63 (dd, J = 20, 8 Hz, 4H), 6.76 (d, J = 4 Hz, 1H), 6.47 (d, J =

4 Hz, 1H), 5.16-5.07 (m, 2H), 4.34 (q, *J* = 8 Hz, 1H), 1.44 (d, *J* = 8 Hz, 3H), 0.89 (s, 9H), 0.35 (s, 3H).

- ^{13}C NMR (100 MHz, $CDCl_3$) δ 173.1, 151.8, 142.4 (q, *J* = 43 Hz), 134.6, 131.5 (q, *J* = 32 Hz), 124.2 (q, *J* = 272 Hz), 124.1 (q, *J* = 3 Hz), 118.8 (q, *J* = 266 Hz), 118.8 (q, *J* = 266 Hz), 112.3 (q, *J* = 3 Hz), 111.3, 68.5, 57.8, 25.4, 21.2, 18.2, -7.4.
- ^{29}Si NMR (80 MHz, $CDCl_3$) δ 9.28.
- ^{19}F NMR (376 MHz, $CDCl_3$) δ -63.0, -64.29.
- IR (neat) cm $^{-1}$ 3360, 2932, 2859, 1759, 1323, 1260, 1124, 764.
- HRMS calcd for $C_{21}H_{24}F_6O_4Si$ $(M+Na)^+$ 505.1240 found 505.1243.
- $[\alpha]^{25}_D$ = -50.4 (*c* = 1.0, $CHCl_3$).

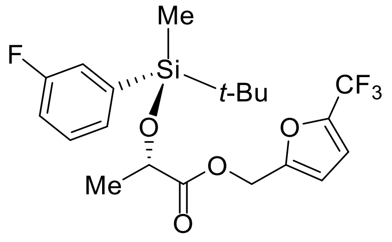

4an

<u>(5-(trifluoromethyl)furan-2-yl)methyl(2S)-2-((tert-butyl(3-fluorophenyl)(methyl)silyl)oxy)propanoate</u> (**4an**)

4an was prepared according to Method **6B**.

S1u (59 mg, 0.3 mmol), BPO (8 mg, 0.03 mmol), CCl_4 (3 mL), 100 °C refluxed for 21 h; then concentration followed by **2i** (48 mg, 0.2 mmol), **3e** (6 mg, 0.04 mmol) and NEt_3 (56 μL, 0.4 mmol) in dry CH_2Cl_2 (2 mL) at -78 °C for 24 h afforded **4an** as a colorless oil (82 mg, 96%, *dr* = 93:7).

- R_f = 0.7 (Petroleum Ether: Ethyl Acetate = 8 : 1).
- ^{1}H NMR (400 MHz, $CDCl_3$) δ 7.37 - 7.27 (m, 2H), 7.21 (dd, *J* = 8, 4 Hz, 1H), 7.10 - 7.04 (m, 1H), 6.76 (d, *J* = 4 Hz, 1H), 6.48 (d, *J* = 4 Hz, 1H), 5.11 (q, *J* = 12 Hz, 2H), 4.34 (q, *J* = 8 Hz, 1H), 1.44 (d, *J* = 8 Hz, 3H), 0.89 (s, 9H), 0.32 (s, 3H).
- ^{13}C NMR (150 MHz, $CDCl_3$) δ 173.1, 162.5 (d, *J* = 246 Hz), 151.9, 142.4 (q, *J* = 44 Hz), 138.7 (d, *J* = 5 Hz), 129.9 (d, *J* = 3 Hz), 129,4 (d, *J* = 6 Hz), 120.7 (d, *J* = 18 Hz), 118.8 (q, *J* = 266 Hz), 116.5 (d, *J* = 21 Hz), 112.3 (q, *J* = 3 Hz), 111.3, 68.4, 57.6, 25.5, 21.2, 18.2, -7.3.
- ^{29}Si NMR (80 MHz, $CDCl_3$) δ 9.38.
- ^{19}F NMR (376 MHz, $CDCl_3$) δ -64.26, -113.61 (td, *J* = 9.0, 5.1 Hz).
- IR (neat) cm $^{-1}$ 3005, 2989, 2859, 1759, 1474, 1275, 1260, 1130, 757.
- HRMS calcd for $C_{20}H_{24}F_4O_4Si$ $(M+Na)^+$ 455.1272 found 455.1267.
- $[\alpha]^{25}_D$ = -54.4 (*c* = 0.5, $CHCl_3$).

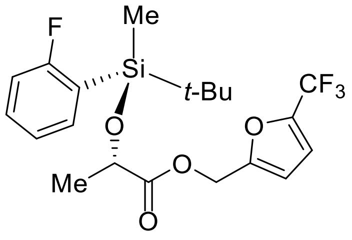

4ao

(5-(trifluoromethyl)furan-2-yl)methyl(2S)-2-((tert-butyl(2-fluorophenyl)(methyl)silyl)oxy)propanoate (**4ao**)

4ao was prepared according to Method **6B**.

S1v (59 mg, 0.3 mmol), BPO (8 mg, 0.03 mmol), CCl_4 (3 mL), 100 °C refluxed for 21 h; then concentration followed by **2i** (48 mg, 0.2 mmol), **3e** (6 mg, 0.04 mmol) and NEt_3 (56 μL, 0.4 mmol) in dry CH_2Cl_2 (2 mL) at -78 °C for 24 h afforded **4ao** as a colorless oil (77 mg, 90%, *dr* = 91:9).

- R_f = 0.7 (Petroleum Ether: Ethyl Acetate = 8 : 1).
- ^{1}H NMR (400 MHz, $CDCl_3$) δ 7.52 (dd, *J* = 8, 4 Hz, 1H), 7.46 - 7.45 (m, 1H), 7.34 (d, *J* = 8 Hz, 1H), 7.06 (dd, *J* = 12, 4 Hz, 1H), 6.76 (s, 1H), 6.47 (s, 1H), 5.11 (q, *J* = 13 Hz, 2H), 4.32 (q, *J* = 8 Hz, 1H), 1.43 (d, *J* = 8 Hz, 3H), 0.88 (s, 9H), 0.31 (s, 3H).
- ^{13}C NMR (150 MHz, $CDCl_3$) δ 173.3, 166.8 (d, *J* = 274 Hz), 151.9, 142.3 (q, *J* = 44 Hz), 136.4 (d, *J* = 10.5 Hz), 131.9 (d, *J* = 9 Hz), 123.7 (d, *J* = 1.5 Hz), 121.6 (d, *J* = 30 Hz), 118.8 (q, *J* = 266 Hz), 115.0 (d, *J* = 25.5 Hz), 112.3 (q, *J* = 3 Hz), 111.3, 68.5, 57.6, 25.5, 21.1, 18.5 (d, *J* = 10 Hz), -6.05 (d, *J* = 10 Hz).
- 29Si NMR (80 MHz, $CDCl_3$) δ 9.91.
- ^{19}F NMR (376 MHz, $CDCl_3$) δ -64.24, -97.79 (q, *J* = 6.8 Hz).
- IR (neat) cm $^{-1}$ 2859, 1759, 1471, 1275, 1260, 1131, 1107, 763.
- HRMS calcd for $C_{20}H_{24}F_4O_4Si$ $(M+Na)^+$ 455.1272 found 455.1275.
- $[\alpha]^{25}_D$ = -38.4 (*c* = 0.25, $CHCl_3$).

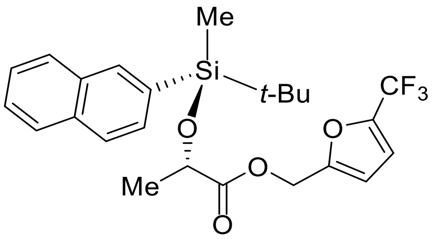

4ap

(5-(trifluoromethyl)furan-2-yl)methyl(2S)-2-((tert-butyl(methyl)(naphthalen-2-yl)silyl)oxy)propanoate (**4ap**)

4ap was prepared according to Method **6B**.

S1w (69 mg, 0.3 mmol), BPO (8 mg, 0.03 mmol), CCl_4 (3 mL), 100 °C refluxed for 21 h; then concentration followed by **2i** (48 mg, 0.2 mmol), **3e** (6 mg, 0.04 mmol) and NEt_3 (56 μL, 0.4 mmol) in dry CH_2Cl_2 (2 mL) at -78 °C for 24 h afforded **4ap** as a colorless oil (88 mg, 87%, *dr* = 94:6).

- R_f = 0.5 (Petroleum Ether: Ethyl Acetate = 10 : 1).
- ^{1}H NMR (400 MHz, $CDCl_3$) δ 8.03 (s, 1H), 7.84 (dd, *J* = 12, 8 Hz, 3H), 7.61 (d, *J* = 8 Hz, 1H), 7.50 (p, *J* = 8 Hz, 2H), 6.74 (d, *J* = 4 Hz, 1H), 6.45 (d, *J* = 4 Hz, 1H), 5.11 (q, *J* = 12 Hz, 1H), 4.38 (q, *J* = 8 Hz, 1H), 1.47 (d, *J* = 8 Hz, 1H), 0.94 (s, 9H), 0.41 (s, 3H).

➢ ^{13}C NMR (100 MHz, $CDCl_3$) δ 173.4, 151.2, 142.3, 142.52 (q, *J* = 42 Hz), 135.5, 134.0, 133.0, 132.7, 130.4, 128.2, 127.7, 126.7, 126.6, 125.9, 118.8 (q, *J* = 266 Hz), 112.3 (q, *J* = 3 Hz), 111.2, 68.4, 57.6, 25.7, 21.2, 18.4, -7.2.

➢ ^{29}Si NMR (80 MHz, $CDCl_3$) δ 10.21.

➢ IR (neat) cm $^{-1}$ 2931, 2858, 1759, 1275, 1260, 1108, 764.

➢ HRMS calcd for $C_{24}H_{27}F_3O_4Si$ $(M+Na)^+$ 487.1523 found 487.1526.

➢ $[\alpha]^{25}_D$ = -24.8 (*c* = 0.25, $CHCl_3$).

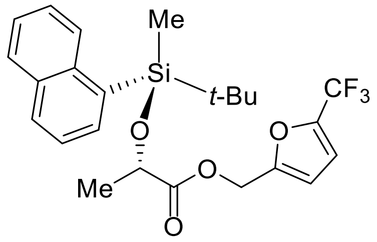

4aq

(5-(trifluoromethyl)furan-2-yl)methyl(2S)-2-((tert-butyl(methyl)(naphthalen-1-yl)silyl)oxy)propanoate (**4aq**)

4aq was prepared according to Method **6B**.

S1x (69 mg, 0.3 mmol), BPO (8 mg, 0.03 mmol), CCl_4 (3 mL), 100 °C refluxed for 21 h; then concentration followed by **2i** (48 mg, 0.2 mmol), **3e** (6 mg, 0.04 mmol) and NEt_3 (56 μL, 0.4 mmol) in dry CH_2Cl_2 (2 mL) at -78 °C for 24 h afforded **4aq** as a colorless oil (70 mg, 76%, *dr* = 93:7).

➢ R_f = 0.5 (Petroleum Ether: Ethyl Acetate = 10 : 1).

➢ ^{1}H NMR (400 MHz, $CDCl_3$) δ 8.46 (dd, *J* = 8, 4 Hz, 1H), 7.90 - 7.83 (m, 2H), 7.69 (dd, *J* = 8, 4 Hz, 1H), 7.49 - 7.44 (m, 3H), 6.77 (d, *J* = 4 Hz, 1H), 6.49 (d, *J* = 4 Hz, 1H), 5.15 (q, *J* = 12 Hz, 2H), 4.42 (q, *J* = 8 Hz, 1H), 1.44 (d, *J* = 8 Hz, 3H), 0.95 (s, 9H), 0.52 (s, 3H).

➢ ^{13}C NMR (150 MHz, $CDCl_3$) δ 173.4, 151.9, 142.4 (q, *J* = 44 Hz), 137.5, 135.1, 134.0, 133.4, 130.4, 129.8, 128.6, 125.5, 125.4, 124.7, 118.8 (q, *J* = 266 Hz), 112.3 (q, *J* = 15 Hz), 113.1, 68.7, 57.6, 26.4, 21.1, 19.3, -5.4.

➢ ^{29}Si NMR (80 MHz, $CDCl_3$) δ 12.96.

➢ IR (neat) cm $^{-1}$ 3008, 2989, 2859, 1757, 1473, 1318, 1275, 1131, 764.

➢ HRMS calcd for $C_{24}H_{27}F_3O_4Si$ $(M+Na)^+$ 487.1523 found 487.1525.

➢ $[\alpha]^{25}_D$ = -25.5 (*c* = 0.25, $CHCl_3$).

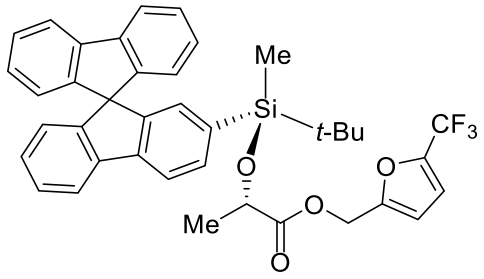

4ar

(5-(trifluoromethyl)furan-2-yl)methyl-(2S)-2-((9,9'-spirobi[fluoren]-3-yl(tert-butyl)(methyl)silyl)oxy)-propanoate (**4ar**)

4ar was prepared according to Method **6B**.

S1y (125 mg, 0.3 mmol), BPO (8 mg, 0.03 mmol), CCl_4 (3 mL), 100 °C refluxed for 21 h; then

concentration followed by **2i** (48 mg, 0.2 mmol), **3e** (6 mg, 0.04 mmol) and NEt_3 (56 μL, 0.4 mmol) in dry CH_2Cl_2 (2 mL) at -78 °C for 24 h afforded **4ar** as a colorless oil (82 mg, 63%, *dr* = 90 : 10).

- R_f = 0.7 (Petroleum Ether: Ethyl Acetate = 8: 1).
- ^{1}H NMR (400 MHz, $CDCl_3$) δ 7.85 (dd, *J* = 8, 4 Hz, 4H), 7.52 (d, *J* = 8 Hz, 1H), 7.35 (dd, *J* = 16, 8 Hz, 3H), 7.14 - 7.05 (m, 3H), 6.84 (s, 1H), 6.75 - 6.67 (m, 4H), 6.41 (d, *J* = 4 Hz, 1H), 5.03 (q, *J* = 12 Hz, 2H), 4.11 (q, *J* = 8 Hz, 1H), 1.09 (d, *J* = 8 Hz, 3H), 0.73 (s, 9H), 0.15 (s, 3H).
- ^{13}C NMR (150 MHz, $CDCl_3$) δ 173.3, 151.9, 149.0, 148.8, 148.6, 148.0, 143.1, 142.3 (q, *J* = 44 Hz), 141.7, 141.5, 135.0, 133.8, 129.9, 127.7, 127.7, 127.6, 127.6, 124.1, 123.9, 123.8, 120.2, 120.0, 119.9, 119.2, 118.8 (q, *J* = 266 Hz), 112.3 (q, *J* = 3 Hz), 111.1, 68.1, 65.9, 57.5, 25.4, 20.5, 17.9, -7.4.
- ^{29}Si NMR (80 MHz, $CDCl_3$) δ 10.52.
- IR (neat) cm $^{-1}$ 2929, 1753, 1275, 1262, 1134, 764.
- HRMS calcd for $C_{35}H_{39}F_4O_4Si$ $(M+Na)^+$ 675.2149 found 675.2152.
- $[\alpha]^{25}_D$ = -2.2 (*c* = 1.0, $CHCl_3$).

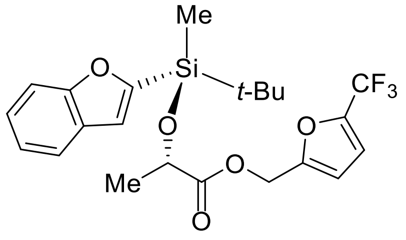

4as

(5-(trifluoromethyl)furan-2-yl)methyl-(2S)-2-((benzofuran-2-yl(tert-butyl)(methyl)silyl)oxy)propanoate (**4as**)

4as was prepared according to Method **6B**.

S1z (66 mg, 0.3 mmol), BPO (8 mg, 0.03 mmol), CCl_4 (3 mL), 100 °C refluxed for 21 h; then concentration followed by **2i** (48 mg, 0.2 mmol), **3e** (6 mg, 0.04 mmol) and NEt_3 (56 μL, 0.4 mmol) in dry CH_2Cl_2 (2 mL) at -78 °C for 24 h afforded **4as** as a colorless oil (73 mg, 80%, *dr* = 88:12).

- R_f = 0.5 (Petroleum Ether: Ethyl Acetate = 6 : 1).
- ^{1}H NMR (400 MHz, $CDCl_3$) δ 7.59 (d, *J* = 8 Hz, 1H), 7.51 (d, *J* = 8 Hz, 1H), 7.34-7.28 (m, 1H), 7.23 (dd, *J* = 12, 4 Hz, 1H), 7.07 (s, 1H), 6.71 (d, *J* = 4 Hz, 1H), 6.38 (d, *J* = 4 Hz, 1H), 5.04 (q, *J* = 12 Hz, 2H), 4.46 (q, *J* = 8 Hz, 1H), 1.45 (d, *J* = 8 Hz, 3H), 1.01 (s, 9H), 0.40 (s, 3H).
- ^{13}C NMR (100 MHz, $CDCl_3$) δ 173.1, 158.7, 158.0, 151.9, 142.2 (q, *J* = 43 Hz), 127.4, 124.8, 122.5, 121.3, 118.9, 118.8 (q, *J* = 266 Hz), 112.7 (q, *J* = 2Hz), 111.5, 112.2, 67.8, 56.6, 25.5, 20.9, 18.2, -6.7.
- ^{29}Si NMR (80 MHz, $CDCl_3$) δ 3.19.
- IR (neat) cm $^{-1}$ 3005, 2989, 1757, 1472, 1275, 1131, 764.

- HRMS calcd for $C_{22}H_{25}F_3O_5Si$ $(M+Na)^+$ 477.1316 found 477.1311..
- $[\alpha]^{25}_D$ = -70 (c = 0.5, $CHCl_3$).

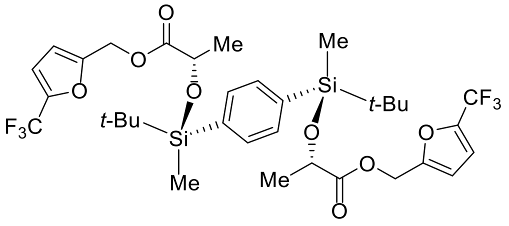

4at

1,4-bis(5-(trifluoromethyl)furan-2-yl)methyl-(2S)-2-((tert-butyl(methyl)(phenyl)silyl)oxy)propanoate (**4at**)

4at was prepared according to Method **6B**.

S1aa (84 mg, 0.3 mmol), BPO (8 mg, 0.03 mmol), CCl_4 (3 mL), 100 °C refluxed for 21 h; then concentration followed by **2i** (48 mg, 0.2 mmol), **3e** (6 mg, 0.04 mmol) and NEt_3 (56 μL, 0.4 mmol) in dry CH_2Cl_2 (2 mL) at -78 °C for 24 h afforded **4at** as a colorless oil (135 mg, 90%, dr = 84:16).

- R_f = 0.6 (Petroleum Ether: Ethyl Acetate = 6 : 1).
- ^{1}H NMR (400 MHz, $CDCl_3$) δ 7.52-7.45 (m, 2H), 6.76 (d, J = 4 Hz, 1H), 6.48 (d, J = 4 Hz, 1H), 5.11 (q, J = 12 Hz, 2H), 5.11 (q, J = 8 Hz, 1H), 1.45 (d, J = 8 Hz, 3H), 0.89 (s, 9H), 0.30 (s, 3H).
- ^{13}C NMR (150 MHz, $CDCl_3$) δ 173.4, 152.0, 136.8, 142.4 (q, J = 42 Hz), 133.4, 118.8 (q, J = 266 Hz), 112.3 (q, J = 3 Hz), 111.2, 68.3, 57.6, 25.6, 21.2, 18.2, -7.3.
- ^{29}Si NMR (80 MHz, $CDCl_3$) δ 9.89.
- IR (neat) cm $^{-1}$ 3013, 2859, 1758, 1317, 1275, 1260, 1130, 801, 763.
- HRMS calcd for $C_{33}H_{44}F_6O_8Si$ $(M+Na)^+$ 773.2371 found 773.2369.
- $[\alpha]^{25}_D$ = -62.4 (c = 0.5, $CHCl_3$).

10 g-Scale Synthesis of 4g, 6g and Transformation of 6g

Methyl (*S*)-2-(((*S*)-*tert*-butyl(methyl)(phenyl)silyl)oxy)propanoate (4g)

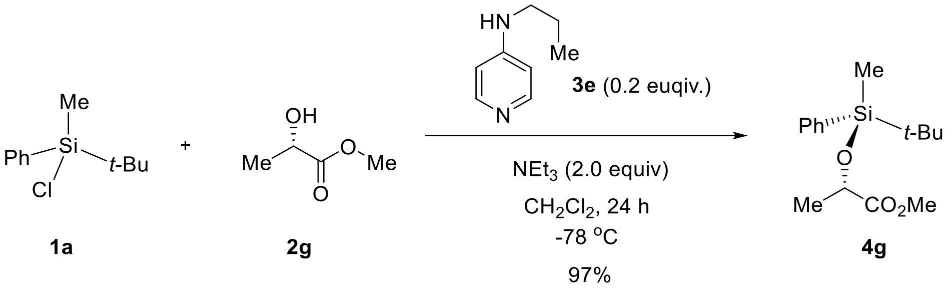

To a 1L round-bottom flask charged with **2g** (11.9 g, 50 mmol, 1.0 equiv.) in dry CH_2Cl_2 (500 mL) were added **1a** (12.7 g, 60 mmol, 1.2 equiv.), **3e** (1.36 g, 10 mmol, 20 mol%) and NEt_3 (14 mL, 100 mmol, 2.0 equiv.) under an inert atmosphere of argon at -78 °C. The mixture was stirred for 24 h at -78 °C before removing the solvent under reduced pressure. Purification by column chromatography on silica gel (gradient eluent: Petroleum Ether to Petroleum Ether /EtOAc =10 : 1) afforded the desired product **4g** as a colorless oil (13.5 g, 97%, *dr* = 92:8).

- R_f = 0.6 (Petroleum Ether: Ethyl Acetate = 8 : 1).
- ^{1}H NMR (400 MHz, $CDCl_3$) δ 7.56 - 7.54 (m, 2H), 7.39 - 7.34 (m, 3H), 4.32 (q, *J* = 8 Hz, 1H), 3.69 (s, 3H), 1.43 (d, *J* = 8 Hz, 3H), 0.93 (s, 9H), 0.37 (s, 3H).
- ^{13}C NMR (100 MHz, $CDCl_3$) δ 174.4, 135.6, 134.4, 129.5, 127.6, 68.3, 51.7, 25.7, 21.3, 18.3, -7.2.
- ^{29}Si NMR (80 MHz, $CDCl_3$) δ 9.42.
- IR (neat) cm $^{-1}$ 3070, 2953, 2857, 1758, 1590, 1426, 1254, 1110, 976, 801.
- HRMS calcd for $C_{15}H_{24}O_3Si$ $(M+Na)^+$ 303.1387 found 303.1391.
- $[\alpha]^{25}_D$ = -58 (*c* = 0.5, $CHCl_3$).

***S*-2-(((*S*)-*tert*-butyl(methyl)(phenyl)silyl)oxy)propanoic acid (6g)**[12]

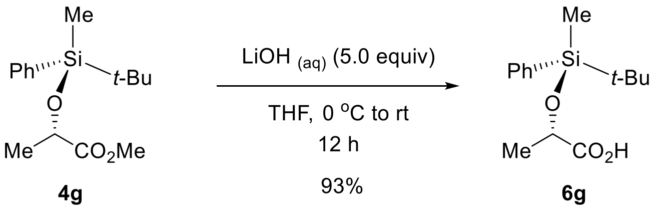

To a 1L round-bottom flask charged with an aqueous solution of lithium hydroxide (80 mL, 5.0 equiv., 3 M) was added dropwise a solution of **4g** (13.5 g, 48 mmol, 1.0 equiv.) in dry THF (500 mL) at 0 °C. The mixture was stirred at room temperature for 12 h. Finally, the solution was adjusted

pH to 4-5 with aq. $KHSO_4$ (3 M). The organic layers were separated and the aqueous layer was extracted with CH_2Cl_2 (100 mL × 3). The combined organic layers were washed with sat. aq. NaCl, dried over Na_2SO_4 and concentrated under reduced pressure. Purification by column chromatography on silica gel (gradient eluent: Petroleum Ether to Petroleum Ether /EtOAc = 3 : 1) afforded the desired product as a white solid (11.9 g, 93%, *dr* = 92:8).

- mp: 65.6 - 76.3 °C.
- R_f = 0.4 (Petroleum Ether: Ethyl Acetate = 4 : 1).
- ^1H NMR (400 MHz, $CDCl_3$) δ 7.56 - 7.54 (m, 2H), 7.41 (dd, *J* = 12, 8 Hz, 3H), 4.37 (q, *J* = 8 Hz, 1H), 1.43 (d, *J* = 8 Hz, 3H), 0.95 (s, 9H), 0.44 (s, 3H).
- ^{13}C NMR (100 MHz, $CDCl_3$) δ 175.4, 134.4, 134.3, 130.0, 127.8, 68.9, 25.7, 21.0, 18.3, -7.4.
- ^{29}Si NMR (80 MHz, $CDCl_3$) δ 8.95.
- IR (neat) cm^{-1} 3394, 3070, 2954, 2857, 1722, 1463, 1255, 1111, 823, 764.
- HRMS calcd for $C_{14}H_{22}O_3Si$ $(M+Na)^+$ 289.1230 found 289.1231.
- $[\alpha]^{25}_D$ = -36.4 (*c* = 0.5, $CHCl_3$).

6g (11.9 g) was recrystallized in Et_2O (60 mL) at 25 °C for 12 h afforded colorless crystals (10.1 g, 84%, *dr* ≥ 99 :1), which were used for X-ray analysis.

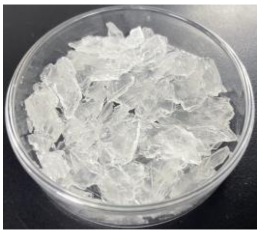

(S)-tert-butyl(butyl)(methyl)(phenyl)silane **(7)**[13]

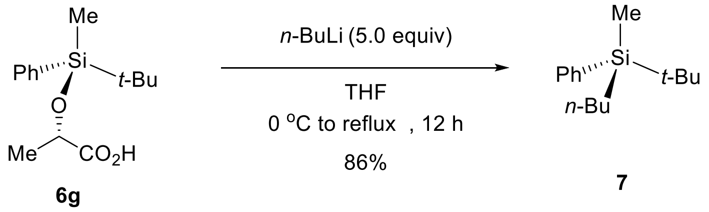

To a solution of **6g** (266 mg, 1 mmol, 1.0 equiv.) in dry THF (10 mL) was added *n*-BuLi (2 mL, 5 mmol, 2.4 M in hexane, 5.0 equiv.) dropwise at 0 °C under argon atmosphere. The mixture was heated to 70 °C and refluxed overnight. The reaction was cooled down to room temperature and quenched with H_2O (5 mL). The organic layers were separated and the aqueous layer was extracted with EtOAc (10 mL × 3). The combined organic layers were washed with sat. aq. NaCl, dried over Na_2SO_4 and concentrated under reduced pressure. Purification by column chromatography on silica gel (gradient eluent: Petroleum Ether = 100%) afforded **7** (201 mg, 86%, *er* = 98:2) as a colorless oil.

- HPLC (Phenomenex lux cellulose-3, solvent MeOH: H_2O = 98:2, flow rate 0.5 mL/min, T = 25 °C): t_R= 10.0 min (minor enantiomer), t_R= 15.8 min (major enantiomer).
- R_f = 0.9 (Petroleum Ether).
- ^{1}H NMR (400 MHz, $CDCl_3$) δ 7.52 - 7.50 (m, 2H), 7.38 - 7.34 (m, 3H), 1.39 - 1.24 (m, 5H), 1.03 - 0.91 (m, 4H), 0.89 (s, 9H), 0.29 (s, 3H).
- ^{13}C NMR (150 MHz, $CDCl_3$) δ 136.7, 134.7, 128.6, 127.4, 26.9, 26.8, 26.3, 17.1, 13.8, 10.1, -8.5.
- ^{29}Si NMR (80 MHz, $CDCl_3$) δ 3.47.
- IR (neat) cm $^{-1}$ 3069, 2954, 2855, 1463, 1108, 822, 779.
- HRMS calcd for $C_{15}H_{26}Si$ $(M+H)^+$ 235.1877 found 235.1880.
- $[\alpha]^{25}_D$ = -7.8 (*c* = 0.5, $CHCl_3$).

(S)-tert-butyl(methyl)(phenyl)(p-tolyl)silane **(8)**

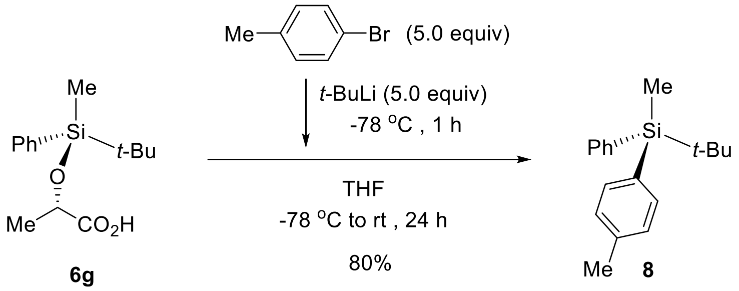

To a 50 mL round-bottom flask charged with 1-bromo-4-methylbenzene (855 mg, 5 mmol, 5.0 equiv.) in dry THF (20 mL) under an inert atmosphere of argon at -78 °C, freshly titrated *t*-BuLi (4 mL, 5 mmol, 1.3 M in pentane, 5.0 equiv.) was added carefully and the mixture was stirred for 1 h at -78 °C. Then a solution of **6g** (266 mg, 1 mmol, 1.0 equiv.) in THF (5 mL) was added at -78 °C and the resulting mixture was slowly warmed up to room temperature and stirred for 12 h. Finally, the reaction was cooled down to room temperature and quenched with H_2O (5 mL). The organic layers were separated and the aqueous layer was extracted with EtOAc (10 mL × 3). The combined organic layers were washed with sat. aq. NaCl, dried over Na_2SO_4 and concentrated under reduced pressure. Purification by column chromatography on silica gel (gradient eluent: gradient eluent: Petroleum Ether = 100%) afforded **8** (214 mg, 80%, *er* = 99:1) as a colorless oil.

- HPLC (Daicel Chiralcel OD-H column, solvent *n*-hexane, flow rate 0.5 mL/min, T = 16 °C): t_R= 11.7 min (major enantiomer), t_R= 12.8 min (minor enantiomer).
- R_f = 0.8 (Petroleum Ether).
- ^{1}H NMR (400 MHz, $CDCl_3$) δ 7.72 - 7.69 (m, 2H), 7.62 - 7.60 (m, 2H), 7.43 - 7.41 (m, 3H), 7.26 (d, *J* = 7.7 Hz, 2H), 2.43 (s, 3H), 1.12 (s, 9H), 0.64 (s, 3H).

➢ ^{13}C NMR (100 MHz, $CDCl_3$) δ 138.8, 136.4, 135.4, 135.3, 132.5, 128.9, 128.5, 127.5, 27.5, 21.44, 17.9, -6.2.

➢ ^{29}Si NMR (80 MHz, $CDCl_3$) δ -3.69.

➢ IR (neat) cm^{-1} 3070, 2989, 2855, 1603, 1275, 1260, 1100, 764.

➢ HRMS calcd for $C_{18}H_{24}Si$ $(M+Na)^+$ 291.1539 found 291.1535.

➢ $[\alpha]^{25}_D$ = 6.8 (c = 0.5, $CHCl_3$).

<u>(S)-tert-butyl(methyl)(phenyl)(prop-1-yn-1-yl)silane</u> **(9)**

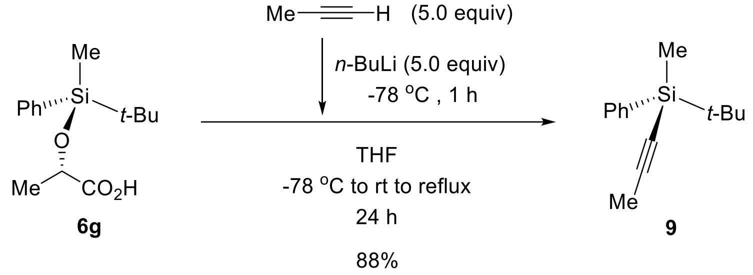

To a 50 mL round-bottom flask charged with propyne (5 mL, 5 mmol, 5.0 equiv., 1 M in THF) in dry THF (20 mL) under an inert atmosphere of argon at -78 °C, freshly titrated *n*-BuLi (2 mL, 5 mmol, 2.4 M in hexane, 5.0 equiv.) was added carefully and the mixture was stirred for 1 h at -78 °C. Then a solution of **6g** (266 mg, 1 mmol, 1.0 equiv.) in THF (5 mL) was added at -78 °C, the resulting mixture was slowly warmed up to room temperature and stirred for 12 h. Finally, the reaction was cooled down to room temperature and quenched with H_2O (5 mL). The organic layer was separated and the aqueous layer was extracted with EtOAc (10 mL × 3). The combined organic layers were washed with sat. aq. NaCl, dried over Na_2SO_4 and concentrated under reduced pressure. Purification by column chromatography on silica gel (gradient eluent: gradient eluent: Petroleum Ether = 100%) afforded **9** (190 mg, 88%, *er* = 98:2) as a colorless oil.

➢ HPLC (Daicel Chiralcel OD-H column, solvent *n*-hexane, flow rate 0.5 mL/min, T = 16 °C): t_R= 15.0 min (minor enantiomer), t_R= 16.0 min (major enantiomer).

➢ R_f = 0.6 (Petroleum Ether).

➢ ^1H NMR (400 MHz, $CDCl_3$) δ 7.71 - 7.65 (m, 2H), 7.42 - 7.34 (m, 3H), 2.00 (s, 3H), 0.97 (s, 9H), 0.42 (s, 3H).

➢ ^{13}C NMR (100 MHz, $CDCl_3$) δ 135.2, 134.8, 129.1, 127.4, 105.2, 79.8, 26.2, 17.3, 5.0, -6.0.

➢ ^{29}Si NMR (80 MHz, $CDCl_3$) δ -12.98.

➢ IR (neat) cm^{-1} 3005, 2954, 2856, 2181, 1471, 1260, 1109, 783.

➢ HRMS calcd for $C_{14}H_{20}Si$ $(M+Na)^+$ 239.1266 found 239.1263.

➢ $[\alpha]^{25}_D$ = -11.4 (c = 1.0, $CHCl_3$).

(S)-tert-butyl(furan-2-yl)(methyl)(phenyl)silane **(10)**

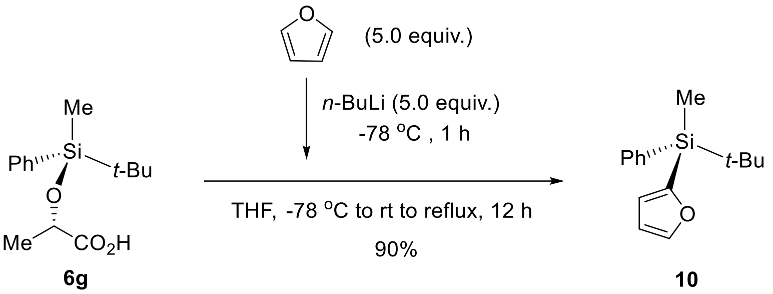

To a 50 mL round-bottom flask charged with furan (363 μL, 5 mmol, 5.0 equiv.) in dry THF (20 mL) under an inert atmosphere of argon at -78 °C, freshly titrated *n*-BuLi (2 mL, 5 mmol, 2.4 M in hexane, 5.0 equiv.) was added carefully and the mixture was stirred for 1 h at -78 °C. Then a solution of **6g** (266 mg, 1 mmol, 1.0 equiv.) in THF (5 mL) was added at -78 °C and the resulting mixture was slowly warmed up to room temperature and stirred for 12 h. Finally, the reaction was cooled down to room temperature and quenched with H_2O (5 mL). The organic layers were separated and the aqueous layer was extracted with EtOAc (10 mL × 3). The combined organic layers were washed with sat. aq. NaCl, dried over Na_2SO_4 and concentrated under reduced pressure. Purification by column chromatography on silica gel (gradient eluent: gradient eluent: Petroleum Ether = 100%) afforded **10** (219 mg, 90%, *er* = 99:1) as a colorless oil.

- HPLC (Daicel Chiralcel OD-H column, solvent *n*-hexane, flow rate 0.5 mL/min, T = 20 °C): t_R= 12.5 min (major minor enantiomer), t_R= 14.3 min (minor enantiomer).
- R_f = 0.6 (Petroleum Ether).
- ^{1}H NMR (400 MHz, $CDCl_3$) δ 7.77 (d, *J* = 1.6 Hz, 1H), 7.67 (dd, *J* = 7.5, 2.0 Hz, 2H), 7.44 - 7.34 (m, 3H), 6.81 (d, *J* = 3.3 Hz, 1H), 6.46 (dd, *J* = 3.3, 1.7 Hz, 1H), 1.03 (s, 9H), 0.59 (s, 3H).
- ^{13}C NMR (100 MHz, $CDCl_3$) δ 156.8, 147.1, 135.0, 129.2, 127.5, 122.2, 109.3, 26.7, 17.6, -7.5.
- ^{29}Si NMR (80 MHz, $CDCl_3$) δ -9.25.
- IR (neat) cm $^{-1}$ 3071, 2929, 2856, 1549, 1427, 1361, 1254, 1107, 821, 783.
- HRMS calcd for $C_{15}H_{20}OSi$ $(M+H)^+$ 245.1356 found 245.1360.
- $[\alpha]^{25}_D$ = 10 (*c* = 1.0, $CHCl_3$).

(S)-Allyl(tert-butyl)(methyl)(phenyl)silane **(11)**

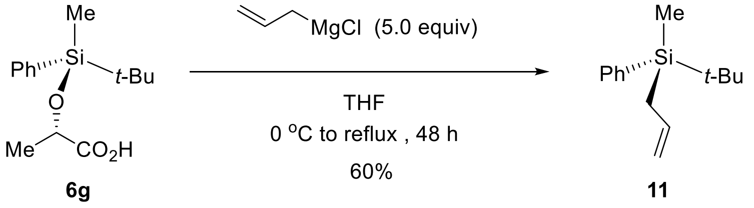

To a solution of **6g** (266 mg, 1 mmol, 1.0 equiv.) in dry THF (10 mL) under argon atmosphere was added allylmagnesium chloride (5 mL, 5 mmol, 1.0 M solution in THF 5.0 equiv.) dropwise at 0 °C. Then the reaction mixture was subsequently heated to 70 °C and stirring for 48 h. Finally, the reaction was cooled down to room temperature and quenched with H_2O (5 mL). The organic layer was separated and the aqueous layer was extracted with EtOAc (10 mL × 3). The combined organic layers were washed with sat. aq. NaCl, dried over Na_2SO_4 and concentrated under reduced pressure. Purification by column chromatography on silica gel (gradient eluent: gradient eluent: Petroleum Ether = 100%) afforded **11** (130 mg, 60%, *er* = 98:2) as a colorless oil.

- HPLC (Phenomenex lux cellulose-3, solvent MeOH: H_2O = 94:6, flow rate 0.5 mL/min, T = 25 °C): t_R= 16.5 min (minor enantiomer), t_R= 18.2 min (major enantiomer).
- R_f = 0.8 (Petroleum Ether).
- ^{1}H NMR (400 MHz, $CDCl_3$) δ 7.53 - 7.51 (m, 2H), 7.38 - 7.34 (m, 3H), 5.78 (dddd, *J* = 16, 12, 8, 4 Hz, 1H), 4.90 (dq, *J* = 16, 4 Hz, 1H), 4.81 (ddt, *J* = 10, 4, 1.0 Hz, 1H), 1.97 (ddt, *J* = 12, 8, 1 Hz, 1H), 1.85 (ddt, *J* = 12, 8, 1 Hz, 1H), 0.91 (s, 9H), 0.29 (s, 3H).
- ^{13}C NMR (100 MHz, $CDCl_3$) δ 135.9, 134.9, 134.7, 128.9, 127.5, 113.6, 26.8, 18.7, 17.4, -8.7.
- ^{29}Si NMR (80 MHz, $CDCl_3$) δ 1.51.
- IR (neat) cm $^{-1}$ 3070, 2929, 2885, 2112, 1630, 1253, 1108, 808.
- HRMS calcd for $C_{14}H_{22}Si$ $(M+Na)^+$ 241.1383 found 241.1385.
- $[\alpha]^{25}_D$ = -2.4 (*c* = 0.5, $CHCl_3$).

(R)-tert-butyl(methyl)(phenyl)silane **(12)**[14, 15]

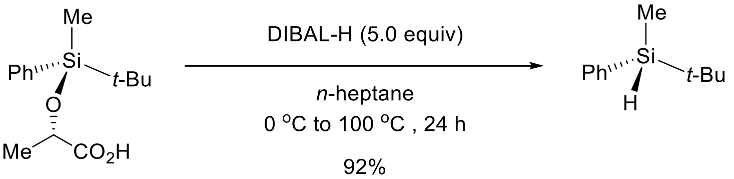

6g **12**

To a solution of **6g** (266 mg, 1 mmol, 1.0 equiv.) in dry *n*-heptane (10 mL) under argon atmosphere was added DIBAL-H (5 mL, 5 mmol, 1.0 M in hexane, 5.0 equiv.) dropwise at 0 °C. The reaction mixture was heated to 100 °C and refluxed overnight. The reaction was cooled down to room temperature and quenched with H_2O (5 mL) followed by neutralization with 10% aq. HCl. The organic layers were separated and the aqueous layer was extracted with EtOAc (10 mL × 3). The combined organic layers were washed with sat. aq. NaCl, dried over Na_2SO_4 and concentrated under reduced pressure. Purification by column chromatography on silica gel (gradient eluent: gradient eluent: Petroleum Ether = 100%) afforded **12** (163 mg, 92%, *er* = 98:2) as a colorless oil.

- HPLC (Phenomenex lux cellulose-3, solvent MeOH: H_2O = 98:2, flow rate 0.5 mL/min, T =

25 °C): t_R= 15.0 min (major enantiomer), t_R= 19.6 min (minor enantiomer).

- R_f = 0.9 (Petroleum Ether).
- ^{1}H NMR (400 MHz, $CDCl_3$) δ 7.58 - 7.56 (m, 2H), 7.41 - 7.36 (m, 3H), 4.18 (q, *J* = 4 Hz, 1H), 0.98 (s, 9H), 0.38 (d, *J* = 4 Hz, 3H).
- ^{13}C NMR (100 MHz, CDCl3) δ 135.5, 135.1, 129.2, 127.6, 26.8, 16.6, -8.5.
- ^{29}Si NMR (80 MHz, $CDCl_3$) δ -1.36.
- IR (neat) cm $^{-1}$ 3069, 2855, 2108, 1470, 1362, 1275, 1260, 1115, 1008, 871.
- HRMS calcd for $C_{11}H_{18}Si$ $(M+Na)^+$ 201.1079 found 201.1075.
- $[\alpha]^{25}_D$ = -8.4 (*c* = 1.5, $CHCl_3$).
- The optical rotation data is in accordance with in the literature[16]. $[\alpha]^{25}_D$ = -2.4 (c = 1.0, $CHCl_3$).

(R)-tert-butyl(methyl)(phenyl)silane-d **(13)**

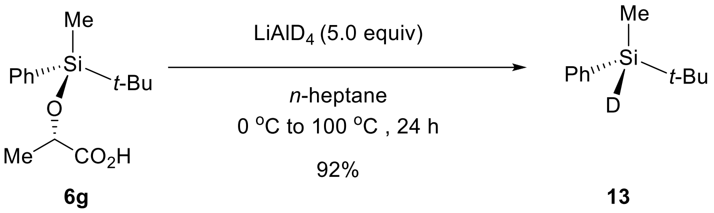

To a solution of **6g** (266 mg, 1 mmol, 1.0 equiv.) in dry *n*-heptane (10 mL) under argon atmosphere was added $LiAlD_4$ (210mg, 5 mmol, 5.0 equiv.) at 0 °C. The reaction mixture was subsequently heated to 100 °C and refluxed overnight. The reaction was cooled down to room temperature and quenched with H_2O (5 mL) followed by neutralization with aq. 10% HCl. The organic layers were separated and the aqueous layer was extracted with EtOAc (10 mL × 3). The combined organic layers were washed with sat. aq. NaCl, dried over Na_2SO_4 and concentrated under reduced pressure. Purification by column chromatography on silica gel (gradient eluent: gradient eluent: Petroleum Ether = 100%) afforded **13** (160 mg, 90%, *er* = 98:2) as a colorless oil.

- HPLC (Phenomenex lux cellulose-3, solvent MeOH: H_2O = 98:2, flow rate 0.5 mL/min, T = 25 °C): t_R= 15.0 min (major minor enantiomer), t_R= 19.8 min (minor enantiomer).
- R_f = 0.9 (Petroleum Ether).
- ^{1}H NMR (400 MHz, $CDCl_3$) δ 7.74 - 7.44 (m, 2H), 7.32 - 7.25 (m, 3H), 0.88 (s, 9H), 0.48 (s, 3H).
- ^{13}C NMR (150 MHz, $CDCl_3$) δ 135.4, 135.0, 129.2, 127.6, 26.8, 16.5, -8.6.
- ^{29}Si NMR (80 MHz, $CDCl_3$) δ -1.79 (t, *J* = 28 Hz).
- IR (neat) cm $^{-1}$ 2920, 2850, 1631, 1471, 1262, 1018, 764.
- HRMS calcd for $C_{11}H_{17}DSi$ $(M+H)^+$ 180.1313 found 180.1315.
- $[\alpha]^{25}_D$ = -9 (*c* = 1.1, $CHCl_3$).

In order to determine the absolute configuration of derivative products, we designed the following experiments:

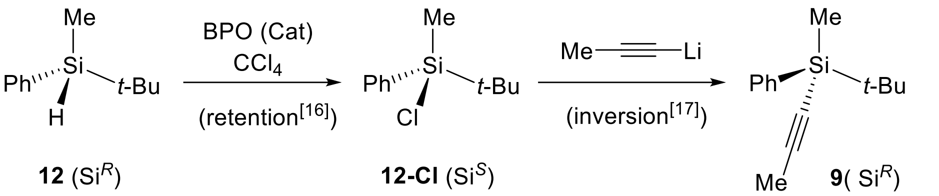

12 (Si^R) **12-Cl** (Si^S) **9**(Si^R)

$[\alpha]^{25}_D$ = + 6 (c = 1.0, $CHCl_3$)

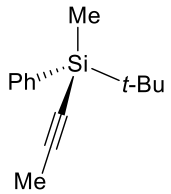

9 (Si^S) from **6g**

$[\alpha]^{25}_D$ = - 11.4 (c = 1.0, $CHCl_3$)

To a 10 mL round-bottom flask charged with **12** (54 mg, 0.3 mmol, 1.0 equiv.) and CCl_4 (3 mL) was added benzoyl peroxide (8 mg, 0.03 mmol). The mixture was refluxed for 21 h followed by stirring for 3 h at room temperature. The solvent of the mixture was removed under reduced pressure. The crude product **12-Cl** was used without purification in the next step.

To a 10 mL round-bottom flask charged with propyne (1.5 mL, 1.5 mmol, 5.0 equiv., 1 M in THF) in dry THF (4 mL) under an inert atmosphere of argon at -78 °C, freshly titrated *n*-BuLi (0.6 mL, 1.5 mmol, 2.4 M in hexane, 5.0 equiv.) was added carefully and the mixture was stirred for 1 h at -78 °C. Then a solution of the crude **12-Cl** in THF (1 mL) was added at -78 °C. The resulting mixture was slowly warmed up to room temperature and stirred for 12 h. The reaction was cooled down to 0 °C and quenched with H_2O (2 mL). The organic layers were separated and the aqueous layer was extracted with EtOAc (5 mL × 3). The combined organic layers were washed with sat. aq. NaCl, dried over Na_2SO_4 and concentrated under reduced pressure. Purification by column chromatography on silica gel (gradient eluent: gradient eluent: Petroleum Ether = 100%) afforded **9** (Si^R) (48 mg, 75%) as a colorless oil. $[\alpha]^{25}_D$ = + 6 (c = 1.0, $CHCl_3$).

General Experimental Procedure for Kinetic Experiments

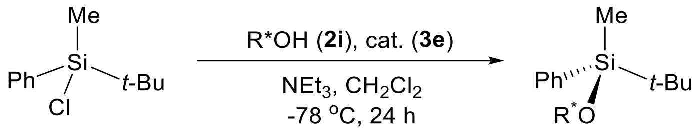

1a (*racemic*) **4i**

$$\mathrm{d}[\mathbf{4i}]/\mathrm{d}t = k[\mathbf{1a}]^1[\mathbf{3e}]^1[\mathbf{2i}]^0[NEt_3]^0$$

Experimental Procedure:

To a 100 mL round-bottomed flask charged with **2i**, **3e**, NEt_3, 1,3,5-trimethoxybenzene (TMB) and CH_2Cl_2 (50 mL) at -78 °C, **1a** was added quickly in one portion (The amounts of each of the reagents used for the different kinetic experiments are shown in **Supplementary Table 1**). At different time intervals, 1 mL was pulled from the reaction vessel and quenched in a vial containing 3 mL of water. 200 μL was pulled from organic phase and concentrated under reduced pressure. All crude products were dissolved $CDCl_3$ for ^{1}H NMR spectroscopy analysis. The yields of **4i** were was determined by ^{1}H NMR of the crude mixture using 1,3,5-trimethoxybenzene (TMB) as an internal standard.

Supplementary Table 1: Reaction Composition for Different Kinetic Experiments.

Entry	Experiment Description	1a	2i, 3e, NEt_3 and TMB
1	Standard Conditions	1.3 g	**2i** (1.3 g), **3e** (140 mg), NEt_3 (1.6 mL), and TMB (856 mg)
2	½ [**1a**]	0.65 g	**2i** (1.3 g), **3e** (140 mg), NEt_3 (1.6 mL), and TMB (856 mg)
3	½ [**2i**]	1.3 g	**2i** (0.65 g), **3e** (140 mg), NEt_3 (1.6 mL), and TMB (856 mg)
4	½ [**NEt_3**]	1.3 g	**2i** (1.3 g), **3e** (140 mg), NEt_3 (0.8 mL), and TMB (856 mg)
5	2.0 equiv. [**3e**]	1.3 g	**2i** (0.65 g), **3e** (280 mg), NEt_3 (1.6 mL), and TMB (856 mg)

Kinetic Data

➢ First-order Kinetics in [1a]

Comparison of the slopes of reaction profiles plotted for [**4i**] as a function of time for reactions with the same $[\mathbf{2i}]_0$ and different values of $[\mathbf{1a}]_0$ gives the dependence on [**1a**]. The fact that the

profiles nearly slower rates with decreasing concentration of [**1a**] at lower reaction conversion, suggests first-order kinetics in [**1a**].

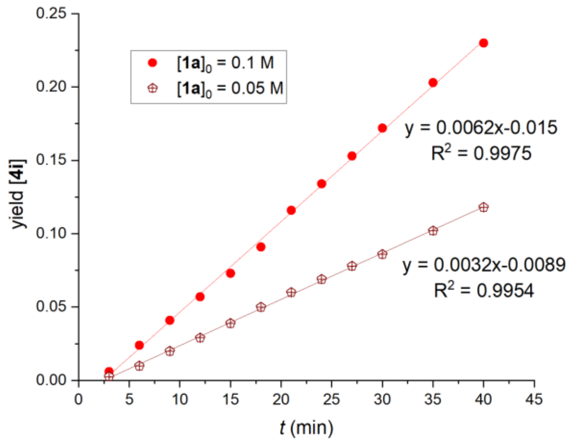

Supplementary Figure 1. Temporal concentration profiles for the standard reaction at two different [**1a**]$_0$: [**1a**]$_0$ as noted; [**2i**]$_0$ = 0.1 M; [NEt_3]$_0$ = 0.2 M; [**3e**]$_0$ = 0.02 M.

➢ Saturation Kinetics in [2i]

Comparison of the slopes of reaction profiles plotted for [**4i**] as a function of time for reactions with the same [**1a**]$_0$ and different values of [**2i**]$_0$ reveals the dependence on [**2i**]. The initial rates for the three reactions gave saturation kinetics in [**2i**].

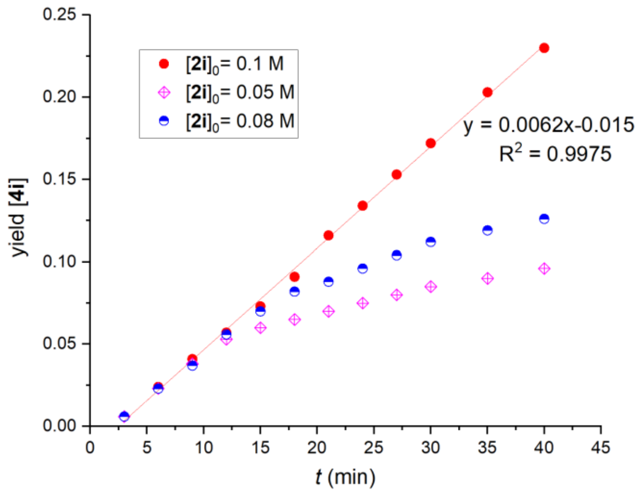

Supplementary Figure 2. Temporal concentration profiles for the standard reaction at three different [**2i**]$_0$: [**2i**]$_0$: [**2i**]$_0$ as noted; [**1a**]$_0$ = 0.1 M; [**NEt_3**]$_0$ = 0.2 M; [**3e**]$_0$ = 0.02 M.

➢ First-order Kinetics in [3e]

Comparison of the slopes of reaction profiles plotted for [**4i**] as a function of time for reactions with the same [**1a**]$_0$ and [**2i**]$_0$ but different values of [**3e**]$_0$ gives the dependence on [**3e**]. The fact that the profiles nearly faster rates with increasing concentration of [**3e**] at higher reaction conversion, suggests first-order kinetics in [**3e**].

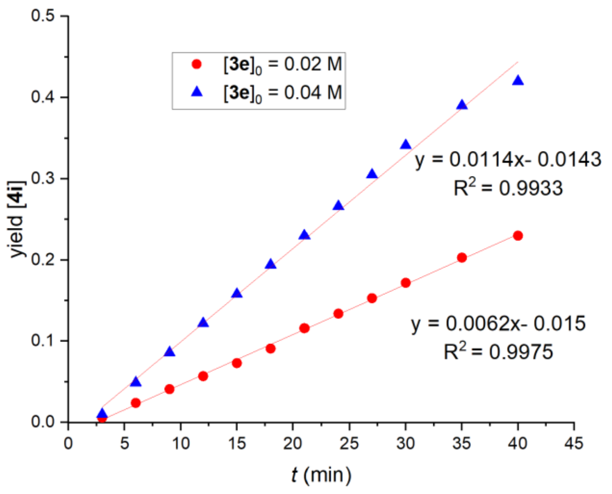

Supplementary Figure 3. Temporal concentration profiles for the standard reaction at two different catalyst loadings. [**3e**]$_0$: [**3e**]$_0$ as noted; [**1a**] = 0.1 M; [**2i**] = 0.1 M; [**NEt$_3$**] = 0.2 M.

➢ Saturation Kinetics in [NEt$_3$]

Comparison of the slopes of reaction profiles plotted for [**4i**] as a function of time for reactions with same [**1a**]$_0$ and [**2i**]$_0$ but different values of [**NEt$_3$**]$_0$ provides the dependence on [**NEt$_3$**]. The fact that the profiles nearly overlay at lower reaction conversion, suggests saturation kinetics in [**NEt$_3$**].

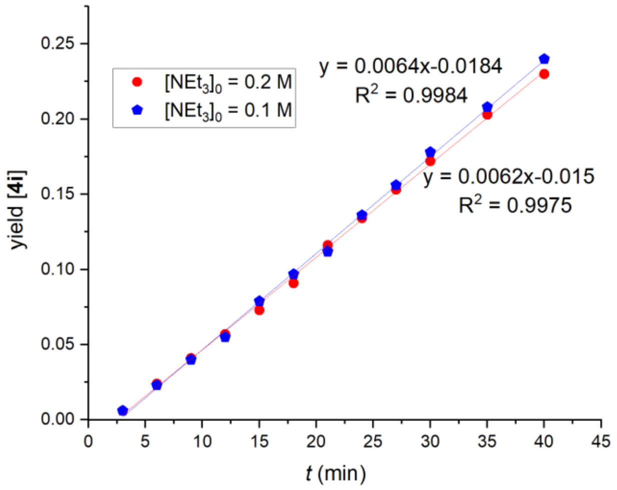

Supplementary Figure 4. Temporal concentration profiles for the standard reaction at two different [**NEt$_3$**]$_0$: [**NEt$_3$**]$_0$ as noted; **[1a]**$_0$ = 0.1 M; **[2i]**$_0$ = 0.1 M; **[3e]**$_0$ = 0.02 M.

1.3 Crystallographic Data of 6g

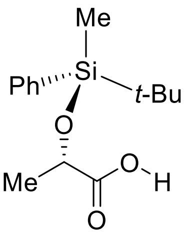

6g

Datablock cu_szl_htb_19_81_1_0m_a - ellipsoid plot

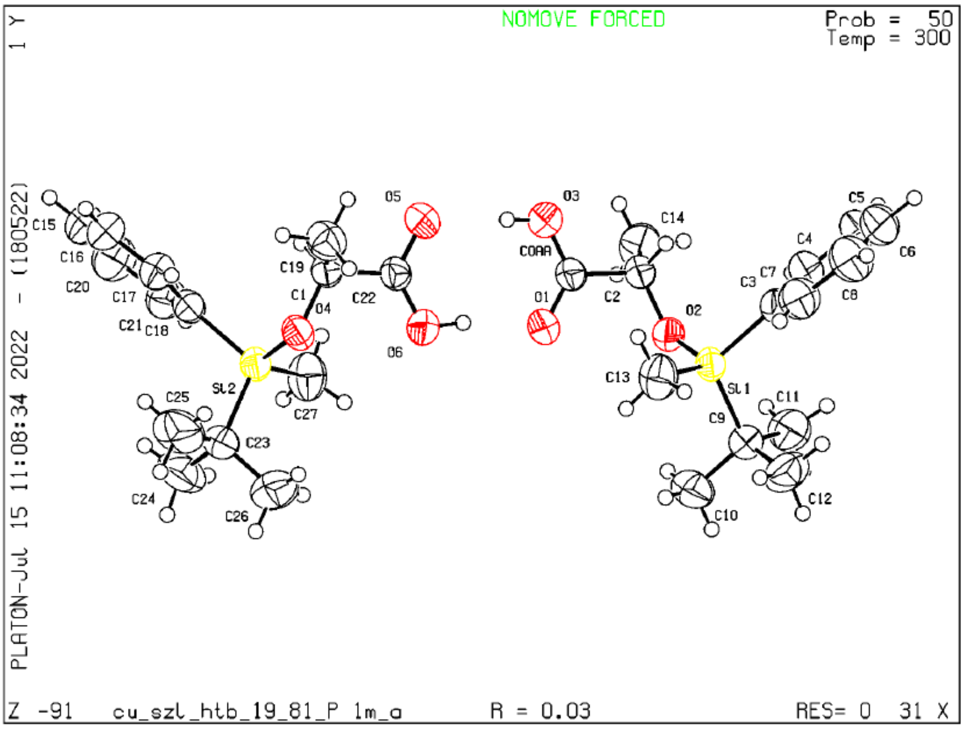

Supplementary Figure 5. X-ray of 6g

Supplementary Table 2. Crystal data and structure refinement for cu_szl_htb_19_81_1_0m_a.

Identification code	cu_szl_htb_19_81_1_0m_a
Empirical formula	$C_{14}H_{22}O_3Si$
Formula weight	266.40
Temperature/K	300.0
Crystal system	triclinic
Space group	P1
a/Å	8.5151(9)

b/Å	9.1986(9)
c/Å	11.9741(12)
α/°	95.386(4)
β/°	103.171(4)
γ/°	116.829(4)
Volume/Å^3	793.76(14)
Z	2
ρ_{calc}g/cm^3	1.115
μ/mm^{-1}	1.299
F(000)	288.0
Crystal size/mm^3	0.25 × 0.17 × 0.04
Radiation	CuKα (λ = 1.54178)
2Θ range for data collection/°	7.784 to 134.086
Index ranges	-10 ≤ h ≤ 10, -10 ≤ k ≤ 10, -14 ≤ l ≤ 14
Reflections collected	12513
Independent reflections	5272 [R_{int} = 0.0393, R_{sigma} = 0.0487]
Data/restraints/parameters	5272/5/343
Goodness-of-fit on F^2	1.033
Final R indexes [I>=2σ (I)]	R_1 = 0.0298, wR_2 = 0.0806
Final R indexes [all data]	R_1 = 0.0307, wR_2 = 0.0816
Largest diff. peak/hole / e Å^{-3}	0.19/-0.14
Flack parameter	0.056(11)

Supplementary Table 3. Fractional Atomic Coordinates (×10^4) and Equiv.alent Isotropic Displacement Parameters (Å^2×10^3) for cu_szl_htb_19_81_1_0m_a. U_{eq} is defined as 1/3 of of the trace of the orthogonalised U_{IJ} tensor.

**Atom**	***x***	***y***	***z***	**U(eq)**
Si1	427.1(7)	-623.0(6)	2909.5(5)	47.36(16)
Si2	7417.2(7)	10664.6(6)	7042.4(5)	48.84(17)
O1	3860(3)	3692(3)	4064(2)	75.0(6)
O2	1711(2)	897(2)	2340.7(15)	51.4(4)
O3	6413(3)	3616(3)	4034(2)	76.9(6)

O4	7604(2)	9128.2(19)	7570.2(15)	53.0(4)
O5	8327(3)	6357(3)	5731(2)	72.1(5)
O6	5761(3)	6313(3)	5866(2)	75.9(6)
C1	8647(3)	8426(3)	7234(2)	50.0(5)
C0AA	4685(3)	3071(3)	3645(2)	49.4(5)
C2	3644(3)	1579(3)	2599(2)	50.9(5)
C3	854(4)	-2413(3)	2587(3)	57.9(6)
C4	1192(4)	-2777(4)	1545(3)	70.1(7)
C5	1385(5)	-4182(5)	1264(4)	92.5(12)
C6	1250(6)	-5213(5)	2035(5)	104.7(15)
C7	758(5)	-3494(4)	3348(4)	79.7(9)
C8	937(7)	-4879(5)	3058(5)	102.0(13)
C9	-2034(4)	-1234(3)	2138(2)	58.0(6)
C10	-2376(5)	243(4)	2358(3)	80.2(9)
C11	-2498(5)	-1835(5)	802(3)	85.6(10)
C12	-3298(5)	-2663(5)	2617(4)	88.9(10)
C13	1026(5)	90(4)	4529(3)	75.7(9)
C14	4192(5)	2121(5)	1519(3)	79.6(9)
C15	13341(5)	15225(4)	7871(4)	87.1(11)
C16	12940(5)	14202(4)	8636(3)	78.4(9)
C17	11194(4)	12828(3)	8372(3)	64.2(7)
C18	9804(4)	12453(3)	7338(2)	54.8(6)
C19	9470(5)	7890(4)	8269(3)	69.3(7)
C20	12005(6)	14889(5)	6840(4)	90.5(11)
C21	10259(5)	13516(4)	6575(3)	72.3(8)
C22	7483(3)	6929(3)	6200(2)	49.0(5)
C23	6103(4)	11284(3)	7869(3)	63.1(7)
C24	5923(7)	12716(6)	7391(5)	104.4(14)
C25	7103(7)	11851(7)	9178(3)	105.7(14)
C26	4157(6)	9850(6)	7639(5)	99.1(12)
C27	6211(6)	9970(5)	5431(3)	78.9(10)

Supplementary Table 4. Anisotropic Displacement Parameters ($Å^2×10^3$) for cu_szl_htb_19_81_1_0m_a. The Anisotropic displacement factor exponent takes the form: -$2π^2[h^2a^{*2}U_{11}+2hka^*b^*U_{12}+…]$.

**Atom**	**U_{11}**	**U_{22}**	**U_{33}**	**U_{23}**	**U_{13}**	**U_{12}**
Si1	49.1(3)	42.1(3)	46.7(3)	5.3(2)	11.4(2)	21.2(3)
Si2	49.9(3)	43.1(3)	49.5(3)	5.6(2)	15.1(3)	20.4(3)
O1	57.9(11)	59.4(11)	94.0(14)	-16.4(10)	14.7(10)	27.6(9)
O2	49.3(9)	45.4(8)	55.5(9)	9.0(6)	12.9(7)	21.7(7)
O3	51.7(11)	63.0(12)	100.3(16)	-8.8(11)	9.4(11)	26.5(9)
O4	60.7(10)	44.6(8)	57.8(9)	9.2(6)	25.7(8)	25.9(7)
O5	63.8(12)	64.2(11)	84.7(13)	-5.9(9)	27.3(10)	30.2(10)
O6	52.8(11)	59.9(11)	94.4(15)	-16.0(10)	20.5(10)	17.7(9)
C1	50.1(13)	42.9(11)	56.1(12)	7.9(9)	20.6(10)	20.4(10)
C0AA	49.3(13)	40.5(10)	60.0(13)	10.8(9)	19.1(11)	22.2(10)
C2	51.4(13)	42.8(11)	58.1(13)	7.5(9)	18.8(11)	22.4(10)
C3	49.9(13)	45.2(12)	71.9(15)	6.9(11)	10.3(12)	22.7(11)
C4	66.4(18)	59.7(16)	80.6(19)	0.0(13)	16.6(15)	33.4(14)
C5	75(2)	77(2)	115(3)	-18(2)	21(2)	39.8(18)
C6	79(2)	56.8(18)	176(5)	11(2)	19(3)	43.1(17)
C7	76(2)	63.4(17)	106(2)	30.3(16)	26.3(18)	37.4(16)
C8	101(3)	67(2)	152(4)	39(2)	34(3)	52(2)
C9	51.3(14)	54.6(13)	64.6(14)	7.6(11)	15.2(12)	25.2(11)
C10	82(2)	80(2)	95(2)	18.2(17)	29.7(18)	51.2(18)
C11	74(2)	100(2)	68.3(18)	-3.4(17)	-1.1(15)	44.7(19)
C12	58.5(18)	75(2)	125(3)	34.0(19)	31.3(19)	22.5(15)
C13	85(2)	74.6(18)	53.8(15)	7.4(13)	16.7(15)	31.6(17)
C14	80(2)	82(2)	65.9(17)	11.6(14)	34.3(16)	25.7(17)
C15	61.6(19)	60.1(17)	128(3)	10.8(18)	41(2)	16.4(15)
C16	53.9(17)	67.1(18)	98(2)	2.4(16)	18.3(16)	21.4(14)
C17	56.9(15)	55.9(14)	73.1(17)	9.1(12)	20.6(13)	22.5(12)
C18	55.7(14)	46.2(12)	66.8(14)	10.1(10)	26.5(12)	25.2(11)
C19	77.7(19)	73.3(17)	62.6(15)	12.9(13)	16.8(14)	44.1(15)
C20	87(3)	70.1(19)	119(3)	40.3(19)	53(2)	28.1(18)

C21	68.8(19)	64.8(16)	83.7(19)	25.2(14)	30.1(16)	28.0(15)
C22	51.1(14)	40.7(10)	56.3(12)	10.4(9)	21.9(11)	20.7(10)
C23	57.6(15)	56.9(14)	80.9(17)	12.0(12)	26.3(14)	31.0(12)
C24	113(3)	86(2)	159(4)	39(3)	69(3)	71(2)
C25	111(3)	145(4)	72(2)	-4(2)	39(2)	71(3)
C26	69(2)	95(3)	135(3)	26(2)	47(2)	34(2)
C27	94(3)	72.8(18)	55.2(15)	9.2(13)	10.5(15)	35.1(17)

Supplementary Table 5. Bond Lengths for cu_szl_htb_19_81_1_0m_a.

Atom	Atom	Length/Å	Atom	Atom	Length/Å
Si1	O2	1.6588(17)	C3	C4	1.387(4)
Si1	C3	1.864(3)	C3	C7	1.398(4)
Si1	C9	1.875(3)	C4	C5	1.395(5)
Si1	C13	1.860(3)	C5	C6	1.371(7)
Si2	O4	1.6574(18)	C6	C8	1.348(7)
Si2	C18	1.873(3)	C7	C8	1.373(5)
Si2	C23	1.879(3)	C9	C10	1.525(4)
Si2	C27	1.860(3)	C9	C11	1.539(4)
O1	C0AA	1.241(3)	C9	C12	1.537(4)
O2	C2	1.414(3)	C15	C16	1.363(5)
O3	C0AA	1.270(3)	C15	C20	1.373(6)
O4	C1	1.415(3)	C16	C17	1.387(4)
O5	C22	1.256(3)	C17	C18	1.391(4)
O6	C22	1.253(3)	C18	C21	1.388(4)
C1	C19	1.516(4)	C20	C21	1.386(5)
C1	C22	1.517(3)	C23	C24	1.540(5)
C0AA	C2	1.520(3)	C23	C25	1.514(5)
C2	C14	1.524(4)	C23	C26	1.520(5)

Supplementary Table 6. Bond Angles for cu_szl_htb_19_81_1_0m_a.

Atom	Atom	Atom	Angle/˚	Atom	Atom	Atom	Angle/˚
O2	Si1	C3	108.08(11)	C6	C5	C4	119.3(4)

O2	Si1	C9	106.00(11)	C8	C6	C5	120.7(3)
O2	Si1	C13	111.06(13)	C8	C7	C3	121.4(4)
C3	Si1	C9	110.34(12)	C6	C8	C7	120.4(4)
C13	Si1	C3	109.60(16)	C10	C9	Si1	110.7(2)
C13	Si1	C9	111.66(16)	C10	C9	C11	108.7(3)
O4	Si2	C18	107.94(11)	C10	C9	C12	109.2(3)
O4	Si2	C23	106.59(11)	C11	C9	Si1	110.6(2)
O4	Si2	C27	110.69(14)	C12	C9	Si1	108.5(2)
C18	Si2	C23	110.26(12)	C12	C9	C11	109.2(3)
C27	Si2	C18	109.75(15)	C16	C15	C20	119.6(3)
C27	Si2	C23	111.51(17)	C15	C16	C17	120.1(4)
C2	O2	Si1	123.57(16)	C16	C17	C18	121.8(3)
C1	O4	Si2	123.24(14)	C17	C18	Si2	121.0(2)
O4	C1	C19	109.5(2)	C21	C18	Si2	122.2(2)
O4	C1	C22	112.0(2)	C21	C18	C17	116.6(3)
C19	C1	C22	109.2(2)	C15	C20	C21	120.4(3)
O1	C0AA	O3	123.8(2)	C20	C21	C18	121.4(4)
O1	C0AA	C2	120.7(2)	O5	C22	C1	116.9(2)
O3	C0AA	C2	115.5(2)	O6	C22	O5	123.5(2)
O2	C2	C0AA	111.61(19)	O6	C22	C1	119.7(2)
O2	C2	C14	108.7(2)	C24	C23	Si2	107.2(2)
C0AA	C2	C14	109.5(2)	C25	C23	Si2	110.9(2)
C4	C3	Si1	121.0(2)	C25	C23	C24	110.4(3)
C4	C3	C7	117.0(3)	C25	C23	C26	109.8(3)
C7	C3	Si1	122.0(2)	C26	C23	Si2	111.1(2)
C3	C4	C5	121.1(4)	C26	C23	C24	107.2(3)

Supplementary Table 7. Torsion Angles for cu_szl_htb_19_81_1_0m_a.

A	B	C	D	Angle/°	A	B	C	D	Angle/°
Si1	O2	C2	C0AA	-91.0(2)	C7	C3	C4	C5	1.4(5)
Si1	O2	C2	C14	148.1(2)	C9	Si1	O2	C2	-172.97(17)
Si1	C3	C4	C5	-175.5(3)	C9	Si1	C3	C4	80.9(3)

Si1	C3	C7	C8	175.0(3)	C9	Si1	C3	C7	-95.8(3)
Si2	O4	C1	C19	147.00(19)	C13	Si1	O2	C2	65.6(2)
Si2	O4	C1	C22	-91.8(2)	C13	Si1	C3	C4	-155.7(3)
Si2	C18	C21	C20	176.3(3)	C13	Si1	C3	C7	27.6(3)
O1	C0AA	C2	O2	-9.6(3)	C13	Si1	C9	C10	60.1(3)
O1	C0AA	C2	C14	110.9(3)	C13	Si1	C9	C11	-179.4(2)
O2	Si1	C3	C4	-34.5(3)	C13	Si1	C9	C12	-59.7(3)
O2	Si1	C3	C7	148.7(2)	C15	C16	C17	C18	-0.4(5)
O2	Si1	C9	C10	-61.0(2)	C15	C20	C21	C18	0.4(6)
O2	Si1	C9	C11	59.5(2)	C16	C15	C20	C21	-0.1(6)
O2	Si1	C9	C12	179.2(2)	C16	C17	C18	Si2	-176.4(2)
O3	C0AA	C2	O2	170.8(2)	C16	C17	C18	C21	0.6(4)
O3	C0AA	C2	C14	-68.7(3)	C17	C18	C21	C20	-0.6(5)
O4	Si2	C18	C17	-36.6(2)	C18	Si2	O4	C1	-56.0(2)
O4	Si2	C18	C21	146.6(2)	C18	Si2	C23	C24	62.9(3)
O4	Si2	C23	C24	179.8(3)	C18	Si2	C23	C25	-57.7(3)
O4	Si2	C23	C25	59.3(3)	C18	Si2	C23	C26	179.9(3)
O4	Si2	C23	C26	-63.2(3)	C19	C1	C22	O5	-68.2(3)
O4	C1	C22	O5	170.5(2)	C19	C1	C22	O6	110.9(3)
O4	C1	C22	O6	-10.5(3)	C20	C15	C16	C17	0.1(5)
C3	Si1	O2	C2	-54.7(2)	C23	Si2	O4	C1	-174.44(19)
C3	Si1	C9	C10	-177.8(2)	C23	Si2	C18	C17	79.4(3)
C3	Si1	C9	C11	-57.3(3)	C23	Si2	C18	C21	-97.4(3)
C3	Si1	C9	C12	62.4(3)	C27	Si2	O4	C1	64.1(2)
C3	C4	C5	C6	-0.4(6)	C27	Si2	C18	C17	-157.4(2)
C3	C7	C8	C6	1.3(7)	C27	Si2	C18	C21	25.8(3)
C4	C3	C7	C8	-1.8(5)	C27	Si2	C23	C24	-59.3(3)
C4	C5	C6	C8	-0.1(6)	C27	Si2	C23	C25	-179.8(3)
C5	C6	C8	C7	-0.3(7)	C27	Si2	C23	C26	57.7(3)

Supplementary Table 8. Hydrogen Atom Coordinates (Å×10^4) and Isotropic Displacement Parameters (Å^2×10^3) for cu_szl_htb_19_81_1_0m_a.

**Atom**	***x***	***y***	***z***	**U(eq)**
H1	9656	9279	7011	60
H2	3975	717	2779	61
H4	1292	-2072	1025	84
H5	1604	-4417	560	111
H6	1375	-6152	1851	126
H7	568	-3270	4064	96
H8	843	-5592	3572	122
H10A	-3652	-105	1982	120
H10B	-1612	1128	2041	120
H10C	-2079	636	3190	120
H11A	-3713	-2023	412	128
H11B	-2457	-2858	645	128
H11C	-1613	-999	515	128
H12A	-2992	-2303	3453	133
H12B	-3130	-3614	2446	133
H12C	-4563	-2963	2250	133
H13A	2319	487	4888	114
H13B	317	-828	4843	114
H13C	752	977	4692	114
H14A	3609	1163	875	119
H14B	5509	2628	1695	119
H14C	3803	2915	1308	119
H15	14513	16146	8046	105
H16	13839	14425	9337	94
H17	10944	12137	8901	77
H19A	10303	8852	8900	104
H19B	10129	7364	8030	104
H19C	8498	7112	8531	104
H20	12274	15586	6316	109
H21	9372	13302	5871	87
H24A	5329	12353	6555	157

H24B	7129	13664	7553	157
H24C	5198	13024	7765	157
H25A	6436	12206	9578	158
H25B	8324	12766	9319	158
H25C	7183	10940	9467	158
H26A	4214	8976	7995	149
H26B	3572	9424	6805	149
H26C	3457	10246	7970	149
H27A	6959	9742	5029	118
H27B	6007	10836	5156	118
H27C	5046	8974	5276	118
H3	7040(100)	4410(70)	4660(40)	180(30)
H6A	5280(110)	5410(60)	5330(50)	200(30)

2. Supplementary Discussion

2.1 Optimization of Reaction Conditions

Supplementary Table 9. Screening of Chiral Secondary Alcohol **2**[a]

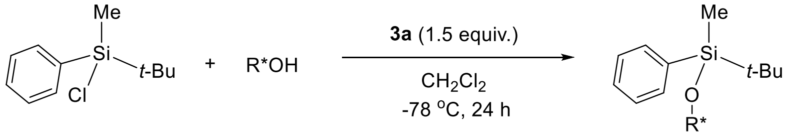

1a **2** **4**

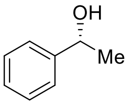

93%,[b] *dr* = 50:50[c]

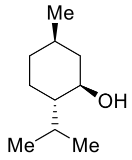

96%, *dr* = 50:50

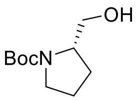

80%, *dr* = 50:50

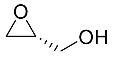

80%, *dr* = 50:50

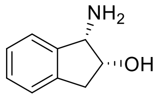

45%, *dr* = 50:50

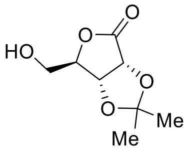

97%, *dr* = 50:50

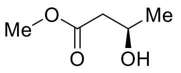

90%, *dr* = 60:40

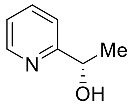

79%, *dr* = 67:33

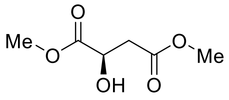

80%, *dr* = 84:16

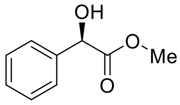

98%, *dr* = 89:11

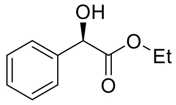

88%, *dr* = 89:11

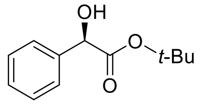

45%, *dr* = 84:16

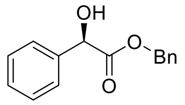

84%, *dr* = 89:11

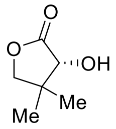

87%, *dr* = 86:14

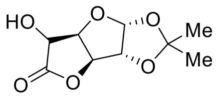

79%, *dr* = 78:22

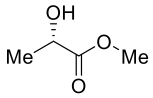

94%, *dr* = 90:10

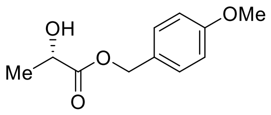

95%, *dr* = 92:8

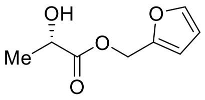

93%, *dr* = 91:9

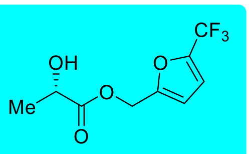

95%, *dr* = 92:8

[a]Reaction condition: **2** (0.1 mmol), **1a** (0.12 mmol) and **3a** (0.15 mmol) in CH_2Cl_2 (1 mL) at -78 °C for 24 h. [b]Isolated yield. [c]Determined by ^1H NMR.

Supplementary Table 10. Screening of Base[a]

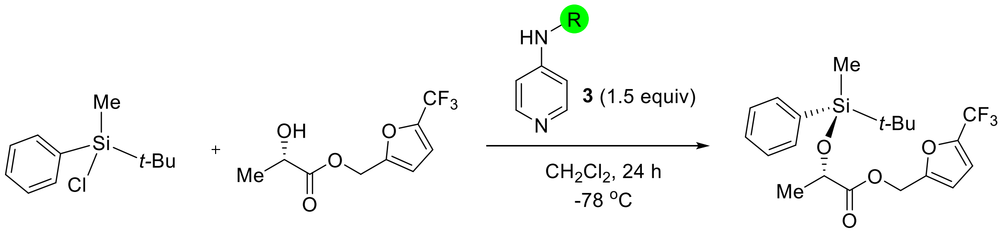

1a **2i** **4i**

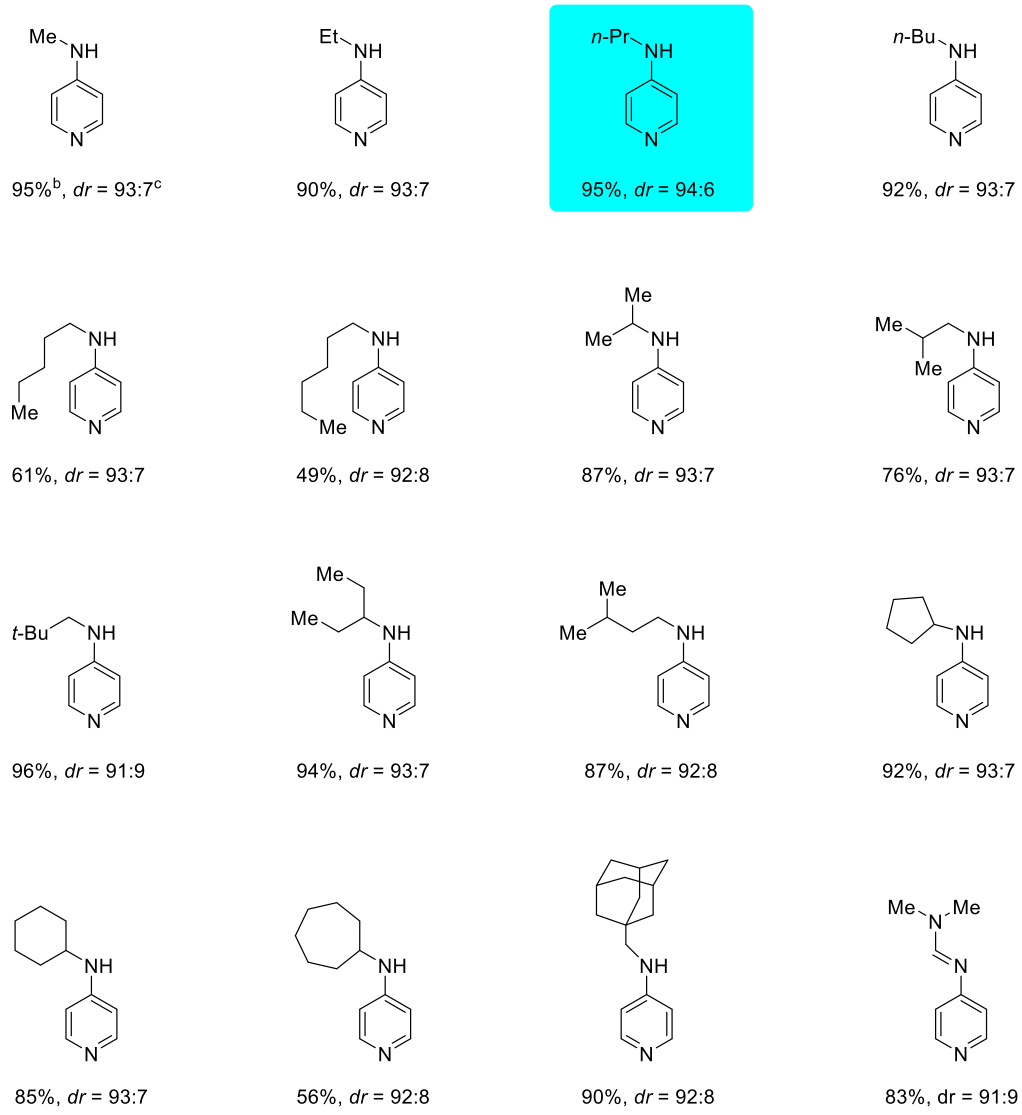

[a]Reaction condition: **2i** (0.1 mmol), **1a** (0.12 mmol) and **3** (0.15 mmol) in CH_2Cl_2 (1 mL) at -78 °C for 24 h. [b]solated yield. [c]Determined by ^{1}H NMR.

2.2 ^{29}Si NMR Studies

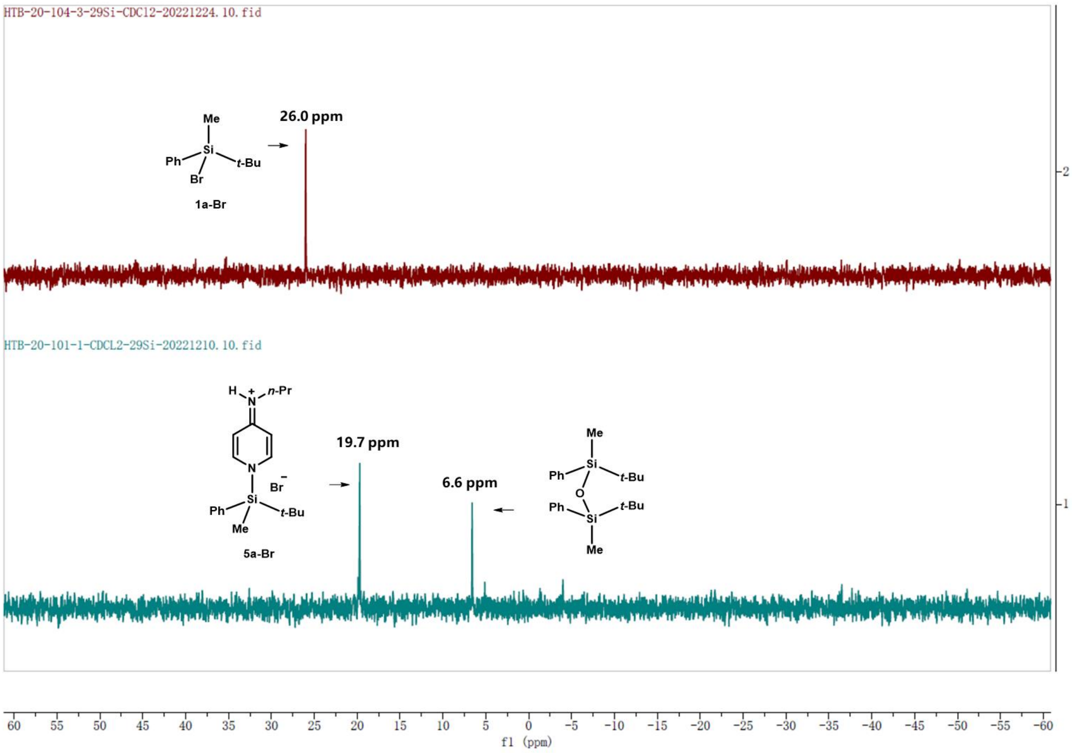

Supplementary Figure 6. ^{29}Si NMR spectra of **1a-Br** (A) and **1a-Br/3e** (1:1, B) in CD_2Cl_2 at 25 °C for 6 h.

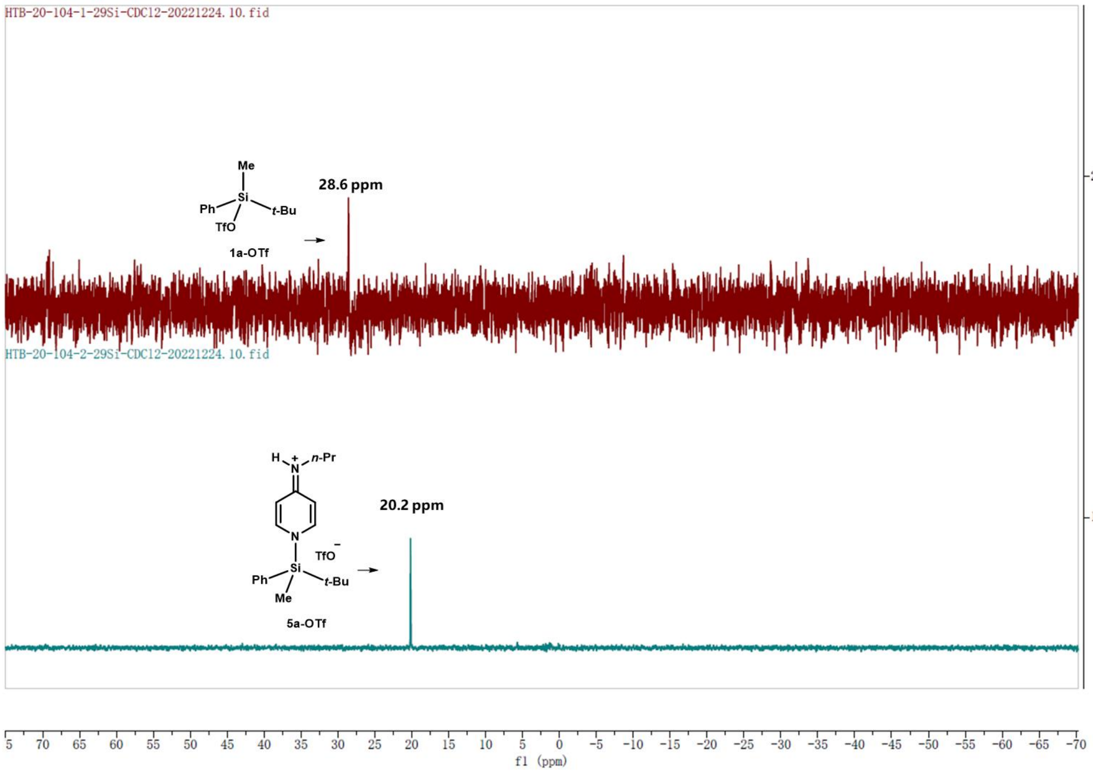

Supplementary Figure 7. ^{29}Si NMR spectra of 1a-OTf (A) and **1a-OTf /3e** (1:1, B) in CD_2Cl_2 at 25 °C

for 6 h.

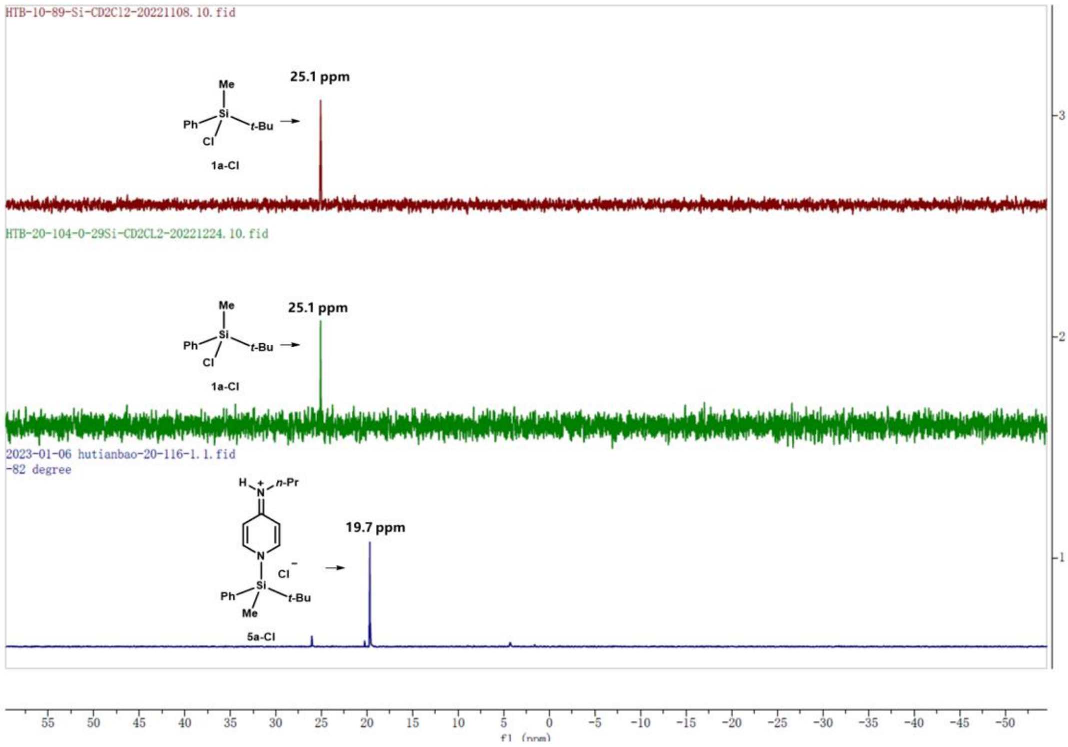

Supplementary Figure 8. ^{29}Si NMR spectra of **1a-Cl** (A) and **1a-Cl/3e** (1:1, B) in CD_2Cl_2 at 25 °C for 6 h, and of **1a-Cl/3e** (1:1, C) at -78 °C for 10 min.

2.3 DFT studies

To understand the reaction mechanism, DFT calculations at the M062X-D3/6-311G(d, p) (SMD, CH_2Cl_2) level of theory have been performed by using Gaussian 09 program[18]. The O-Si bonds in **Si^S-4g** and **Si^R-4g** were formed through S_N2 nucleophilic substitution, accompanying with the deprotonation of **2g** by **NEt_3** (see **Figure 9**). For **Si^S/C^S-TS2** and **Si^R/C^S-TS2**, the O atoms of the ester group in **2g** coordinate to the Si atoms, with the O…Si distances of 2.02 and 2.00 Å, respectively. In contrast to **Si^S/C^S-TS2**, the steric repulsion between the Ph group and **2g** was more significant in **Si^R/C^S-TS2** (see **Figure 10**). Accordingly, the **Si^R/C^S-TS2** was less stable than **Si^S/C^S-TS2** by 5.4 kcal mol^{-1} at 195K. The theoretical selectivity was predicated to be >99%, which was close to experimental result (86%). Note that chiral silicon atom in **Si^R-IM1** could transform into **Si^S-IM1** by configuration inversion with low energy barrier of 3.3 kcal mol^{-1}, assisted by one more catalyst **3e**. As a result, **Si^S-4g** was obtained predominantly, with high yield of 95%.

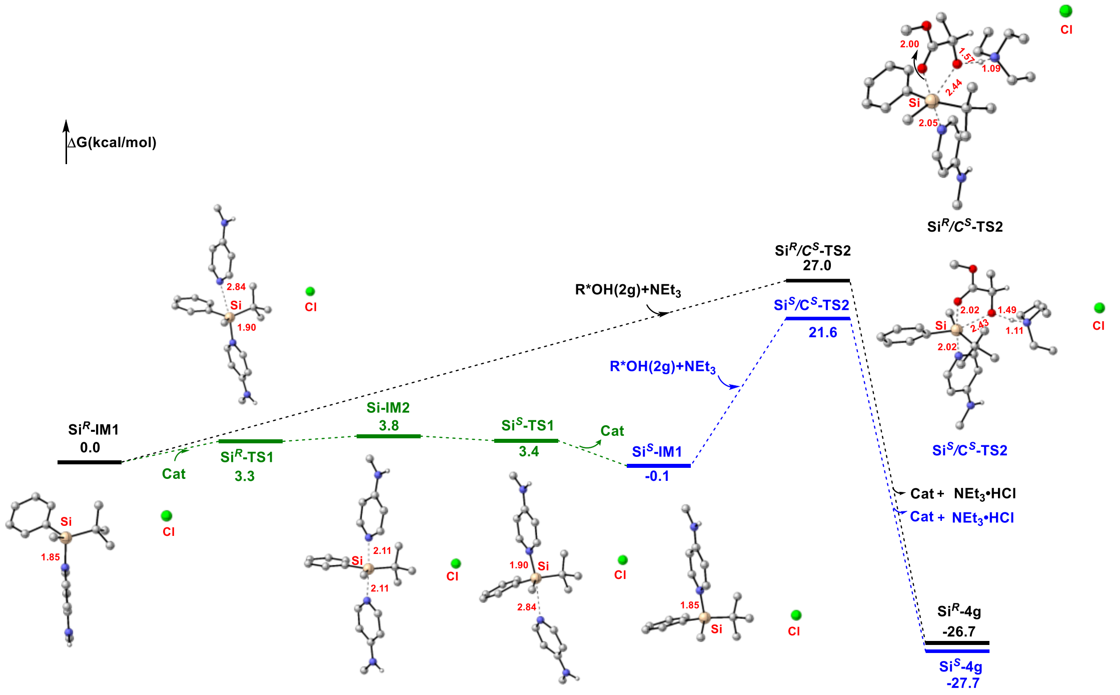

Supplementary Figure 9. Energy profiles associated with the formation of **Si^S-4g** and **Si^R-4g** catalyzed by Cat (**3e**) in the presence of **NEt$_3$**.

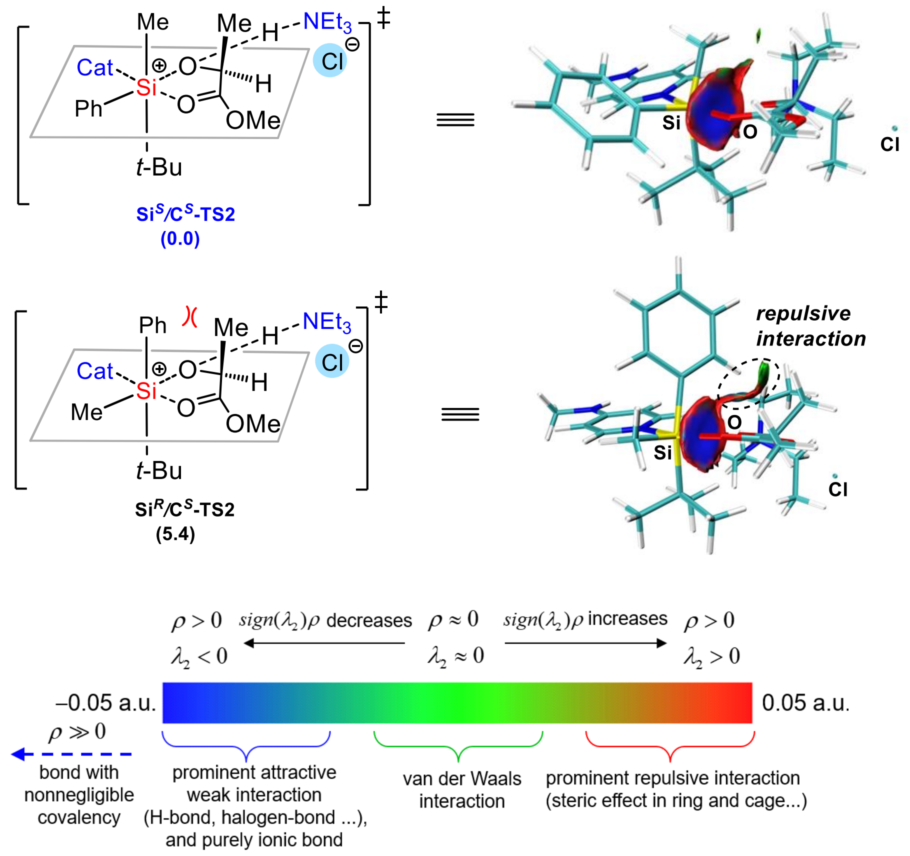

Supplementary Figure 10. Non-covalent interaction analysis for transition states **Si^S/C^S-TS2** and **Si^R/C^S-TS2**, visualized by Multiwfn software[24].

Computational details

All calculations have been performed by using Gaussian 09[18]. Geometries were optimized using the M062X-D3[19, 20] functional and 6-311G(d,p) basis set. The solvent effect of CH_2Cl_2 was evaluated using the self-consistent reaction field (SCRF) method and SMD[21] model. The intrinsic reaction coordinate (IRC)[22] was used to examine the transition state (TS) associated with the corresponding minimum on the potential energy surfaces. The optimized molecular structures were visualized by CYLview (1.0 version) software.[23] The weak interaction of transition states **Si^S/C^S-TS2** and **Si^R/C^S-TS2** were analyzed by Multiwfn software[24].

Supplementary Table 11. The electronic energies (E_Z), enthalpies (H), and Gibbs free energies (G) for all stationary points (in Hartree), obtained at the M062X-D3/6-311G(d,p) (SMD, CH_2Cl_2) theoretical level.

Structures	^aZPE	^bH_c	^cG_c	E_{ZPE}	H	G
**Cat (3e)**	0.13427	0.13838	0.11574	-342.76939	-342.76528	-342.78792
**2g**	0.12395	0.12879	0.10467	-382.74907	-382.74423	-382.76835
**NEt_3**	0.20659	0.21176	0.18718	-292.14141	-292.13624	-292.16082
**NEt_3·HCl**	0.22239	0.22876	0.20058	-752.98384	-752.97747	-753.00565
**Si^S-IM1**	0.39076	0.40294	0.36028	-1521.62426	-1521.61208	-1521.65474
**Si^S-TS1**	0.52666	0.54266	0.49066	-1864.40120	-1864.38520	-1864.43719
**Si^S/C^S-TS2**	0.72783	0.74775	0.69037	-2196.51185	-2196.49193	-2196.54931
**Si^R-IM1**	0.39056	0.40255	0.36103	-1521.62508	-1521.61309	-1521.65462
**Si^R-TS1**	0.52666	0.54266	0.49065	-1864.40120	-1864.38520	-1864.43721
**Si-IM2**	0.52843	0.54417	0.49449	-1864.40250	-1864.38677	-1864.43644
**Si^R/C^S-TS2**	0.72854	0.74851	0.69060	-2196.50290	-2196.48292	-2196.54083
**Si^S-4g**	0.36524	0.37653	0.33708	-1100.80618	-1100.79490	-1100.83435
**Si^R-4g**	0.36663	0.37752	0.33940	-1100.80550	-1100.79460	-1100.83272

a Zero-point correction energy;

b Thermal correction to enthalpy;

c Thermal correction to Gibbs free energy.

3. Supplementary Figures 11-210

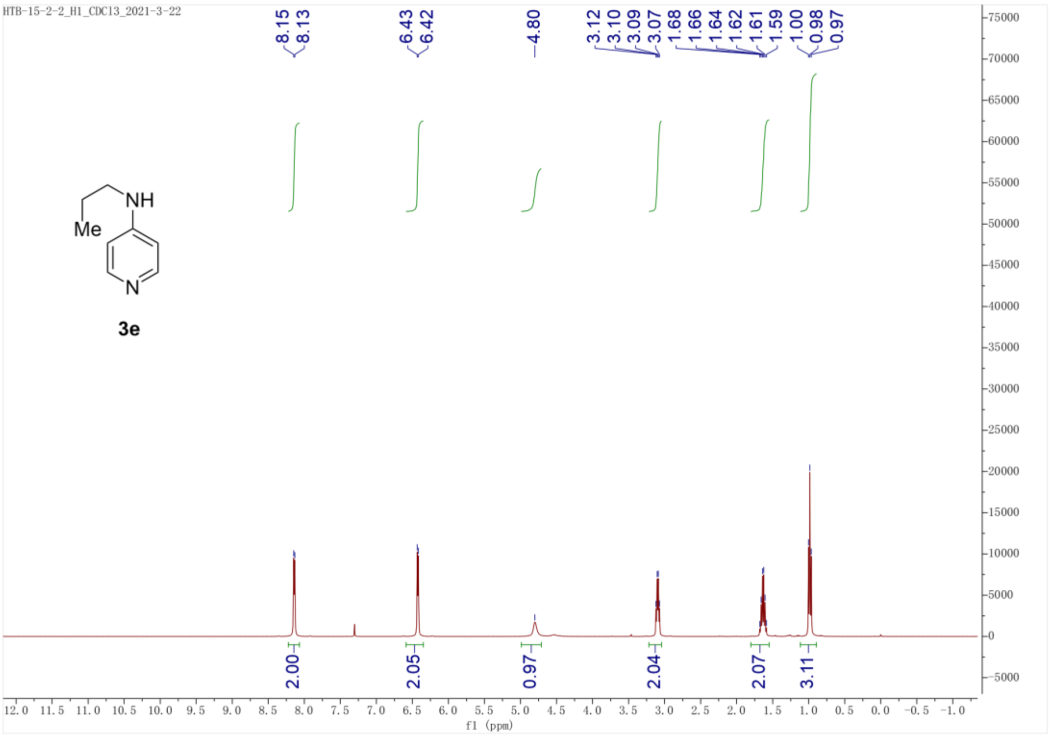

Supplementary Figure 11. ^{1}H NMR (400 M, $CDCl_3$, 25 °C) of compound **3e**

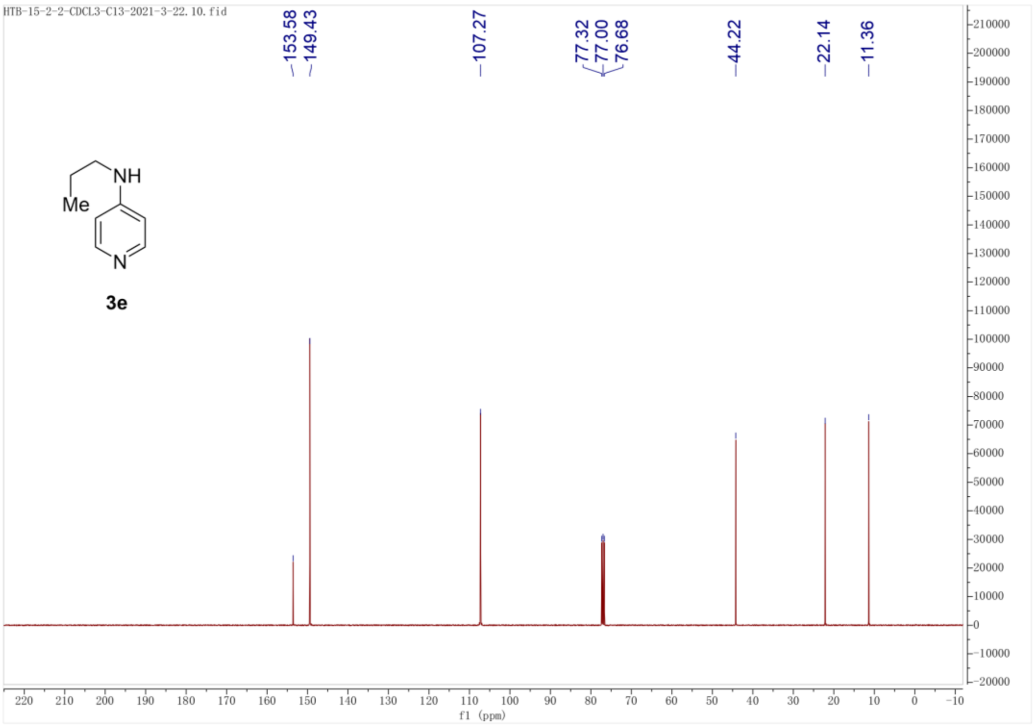

Supplementary Figure 12. ^{13}C NMR (100 M, $CDCl_3$, 25 °C) of compound **3e**

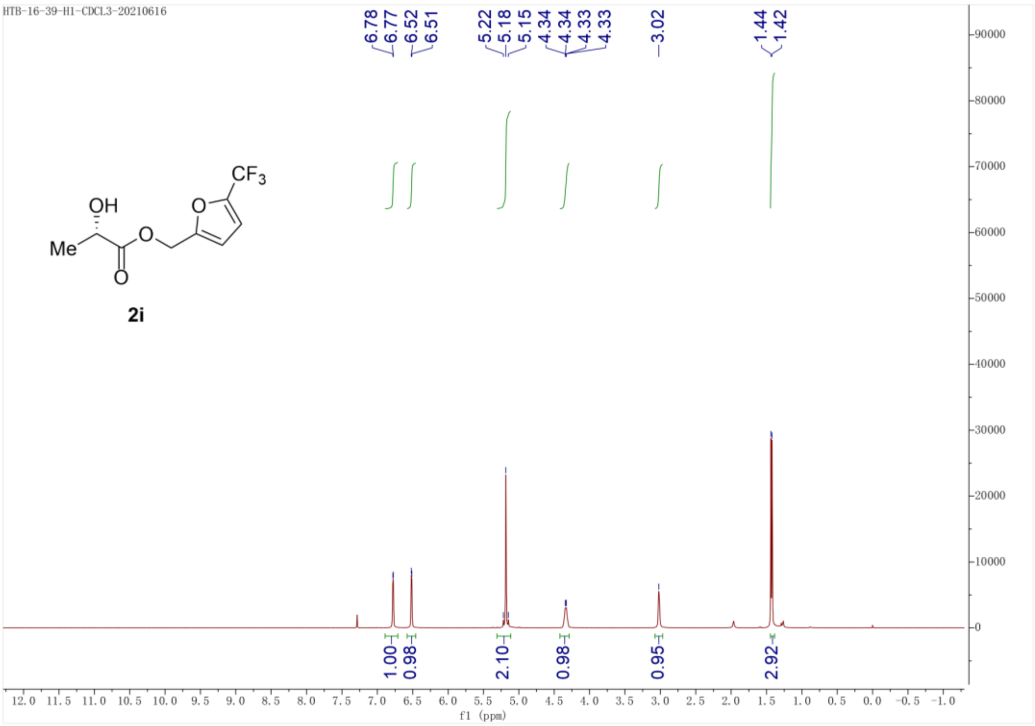

Supplementary Figure 13. ^{1}H NMR (400 M, $CDCl_3$, 25 °C) of compound **2i**

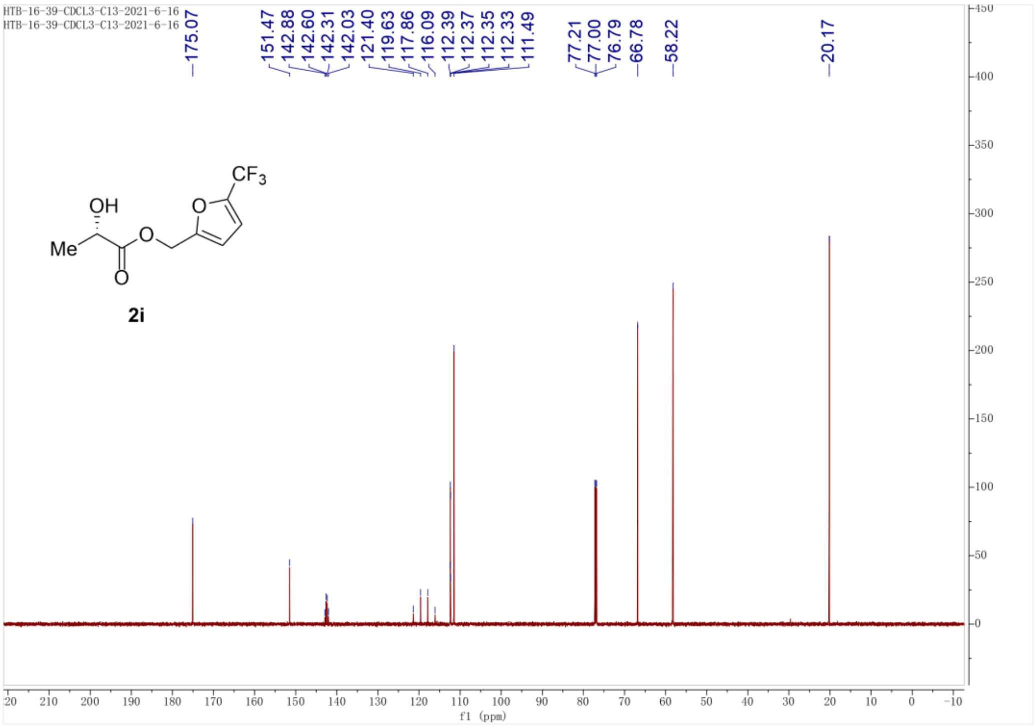

Supplementary Figure 14. ^{13}C NMR (150 M, $CDCl_3$, 25 °C) of compound **2i**

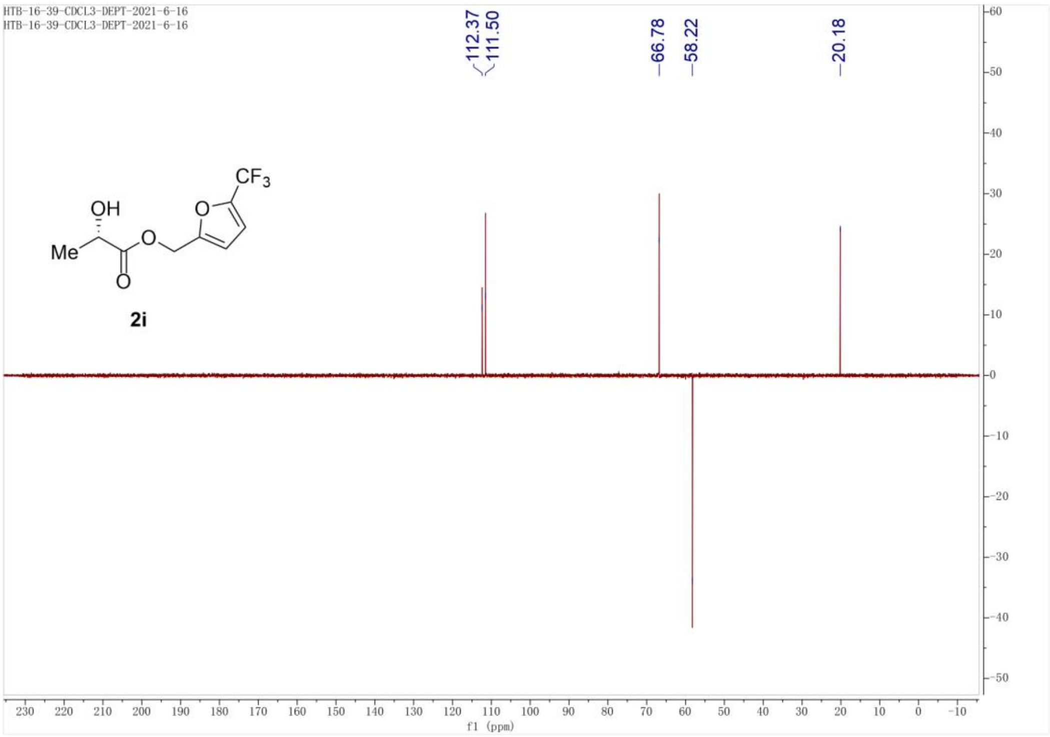

Supplementary Figure 15. DEPT NMR (150 M, $CDCl_3$, 25 °C) of compound **2i**

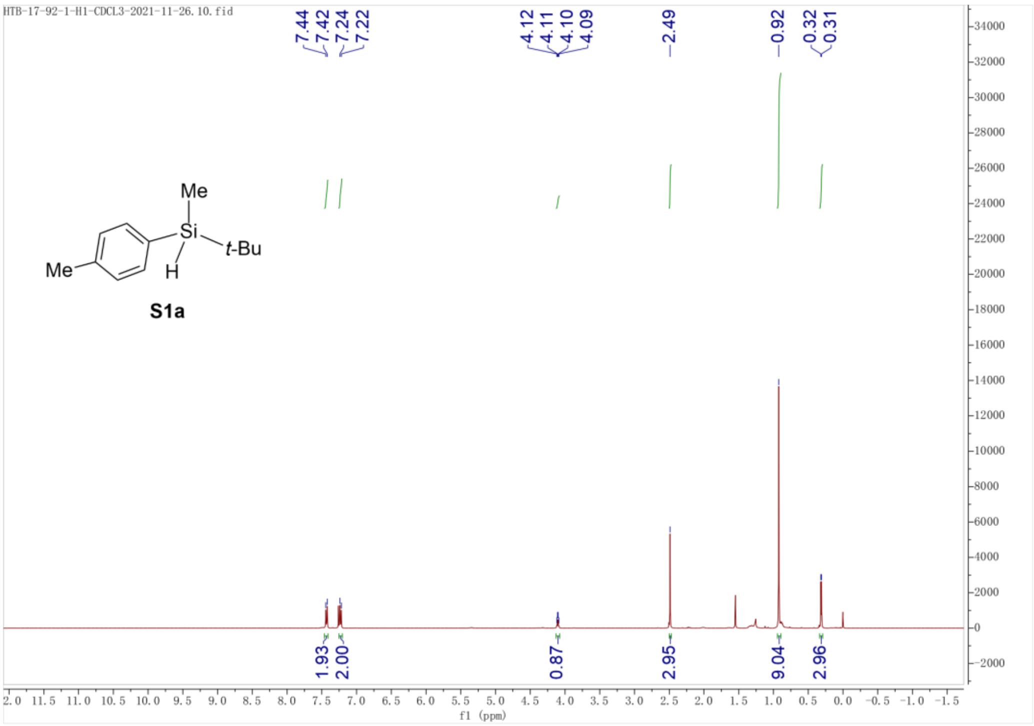

Supplementary Figure 16. ^{1}H NMR (400 M, $CDCl_3$, 25 °C) of compound **S1a**

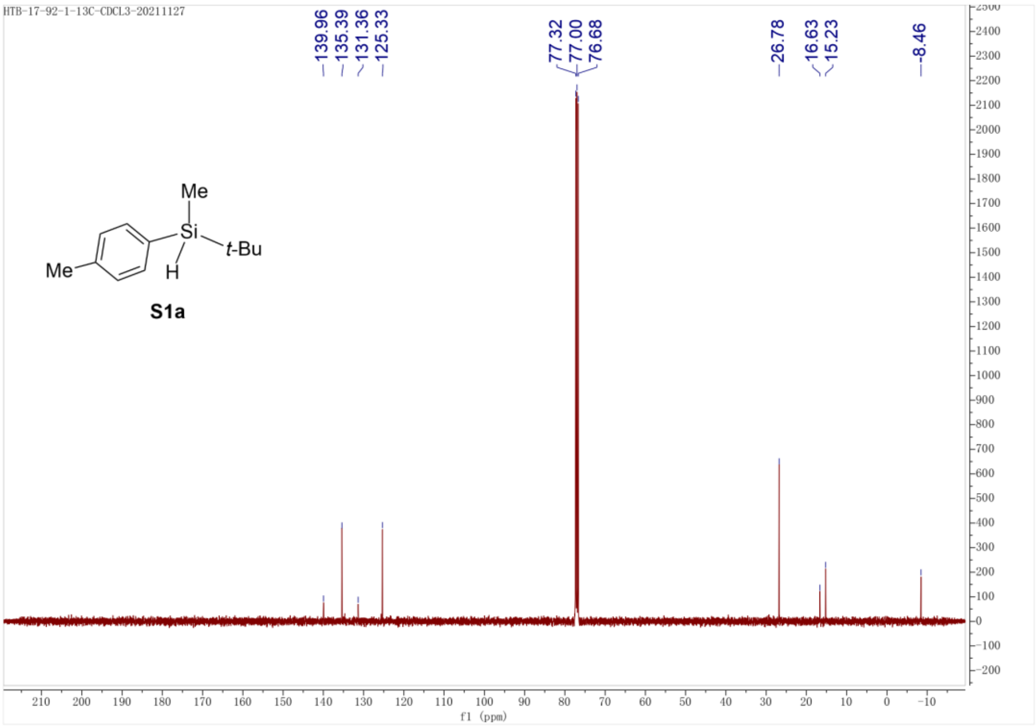

Supplementary Figure 17. ^{13}C NMR (100 M, $CDCl_3$, 25 °C) of compound **S1a**

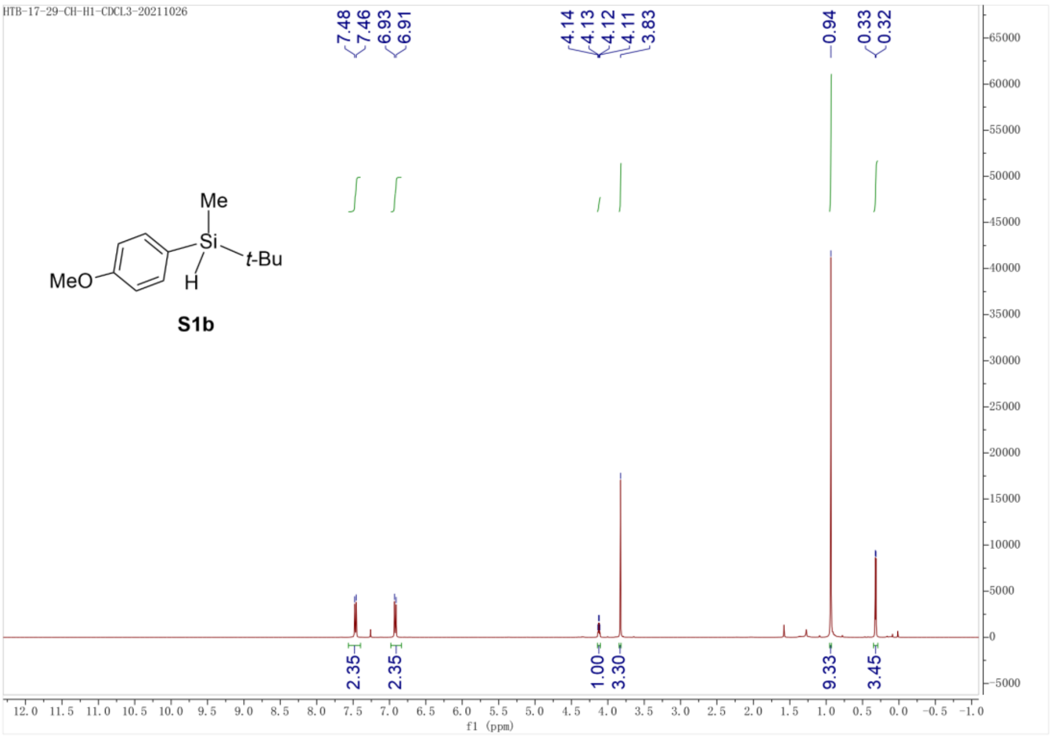

Supplementary Figure 18. ^{1}H NMR (400 M, $CDCl_3$, 25 °C) of compound **S1b**

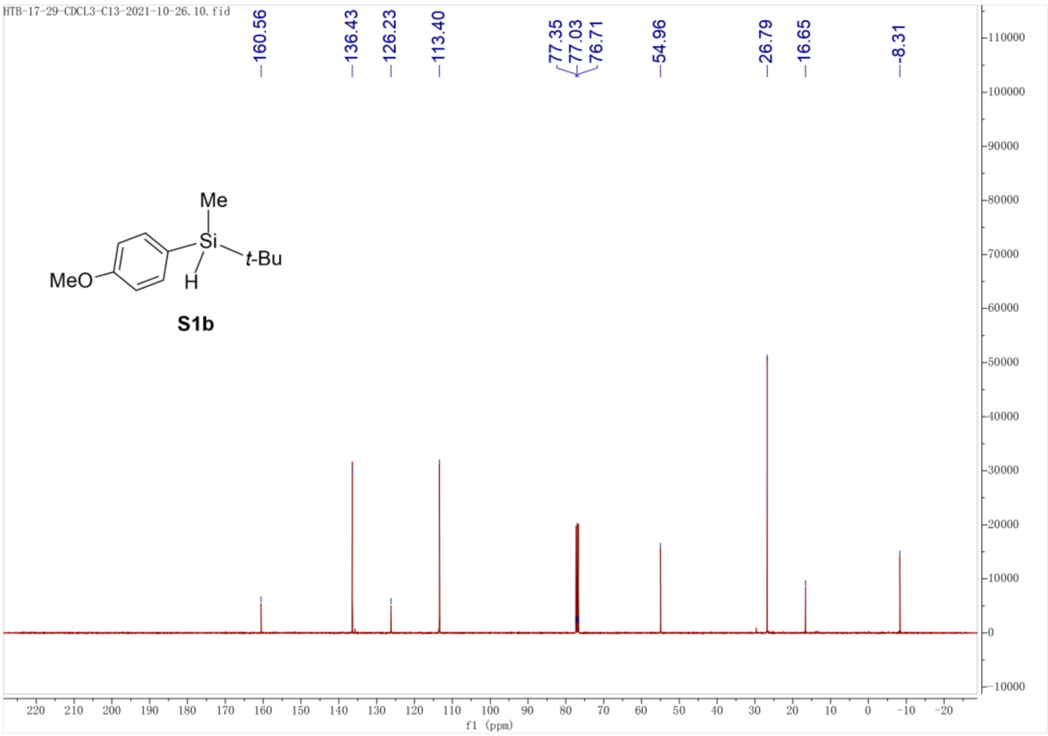

Supplementary Figure 19. ^{13}C NMR (100 M, $CDCl_3$, 25 °C) of compound **S1b**

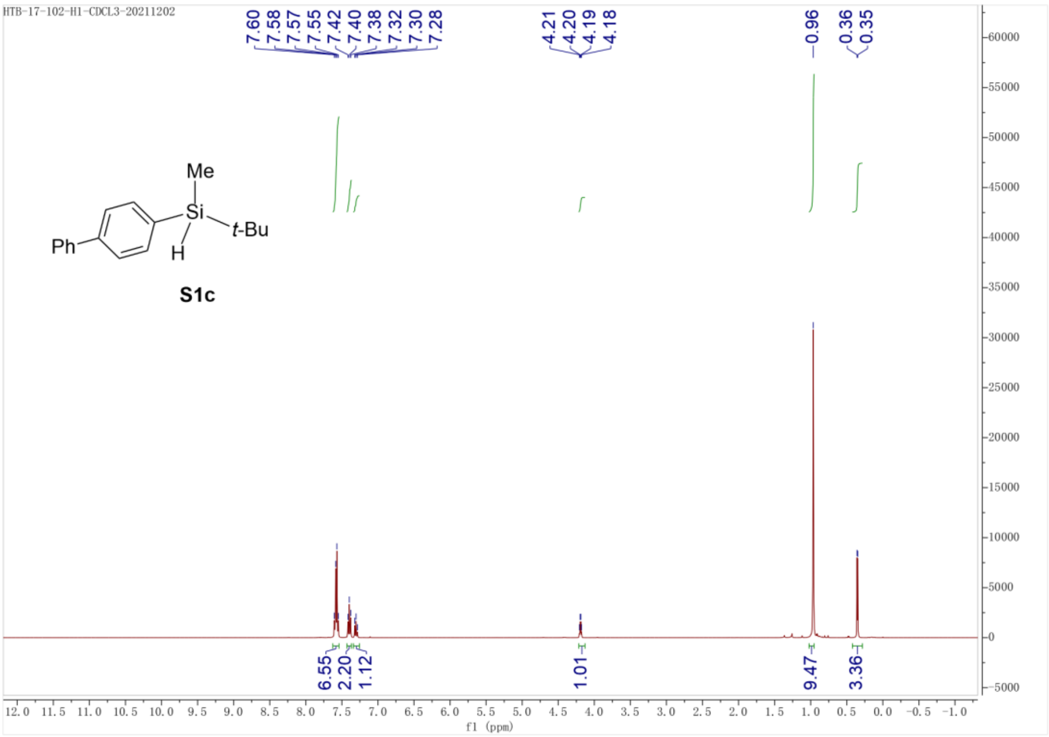

Supplementary Figure 20. ^{1}H NMR (400 M, $CDCl_3$, 25 °C) of compound **S1c**

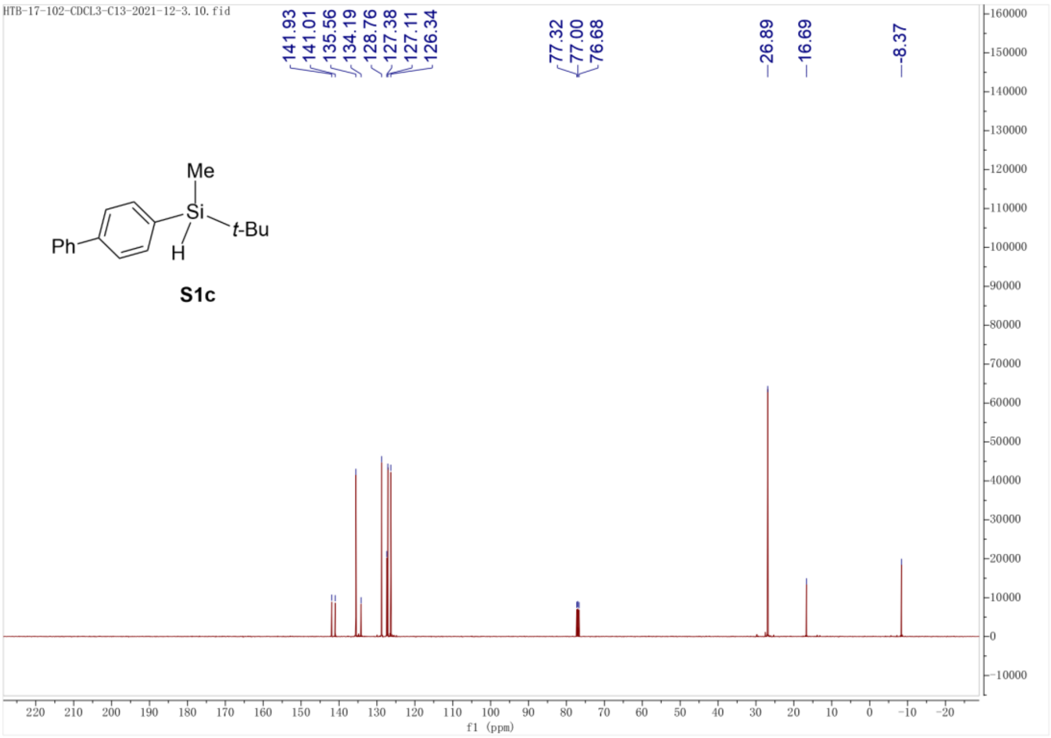

Supplementary Figure 21. ^{13}C NMR (100 M, $CDCl_3$, 25 °C) of compound **S1c**

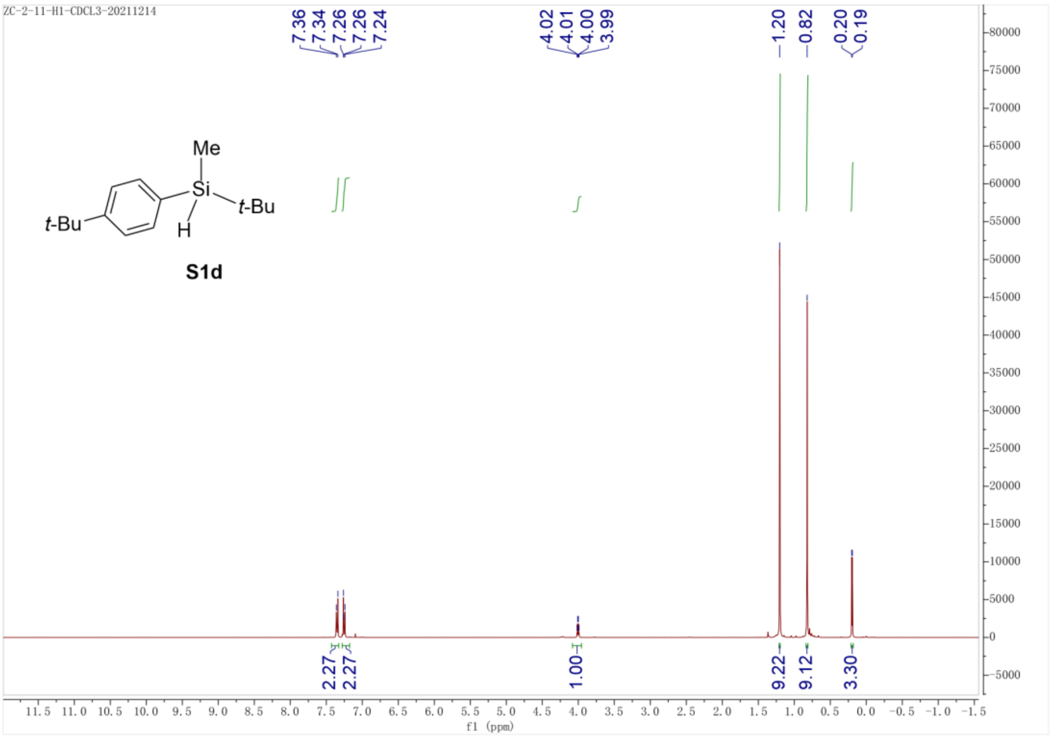

Supplementary Figure 22. ^{1}H NMR (400 M, $CDCl_3$, 25 °C) of compound **S1d**

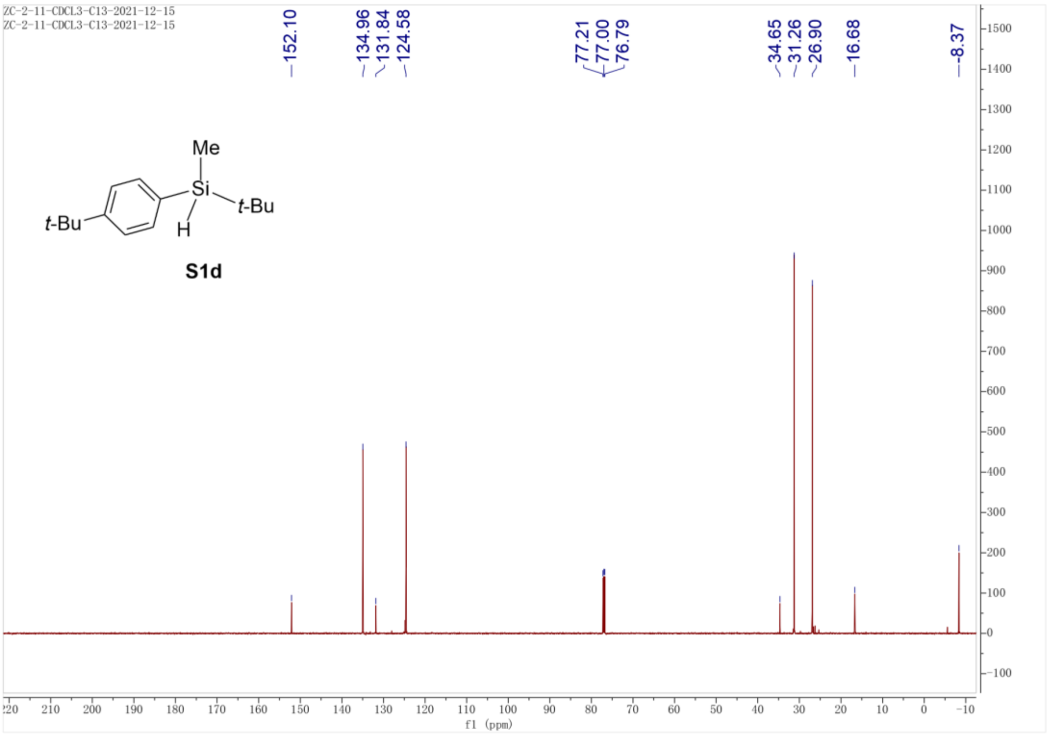

Supplementary Figure 23. ^{13}C NMR (150 M, $CDCl_3$, 25 ^oC) of compound **S1d**

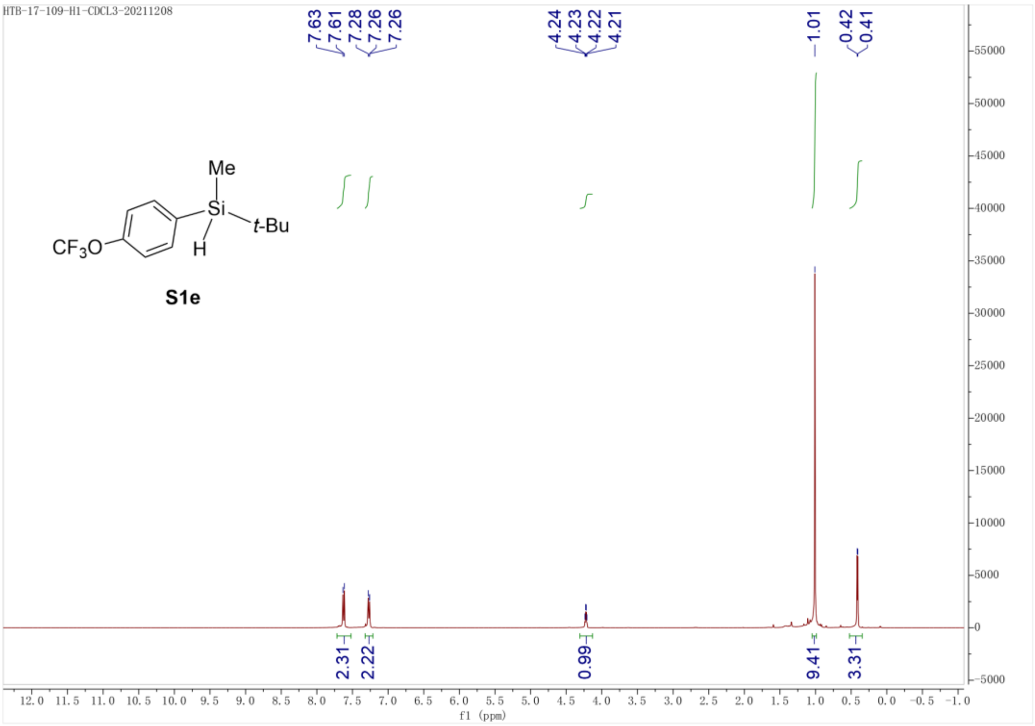

Supplementary Figure 24. ^{1}H NMR (400 M, $CDCl_3$, 25 ^oC) of compound **S1e**

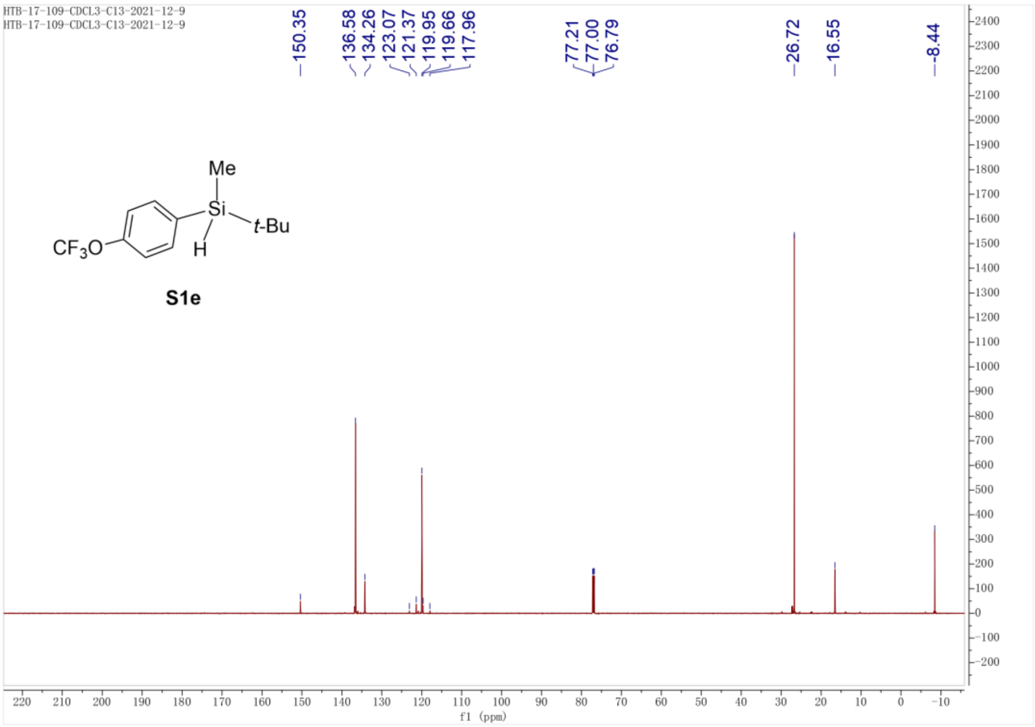

Supplementary Figure 25. ^{13}C NMR (150 M, $CDCl_3$, 25 °C) of compound **S1e**

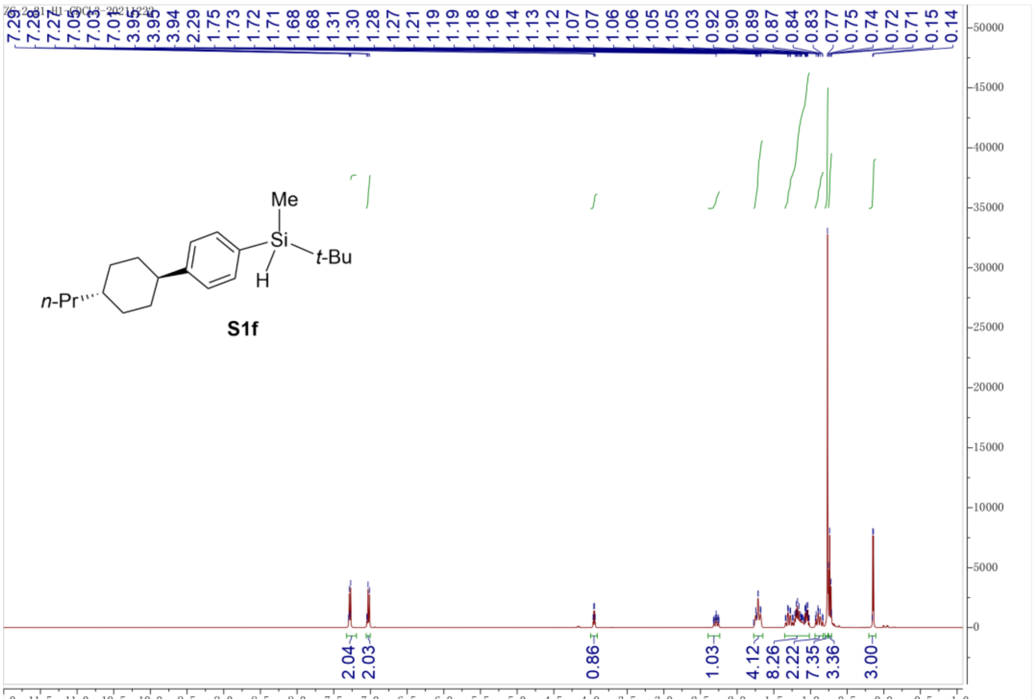

Supplementary Figure 26. ^{1}H NMR (400 M, $CDCl_3$, 25 °C) of compound **S1f**

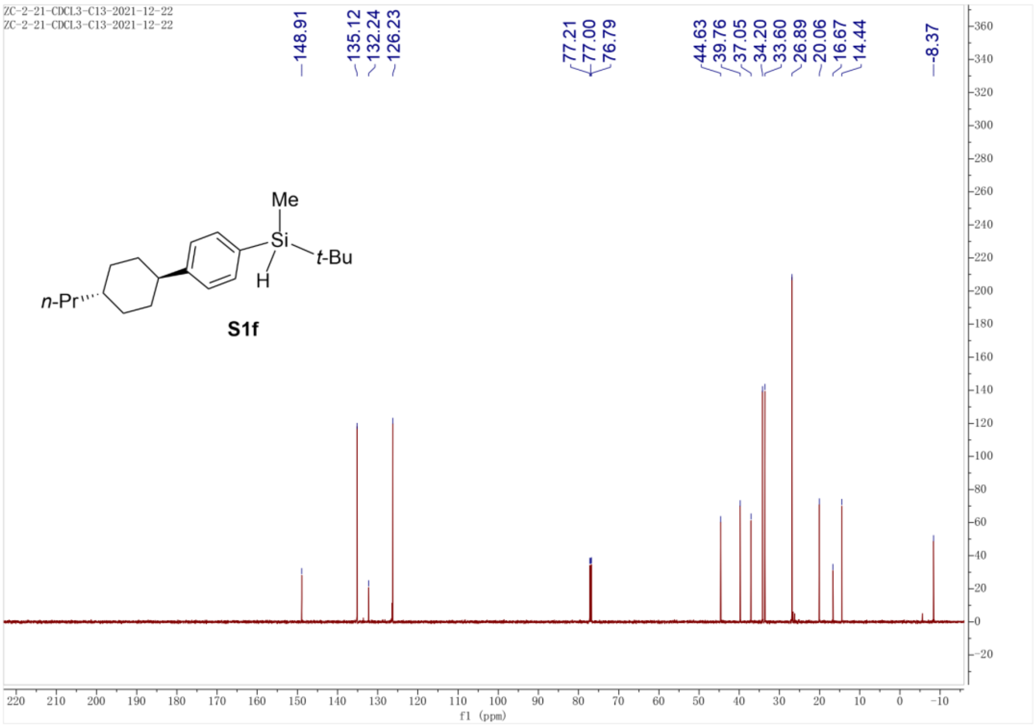

Supplementary Figure 27. ^{13}C NMR (100 M, $CDCl_3$, 25 °C) of compound **S1f**

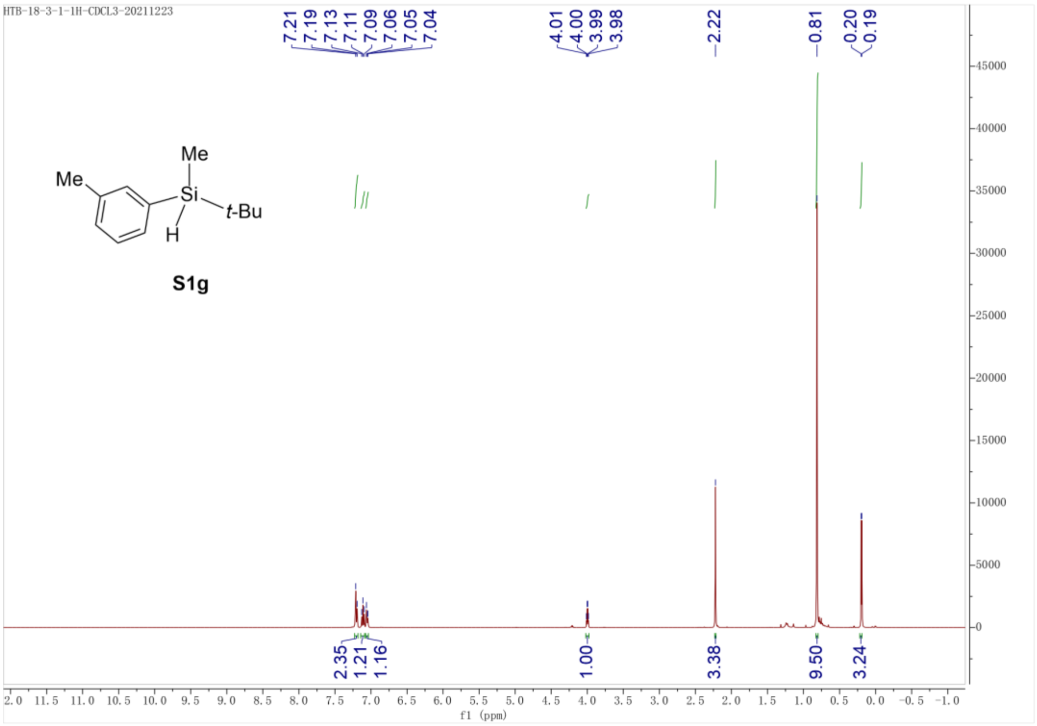

Supplementary Figure 28. ^{1}H NMR (400 M, $CDCl_3$, 25 °C) of compound **S1g**

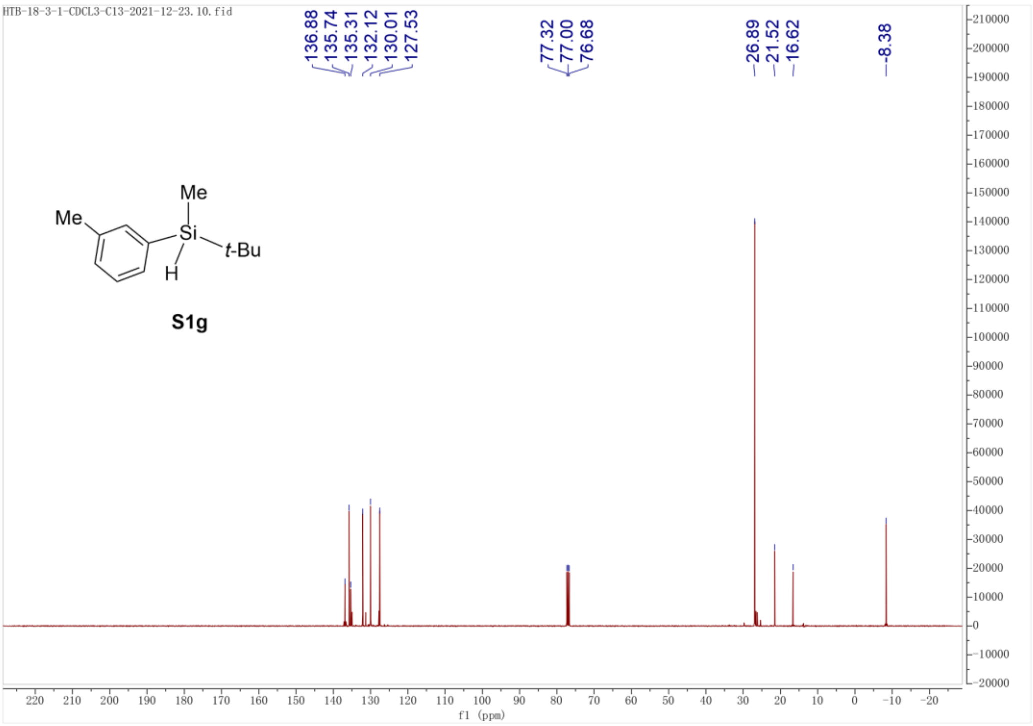

Supplementary Figure 29. ^{13}C NMR (100 M, $CDCl_3$, 25 °C) of compound **S1g**

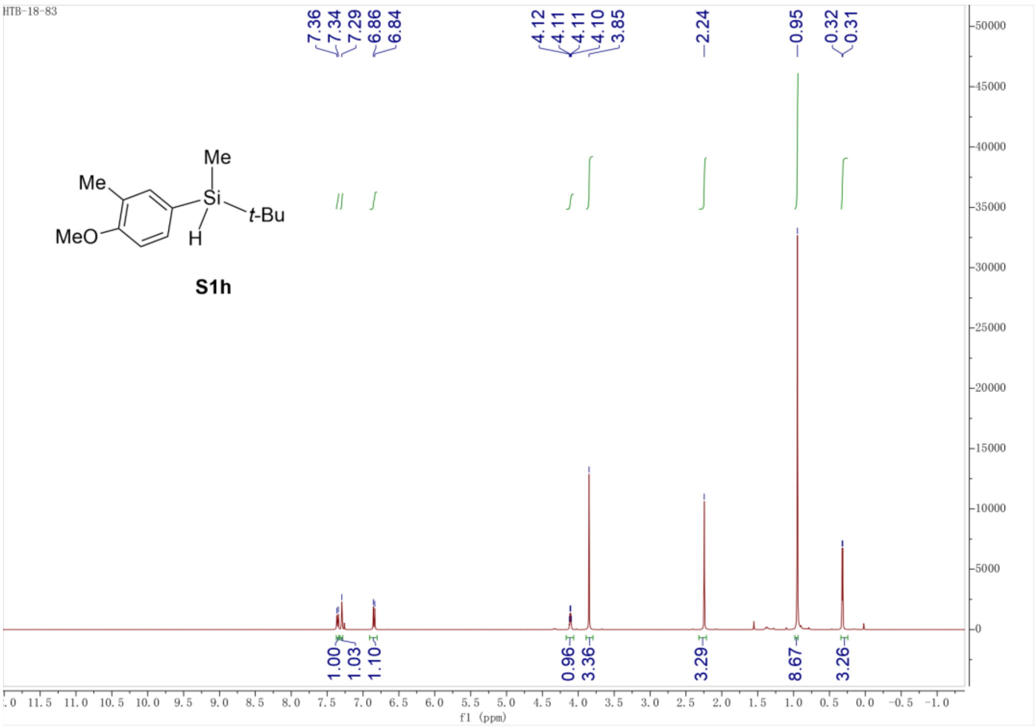

Supplementary Figure 30. ^{1}H NMR (400 M, $CDCl_3$, 25 °C) of compound **S1h**

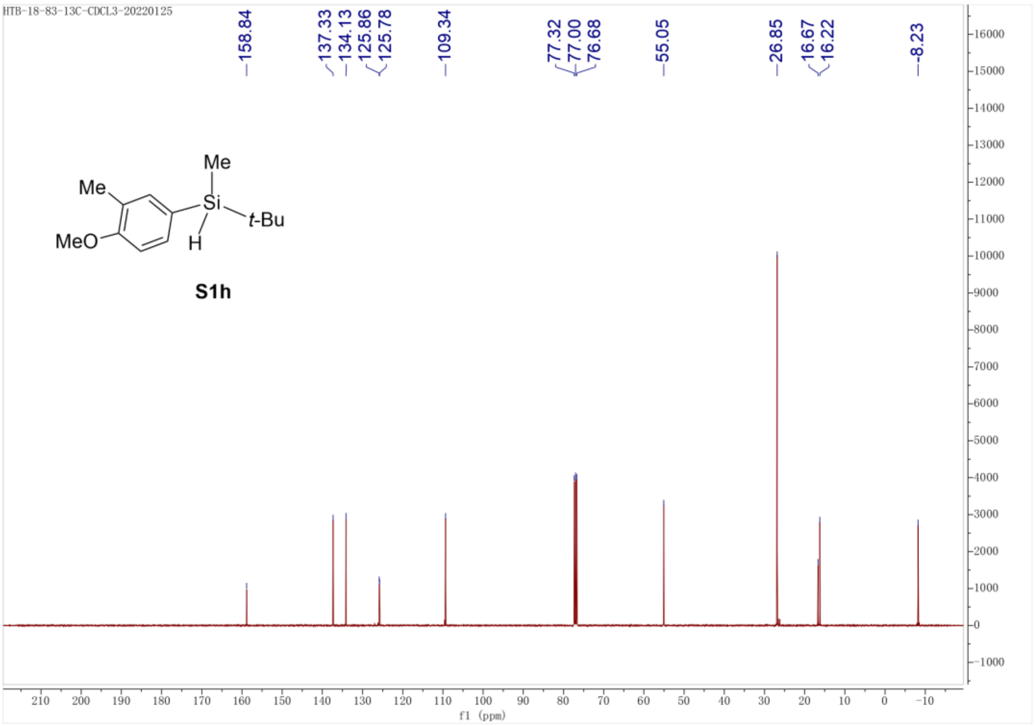

Supplementary Figure 31. ^{13}C NMR (100 M, $CDCl_3$, 25 °C) of compound **S1h**

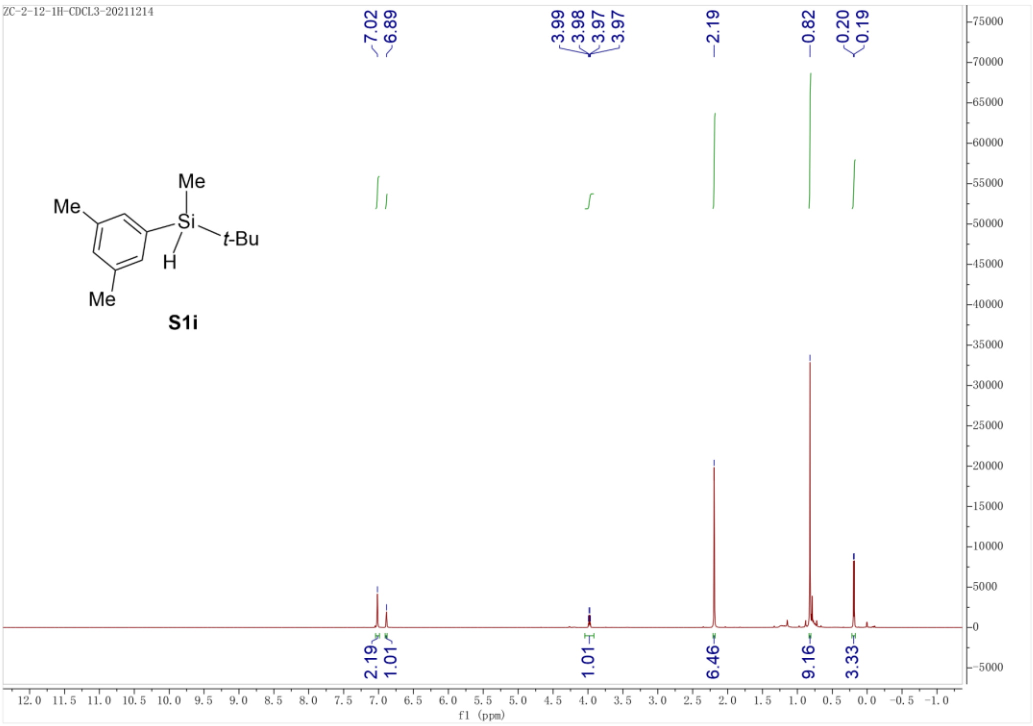

Supplementary Figure 32. ^{1}H NMR (400 M, $CDCl_3$, 25 °C) of compound **S1i**

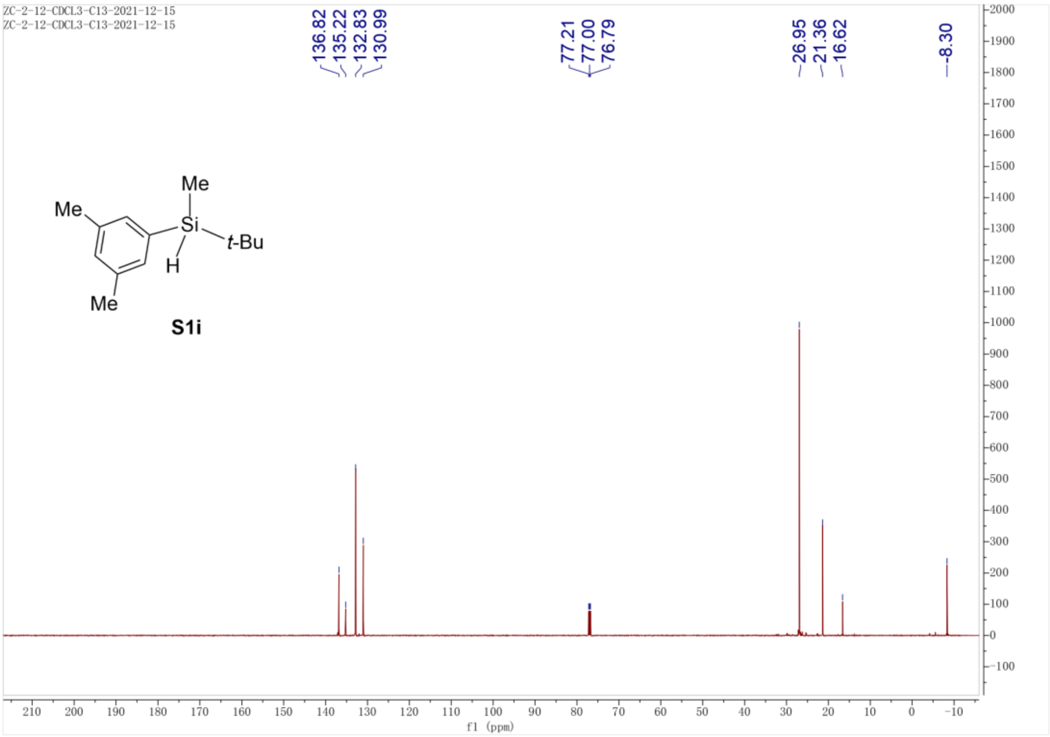

Supplementary Figure 33. ^{13}C NMR (150 M, $CDCl_3$, 25 °C) of compound **S1i**

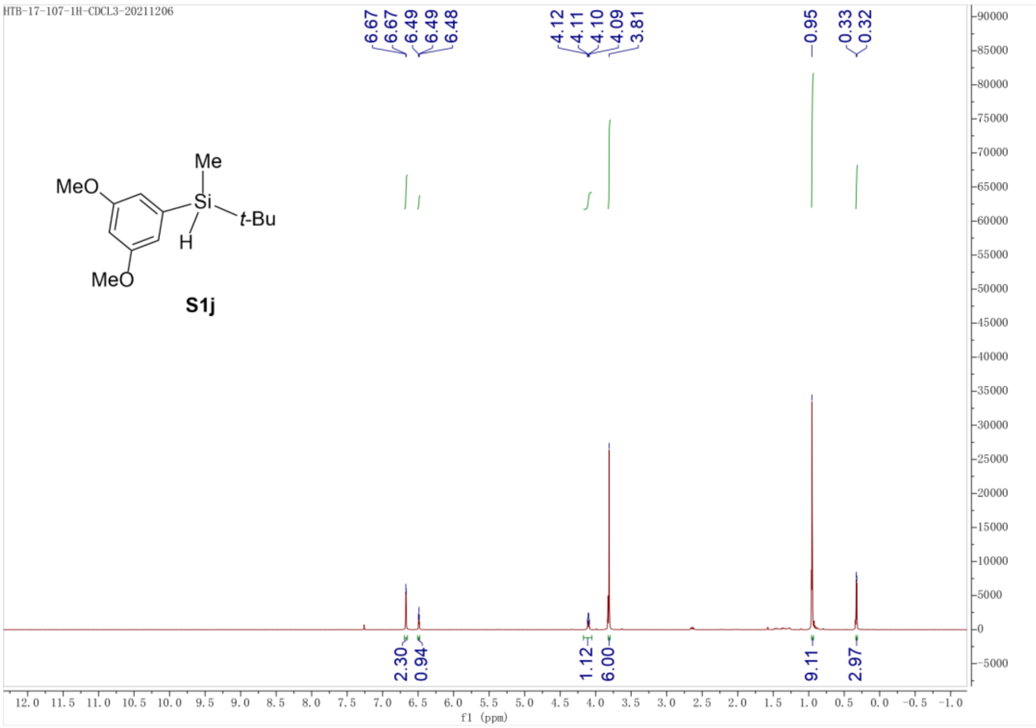

Supplementary Figure 34. ^{1}H NMR (400 M, $CDCl_3$, 25 °C) of compound **S1j**

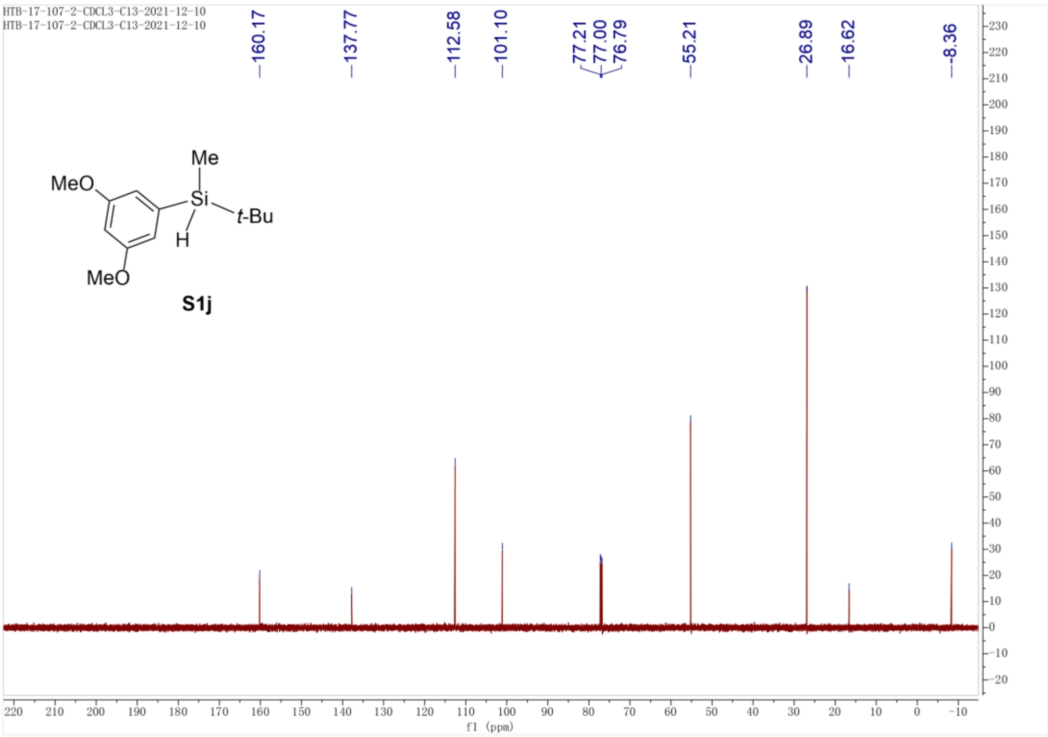

Supplementary Figure 35. ^{13}C NMR (100 M, $CDCl_3$, 25 °C) of compound **S1j**

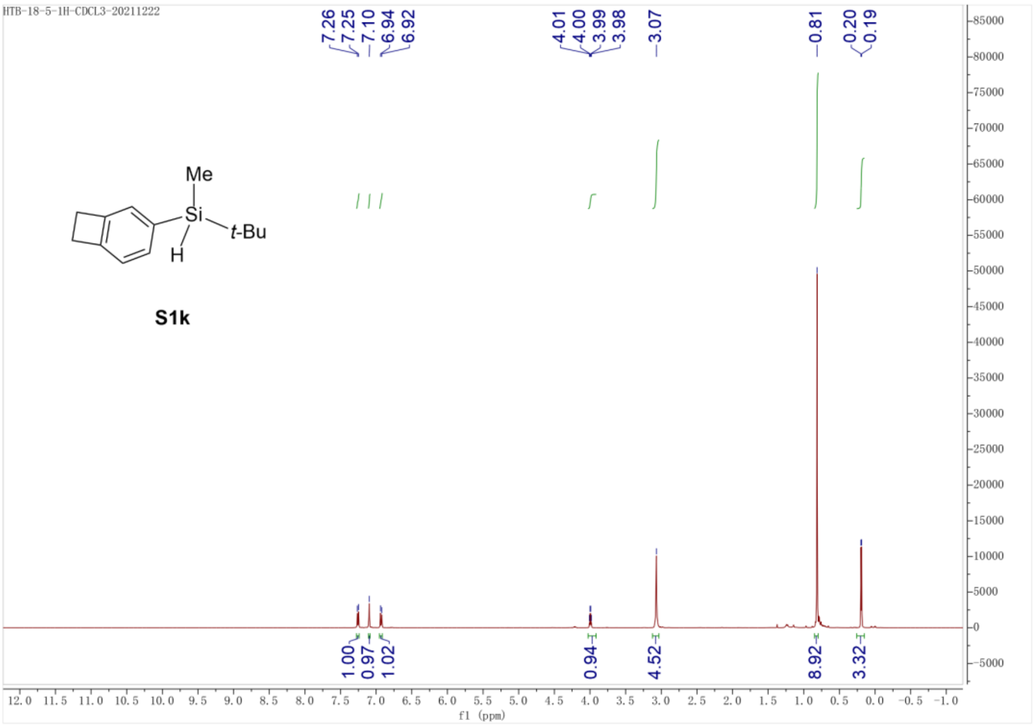

Supplementary Figure 36. ^{1}H NMR (400 M, $CDCl_3$, 25 °C) of compound **S1k**

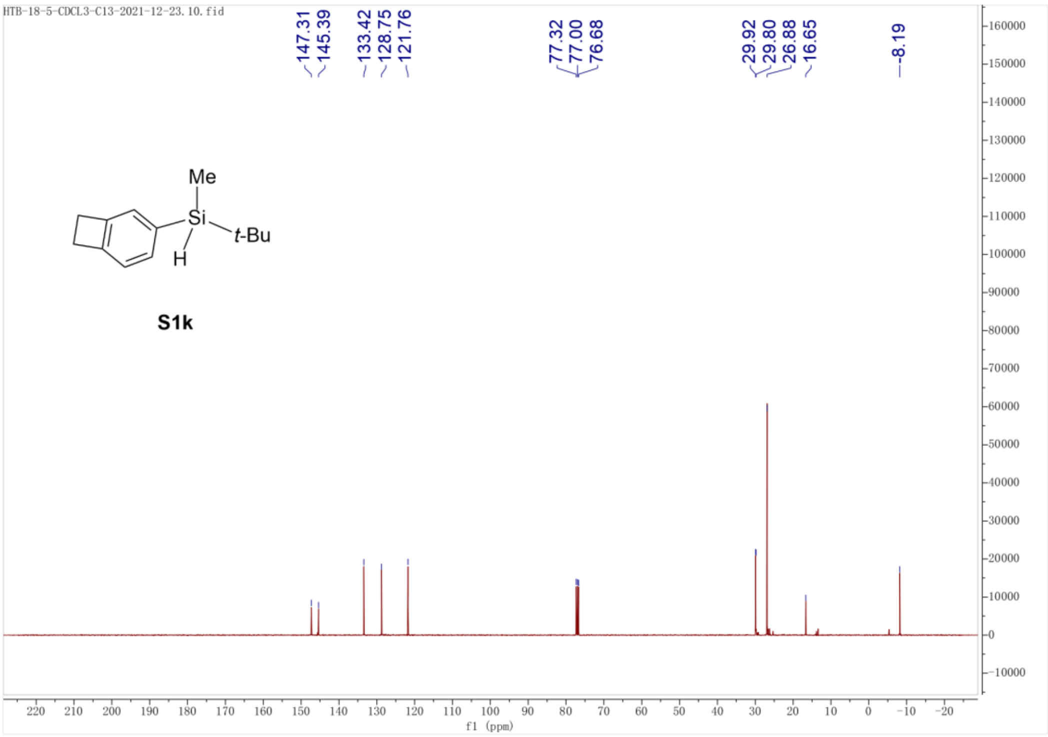

Supplementary Figure 37. ^{13}C NMR (100 M, $CDCl_3$, 25 °C) of compound **S1k**

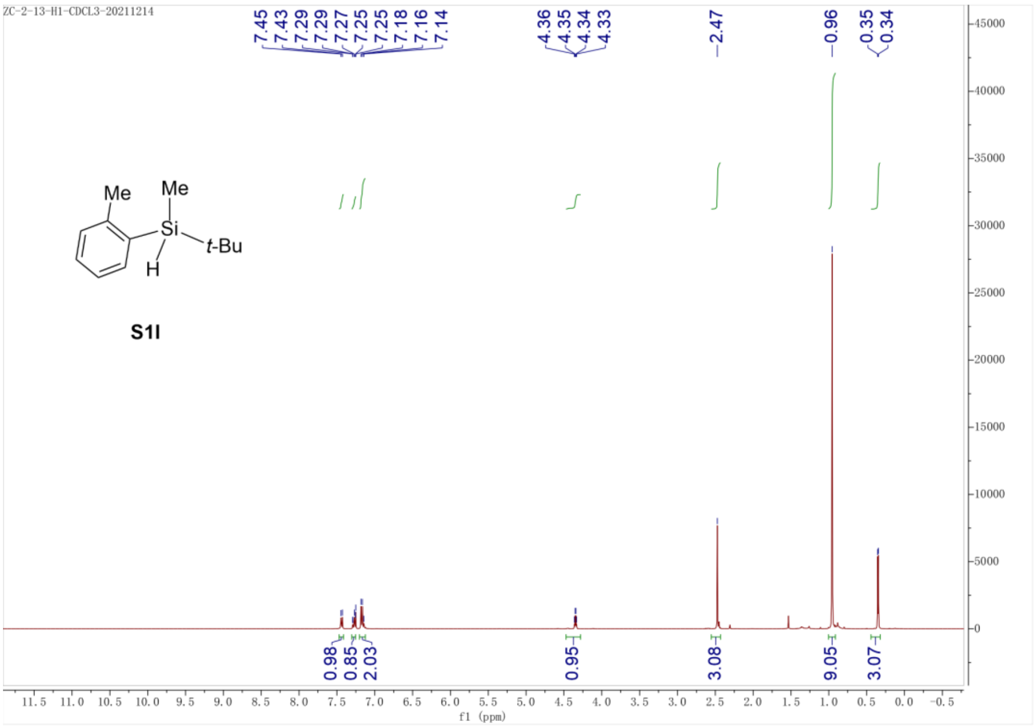

Supplementary Figure 38. ^{1}H NMR (400 M, $CDCl_3$, 25 °C) of compound **S1l**

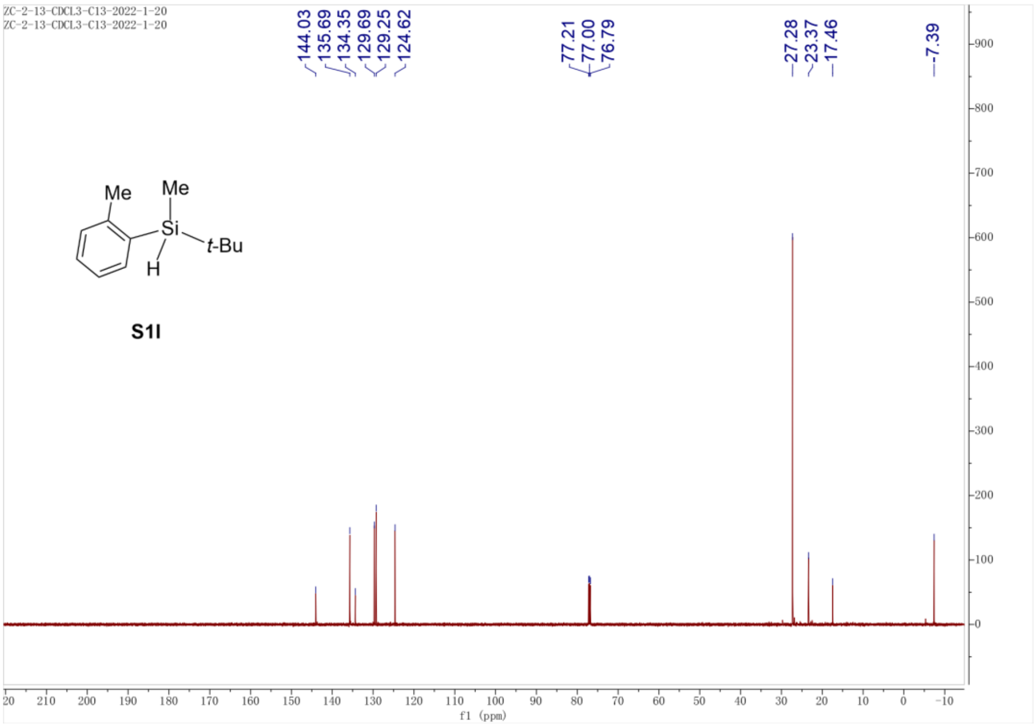

Supplementary Figure 39. ^{13}C NMR (100 M, $CDCl_3$, 25 °C) of compound **S1l**

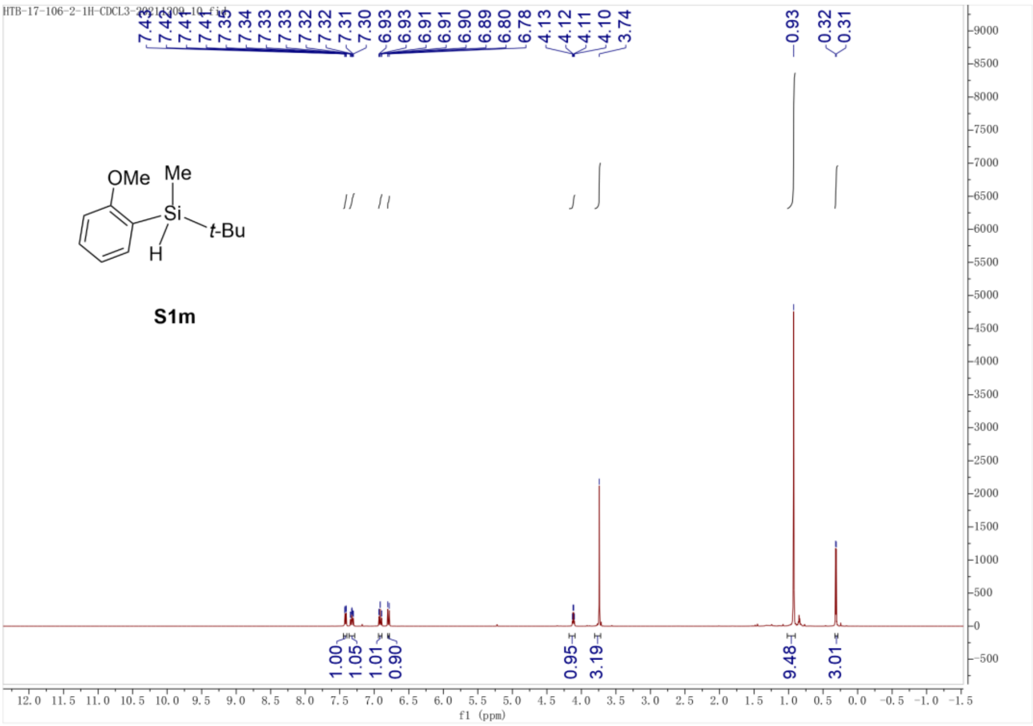

Supplementary Figure 40. ^{1}H NMR (400 M, $CDCl_3$, 25 °C) of compound **S1m**

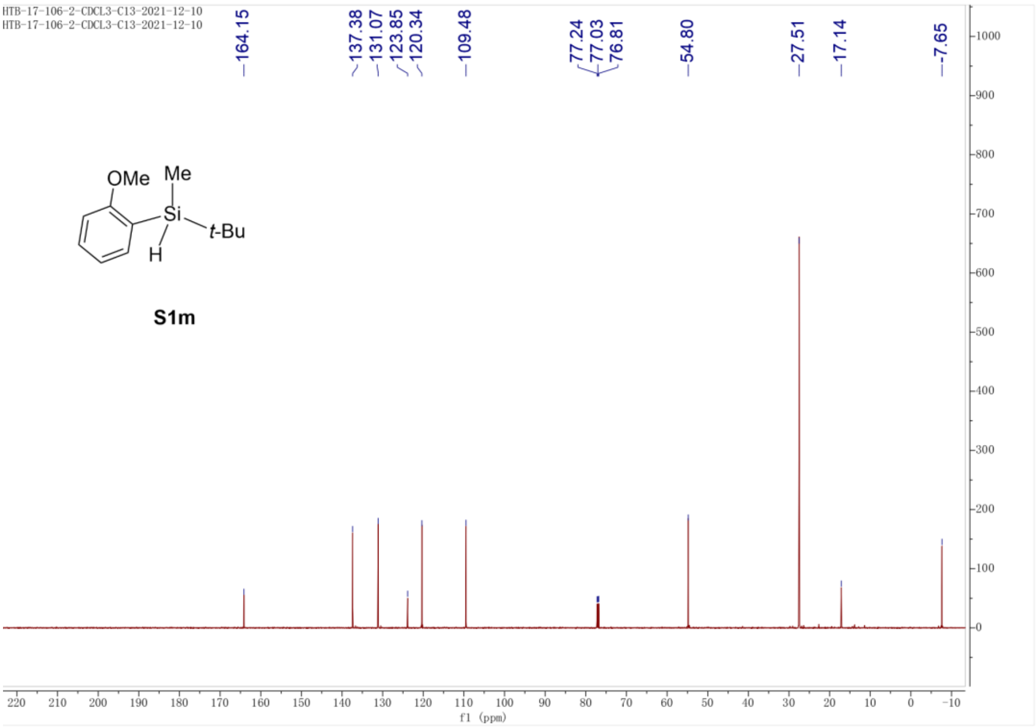

Supplementary Figure 41. ^{13}C NMR (100 M, $CDCl_3$, 25 °C) of compound **S1m**

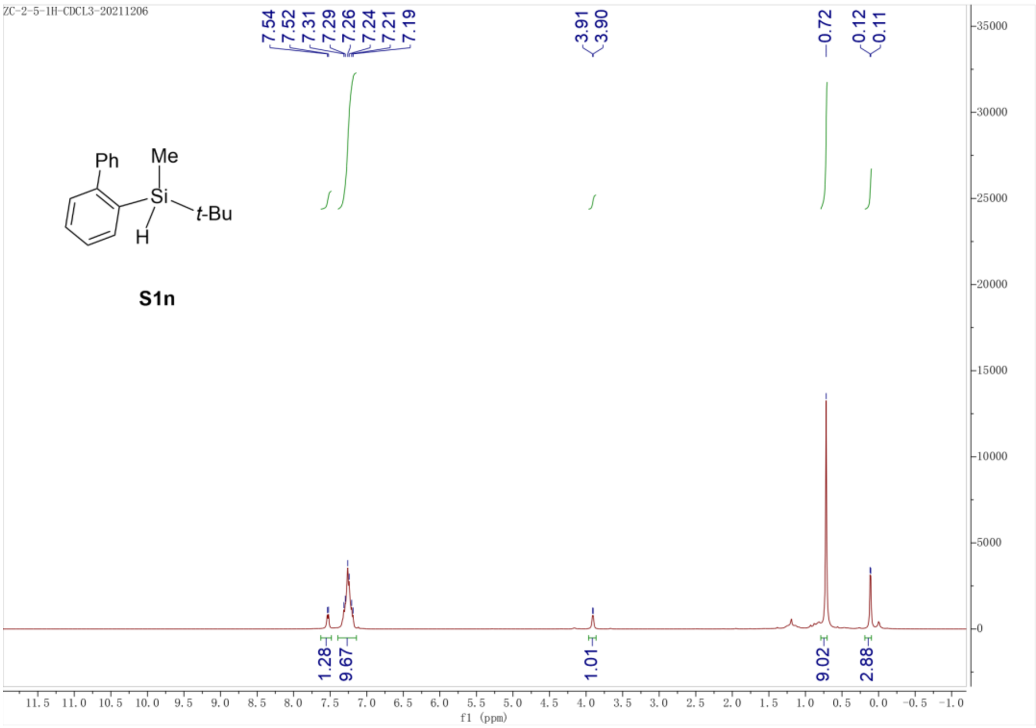

Supplementary Figure 42. ^{1}H NMR (400 M, $CDCl_3$, 25 °C) of compound **S1n**

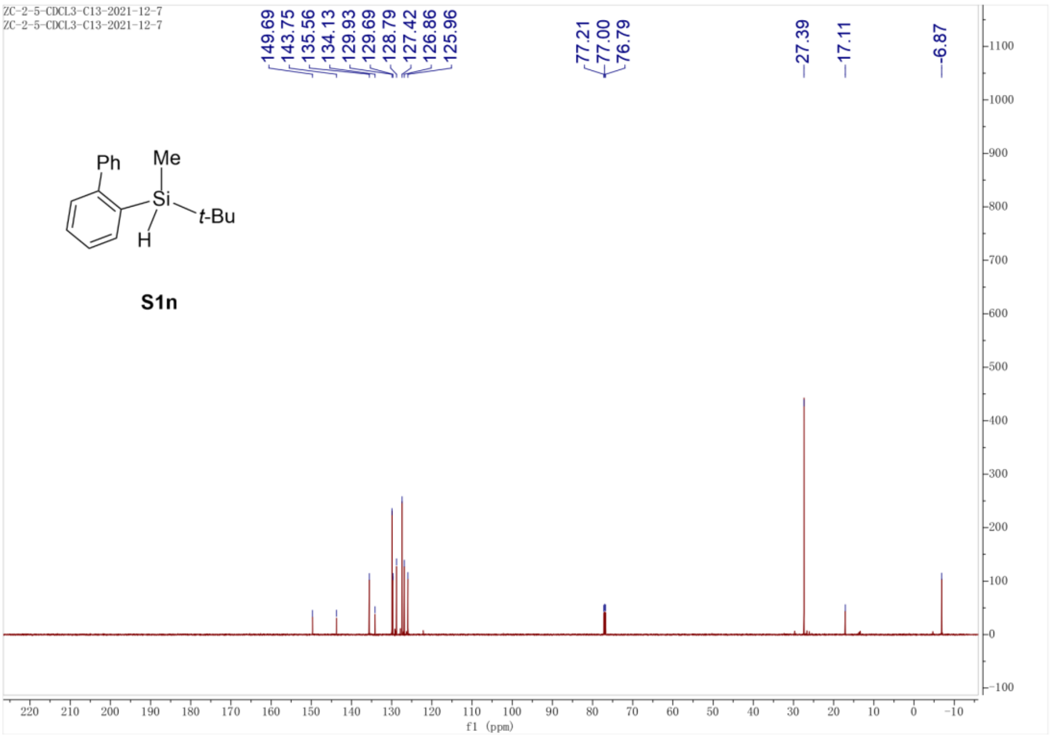

Supplementary Figure 43. ^{13}C NMR (100 M, $CDCl_3$, 25 °C) of compound **S1n**

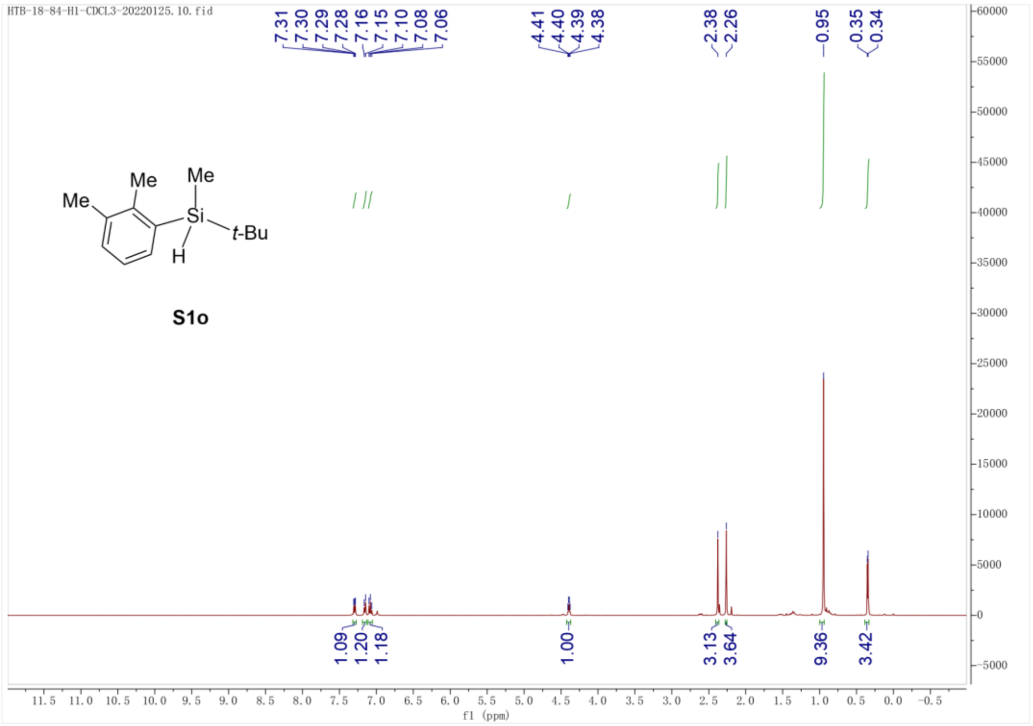

Supplementary Figure 44. ^{1}H NMR (400 M, $CDCl_3$, 25 °C) of compound **S1o**

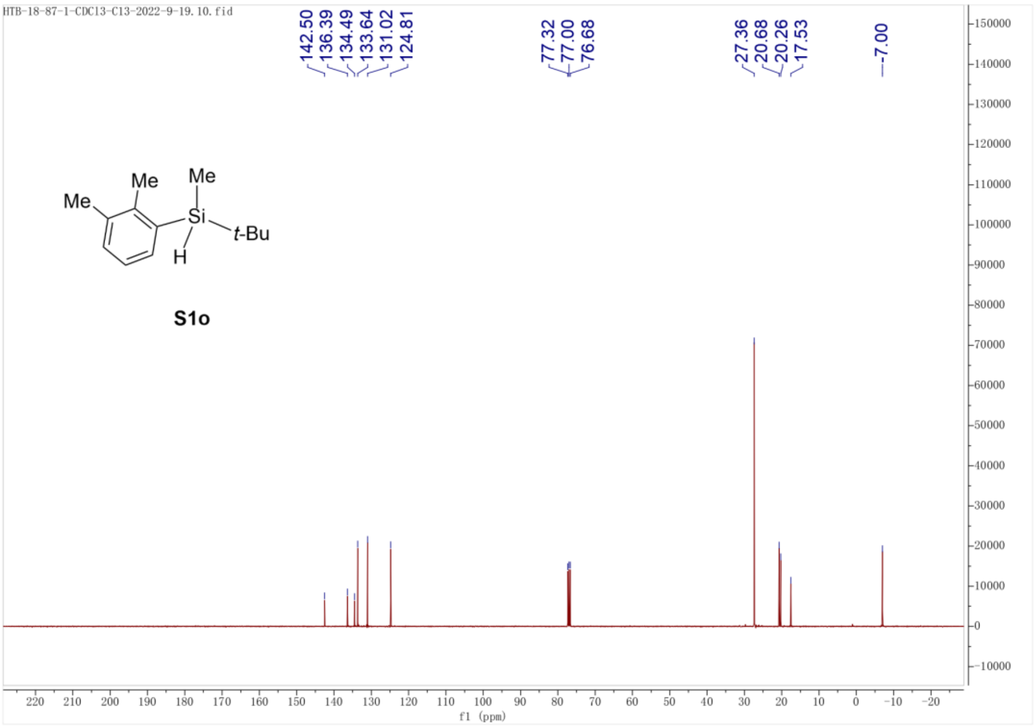

Supplementary Figure 45. ^{13}C NMR (100 M, $CDCl_3$, 25 °C) of compound **S1o**

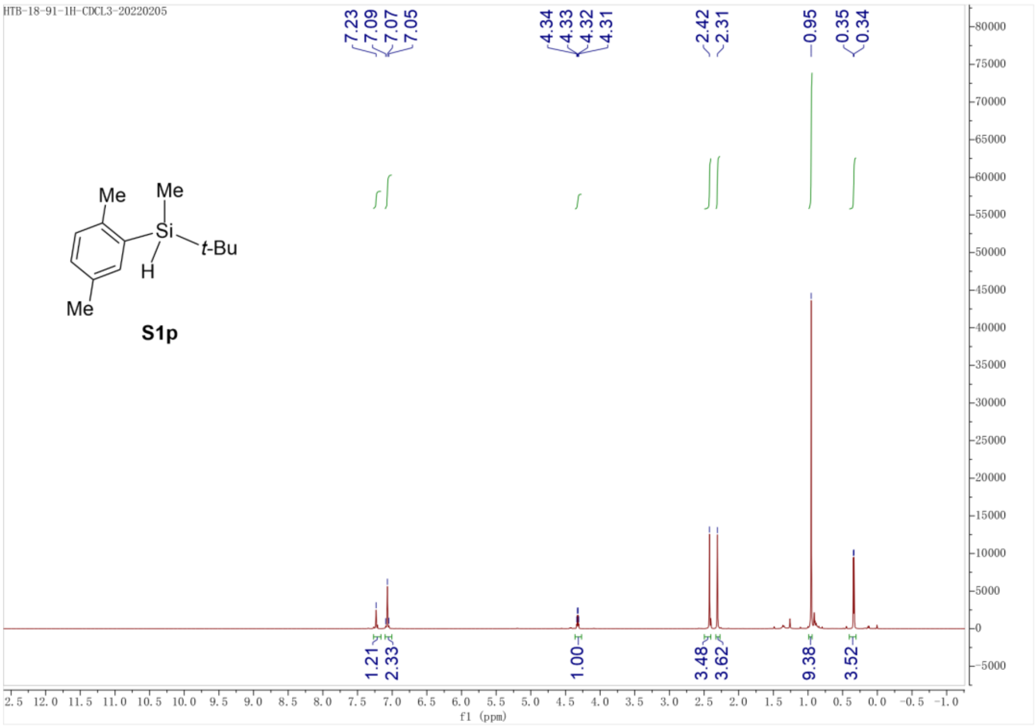

Supplementary Figure 46. ^{1}H NMR (400 M, $CDCl_3$, 25 °C) of compound **S1p**

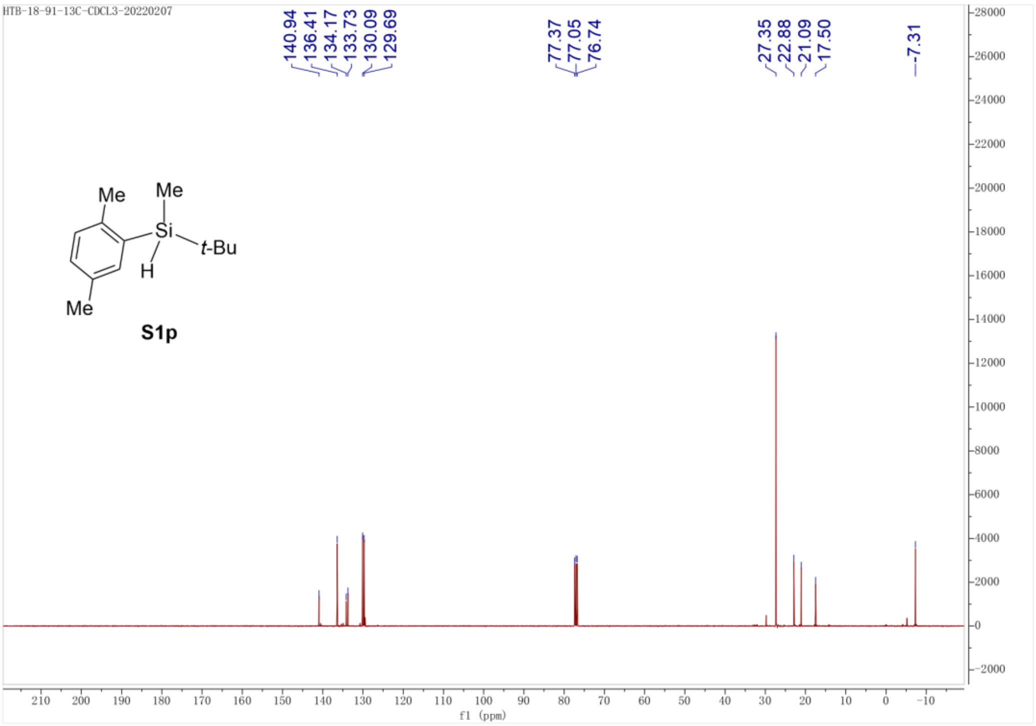

Supplementary Figure 47. ^{13}C NMR (100 M, $CDCl_3$, 25 °C) of compound **S1p**

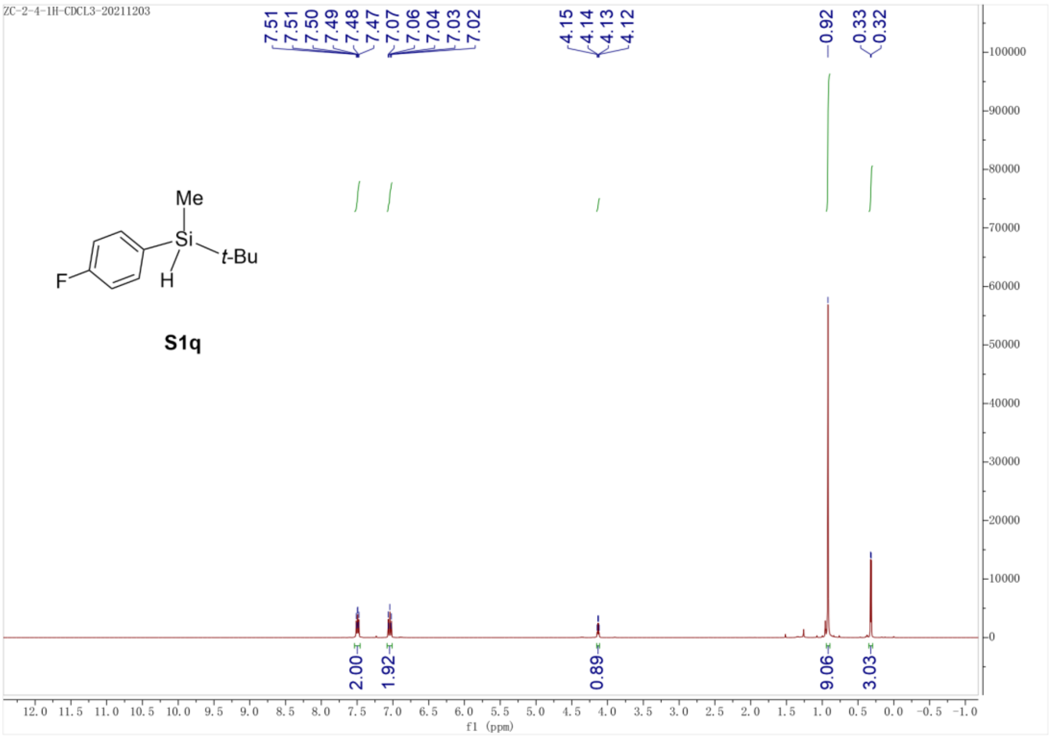

Supplementary Figure 48. ^{1}H NMR (400 M, $CDCl_3$, 25 °C) of compound **S1q**

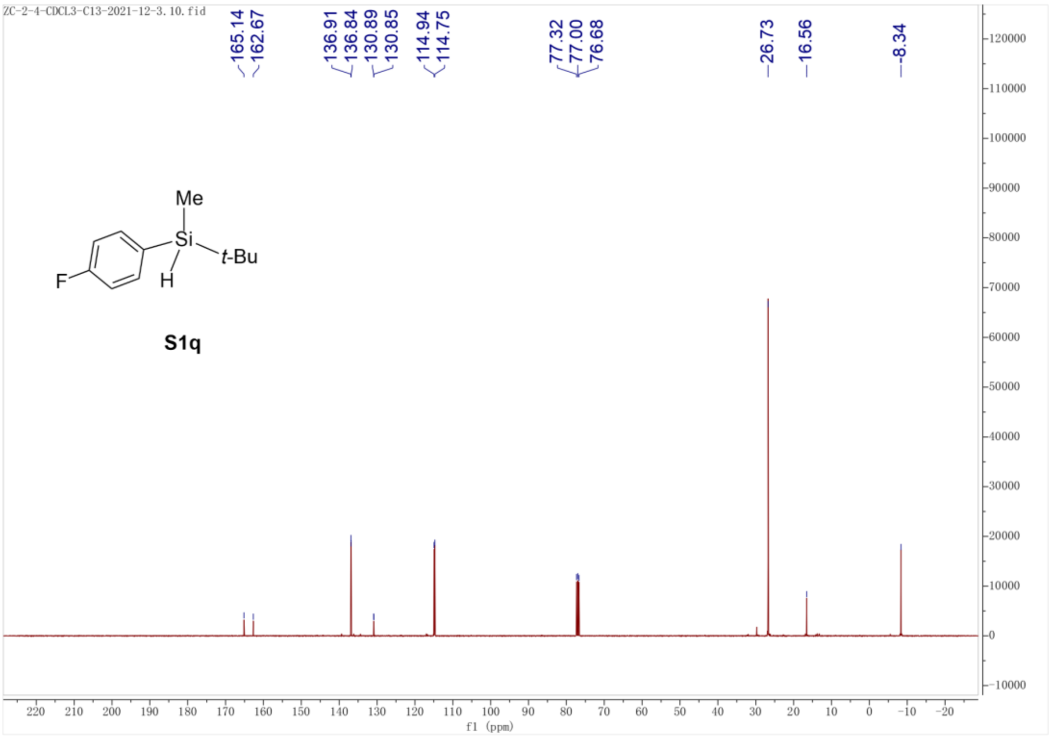

Supplementary Figure 49. ^{13}C NMR (100 M, $CDCl_3$, 25 °C) of compound **S1q**

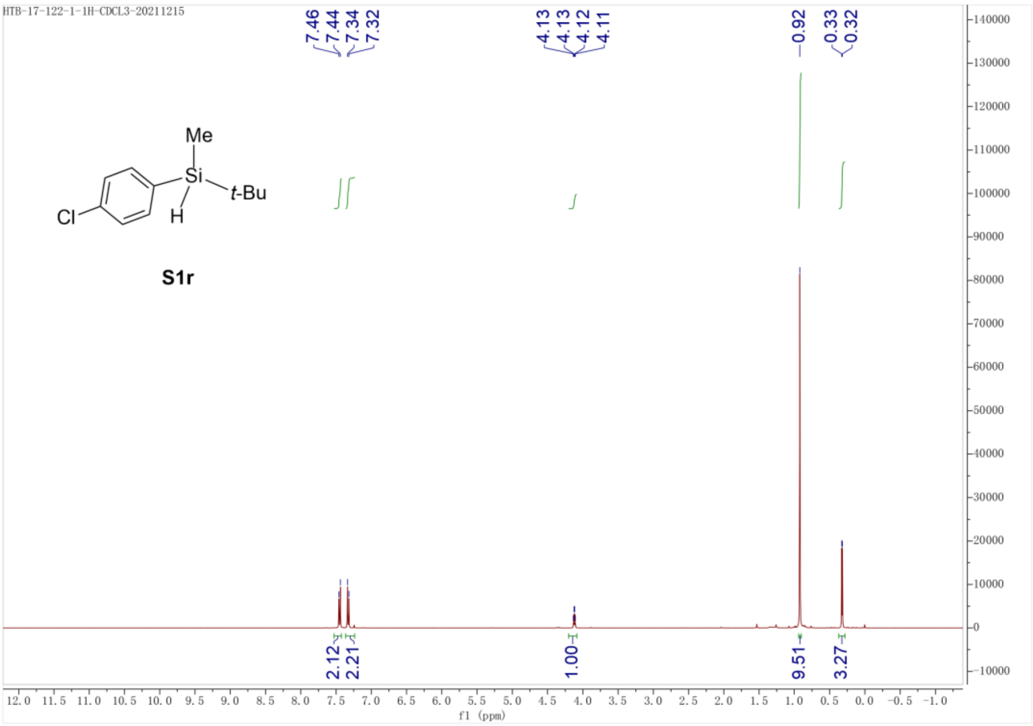

Supplementary Figure 50. ^{1}H NMR (400 M, $CDCl_3$, 25 °C) of compound **S1r**

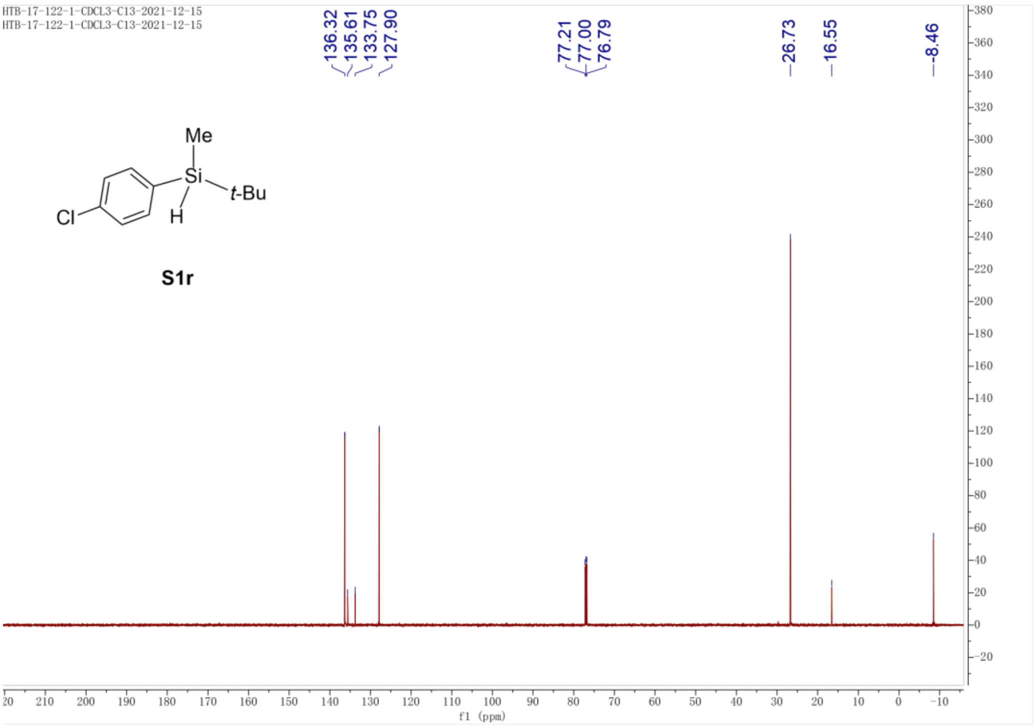

Supplementary Figure 51. ^{13}C NMR (100 M, $CDCl_3$, 25 °C) of compound **S1r**

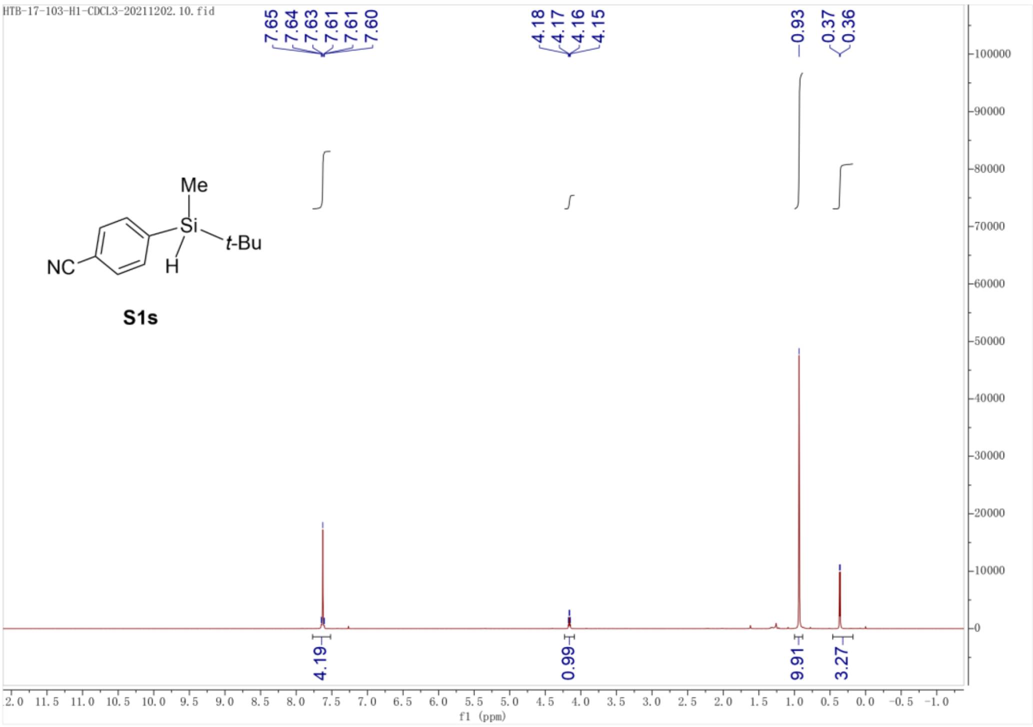

Supplementary Figure 52. ^{1}H NMR (400 M, $CDCl_3$, 25 °C) of compound **S1s**

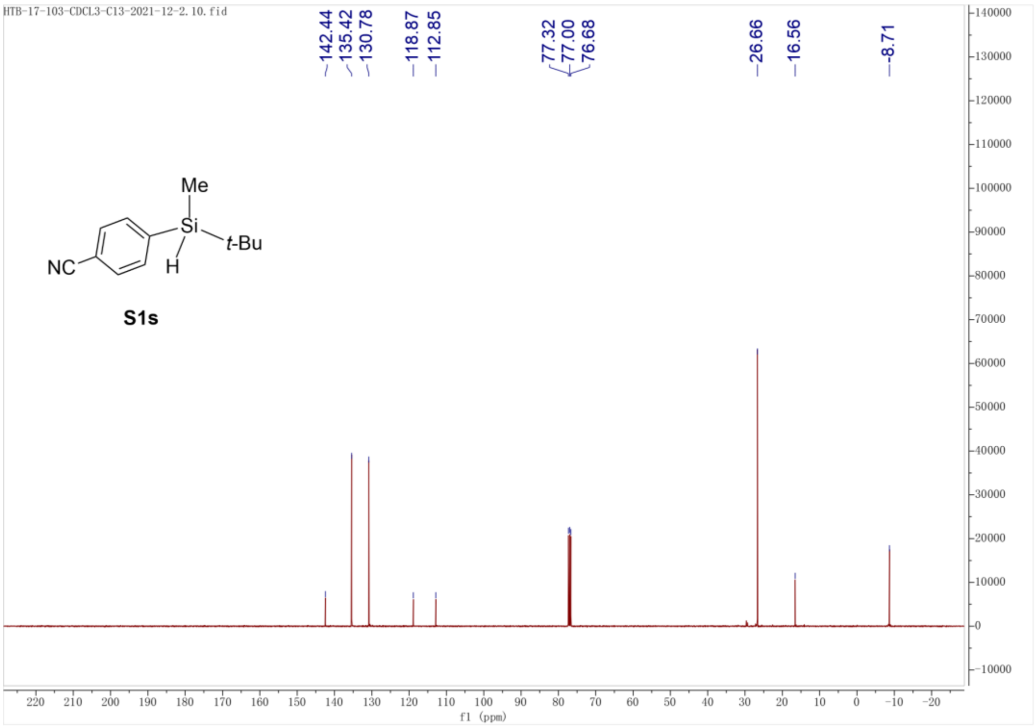

Supplementary Figure 53. ^{13}C NMR (100 M, $CDCl_3$, 25 ^{o}C) of compound **S1s**

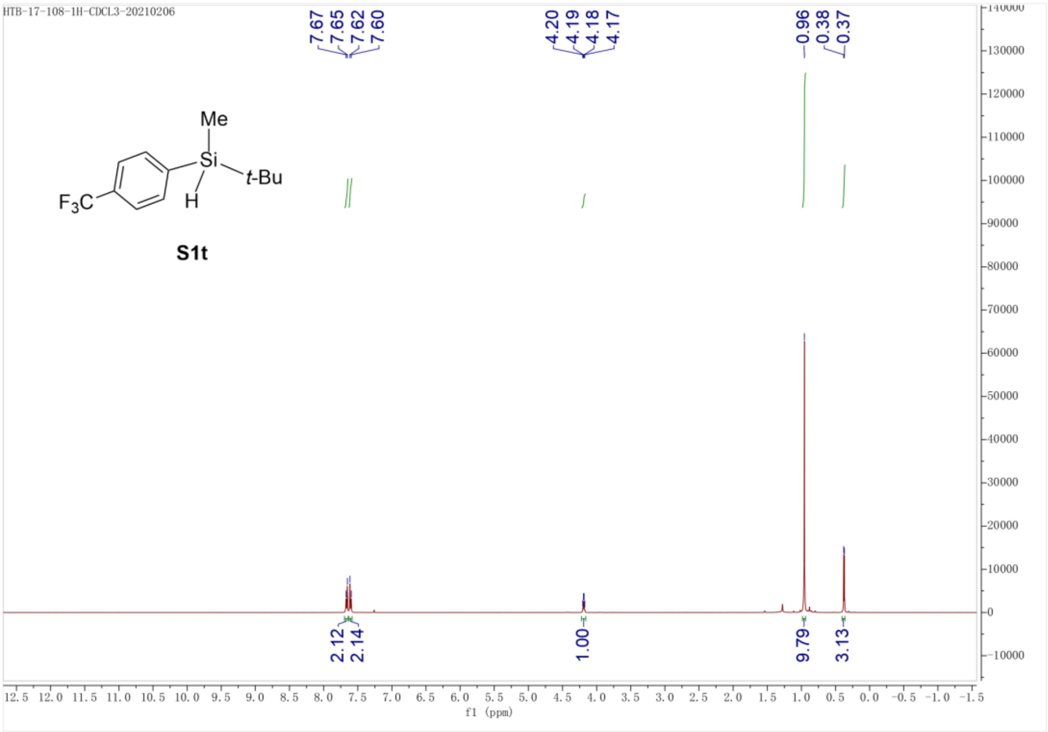

Supplementary Figure 54. ^{1}H NMR (400 M, $CDCl_3$, 25 ^{o}C) of compound **S1t**

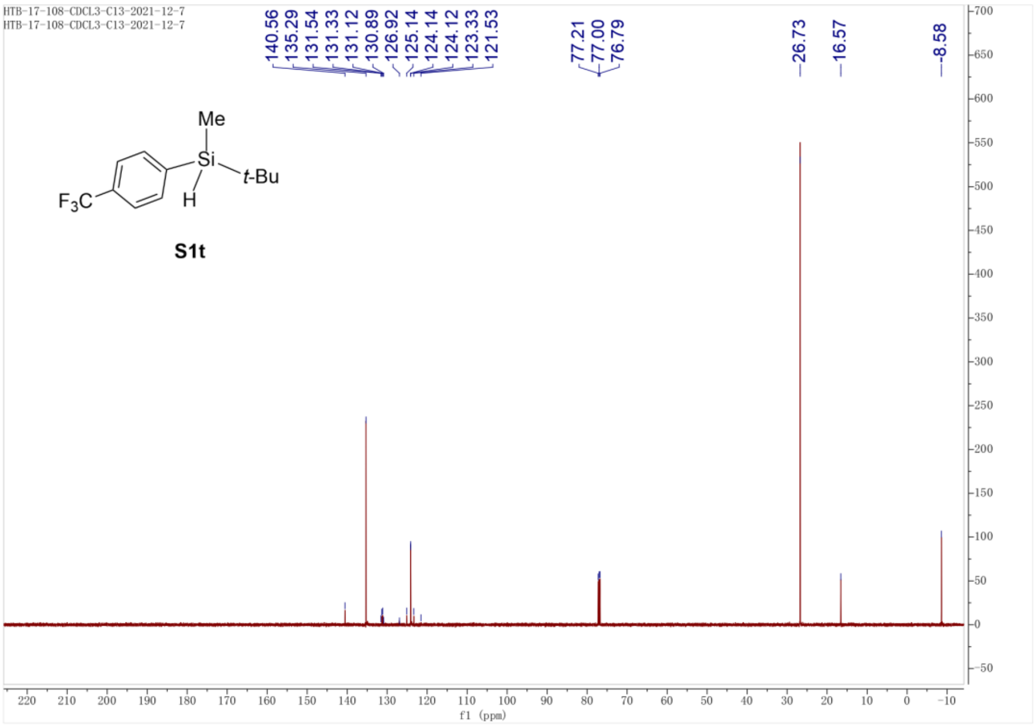

Supplementary Figure 55. ^{13}C NMR (150 M, $CDCl_3$, 25 °C) of compound **S1t**

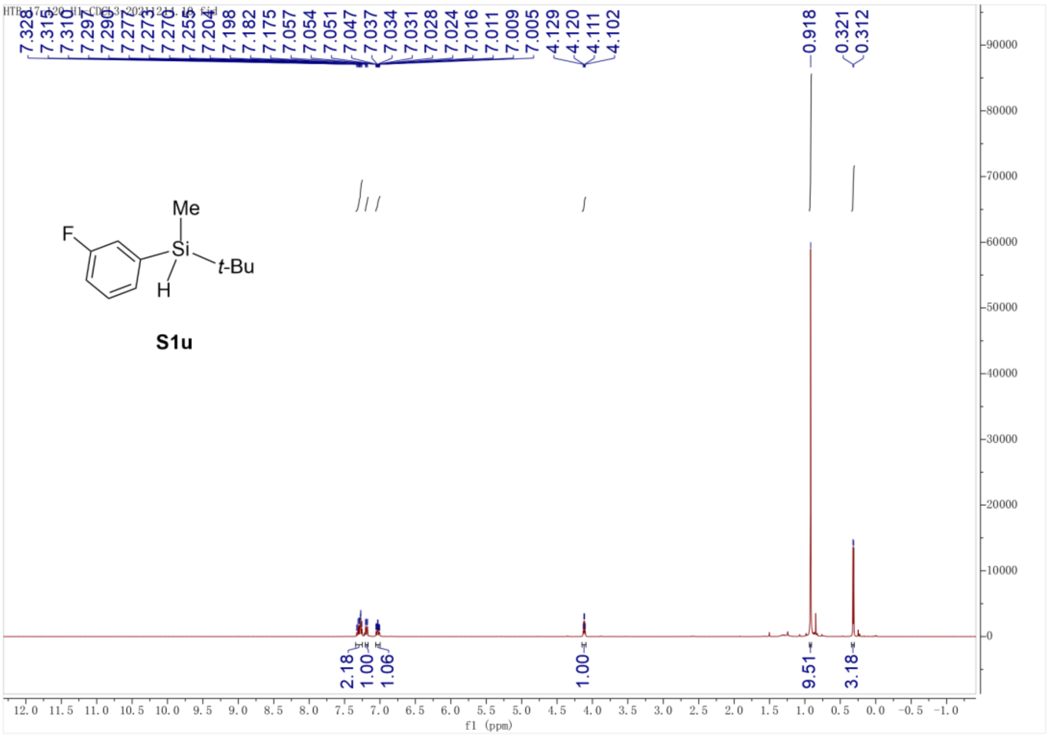

Supplementary Figure 56. ^{1}H NMR (400 M, $CDCl_3$, 25 °C) of compound **S1u**

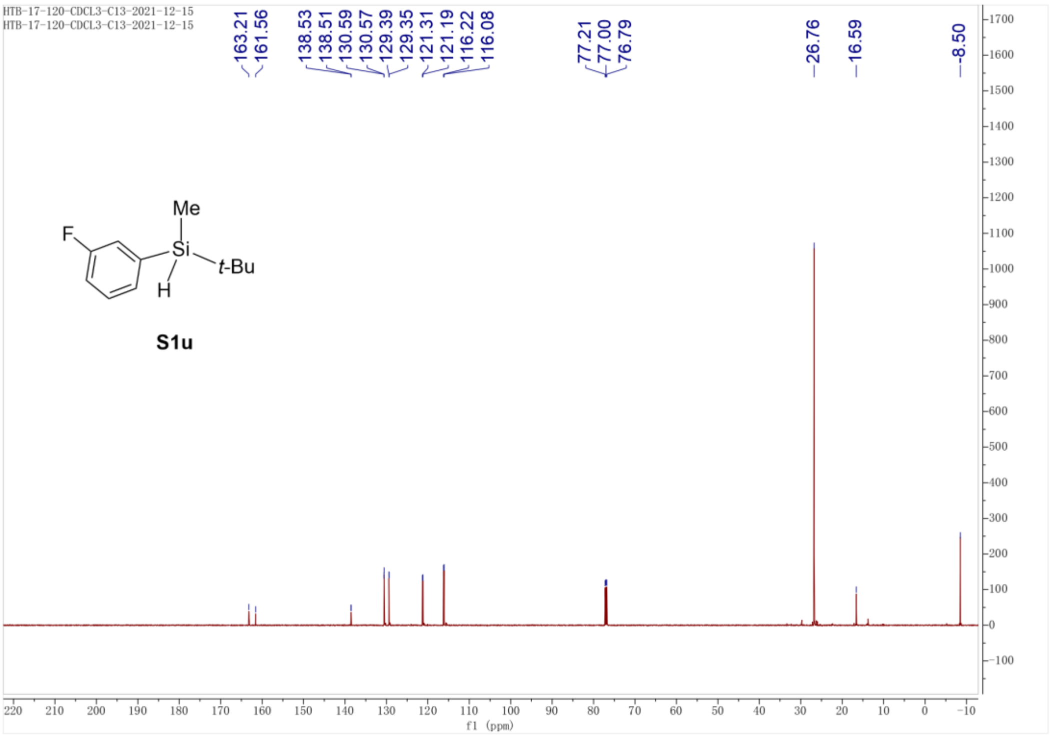

Supplementary Figure 57. ^{13}C NMR (150 M, $CDCl_3$, 25 °C) of compound **S1u**

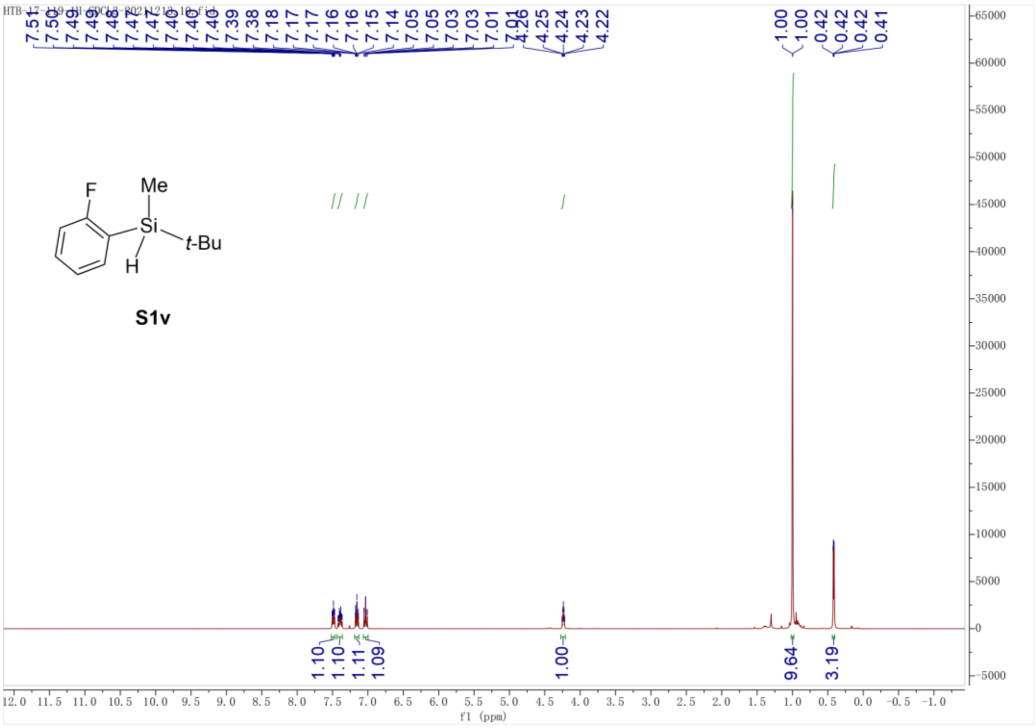

Supplementary Figure 58. ^{1}H NMR (400 M, $CDCl_3$, 25 °C) of compound **S1v**

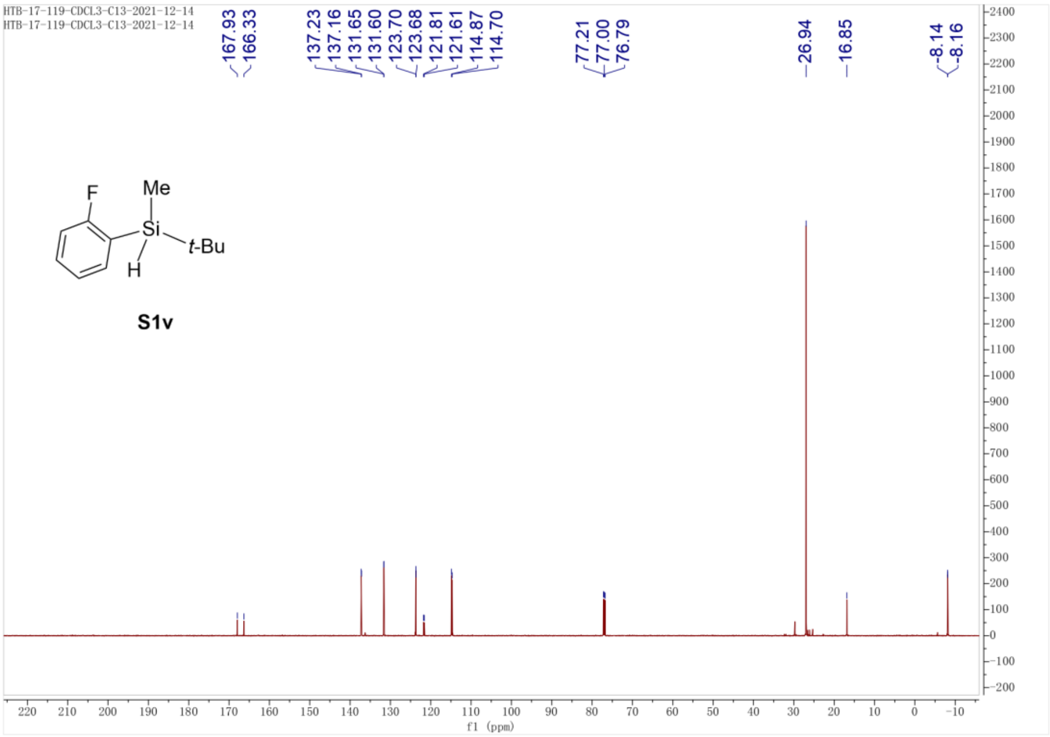

Supplementary Figure 59. ^{13}C NMR (150 M, $CDCl_3$, 25 ^{o}C) of compound **S1v**

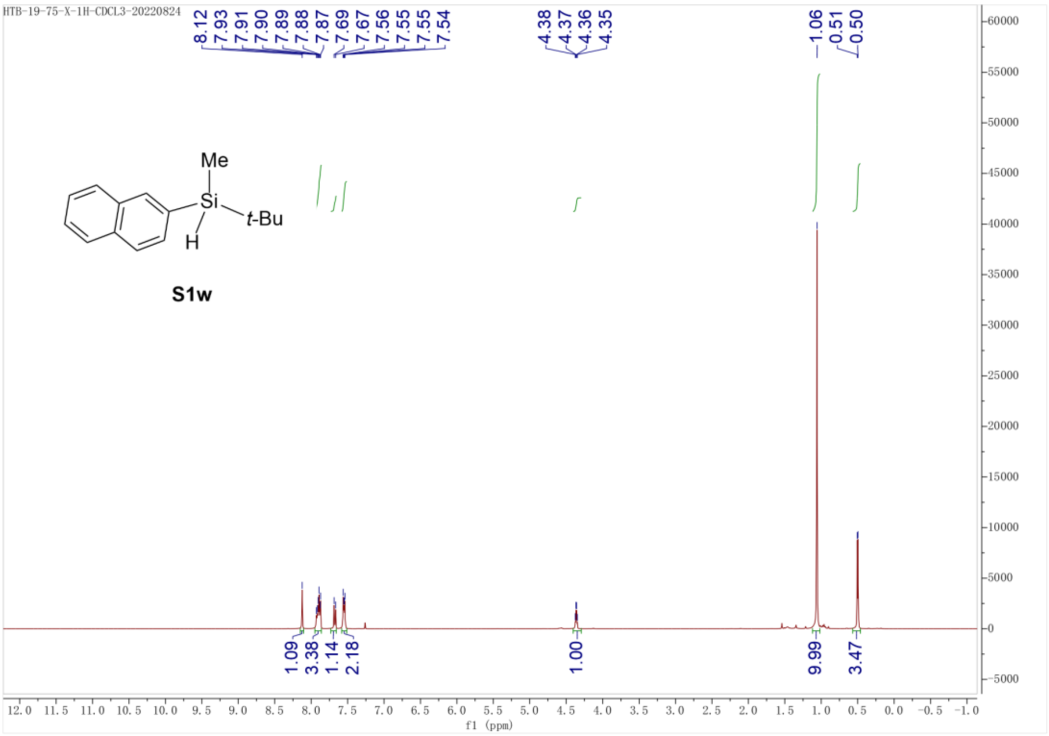

Supplementary Figure 60. ^{1}H NMR (400 M, $CDCl_3$, 25 ^{o}C) of compound **S1w**

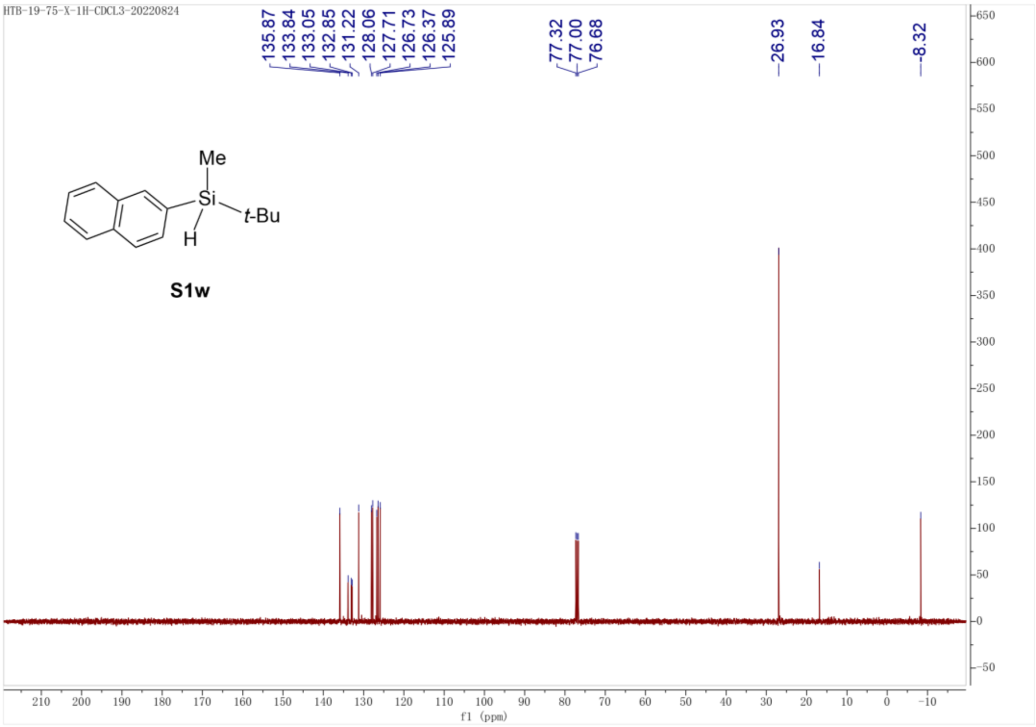

Supplementary Figure 61. ^{13}C NMR (100 M, $CDCl_3$, 25 °C) of compound **S1w**

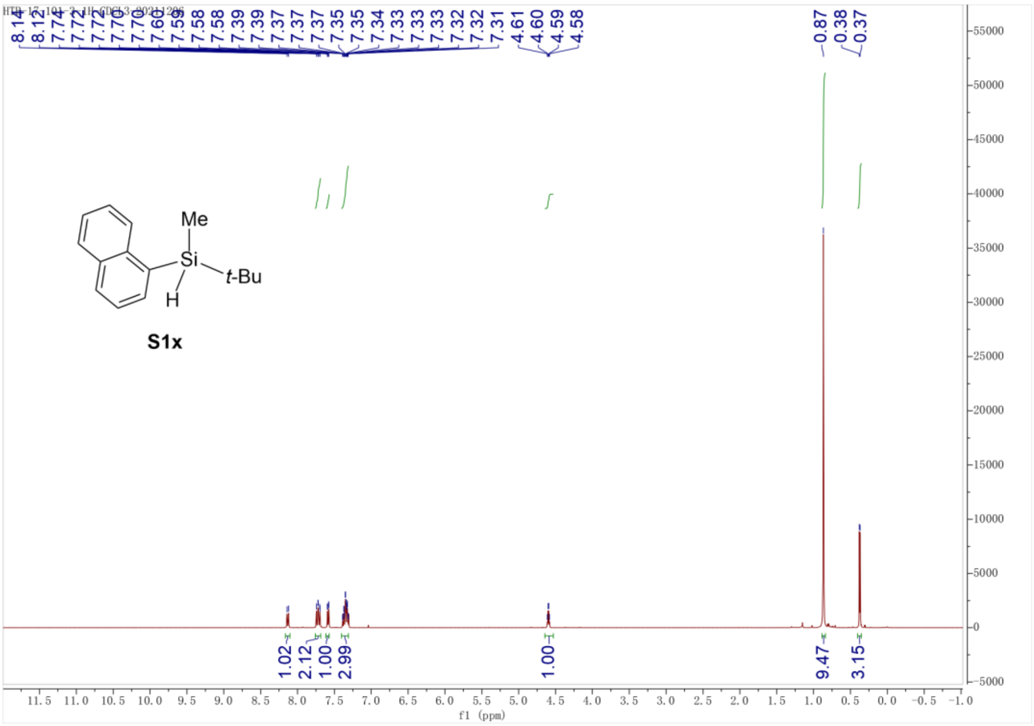

Supplementary Figure 62. ^{1}H NMR (400 M, $CDCl_3$, 25 °C) of compound **S1x**

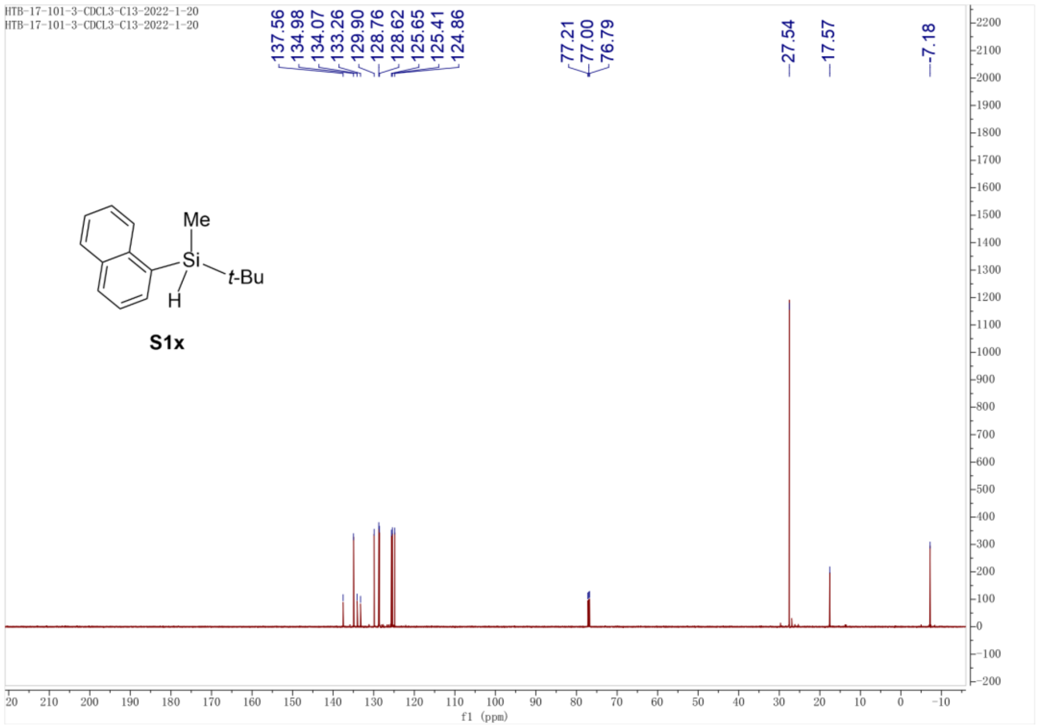

Supplementary Figure 63. ^{13}C NMR (100 M, $CDCl_3$, 25 °C) of compound **S1x**

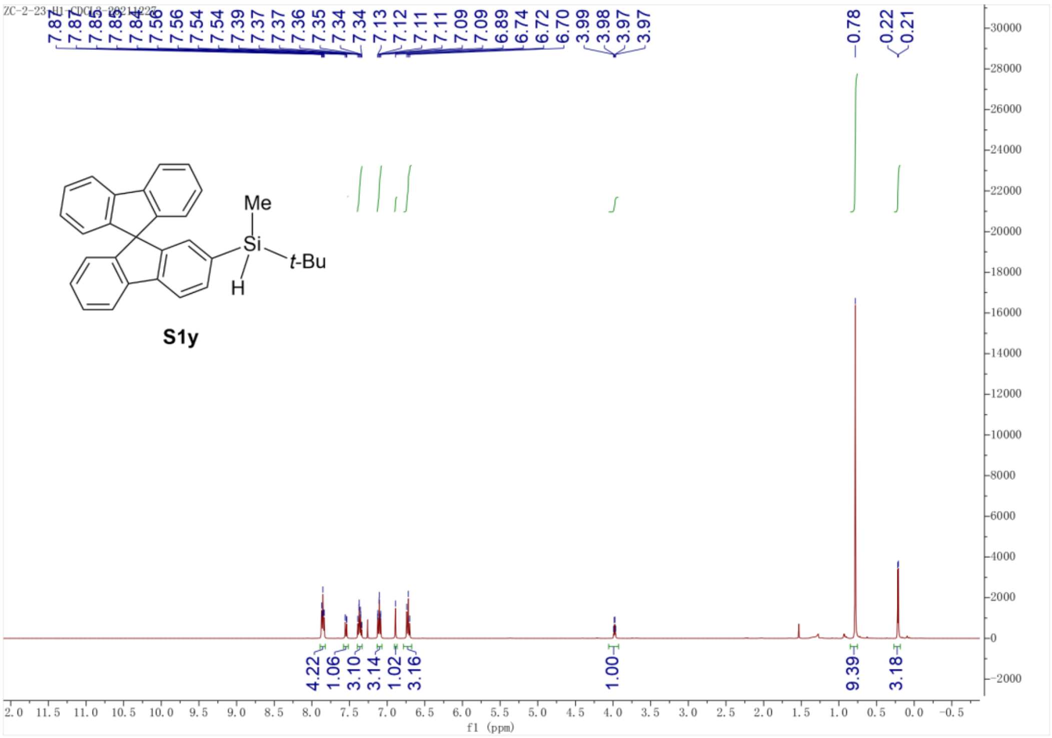

Supplementary Figure 64. ^{1}H NMR (400 M, $CDCl_3$, 25 °C) of compound **S1y**

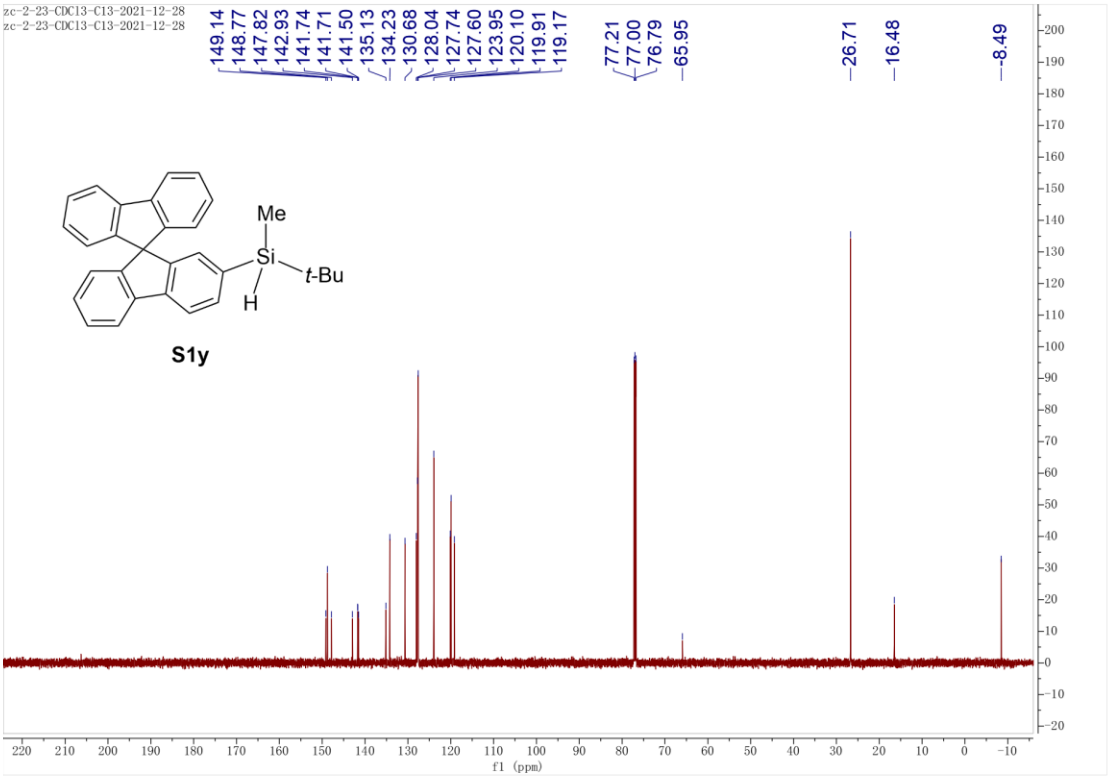

Supplementary Figure 65. ^{13}C NMR (100 M, $CDCl_3$, 25 °C) of compound **S1y**

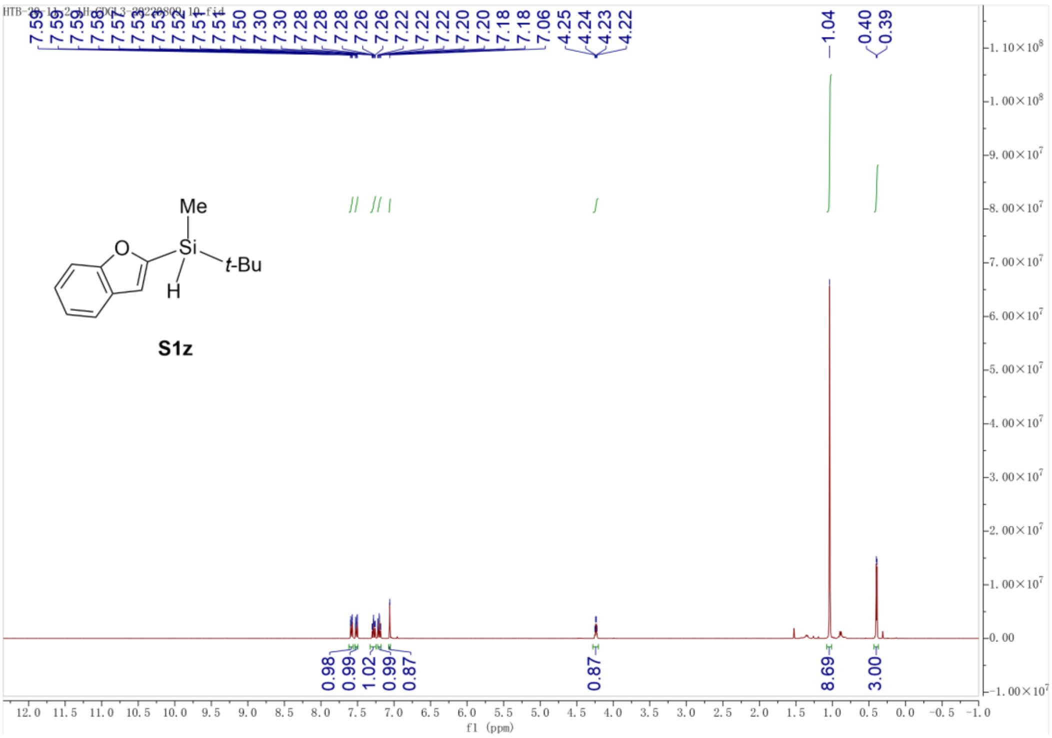

Supplementary Figure 66. ^{1}H NMR (400 M, $CDCl_3$, 25 °C) of compound **S1z**

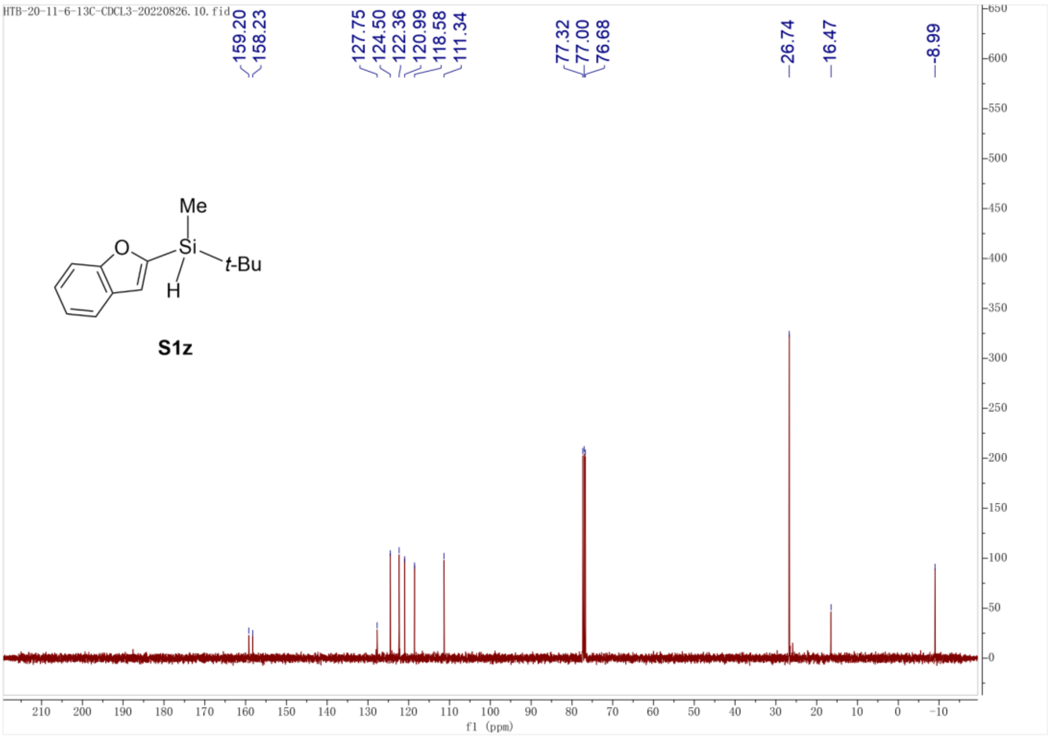

Supplementary Figure 67. ^{13}C NMR (100 M, $CDCl_3$, 25 °C) of compound **S1z**

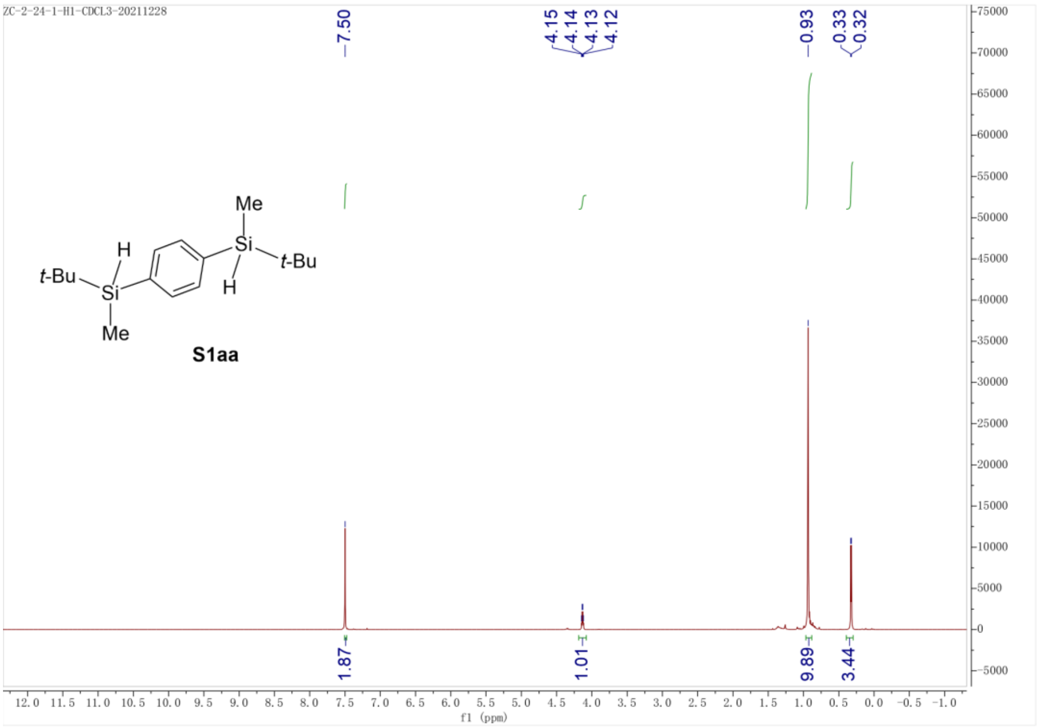

Supplementary Figure 68. ^{1}H NMR (400 M, $CDCl_3$, 25 °C) of compound **S1aa**

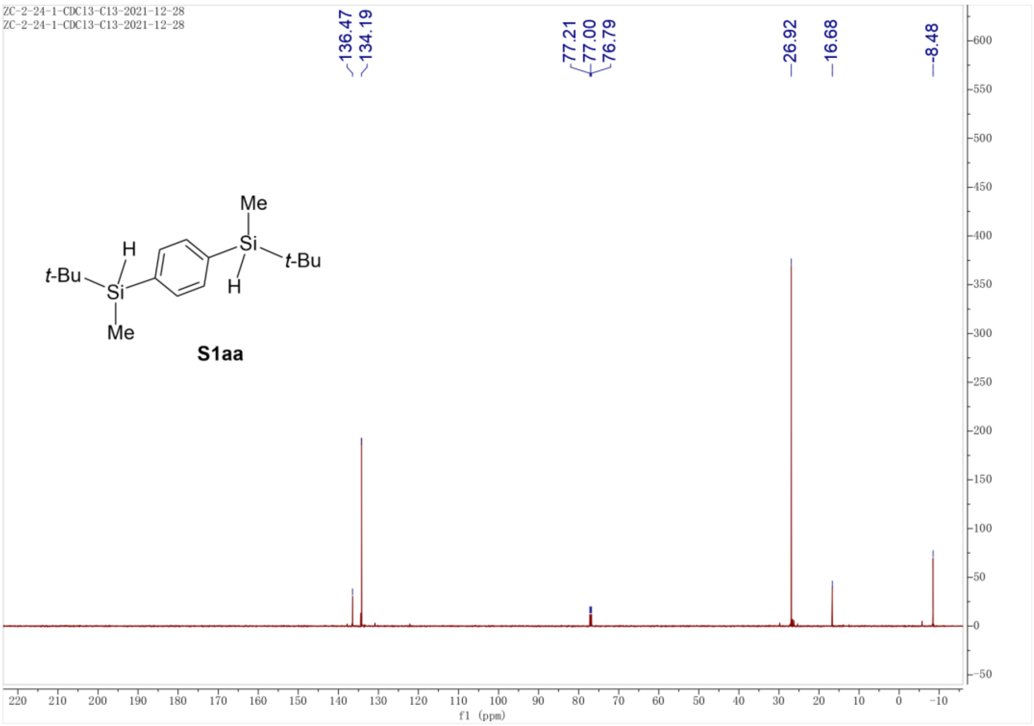

Supplementary Figure 69. ^{13}C NMR (100 M, $CDCl_3$, 25 °C) of compound **S1aa**

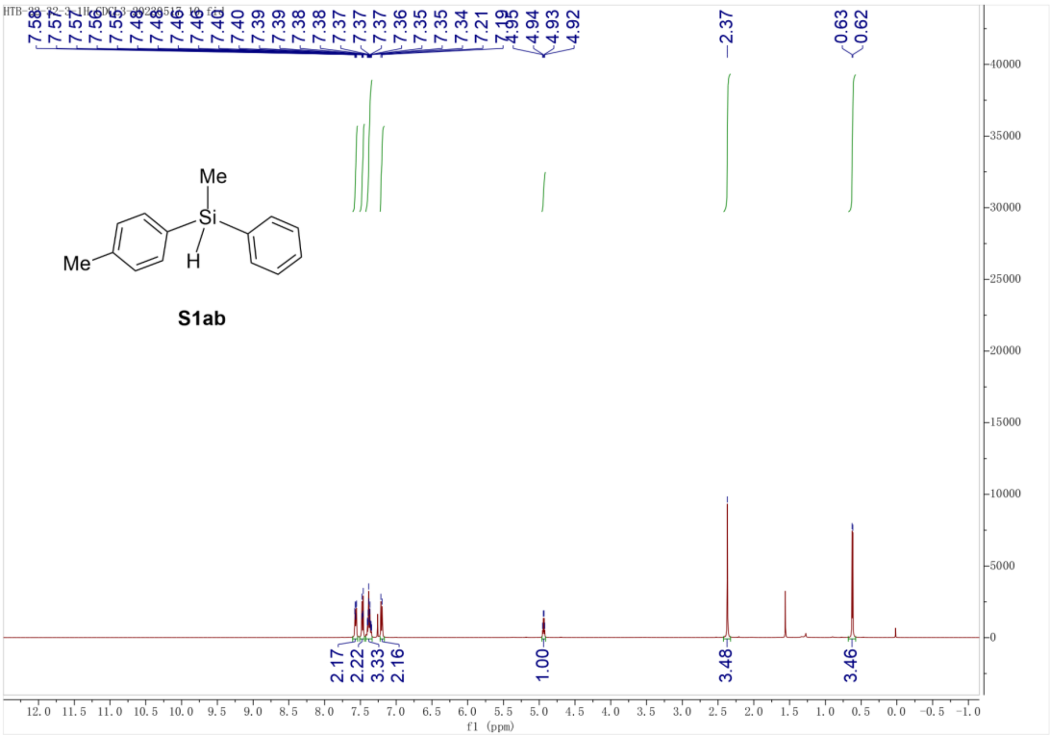

Supplementary Figure 70. ^{1}H NMR (400 M, $CDCl_3$, 25 °C) of compound **S1ab**

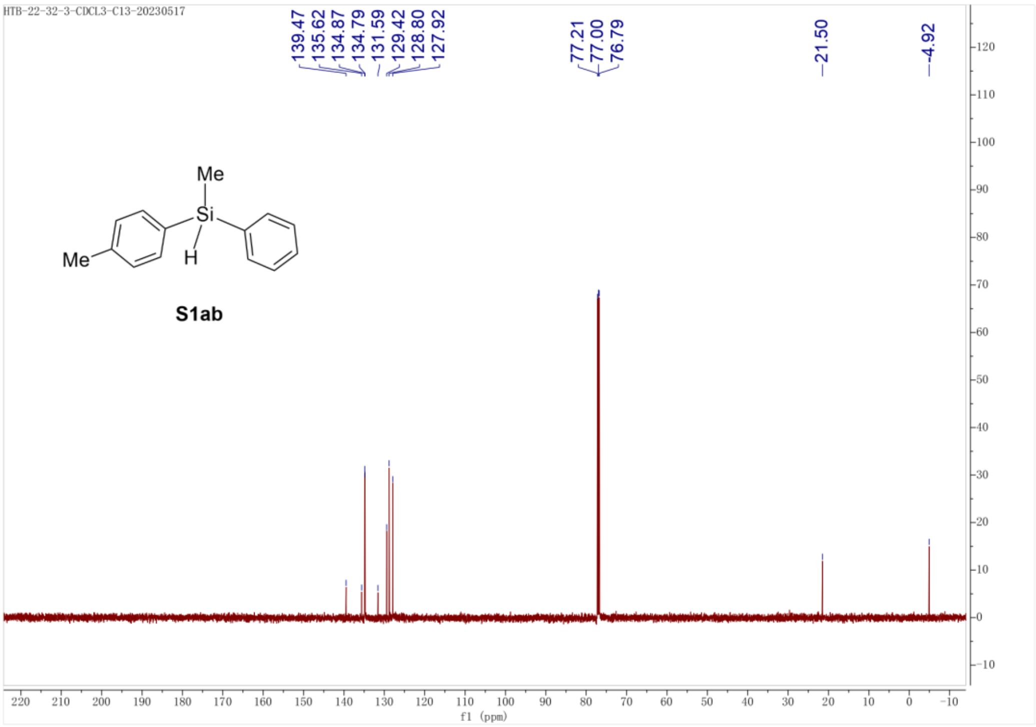

Supplementary Figure 71. ^{13}C NMR (100 M, $CDCl_3$, 25 °C) of compound **S1ab**

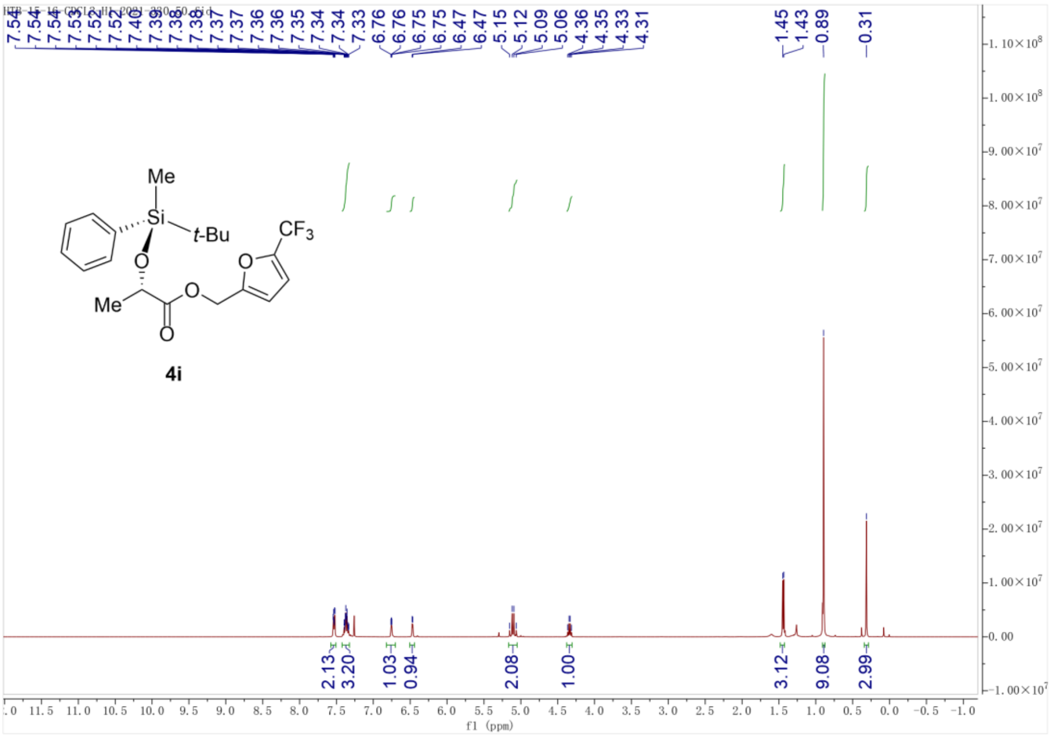

Supplementary Figure 72. ^{1}H NMR (400 M, $CDCl_3$, 25 °C) of compound **4i**

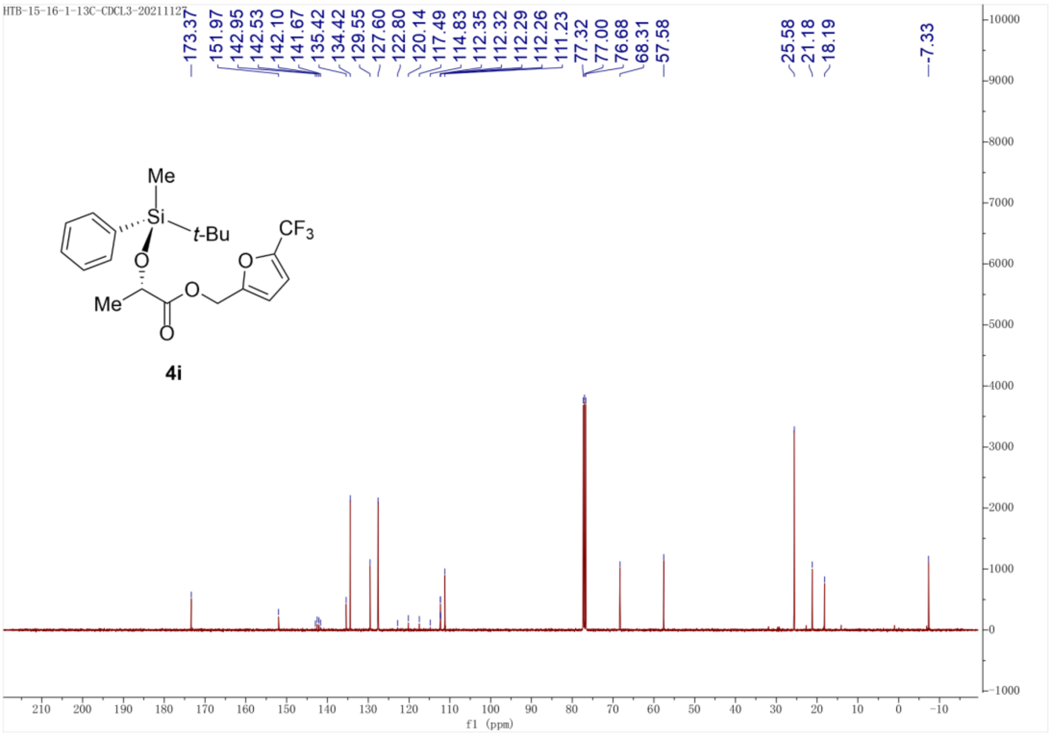

Supplementary Figure 73. ^{13}C NMR (100 M, $CDCl_3$, 25 °C) of compound **4i**

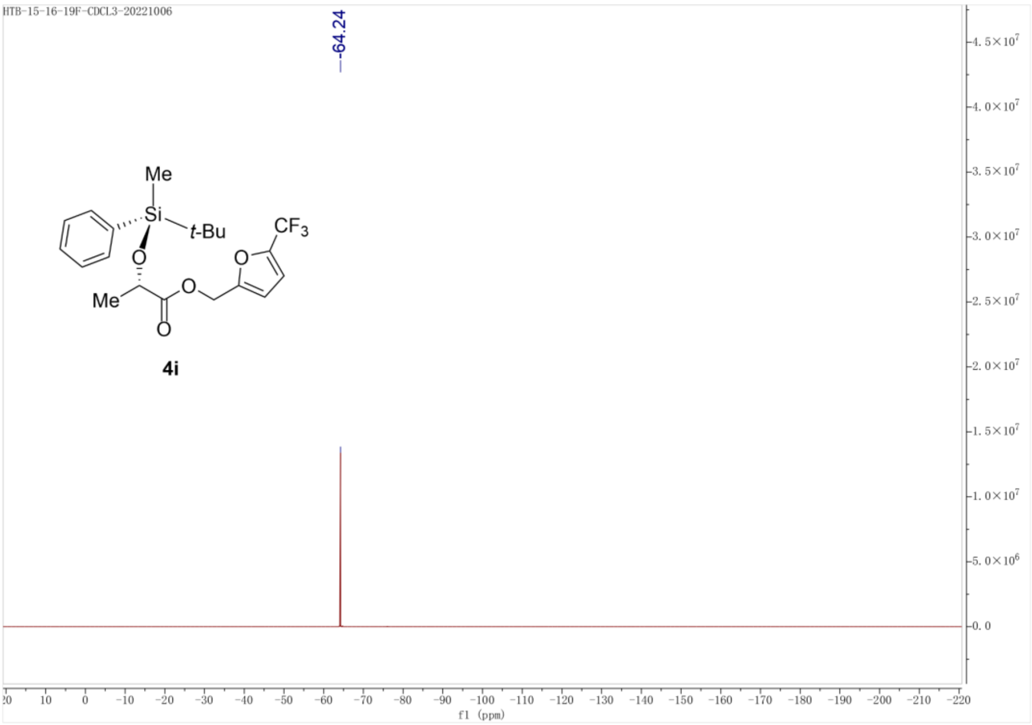

Supplementary Figure 74. ^{19}F NMR (376 M, $CDCl_3$, 25 °C) of compound **4i**

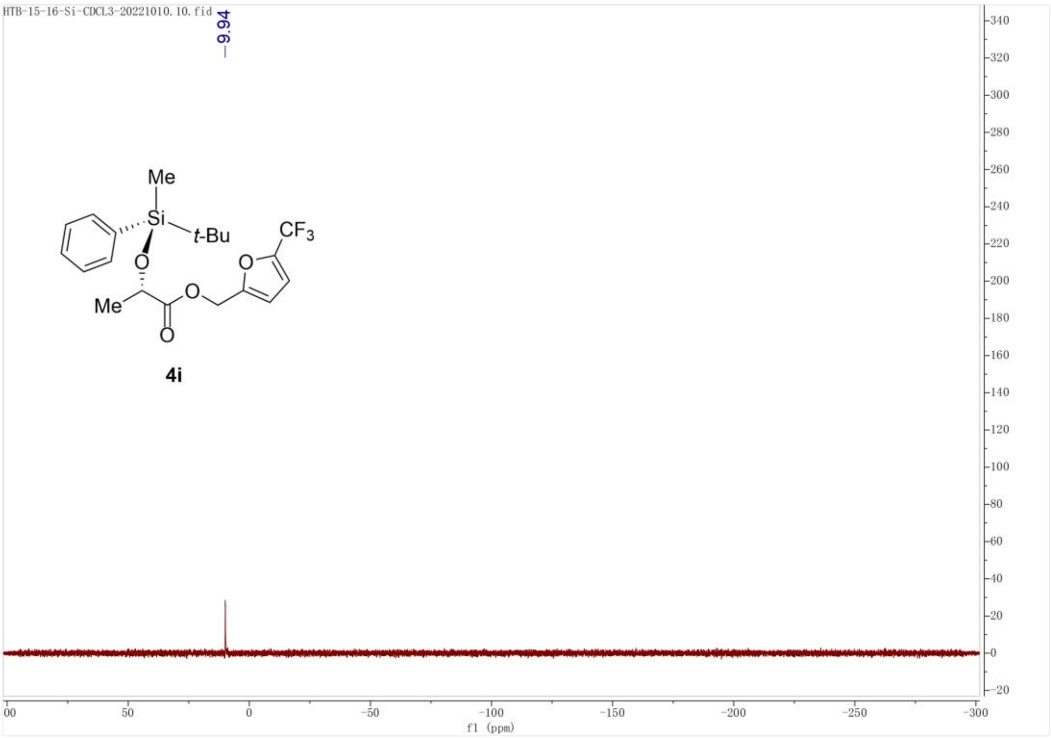

Supplementary Figure 75. ^{29}Si NMR (80 M, $CDCl_3$, 25 ^oC) of compound **4i**

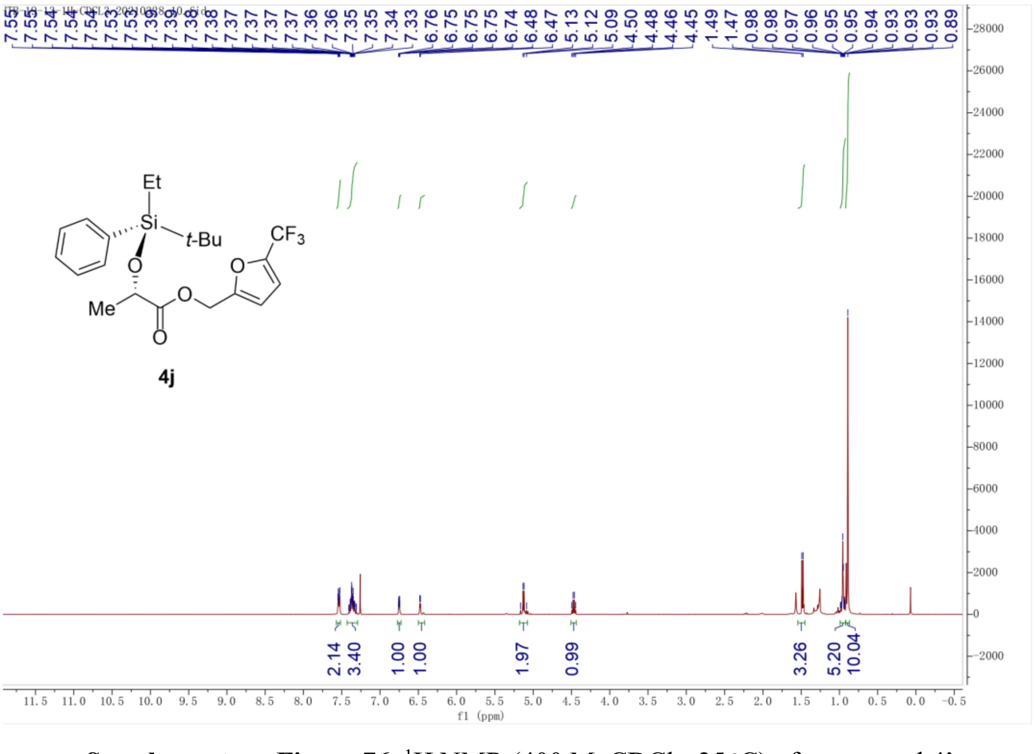

Supplementary Figure 76. ^{1}H NMR (400 M, $CDCl_3$, 25 ^oC) of compound **4j**

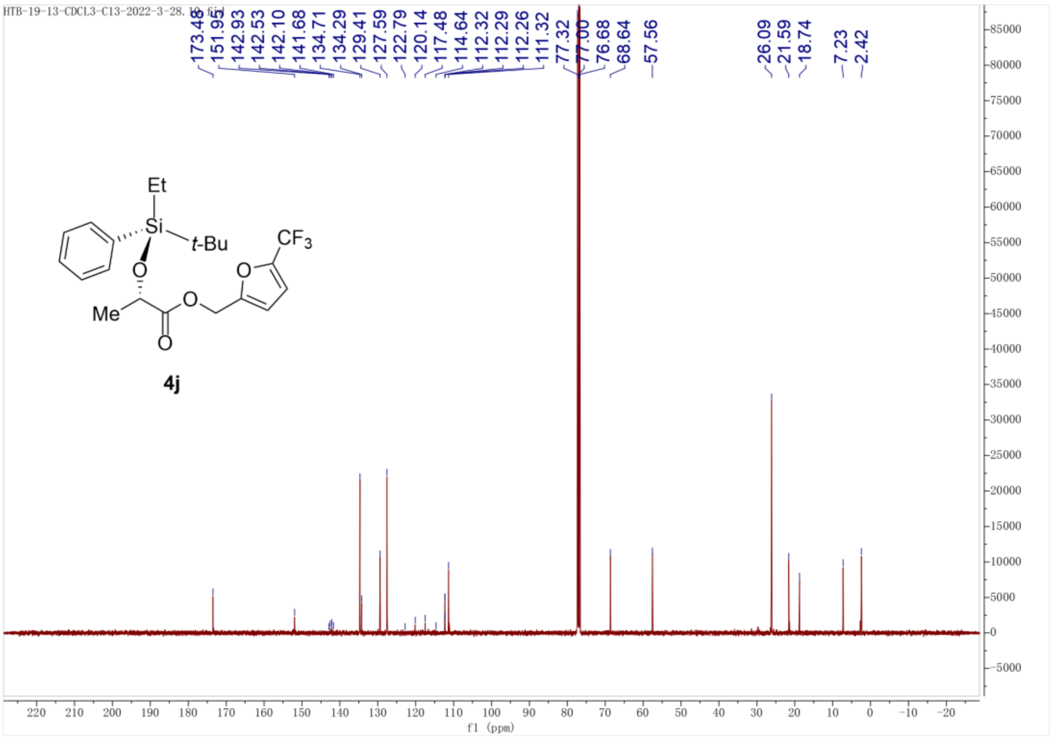

Supplementary Figure 77. ^{13}C NMR (100 M, $CDCl_3$, 25 °C) of compound **4j**

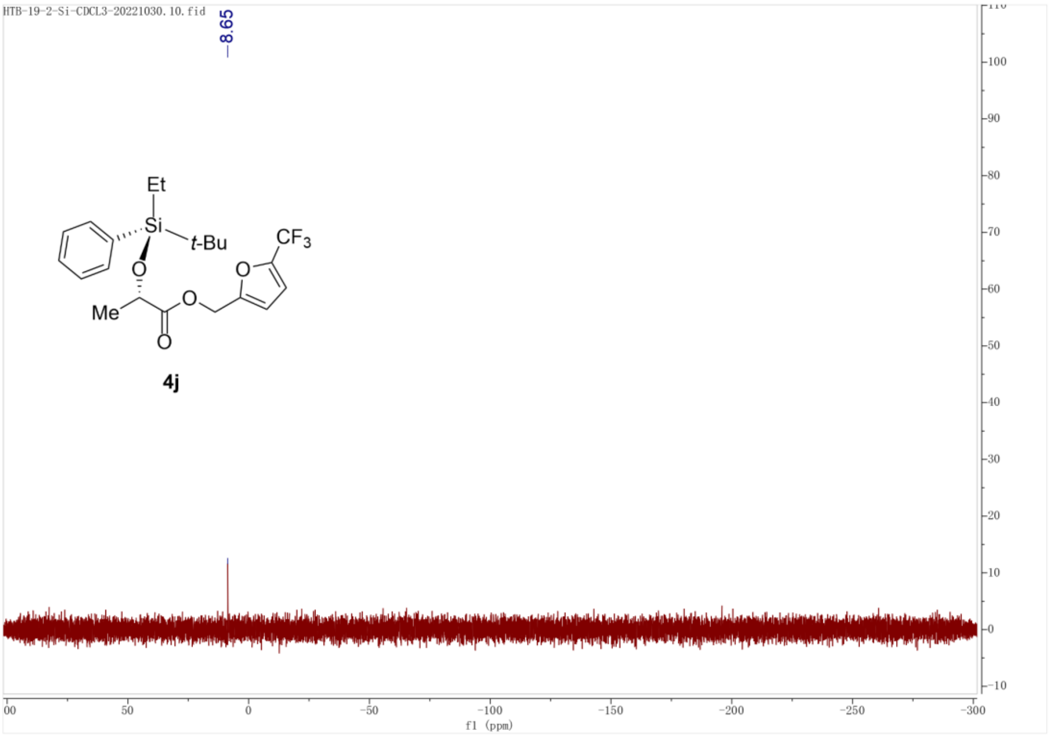

Supplementary Figure 78. ^{29}Si NMR (80 M, $CDCl_3$, 25 °C) of compound **4j**

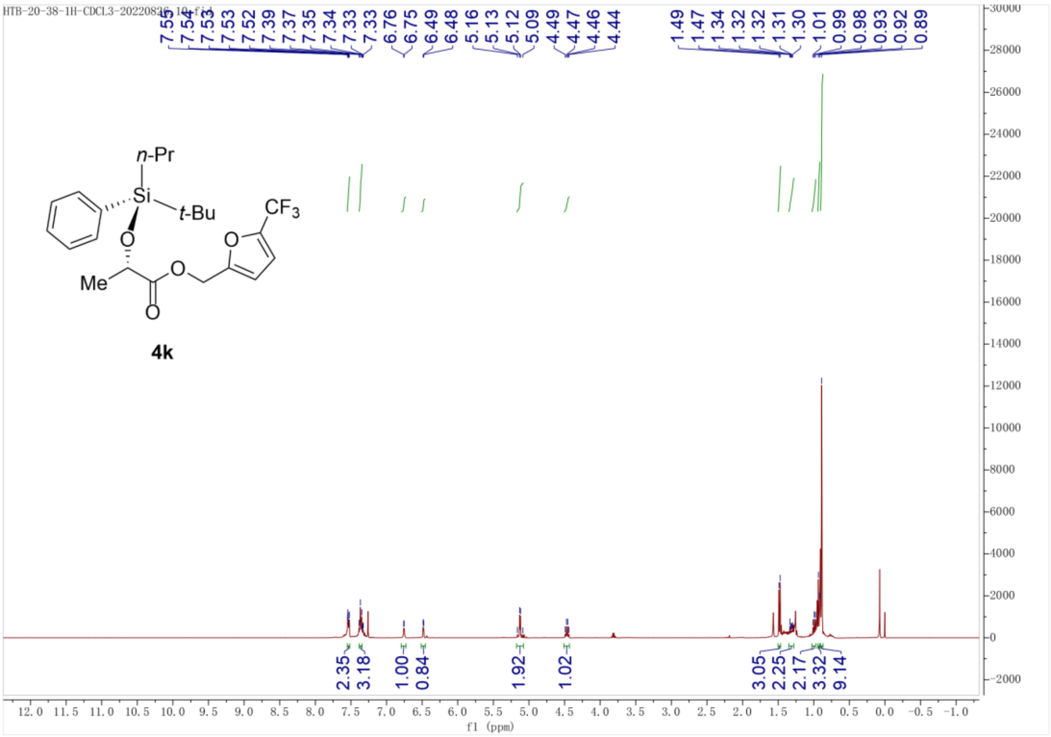

Supplementary Figure 79. ^{1}H NMR (400 M, $CDCl_3$, 25 °C) of compound **4k**

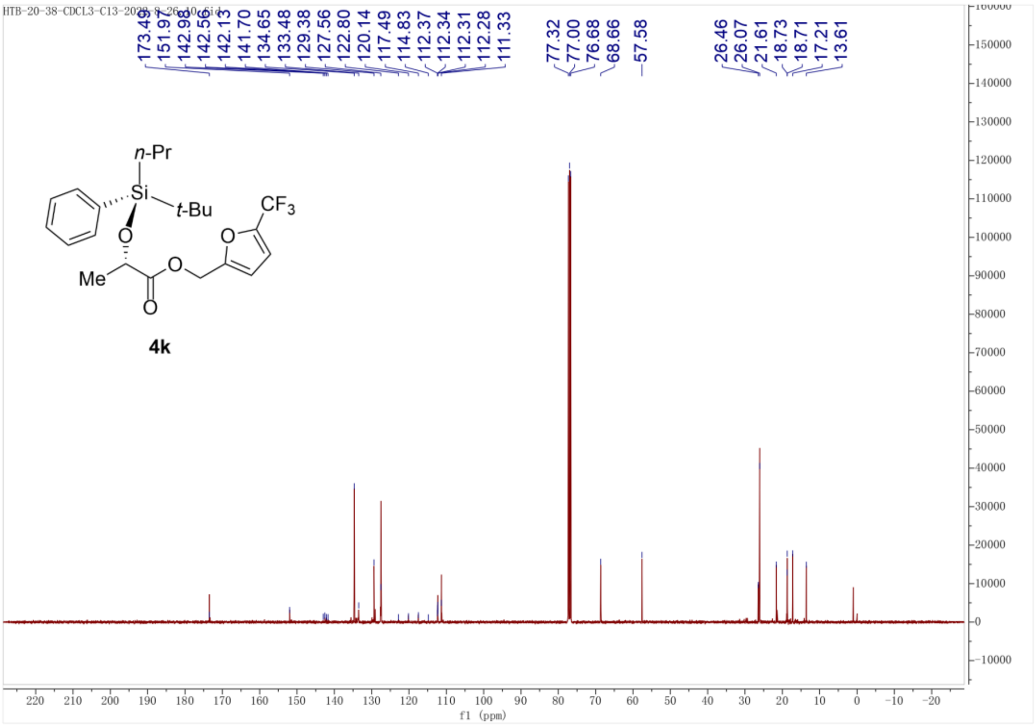

Supplementary Figure 80. ^{13}C NMR (100 M, $CDCl_3$, 25 °C) of compound **4k**

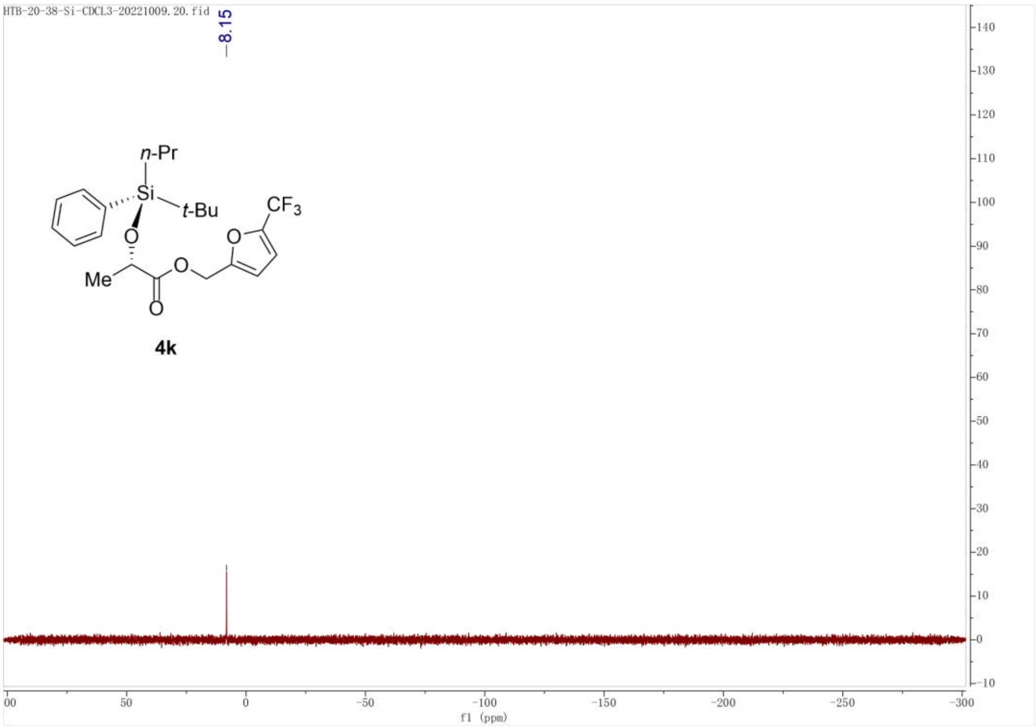

Supplementary Figure 81. ^{29}Si NMR (80 M, $CDCl_3$, 25 °C) of compound **4k**

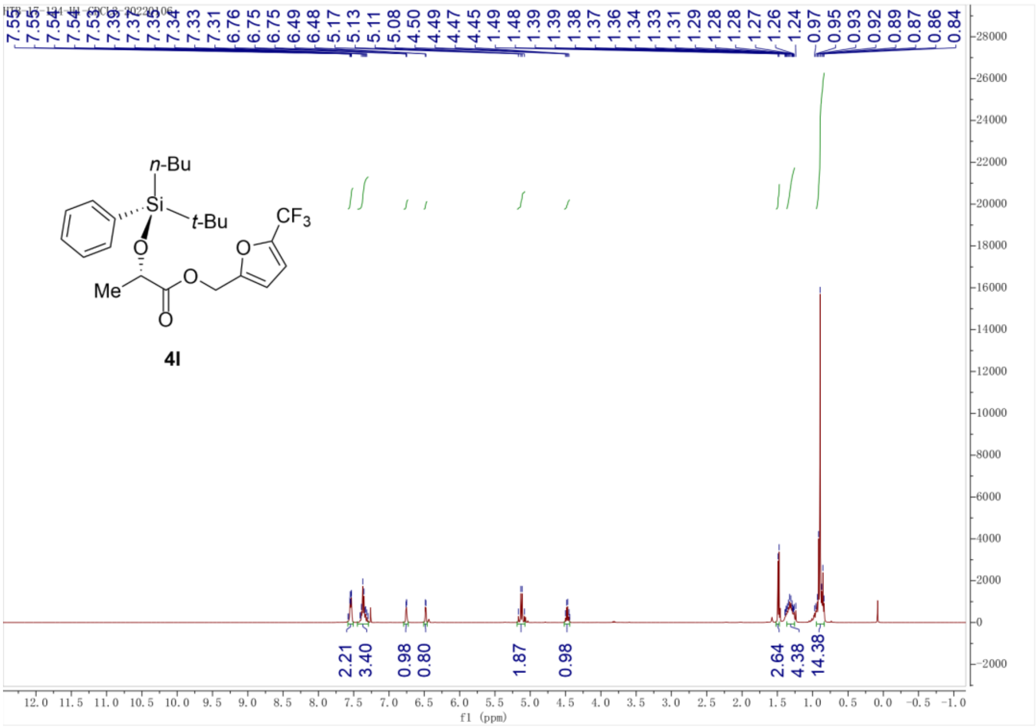

Supplementary Figure 82. ^{1}H NMR (400 M, $CDCl_3$, 25 °C) of compound **4l**

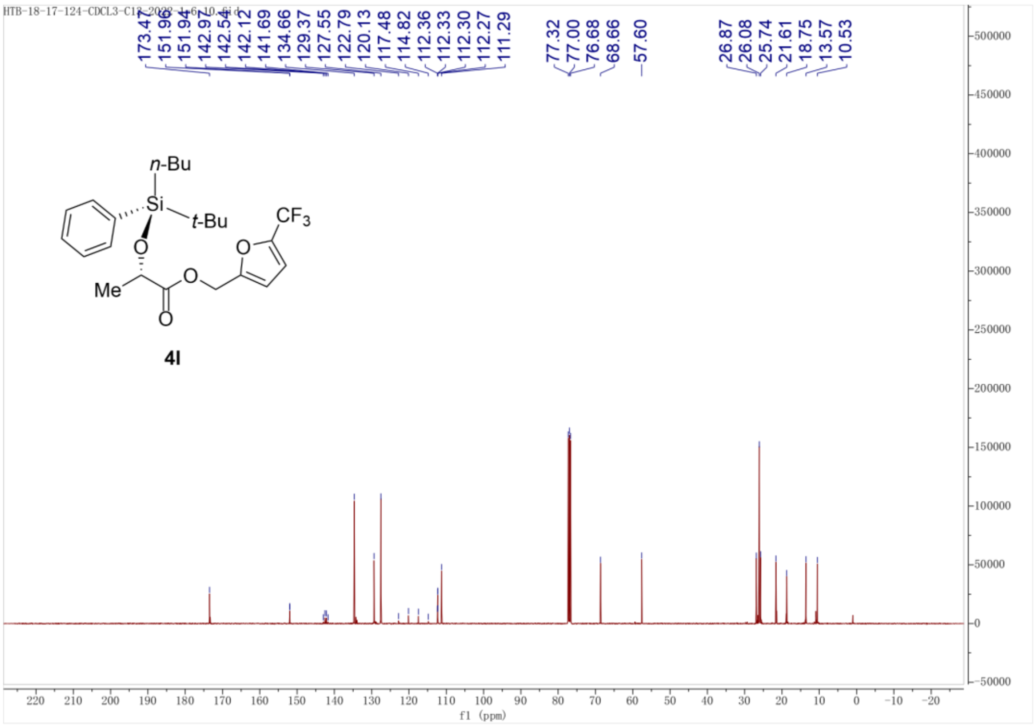

Supplementary Figure 83. ^{13}C NMR (100 M, $CDCl_3$, 25 °C) of compound **4l**

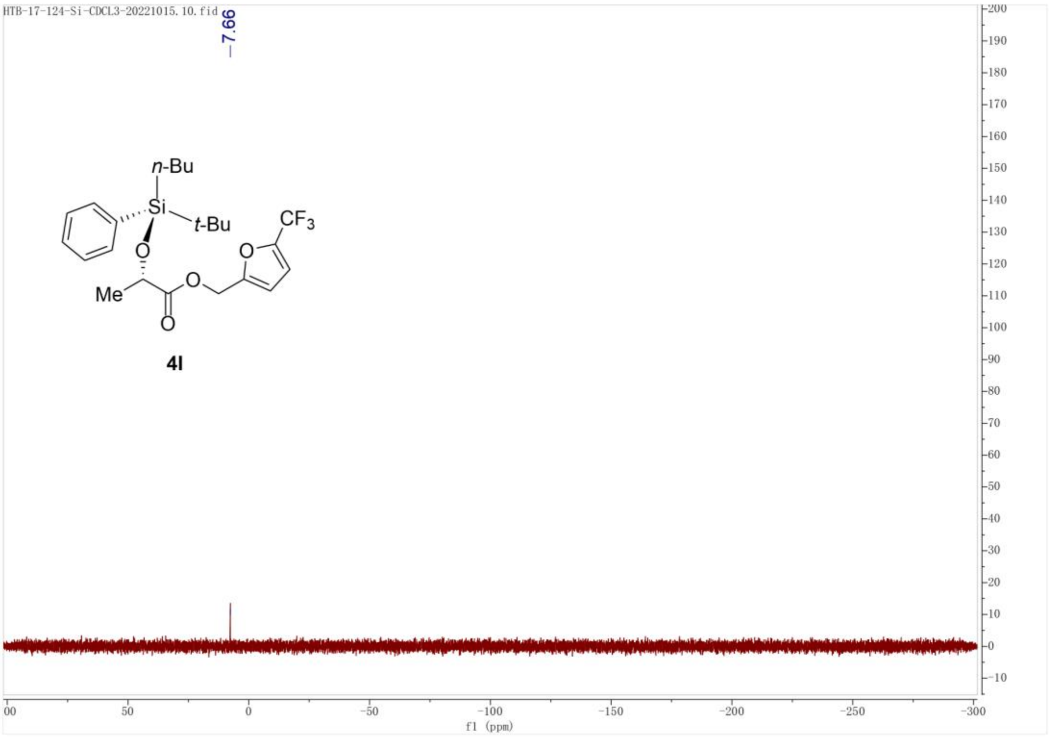

Supplementary Figure 84. ^{29}Si NMR (80 M, $CDCl_3$, 25 °C) of compound **4l**

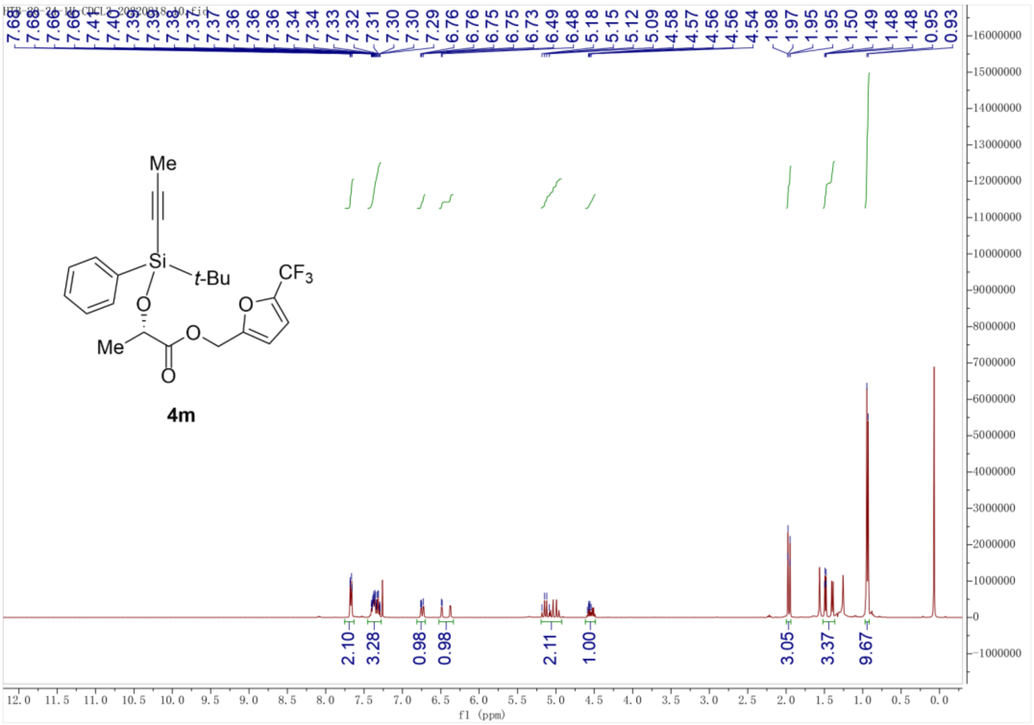

Supplementary Figure 85. ^{1}H NMR (400 M, $CDCl_3$, 25 ^{o}C) of compound **4m**

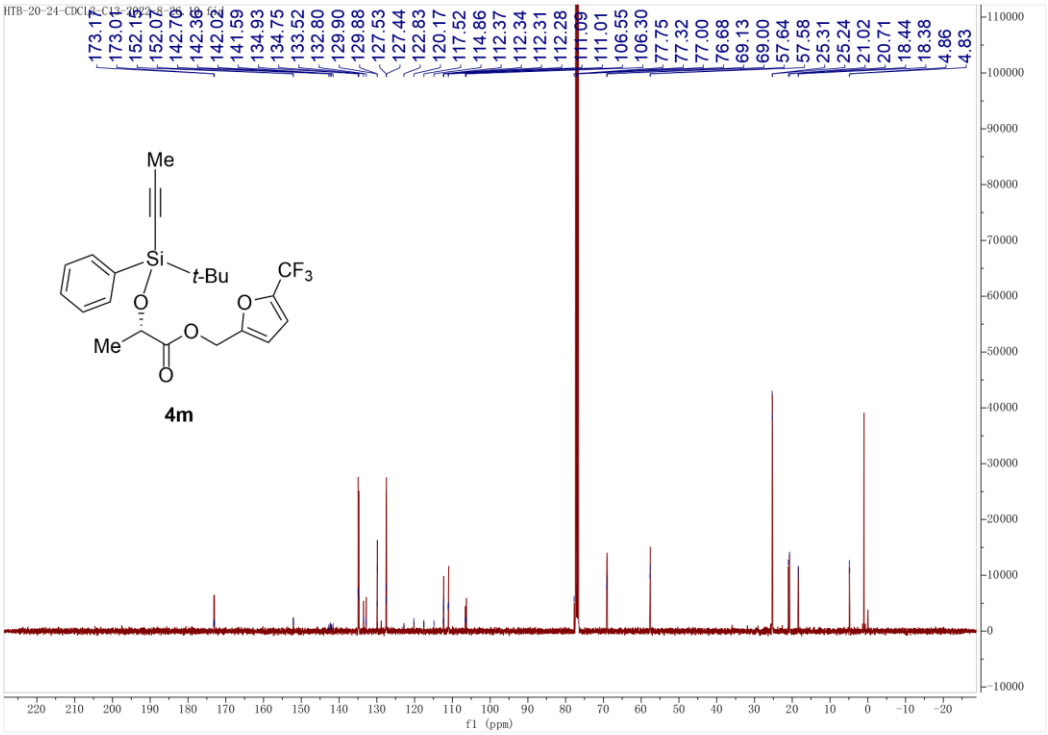

Supplementary Figure 86. ^{13}C NMR (100 M, $CDCl_3$, 25 ^{o}C) of compound **4m**

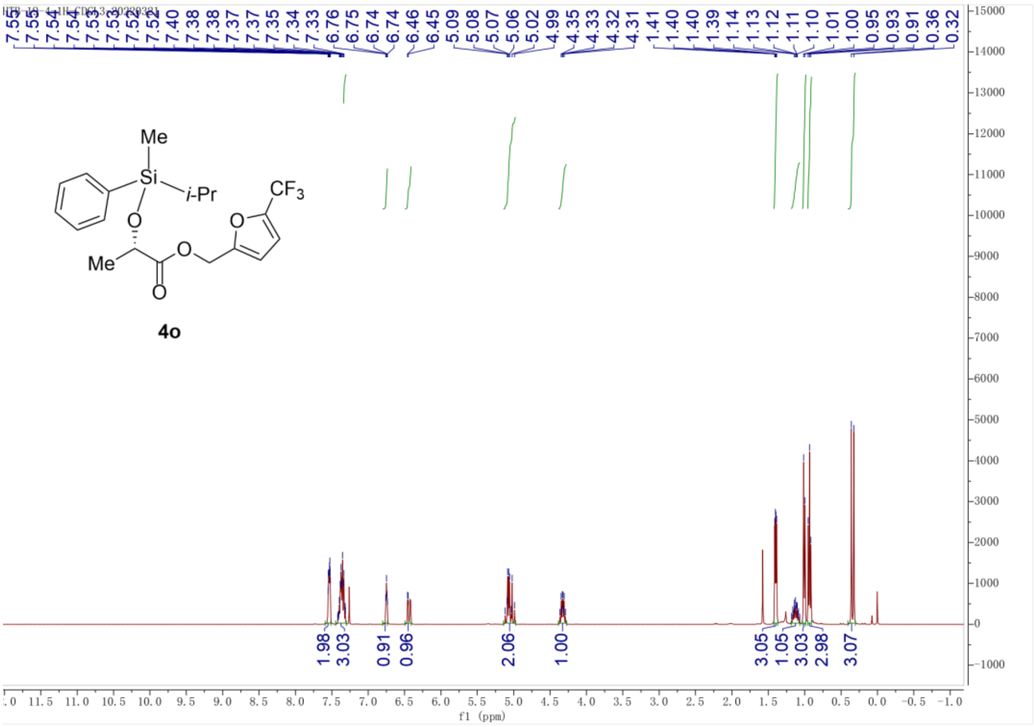

Supplementary Figure 87. ^{1}H NMR (400 M, $CDCl_3$, 25 °C) of compound **4o**

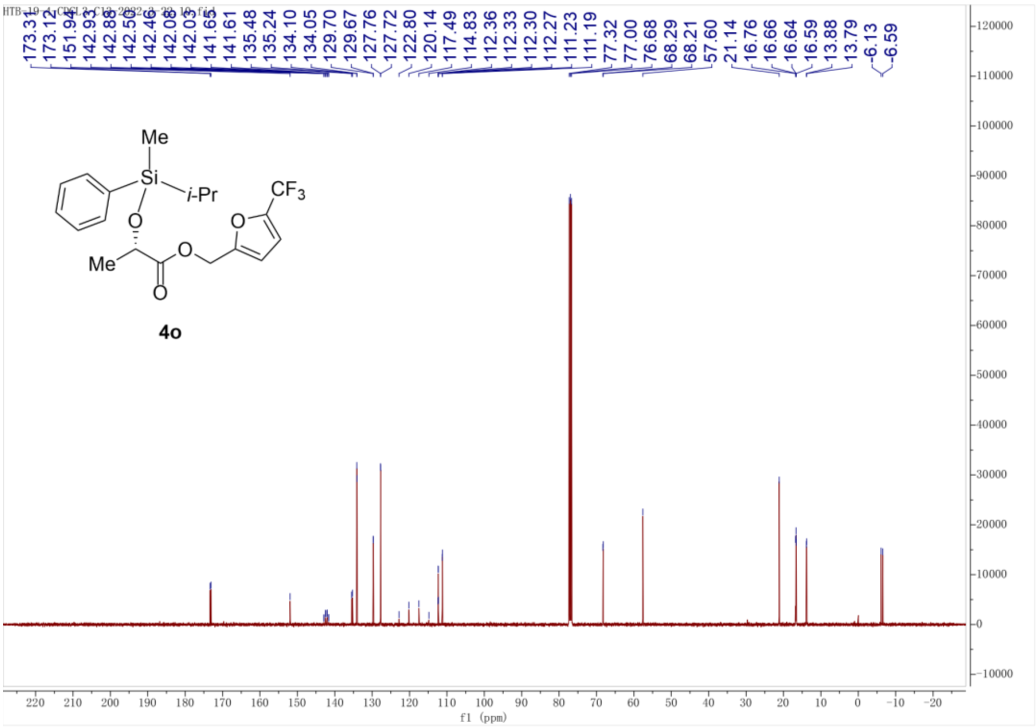

Supplementary Figure 88. ^{13}C NMR (100 M, $CDCl_3$, 25 °C) of compound **4o**

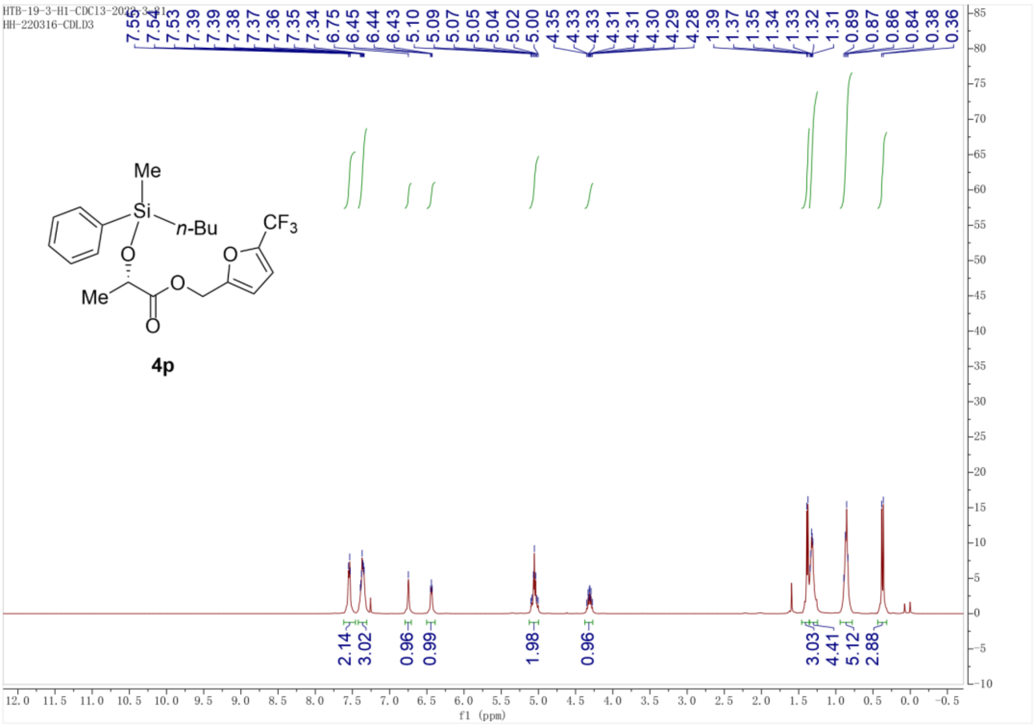

Supplementary Figure 89. ^{1}H NMR (400 M, $CDCl_3$, 25 °C) of compound **4p**

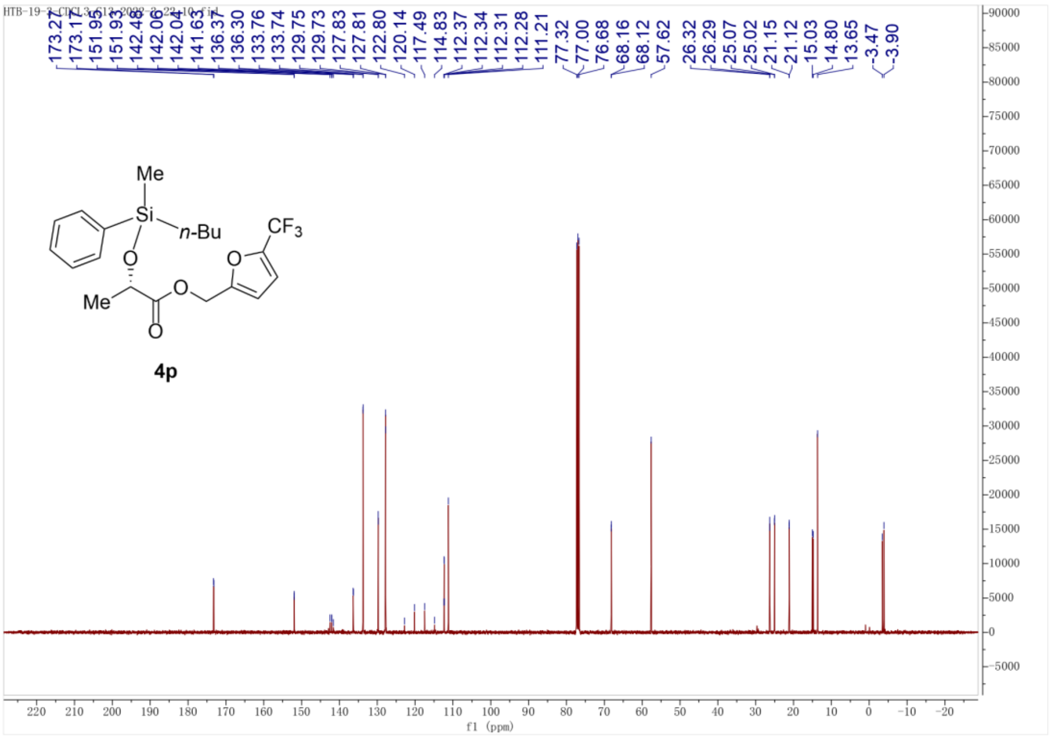

Supplementary Figure 90. ^{13}C NMR (100 M, $CDCl_3$, 25 °C) of compound **4p**

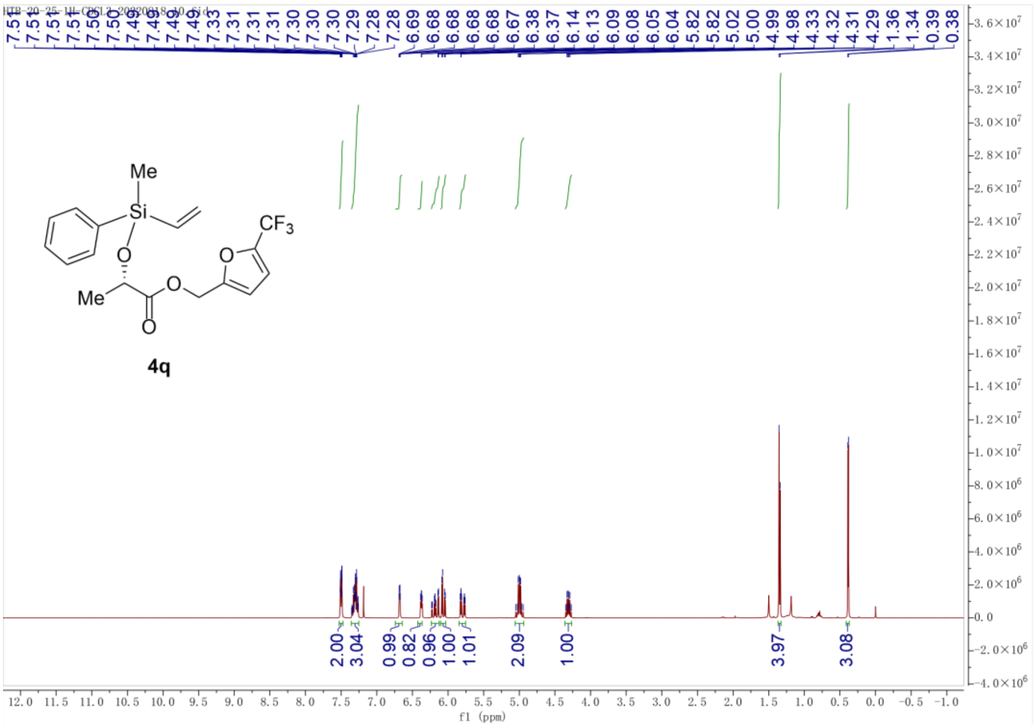

Supplementary Figure 91. ^{1}H NMR (400 M, $CDCl_3$, 25 °C) of compound **4q**

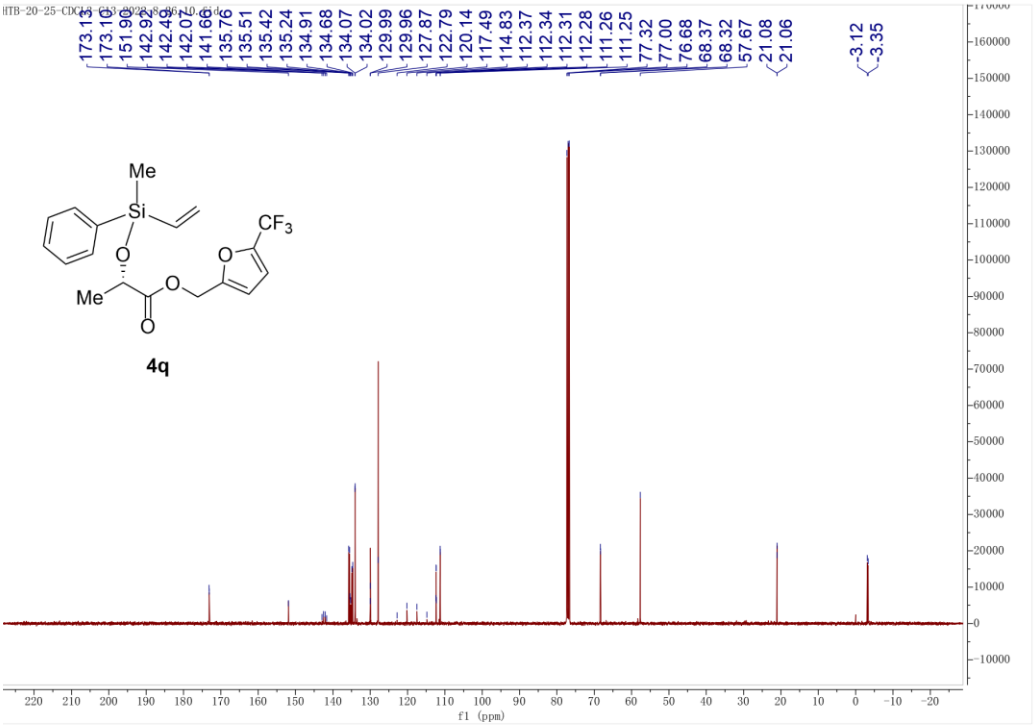

Supplementary Figure 92. ^{13}C NMR (100 M, $CDCl_3$, 25 °C) of compound **4q**

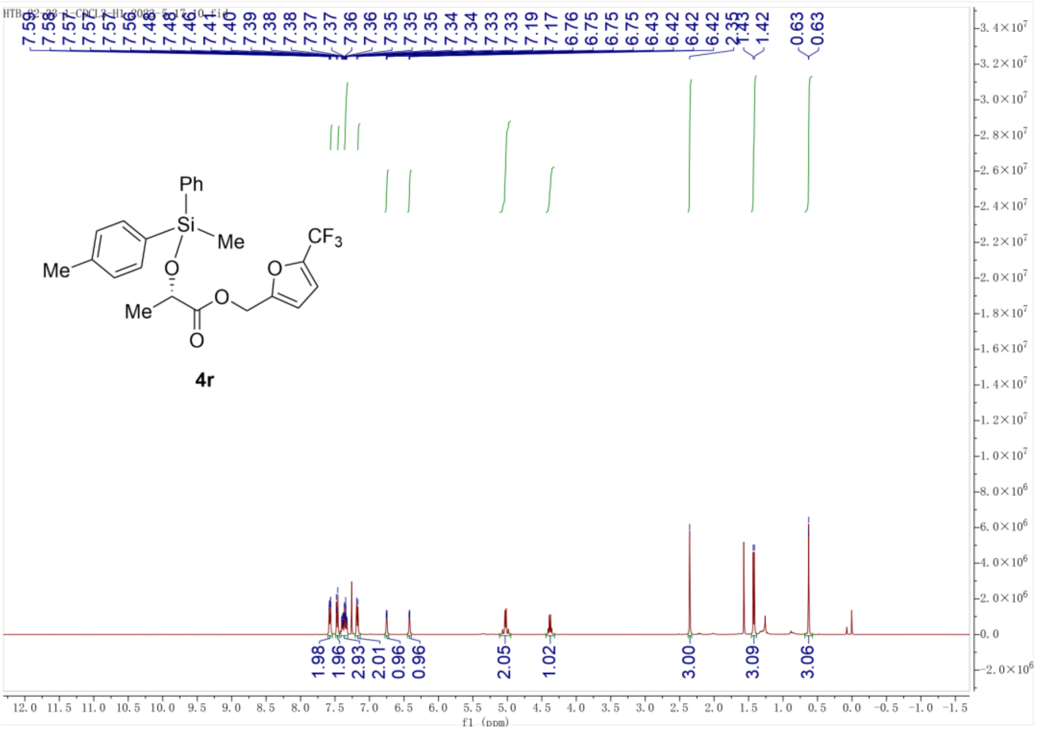

Supplementary Figure 93. ^{1}H NMR (400 M, $CDCl_3$, 25 ^{o}C) of compound **4r**

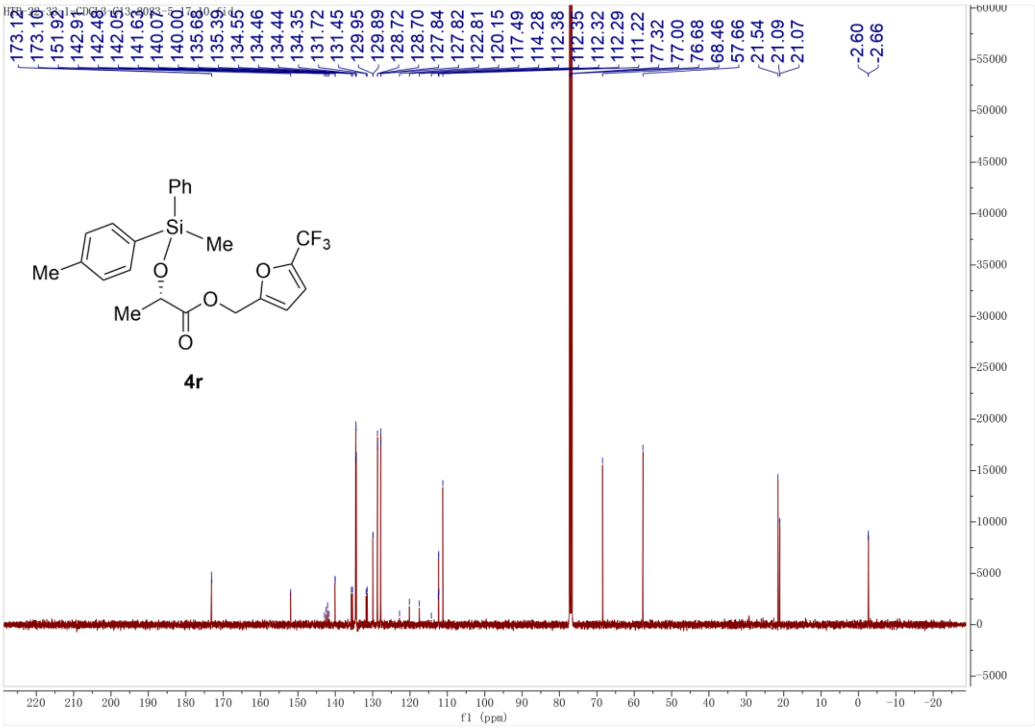

Supplementary Figure 94. ^{13}C NMR (100 M, $CDCl_3$, 25 ^{o}C) of compound **4r**

\

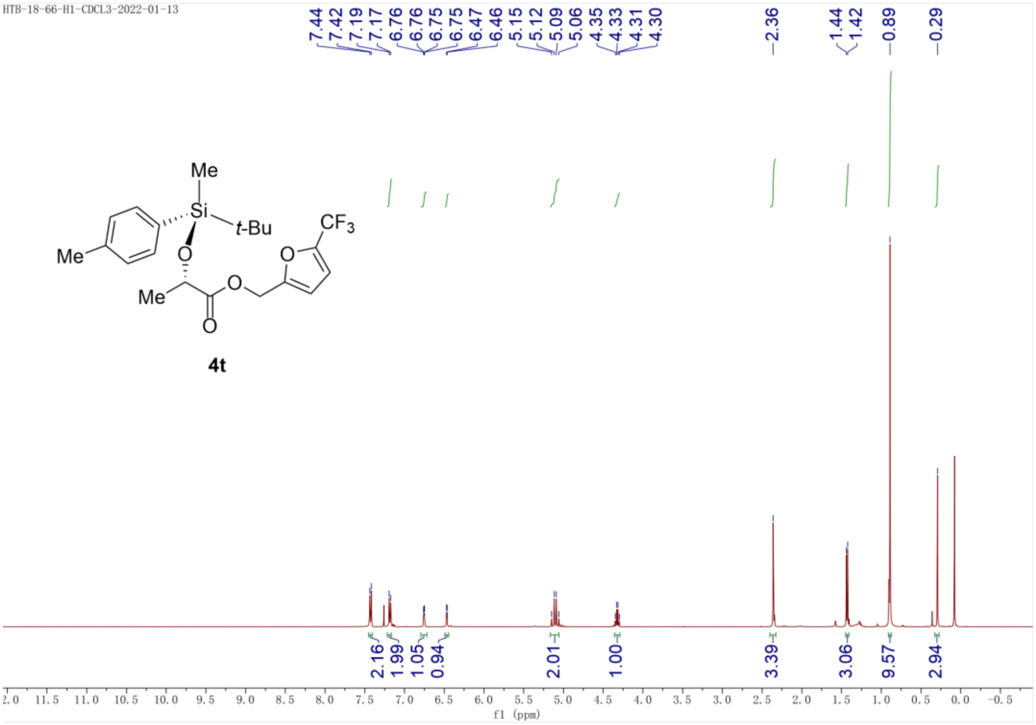

Supplementary Figure 95. ^{1}H NMR (400 M, $CDCl_3$, 25 °C) of compound **4t**

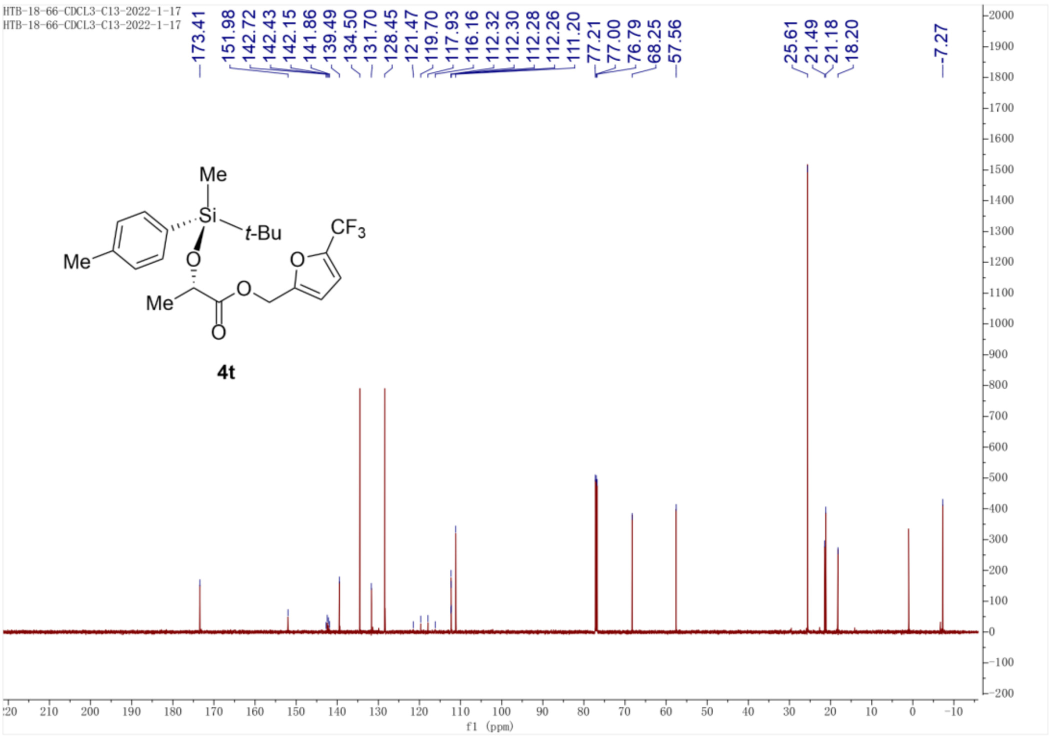

Supplementary Figure 96. ^{13}C NMR (100 M, $CDCl_3$, 25 °C) of compound **4t**

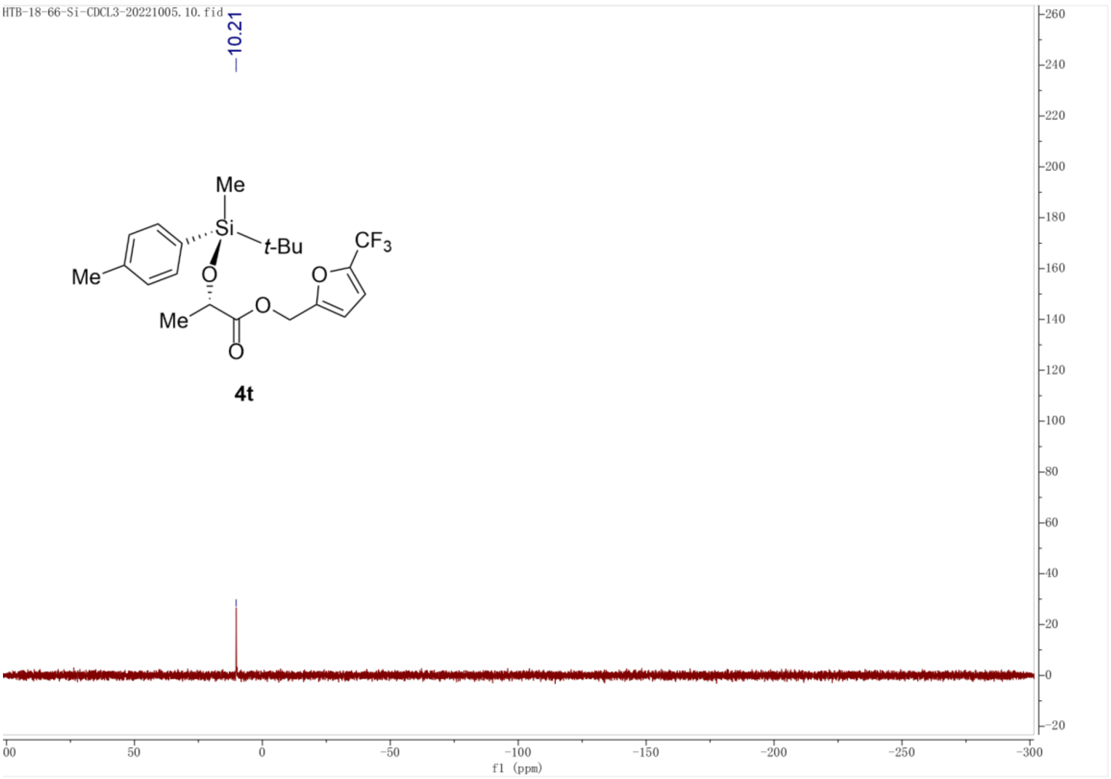

Supplementary Figure 97. ^{29}Si NMR (80 M, $CDCl_3$, 25 ^{o}C) of compound **4t**

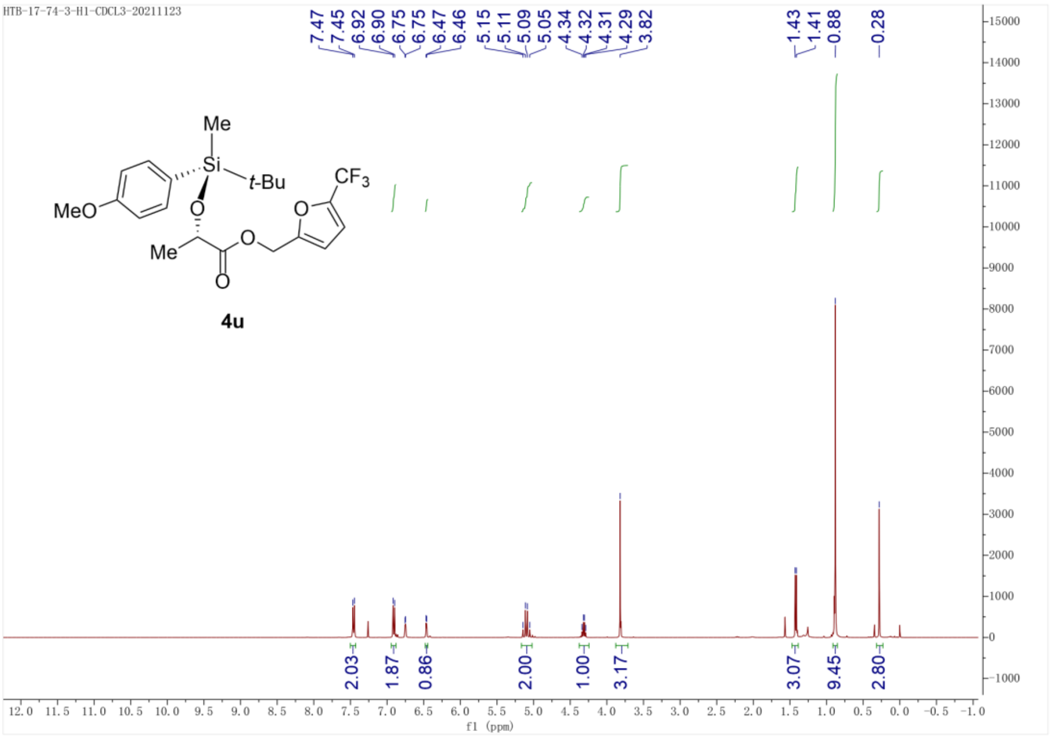

Supplementary Figure 98. ^{1}H NMR (400 M, $CDCl_3$, 25 ^{o}C) of compound **4u**

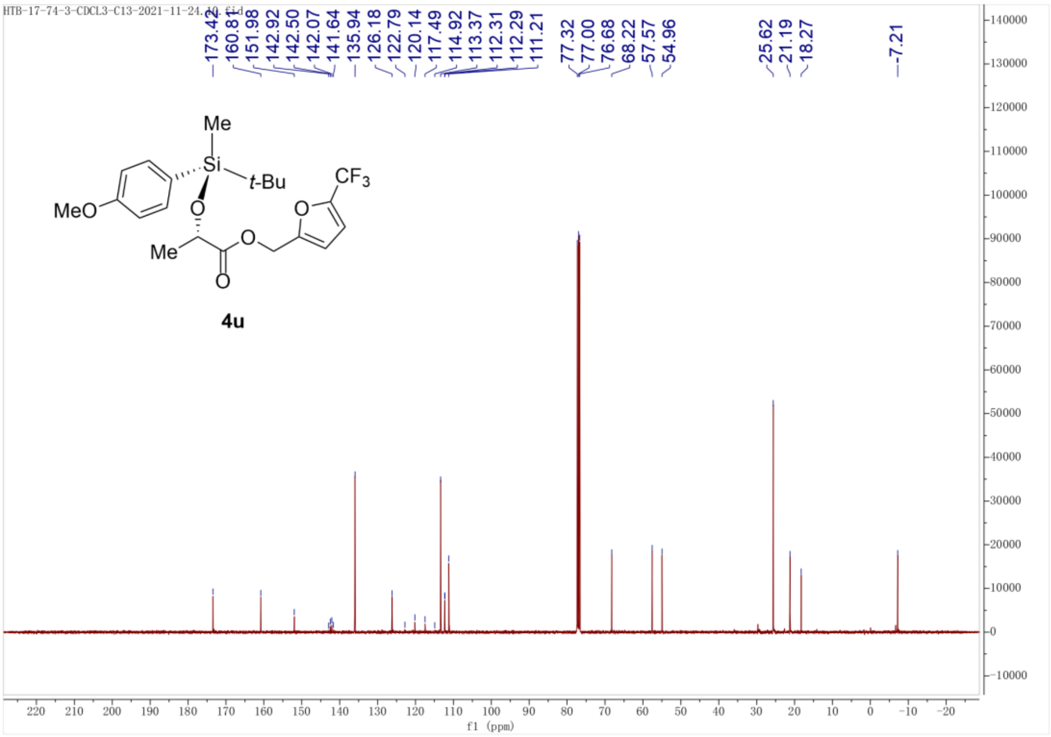

Supplementary Figure 99. ^{13}C NMR (100 M, $CDCl_3$, 25 °C) of compound **4u**

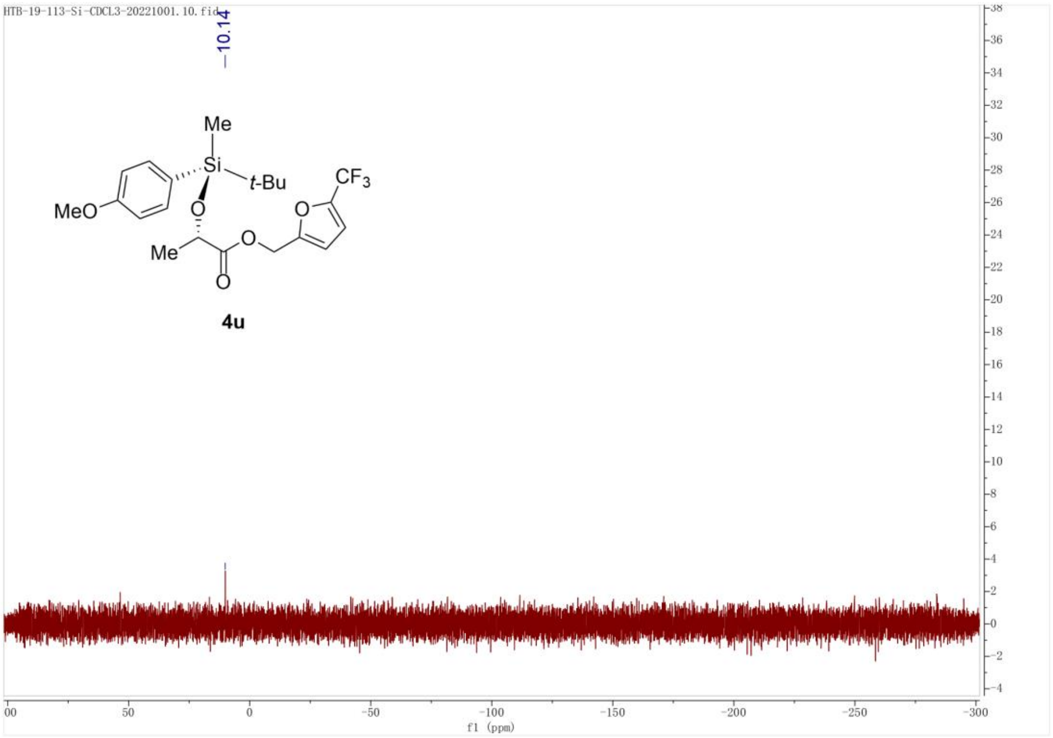

Supplementary Figure 100. ^{29}Si NMR (80 M, $CDCl_3$, 25 °C) of compound **4u**

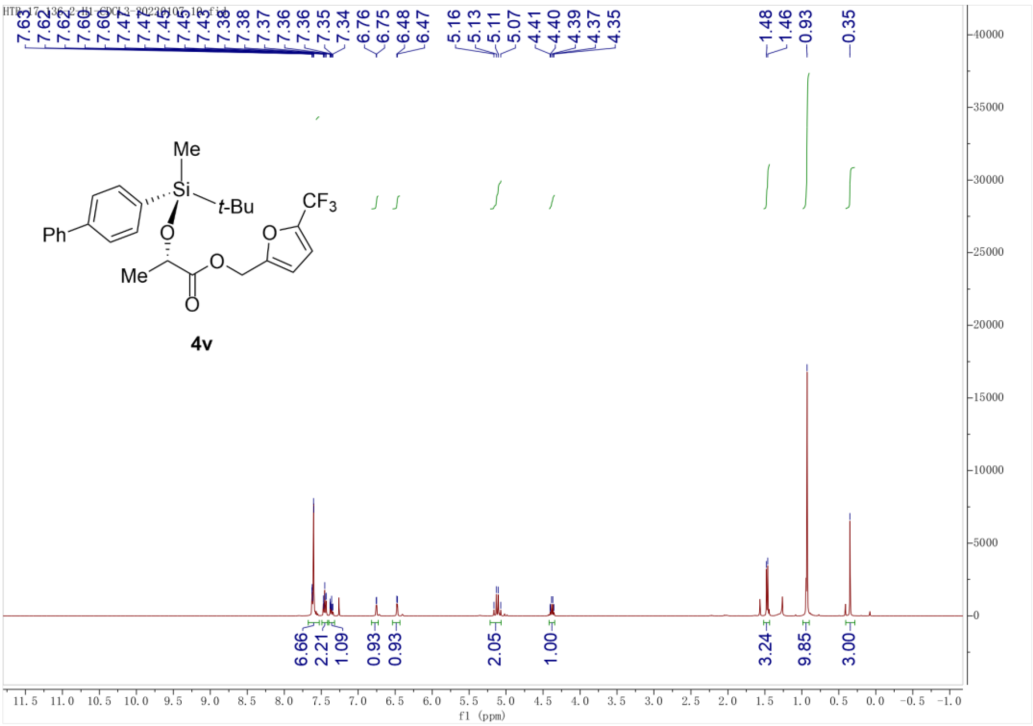

Supplementary Figure 101. ^1H NMR (400 M, $CDCl_3$, 25 °C) of compound **4v**

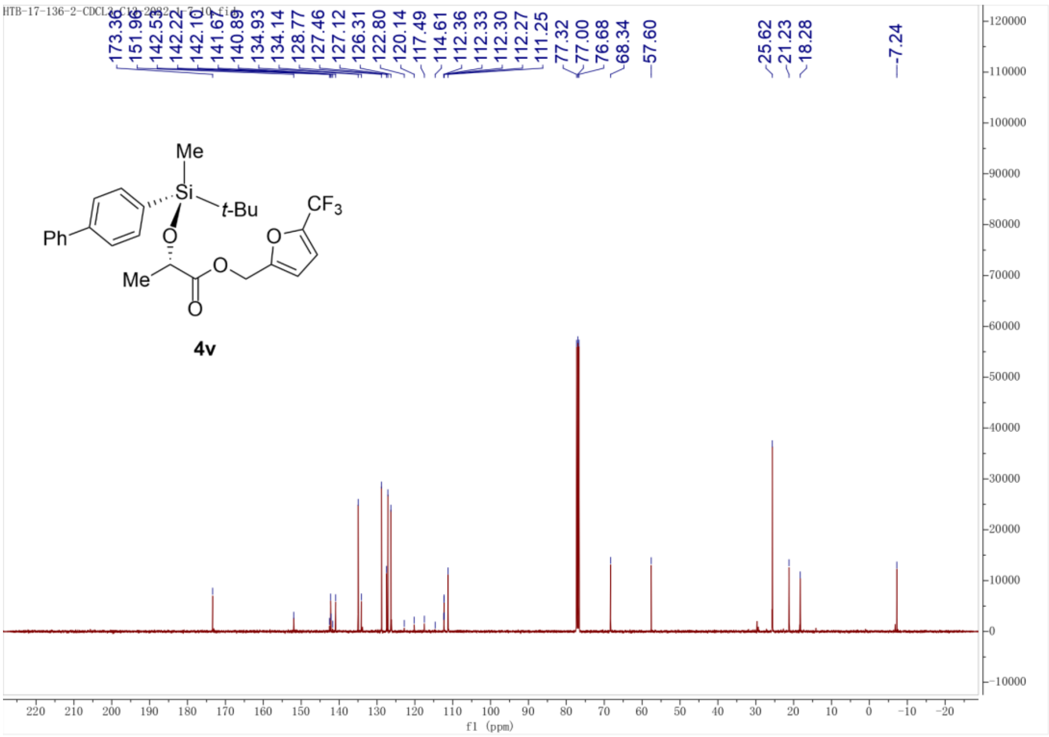

Supplementary Figure 102. ^{13}C NMR (100 M, $CDCl_3$, 25 °C) of compound **4v**

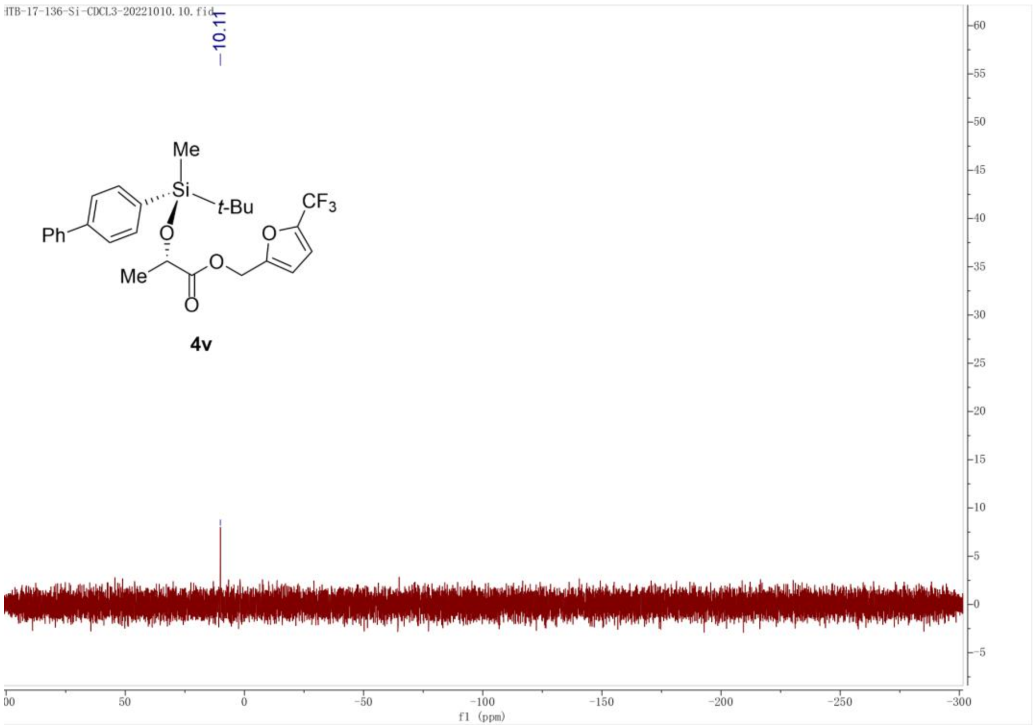

Supplementary Figure 103. ^{29}Si NMR (80 M, $CDCl_3$, 25 °C) of compound **4v**

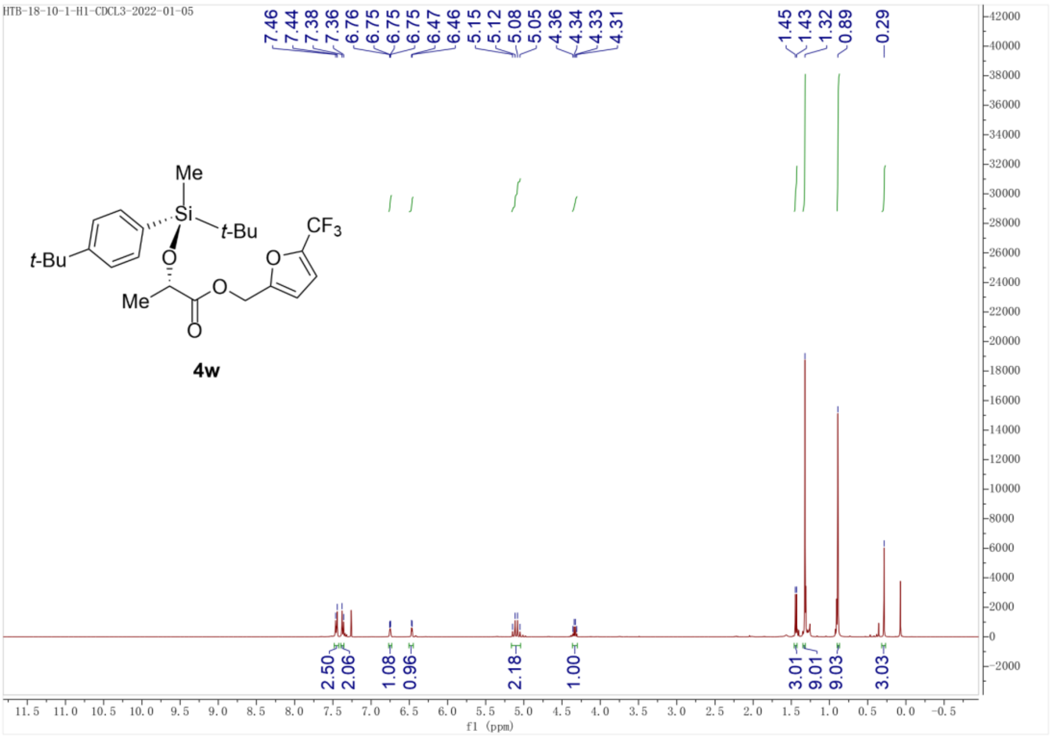

Supplementary Figure 104. ^{1}H NMR (400 M, $CDCl_3$, 25 °C) of compound **4w**

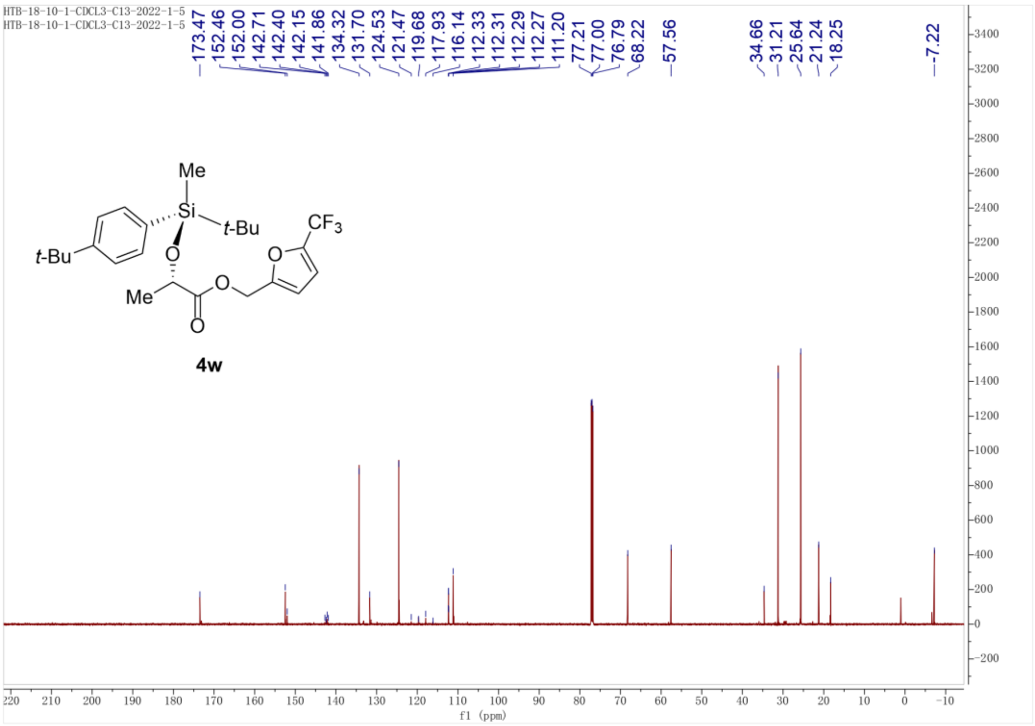

Supplementary Figure 105. ^{13}C NMR (100 M, $CDCl_3$, 25 °C) of compound **4w**

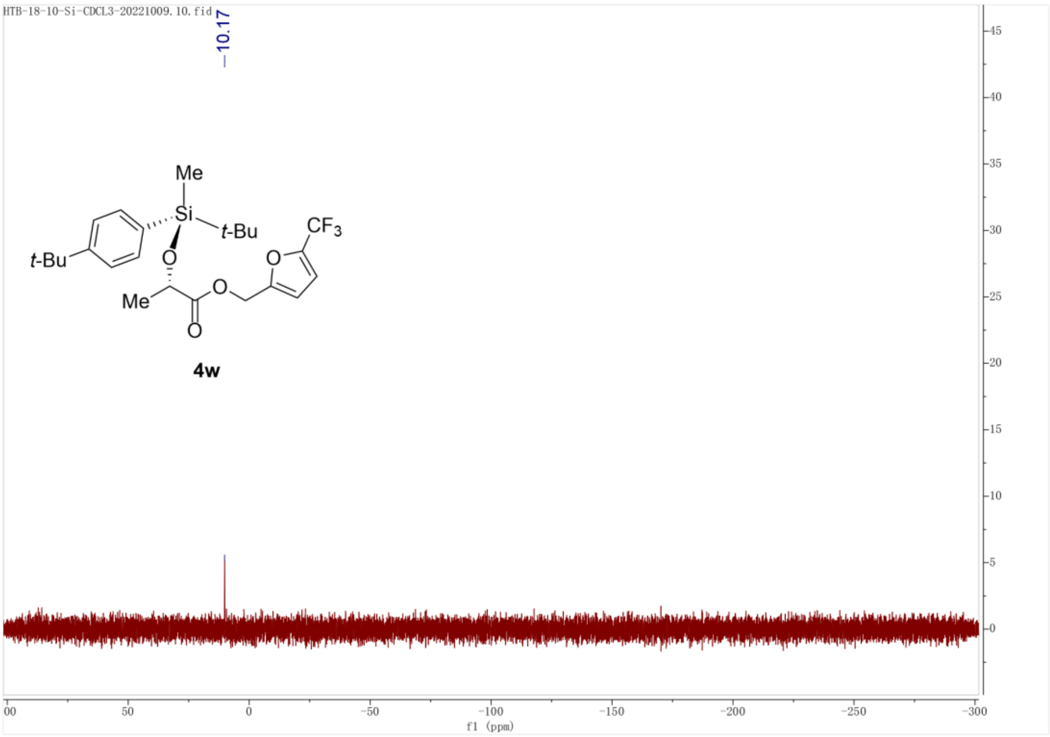

Supplementary Figure 106. ^{29}Si NMR (80 M, $CDCl_3$, 25 °C) of compound **4w**

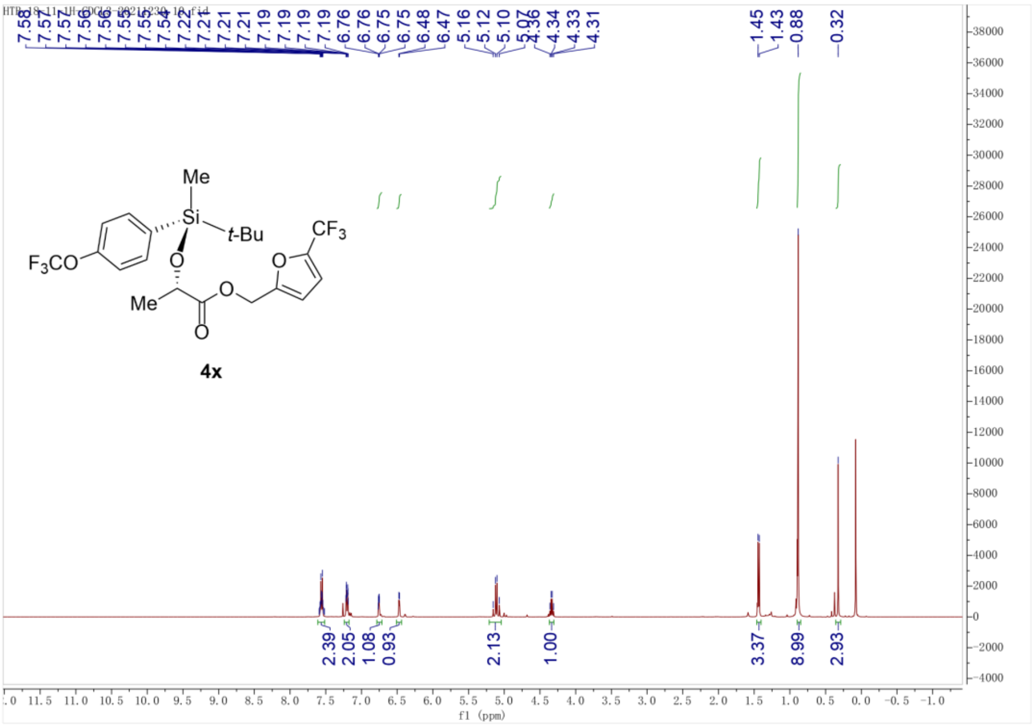

Supplementary Figure 107. ^{1}H NMR (400 M, $CDCl_3$, 25 °C) of compound **4x**

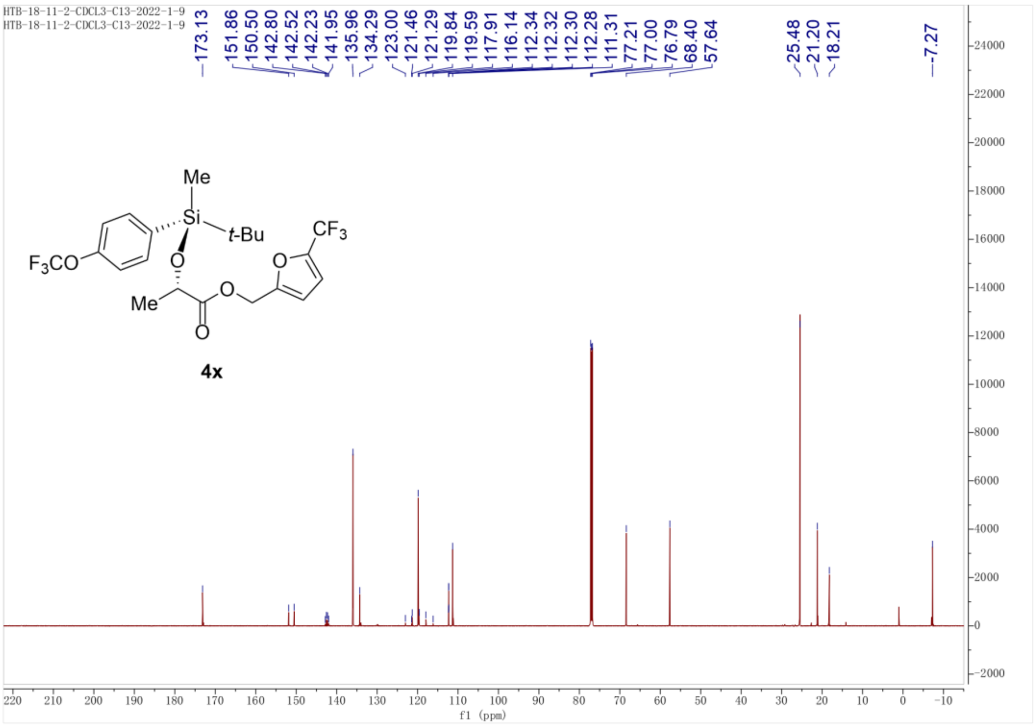

Supplementary Figure 108. ^{13}C NMR (150 M, $CDCl_3$, 25 °C) of compound **4x**

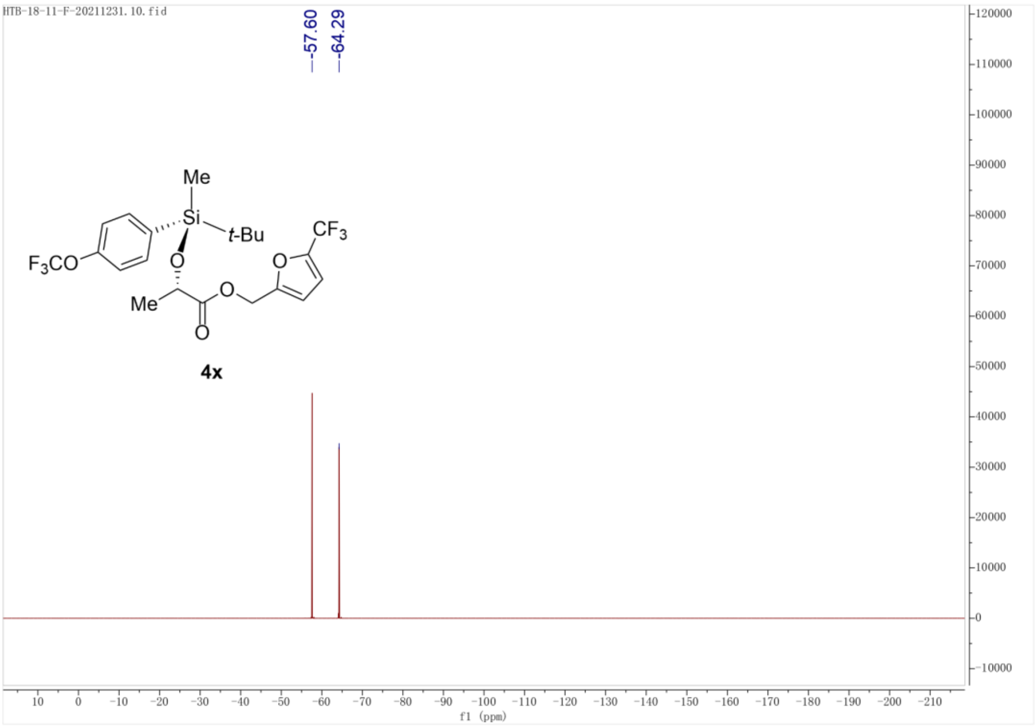

Supplementary Figure 109. ^{19}F NMR (376 M, $CDCl_3$, 25 °C) of compound **4x**

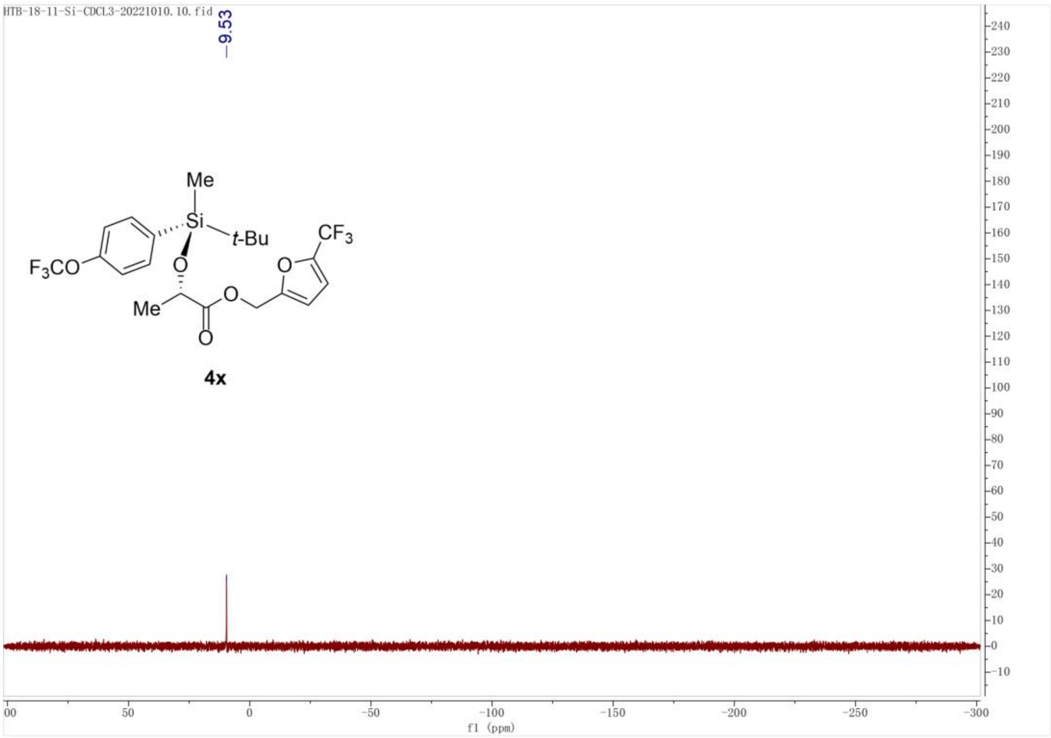

Supplementary Figure 110. ^{29}Si NMR (80 M, $CDCl_3$, 25 °C) of compound **4x**

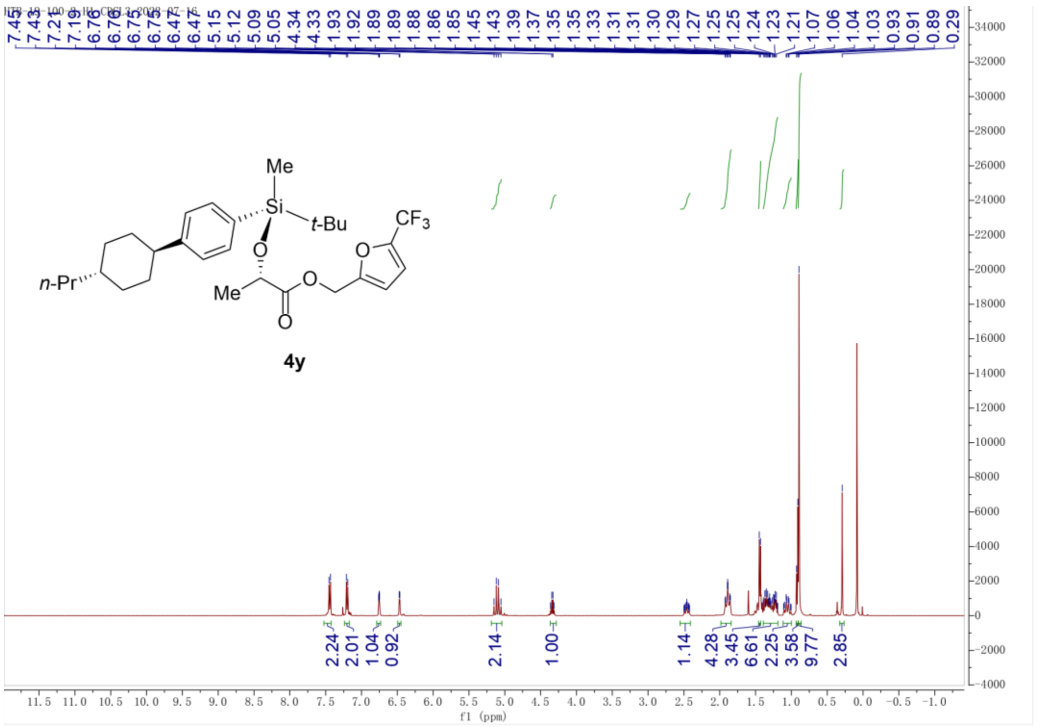

Supplementary Figure 111. ^{1}H NMR (400 M, $CDCl_3$, 25 °C) of compound **4y**

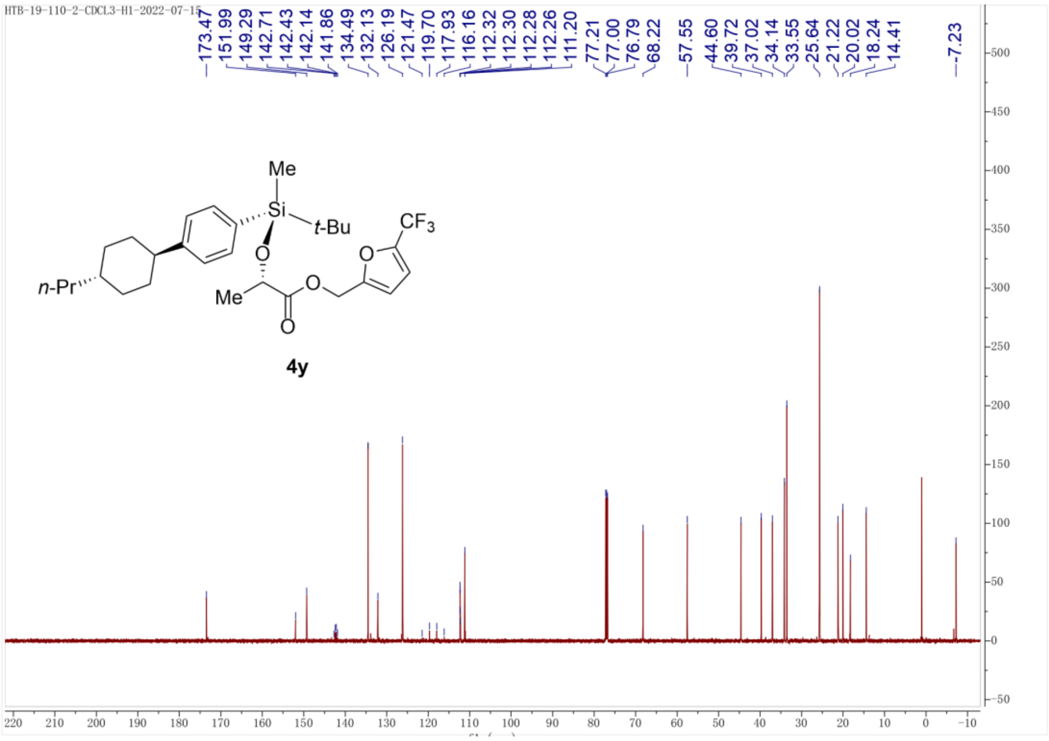

Supplementary Figure 112. ^{13}C NMR (150 M, $CDCl_3$, 25 °C) of compound **4y**

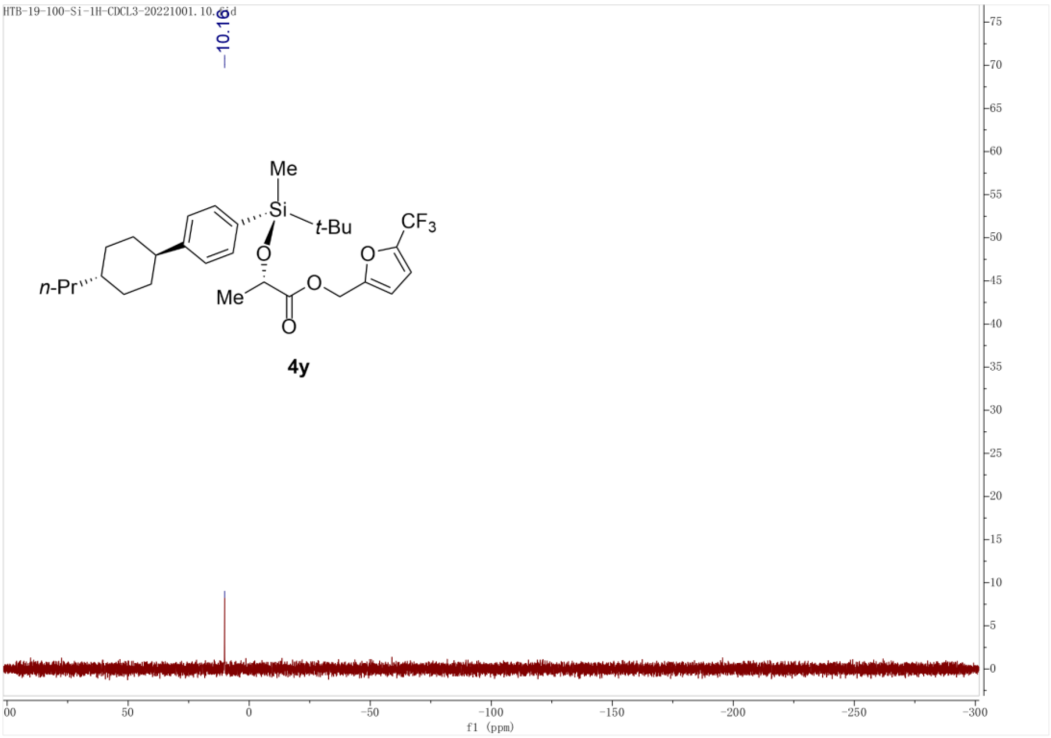

Supplementary Figure 113. ^{29}Si NMR (80 M, $CDCl_3$, 25 ^{o}C) of compound **4y**

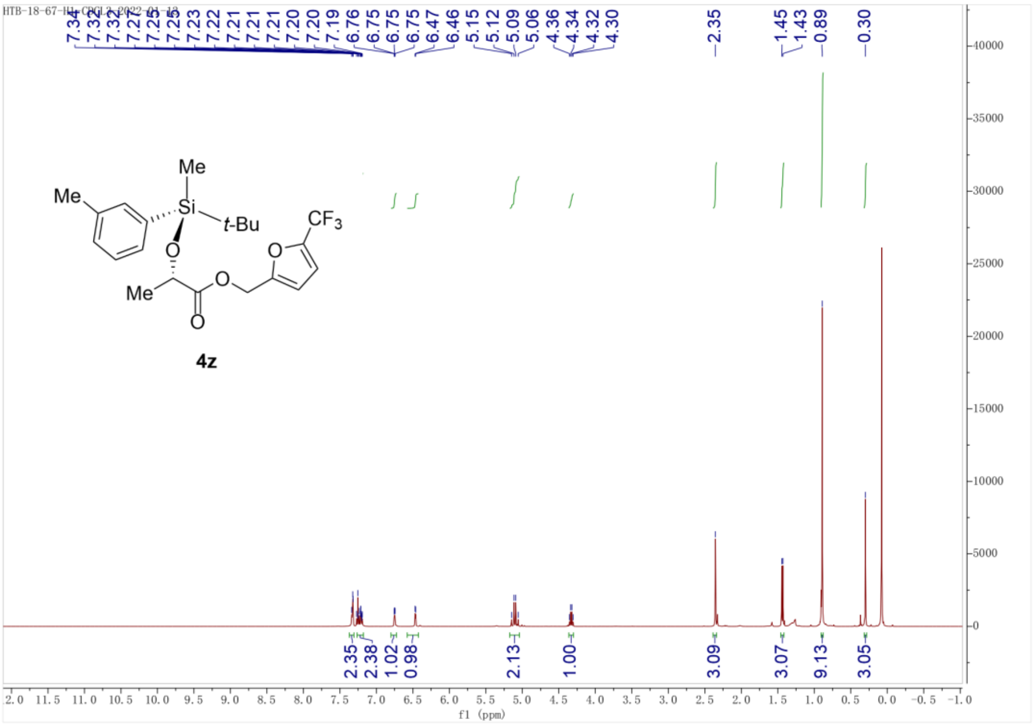

Supplementary Figure 114. ^{1}H NMR (400 M, $CDCl_3$, 25 ^{o}C) of compound **4z**

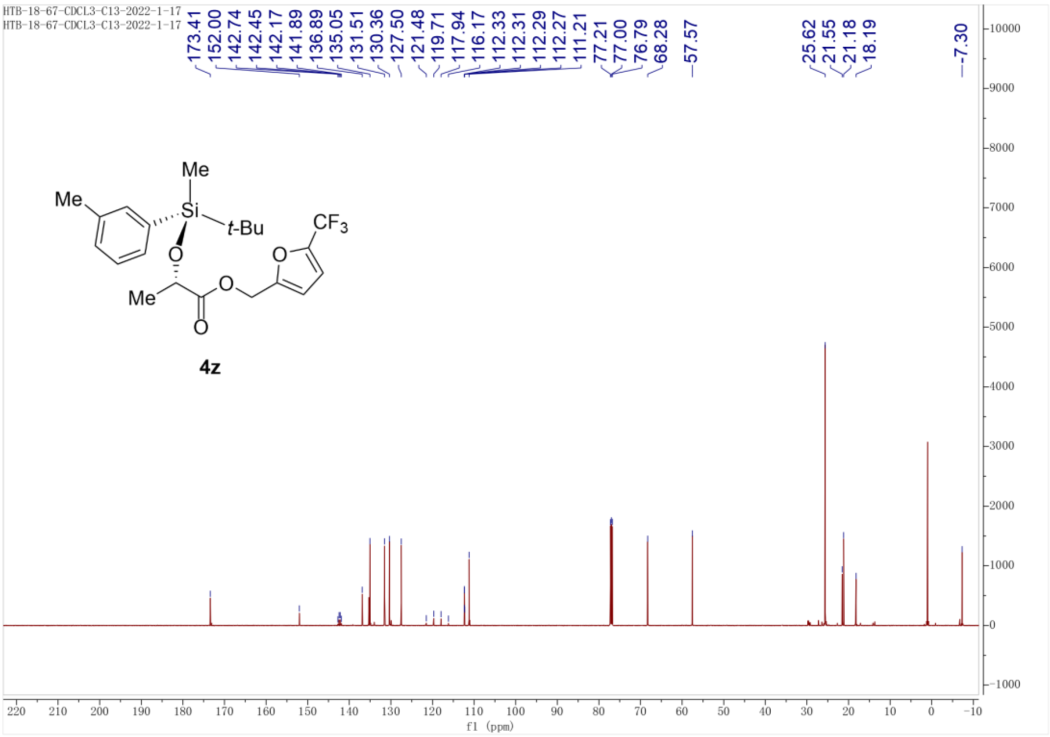

Supplementary Figure 115. ^{13}C NMR (150 M, $CDCl_3$, 25 ^{o}C) of compound **4z**

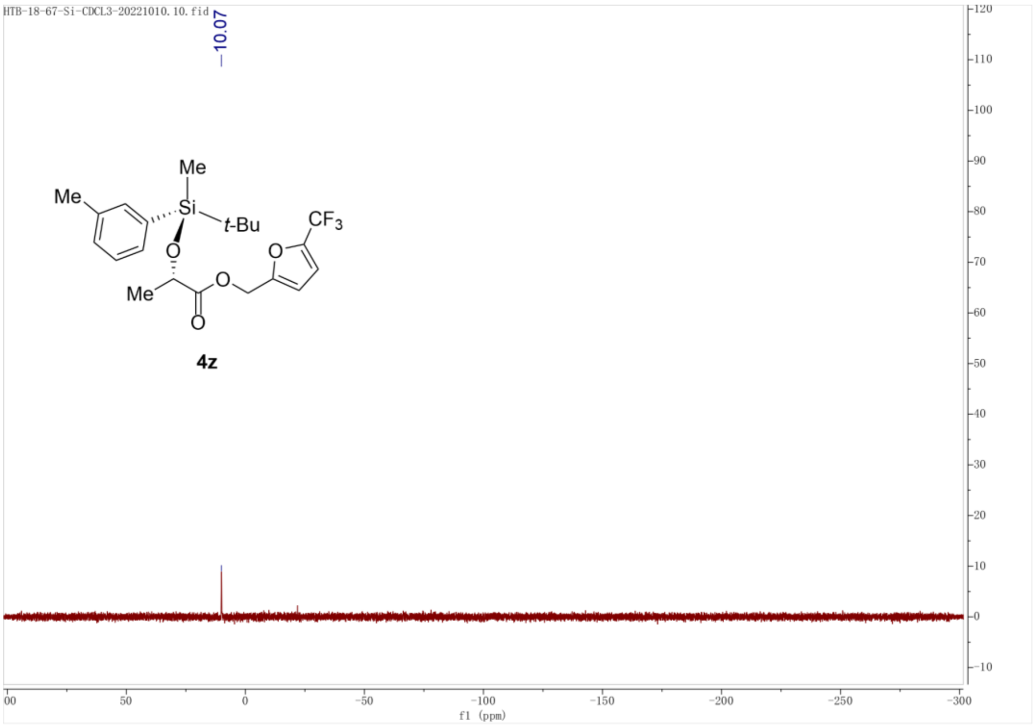

Supplementary Figure 116. ^{29}Si NMR (80 M, $CDCl_3$, 25 ^{o}C) of compound **4z**

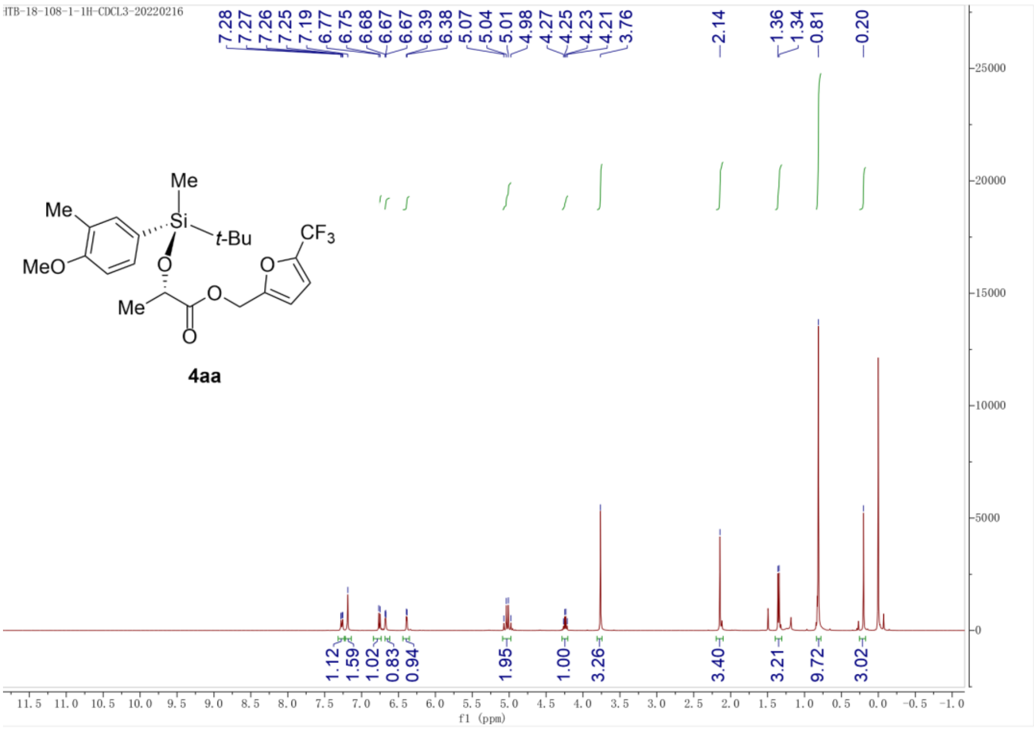

Supplementary Figure 117. ^{1}H NMR (400 M, $CDCl_3$, 25 ^{o}C) of compound **4aa**

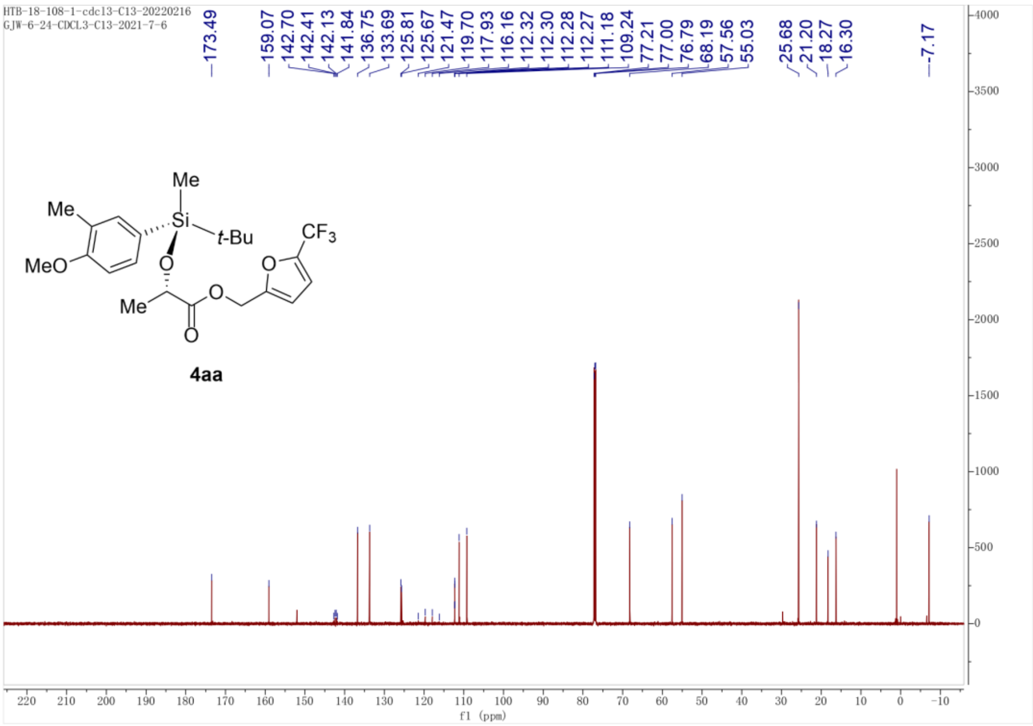

Supplementary Figure 118. ^{13}C NMR (150 M, $CDCl_3$, 25 ^{o}C) of compound **4aa**

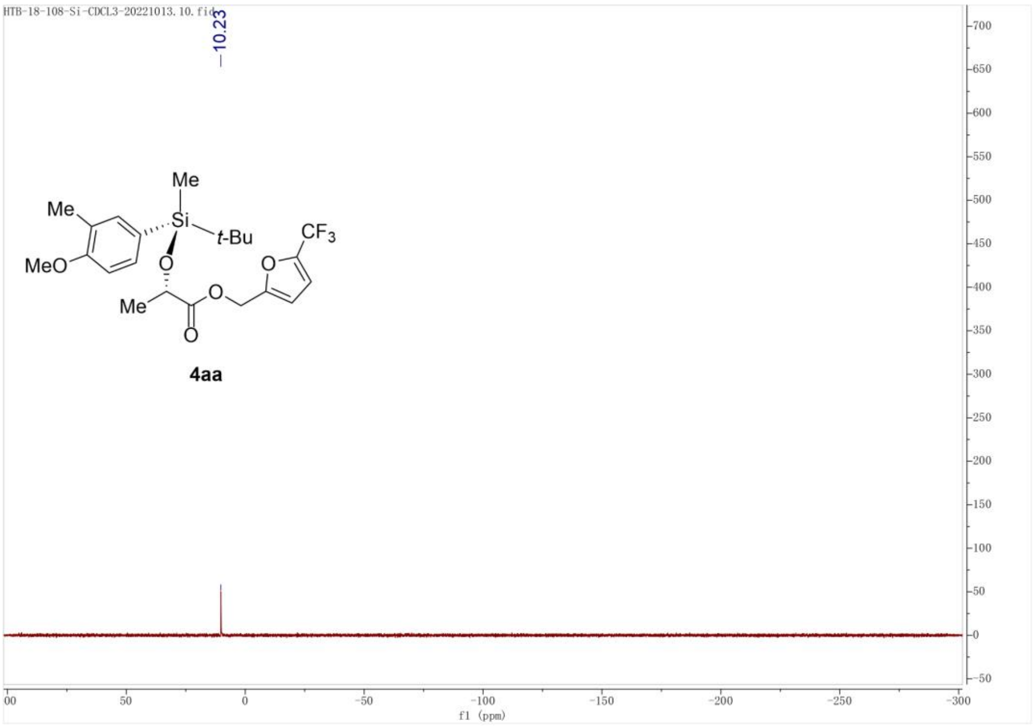

Supplementary Figure 119. ^{29}Si NMR (80 M, $CDCl_3$, 25 ^{o}C) of compound **4aa**

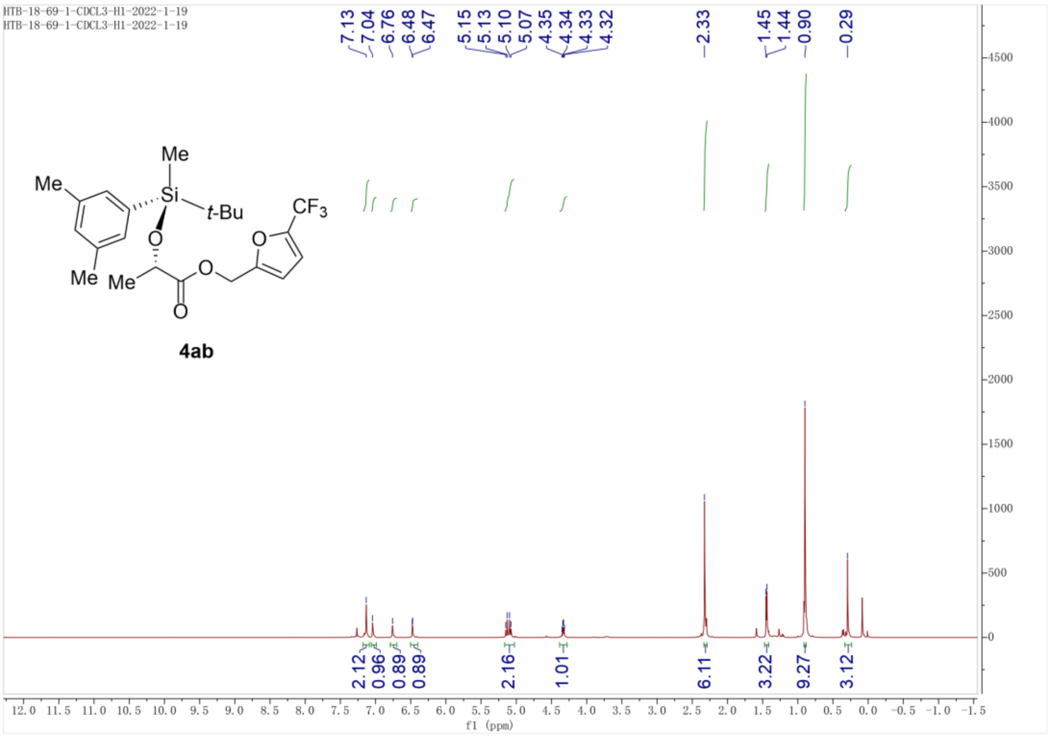

Supplementary Figure 120. ^{1}H NMR (400 M, $CDCl_3$, 25 ^{o}C) of compound **4ab**

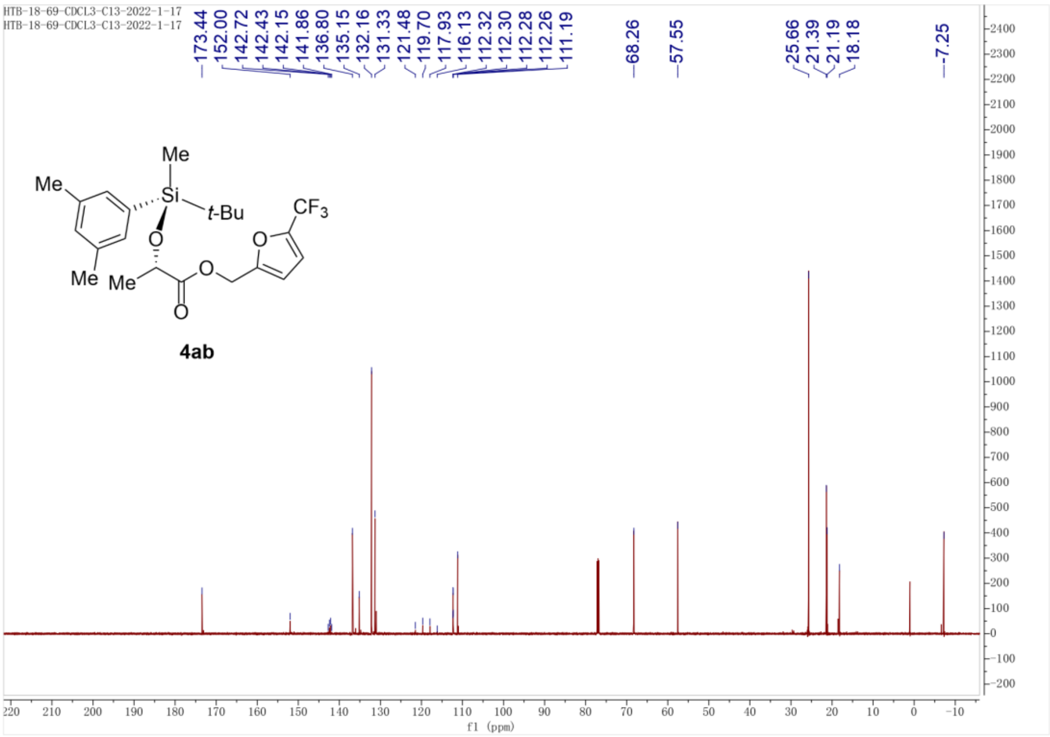

Supplementary Figure 121. ^{13}C NMR (150 M, $CDCl_3$, 25 °C) of compound **4ab**

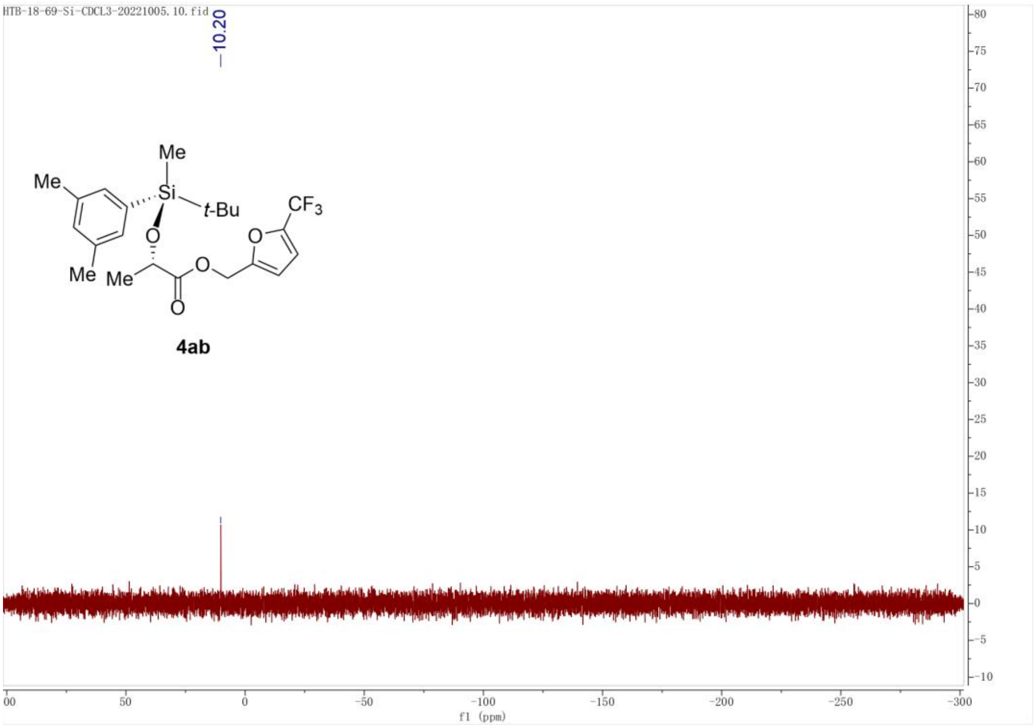

Supplementary Figure 122. ^{29}Si NMR (80 M, $CDCl_3$, 25 °C) of compound **4ab**

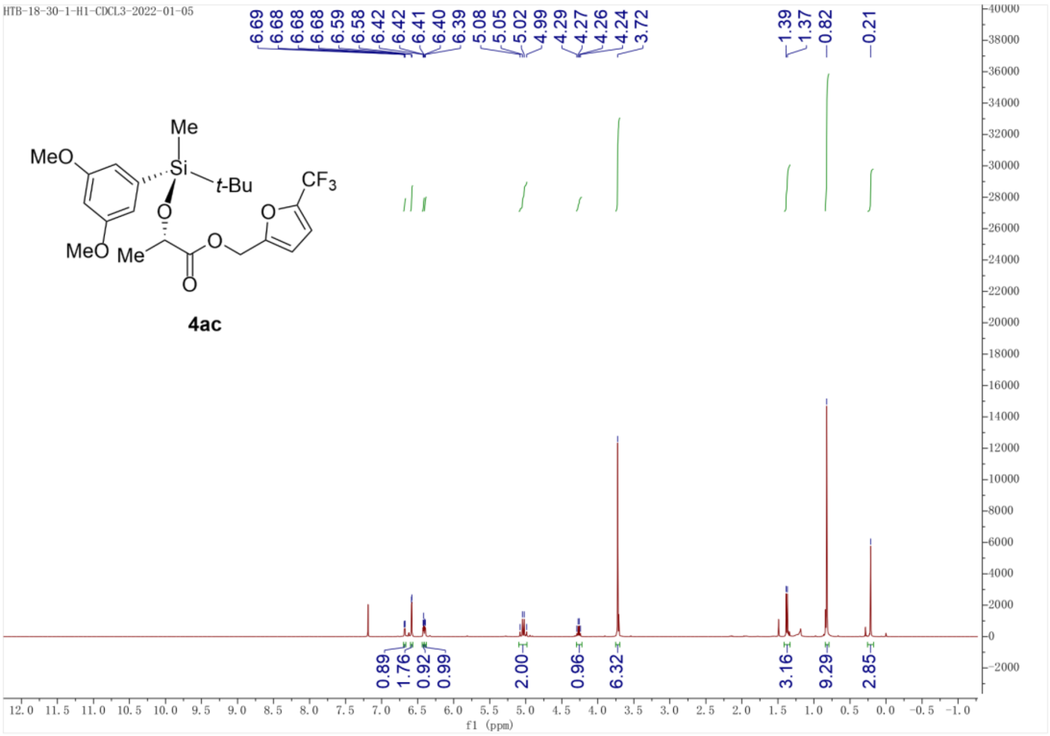

Supplementary Figure 123. ^{1}H NMR (400 M, $CDCl_3$, 25 °C) of compound **4ac**

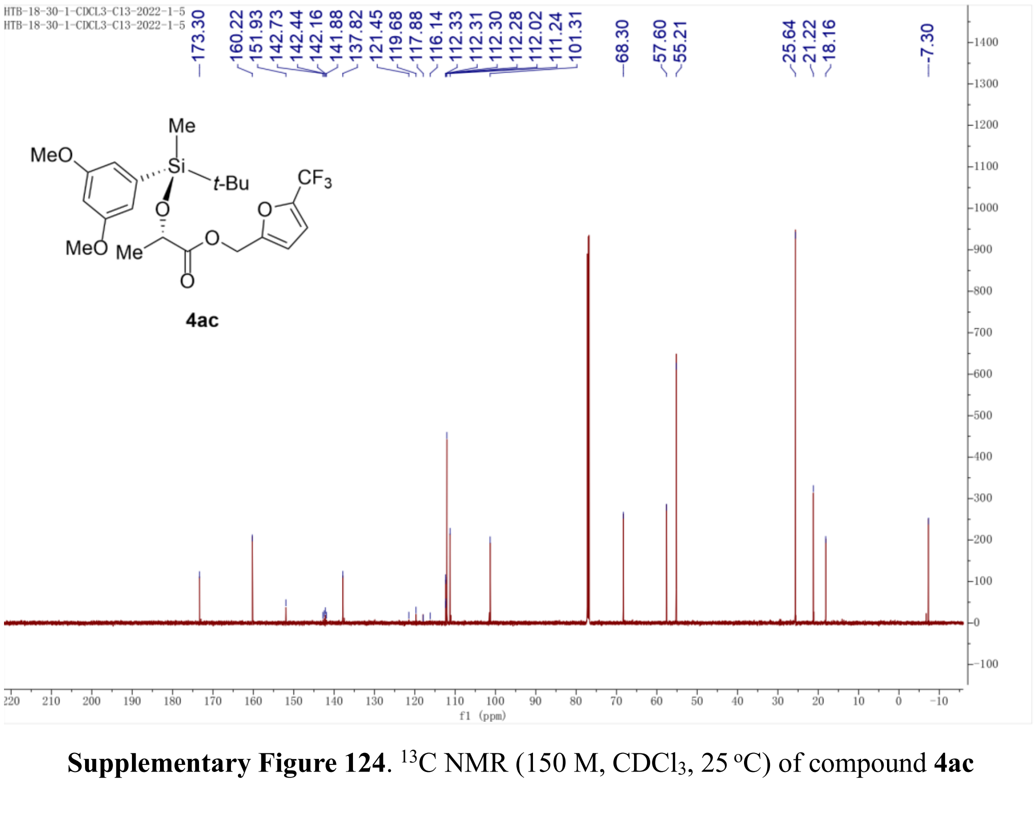

Supplementary Figure 124. ^{13}C NMR (150 M, $CDCl_3$, 25 °C) of compound **4ac**

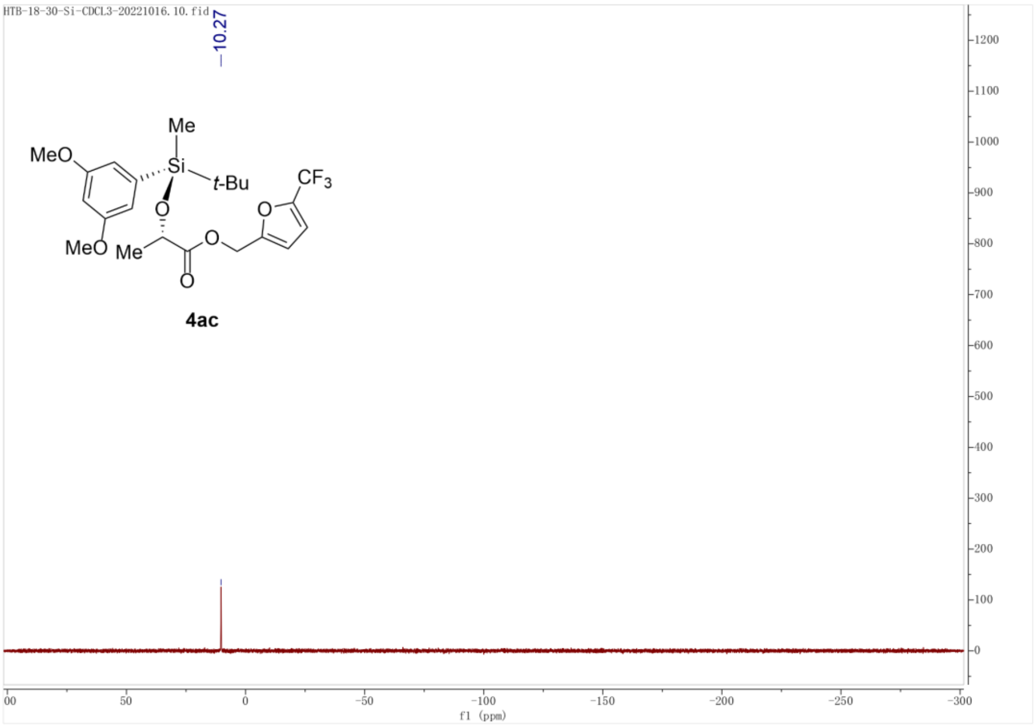

Supplementary Figure 125. ^{29}Si NMR (80 M, $CDCl_3$, 25 ^{o}C) of compound **4ac**

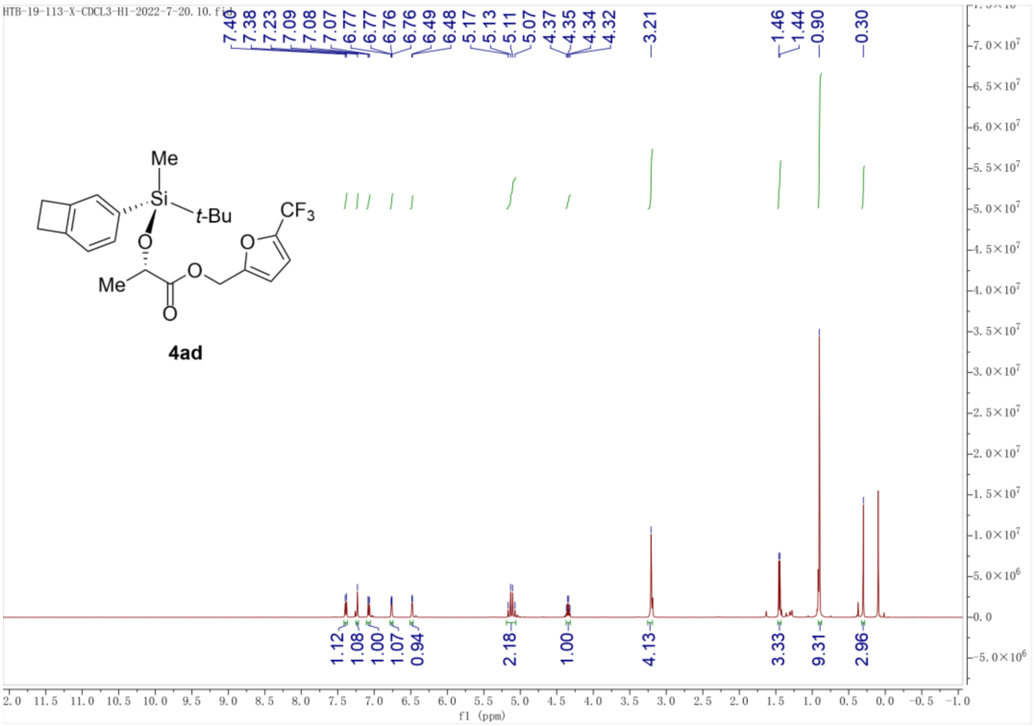

Supplementary Figure 126. ^{1}H NMR (400 M, $CDCl_3$, 25 ^{o}C) of compound **4ad**

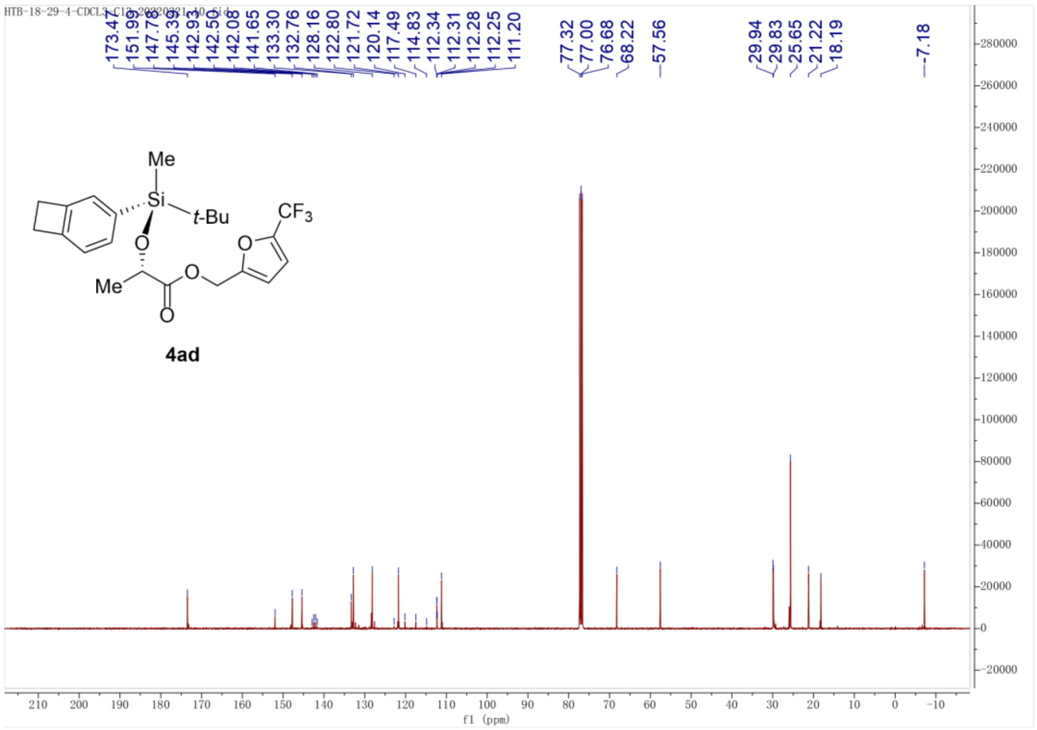

Supplementary Figure 127. ^{13}C NMR (150 M, $CDCl_3$, 25 °C) of compound **4ad**

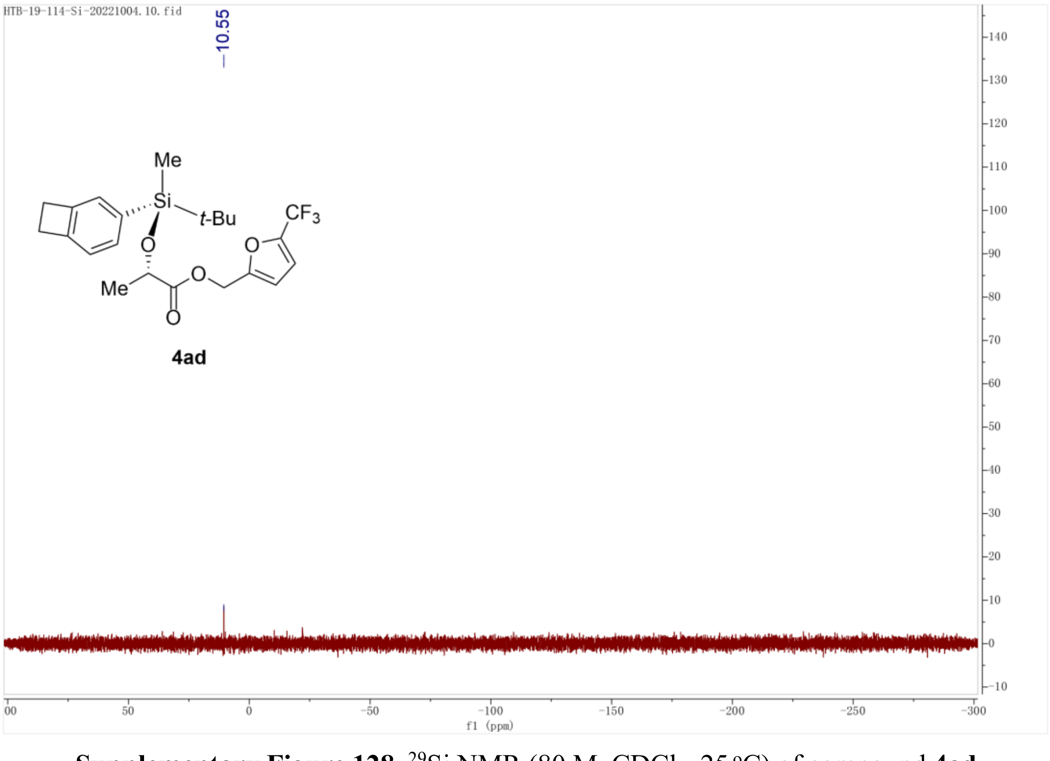

Supplementary Figure 128. ^{29}Si NMR (80 M, $CDCl_3$, 25 °C) of compound **4ad**

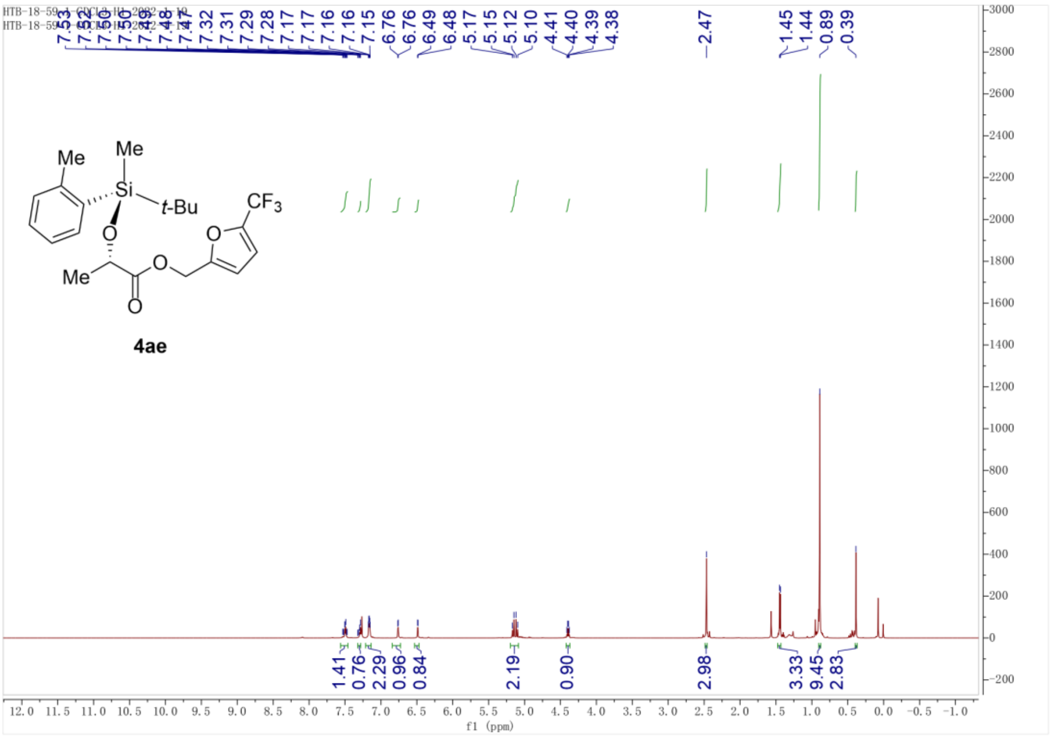

Supplementary Figure 129. ^{1}H NMR (400 M, $CDCl_3$, 25 °C) of compound **4ae**

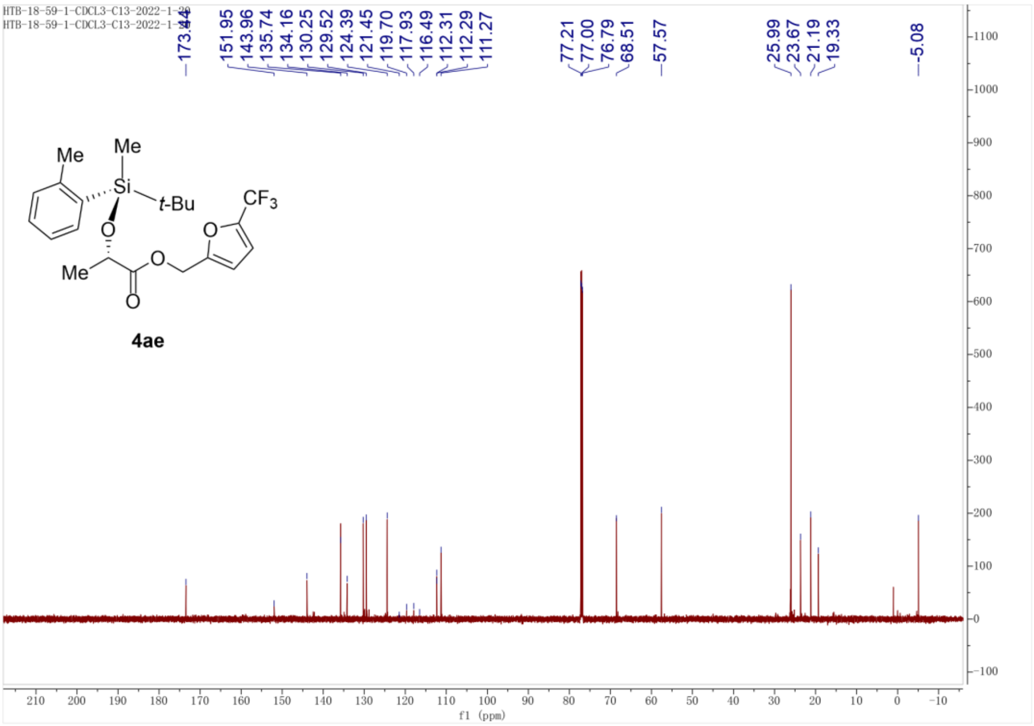

Supplementary Figure 130. ^{13}C NMR (100 M, $CDCl_3$, 25 °C) of compound **4ae**

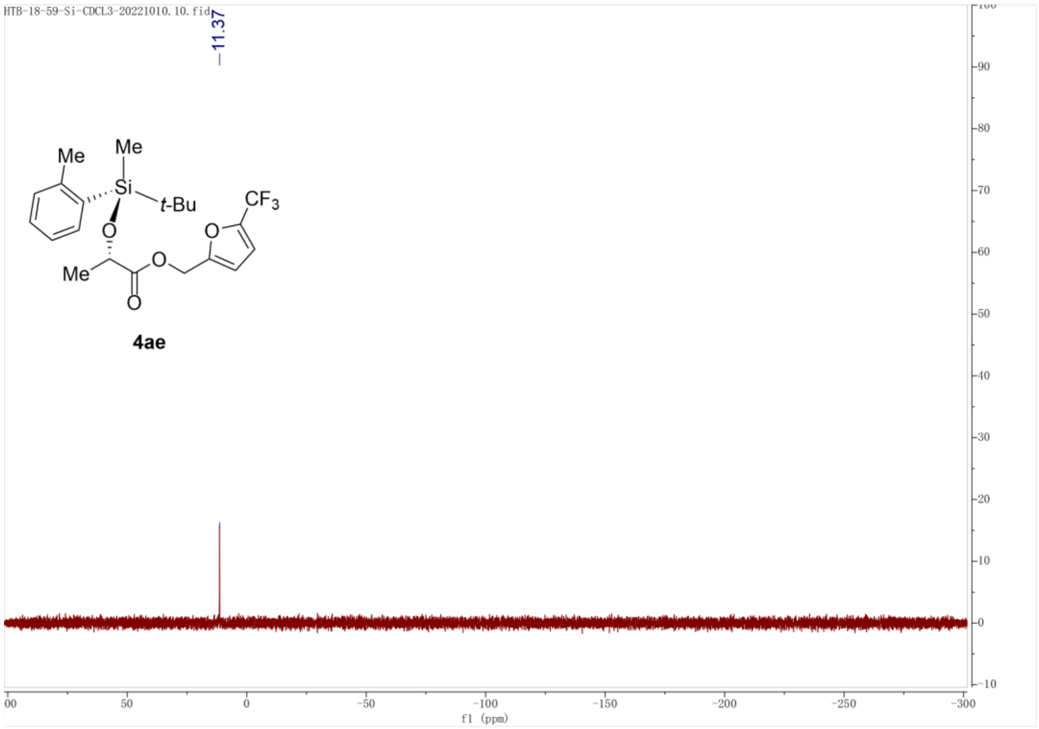

Supplementary Figure 131. ^{29}Si NMR (80 M, $CDCl_3$, 25 °C) of compound **4ae**

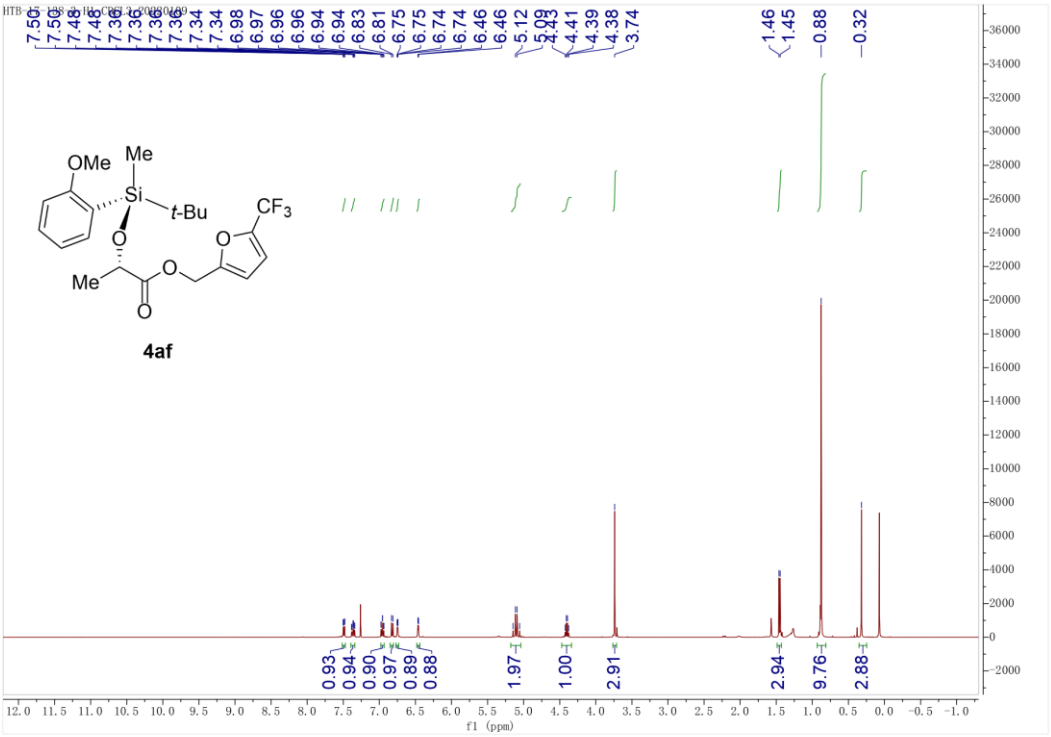

Supplementary Figure 132. ^{1}H NMR (400 M, $CDCl_3$, 25 °C) of compound **4af**

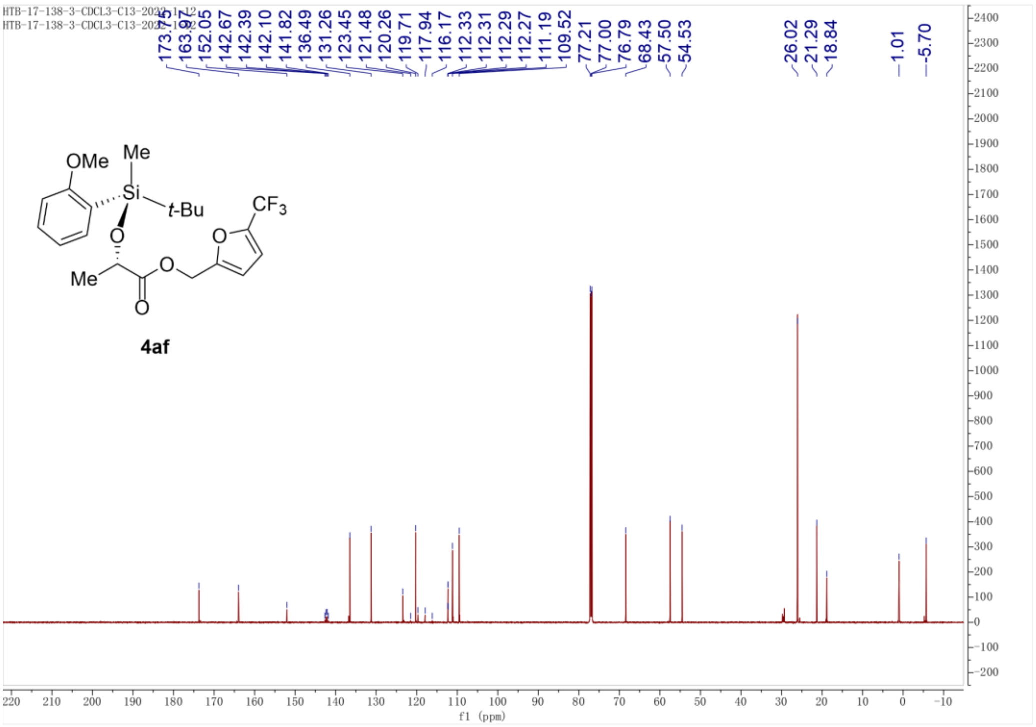

Supplementary Figure 133. ^{13}C NMR (100 M, $CDCl_3$, 25 °C) of compound **4af**

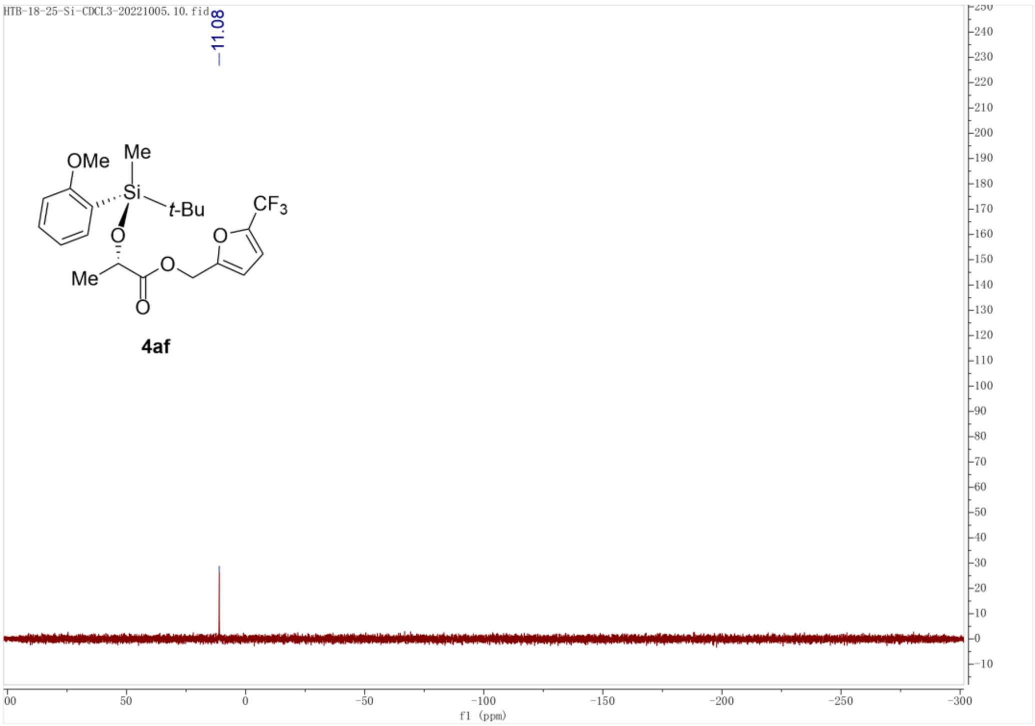

Supplementary Figure 134. ^{29}Si NMR (80 M, $CDCl_3$, 25 °C) of compound **4af**

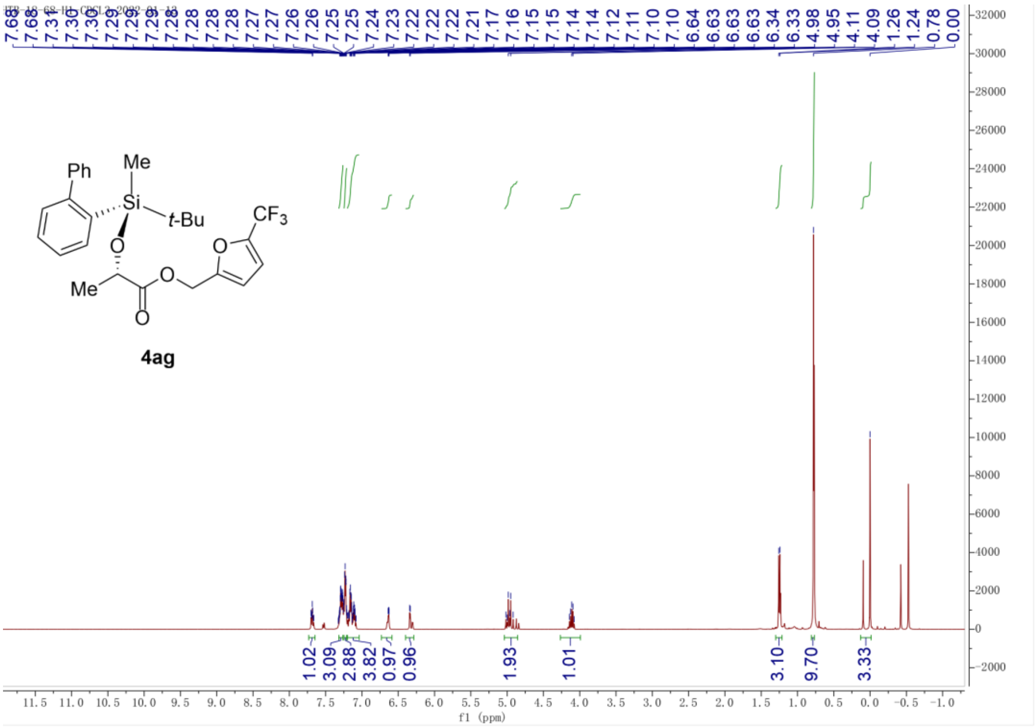

Supplementary Figure 135. ^{1}H NMR (400 M, $CDCl_3$, 25 ^{o}C) of compound **4ag**

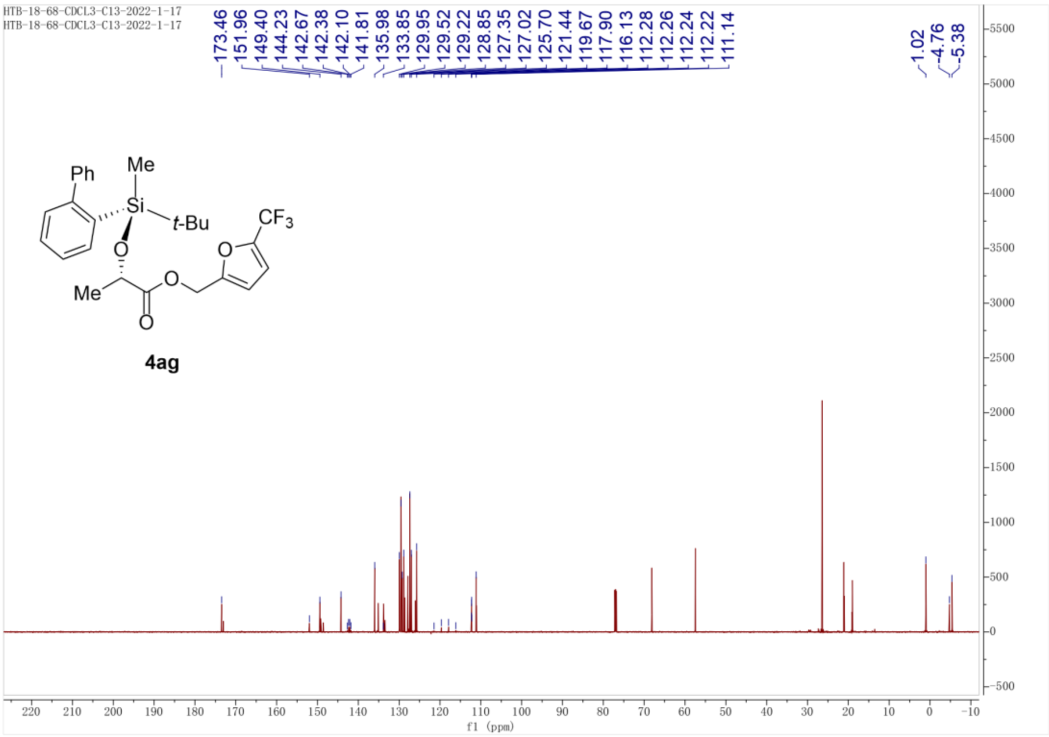

Supplementary Figure 136. ^{13}C NMR (100 M, $CDCl_3$, 25 ^{o}C) of compound **4ag**

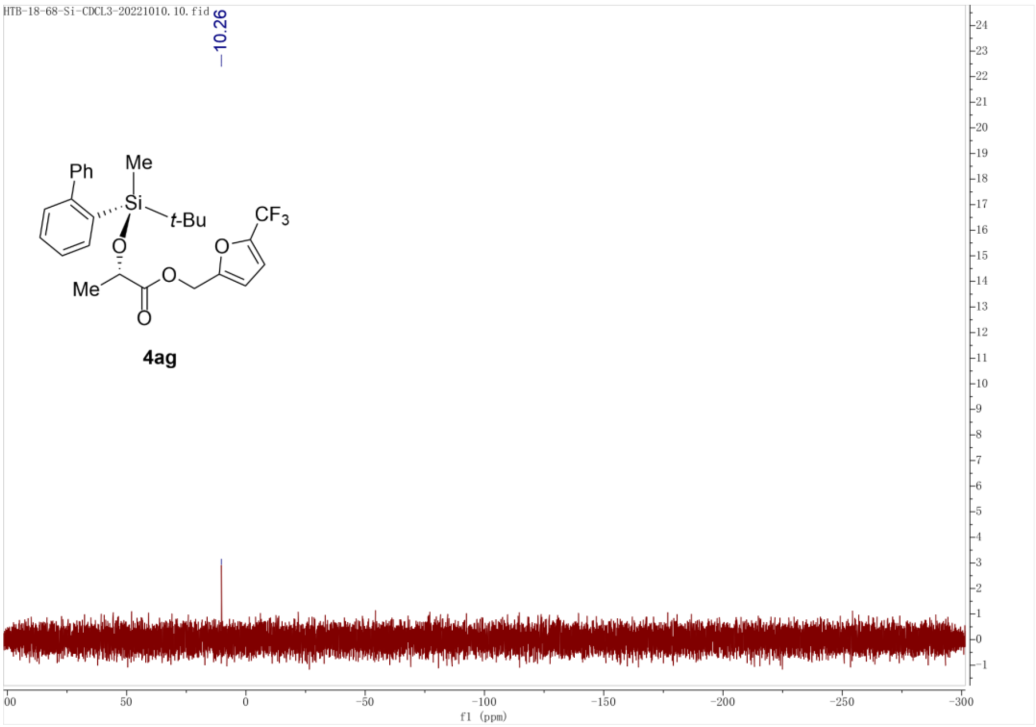

Supplementary Figure 137. ^{29}Si NMR (80 M, $CDCl_3$, 25 °C) of compound **4ag**

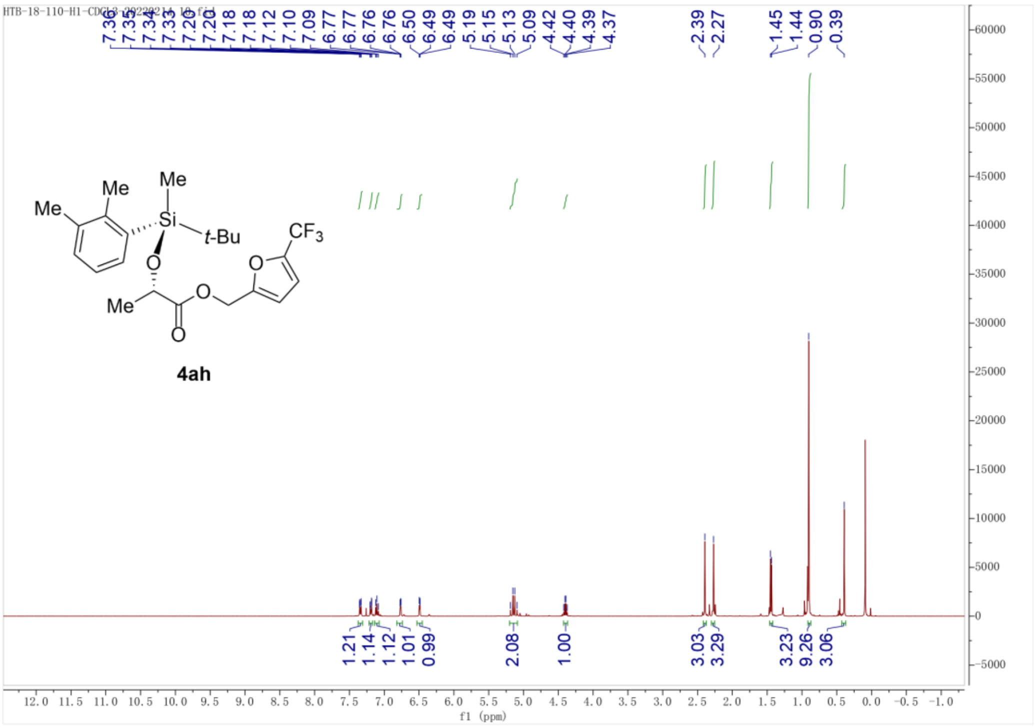

Supplementary Figure 138. ^{1}H NMR (400 M, $CDCl_3$, 25 °C) of compound **4ah**

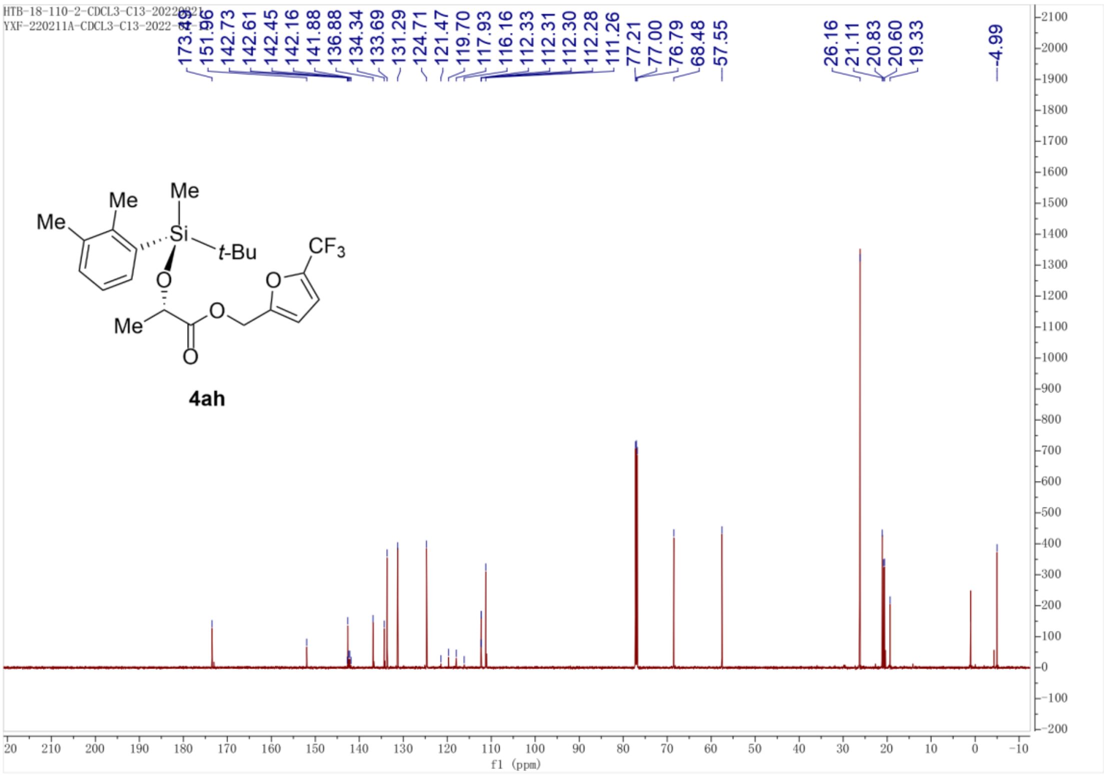

Supplementary Figure 139. ^{13}C NMR (150 M, $CDCl_3$, 25 °C) of compound **4ah**

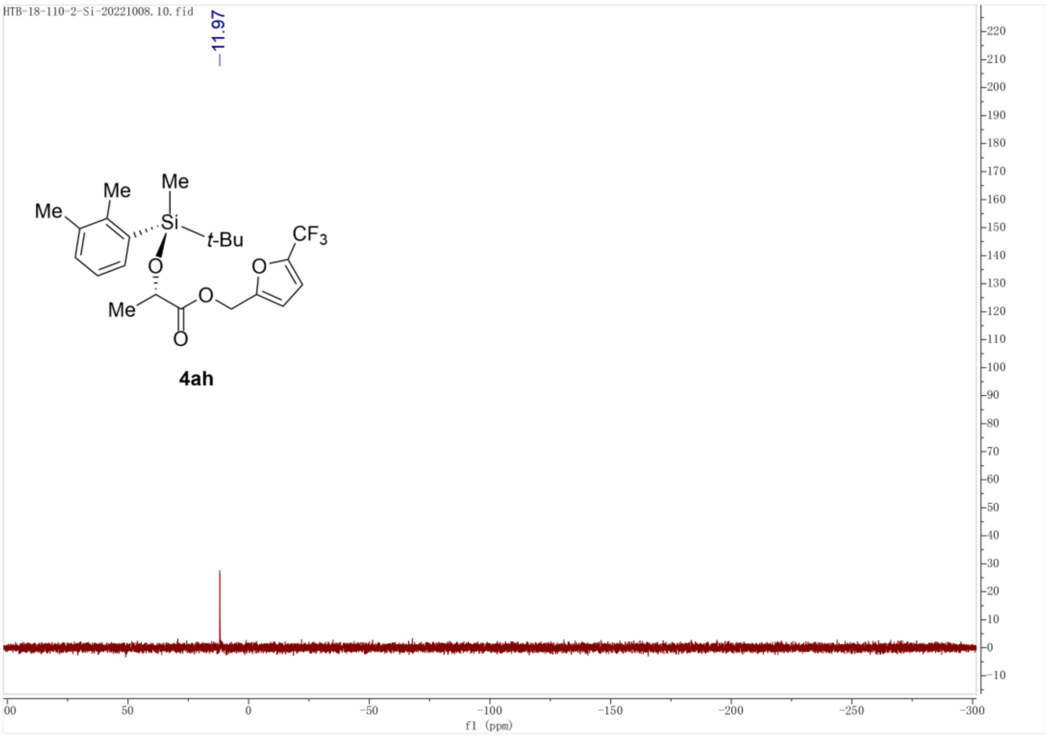

Supplementary Figure 140. ^{29}Si NMR (80 M, $CDCl_3$, 25 °C) of compound **4ah**

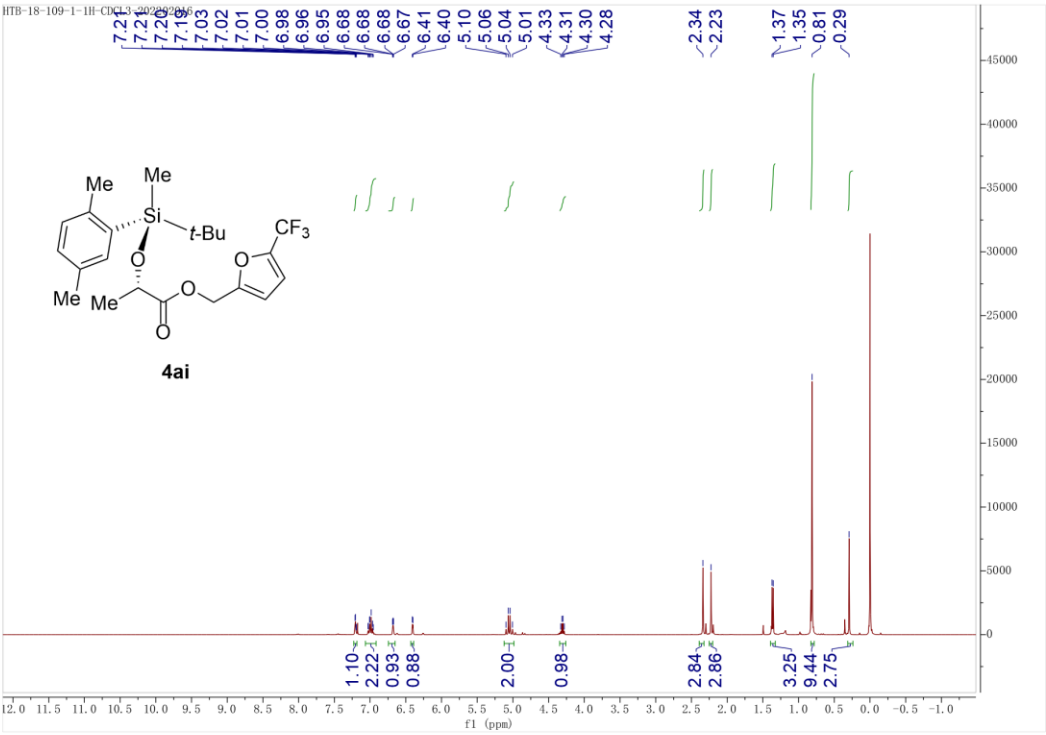

Supplementary Figure 141. ^{1}H NMR (400 M, $CDCl_3$, 25 ^{o}C) of compound **4ai**

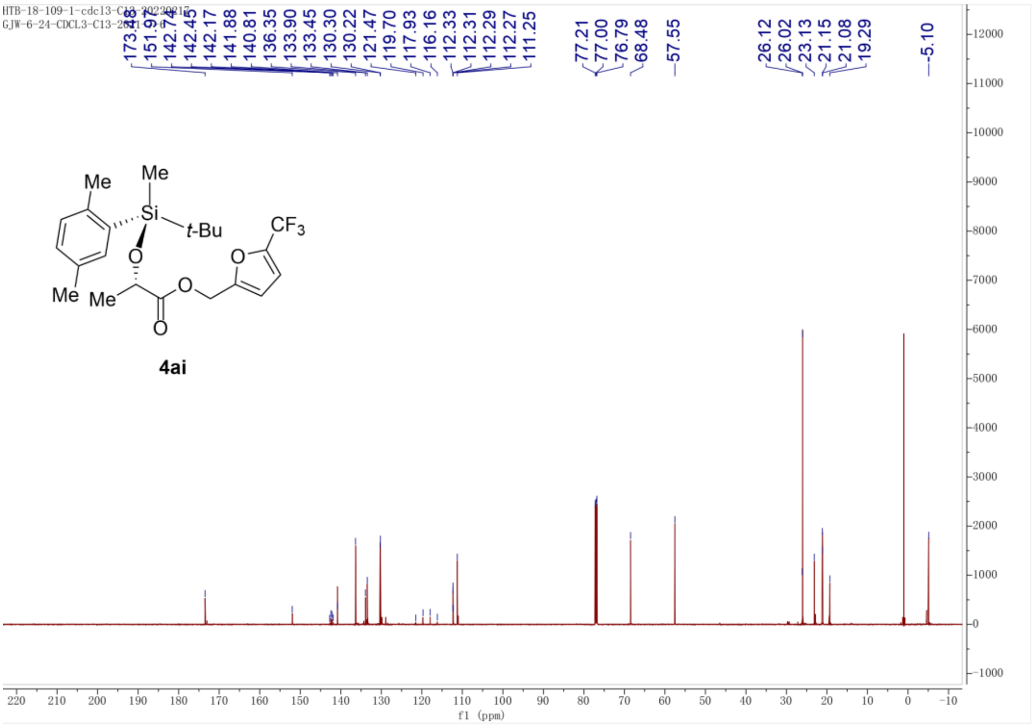

Supplementary Figure 142. ^{13}C NMR (100 M, $CDCl_3$, 25 ^{o}C) of compound **4ai**

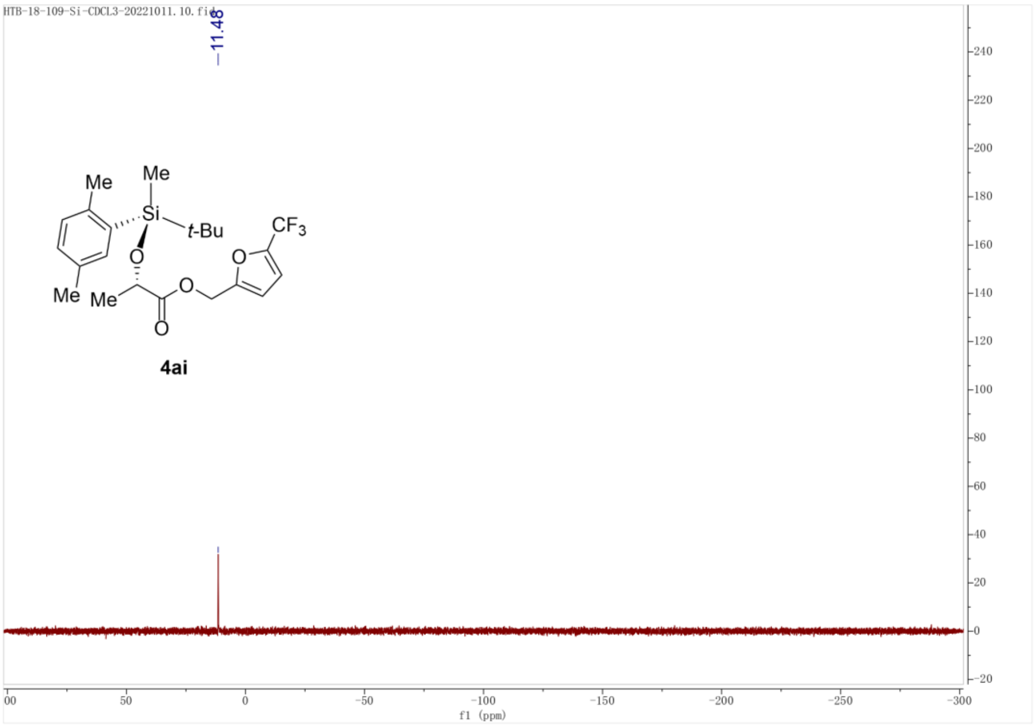

Supplementary Figure 143. ^{29}Si NMR (80 M, $CDCl_3$, 25 °C) of compound **4ai**

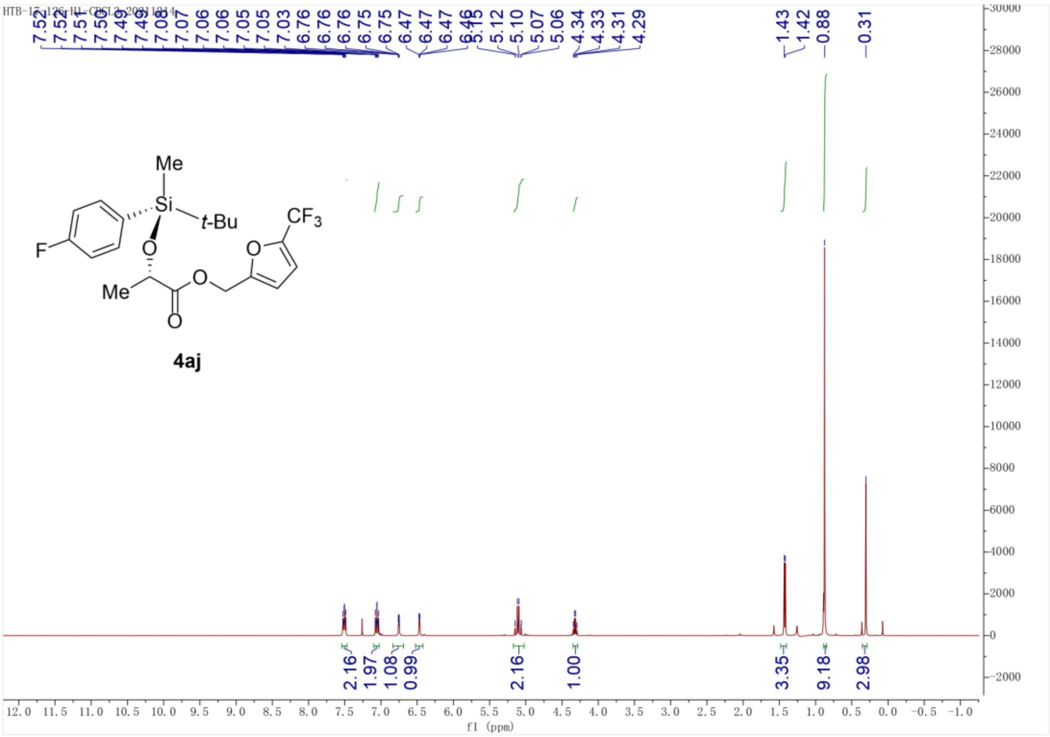

Supplementary Figure 144. ^{1}H NMR (400 M, $CDCl_3$, 25 °C) of compound **4aj**

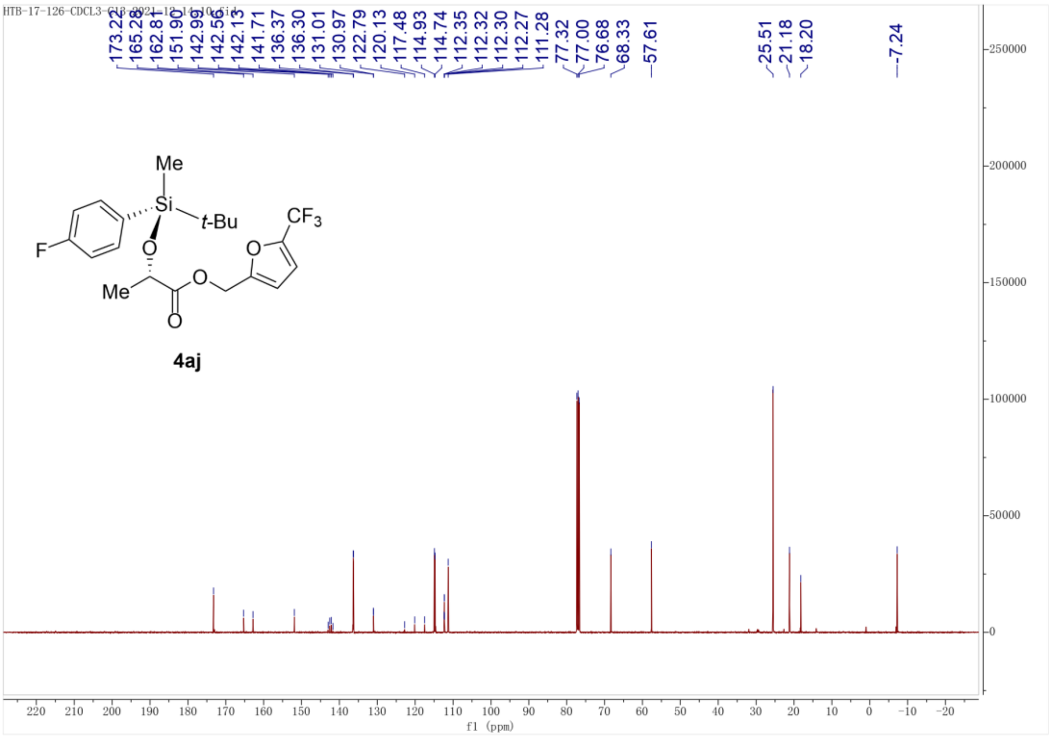

Supplementary Figure 145. ^{13}C NMR (100 M, $CDCl_3$, 25 °C) of compound **4aj**

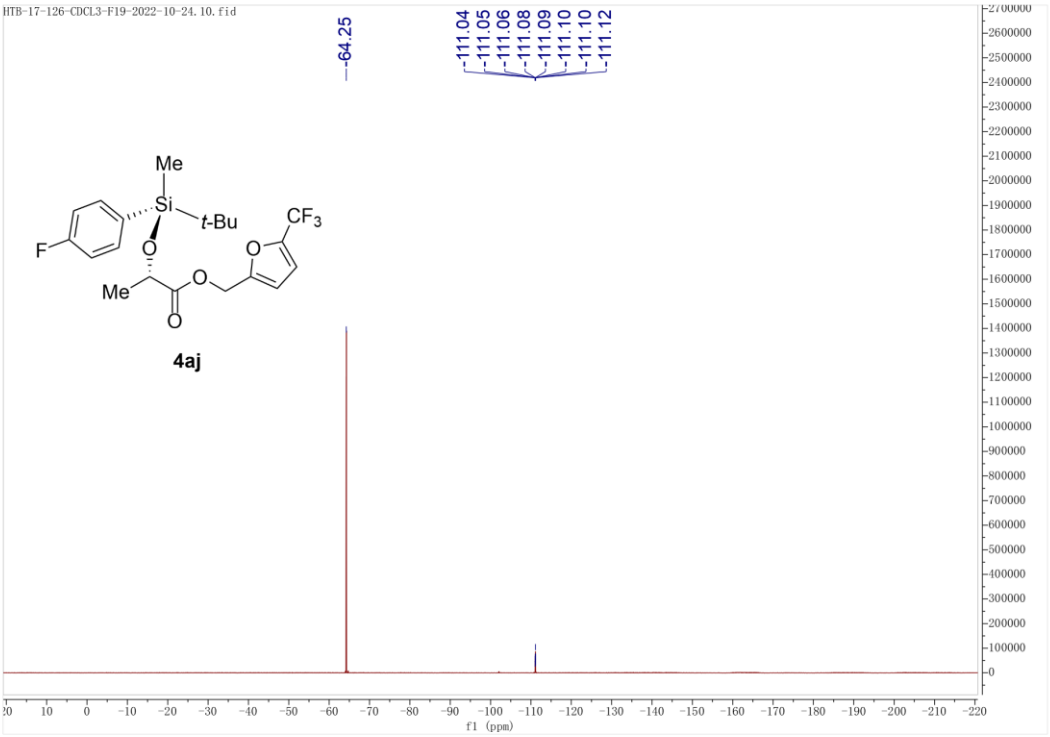

Supplementary Figure 146. ^{19}F NMR (376 M, $CDCl_3$, 25 °C) of compound **4aj**

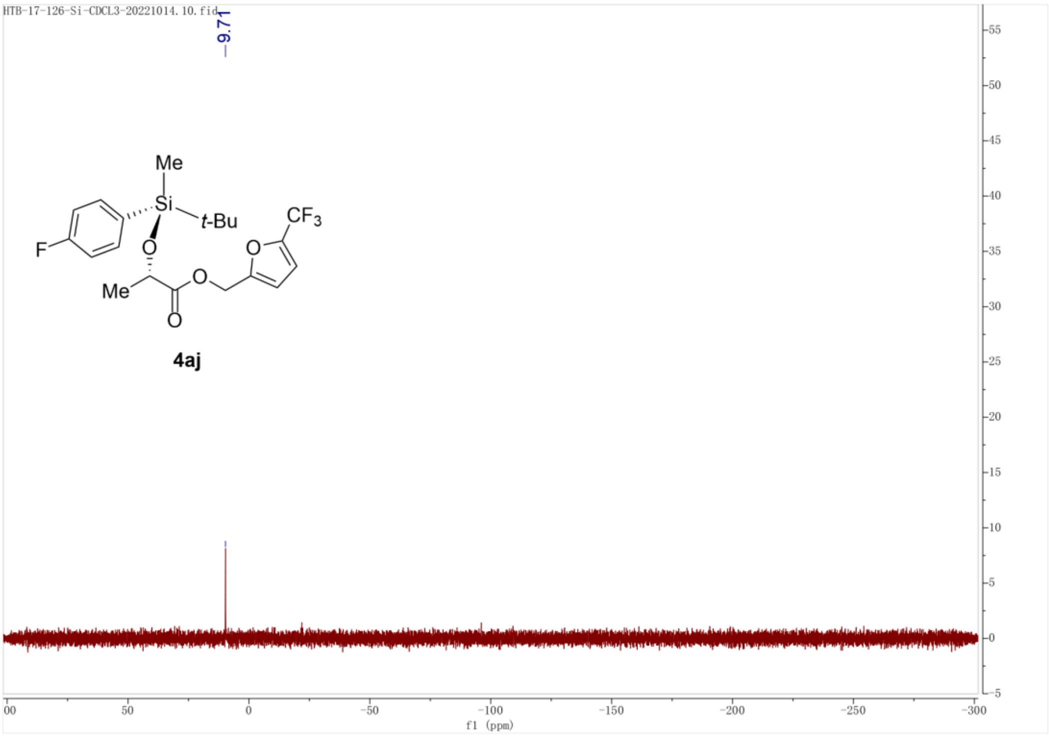

Supplementary Figure 147. ^{29}Si NMR (80 M, $CDCl_3$, 25 ^{o}C) of compound **4aj**

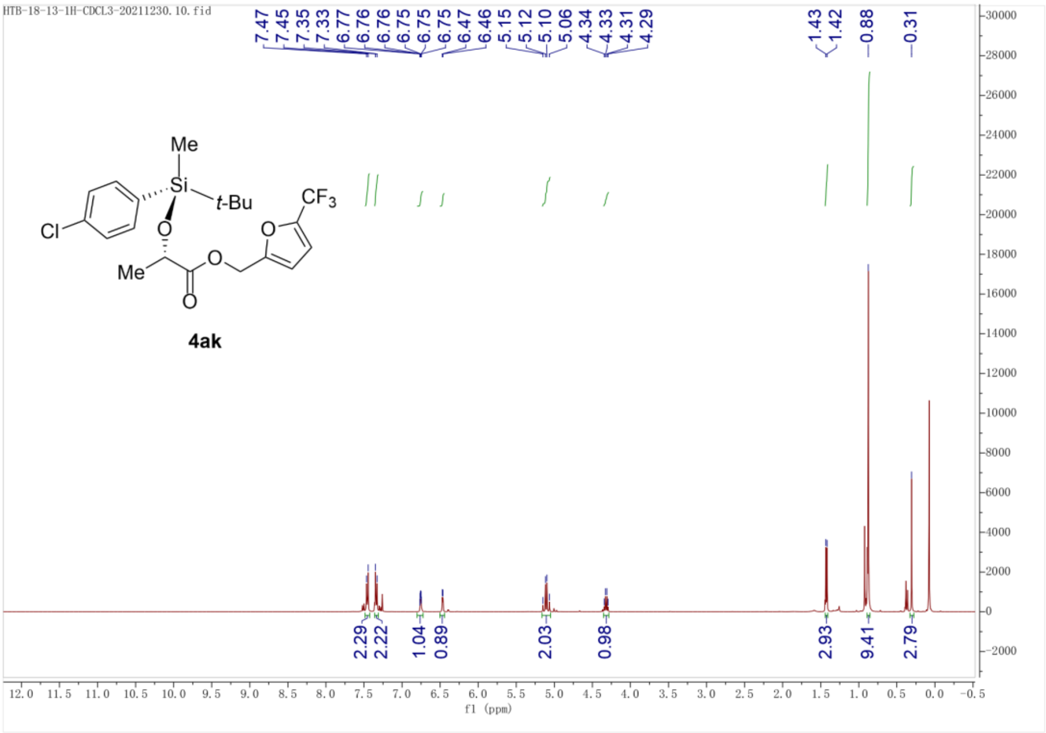

Supplementary Figure 148. ^{1}H NMR (400 M, $CDCl_3$, 25 ^{o}C) of compound **4ak**

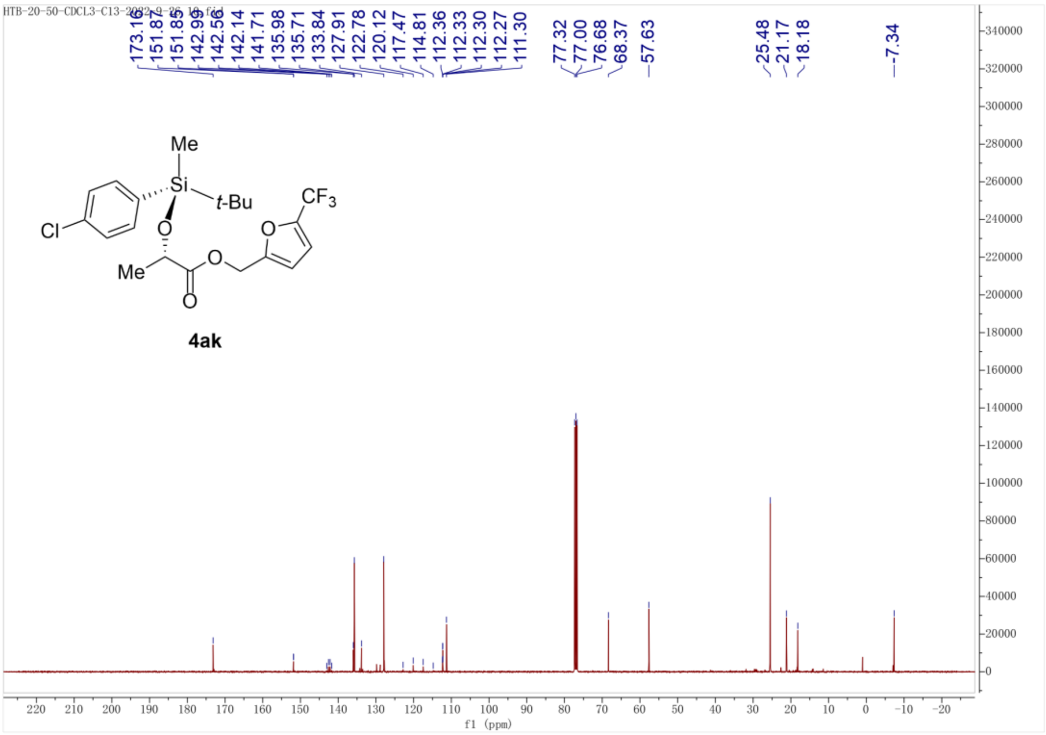

Supplementary Figure 149. ^{13}C NMR (100 M, $CDCl_3$, 25 ^{o}C) of compound **4ak**

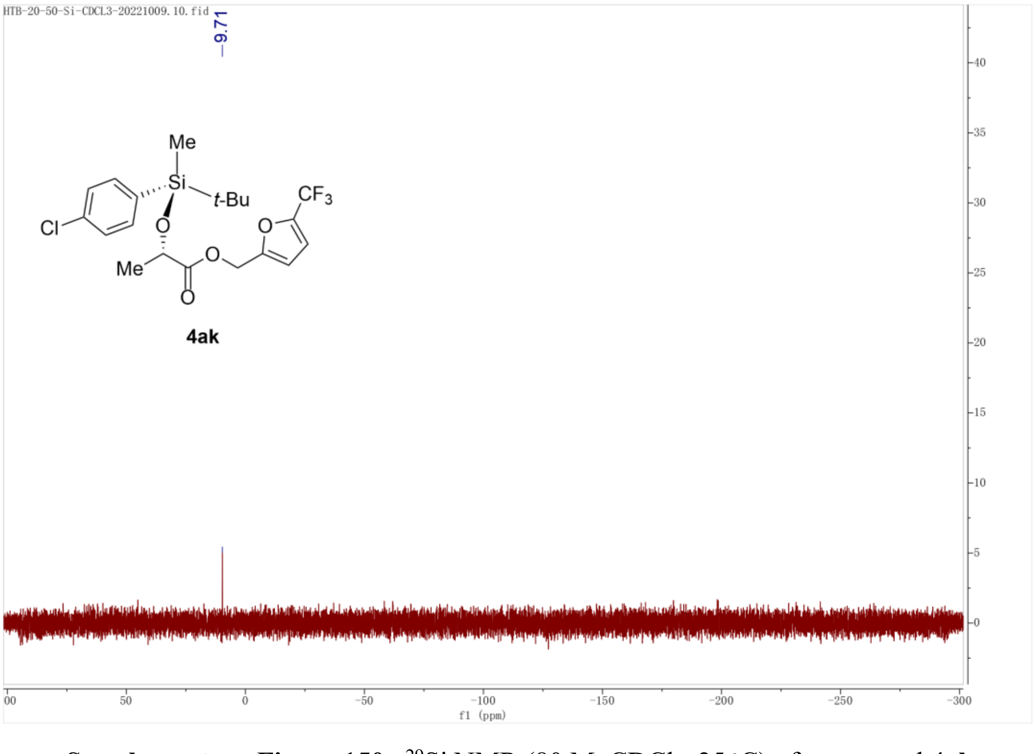

Supplementary Figure 150. ^{29}Si NMR (80 M, $CDCl_3$, 25 ^{o}C) of compound **4ak**

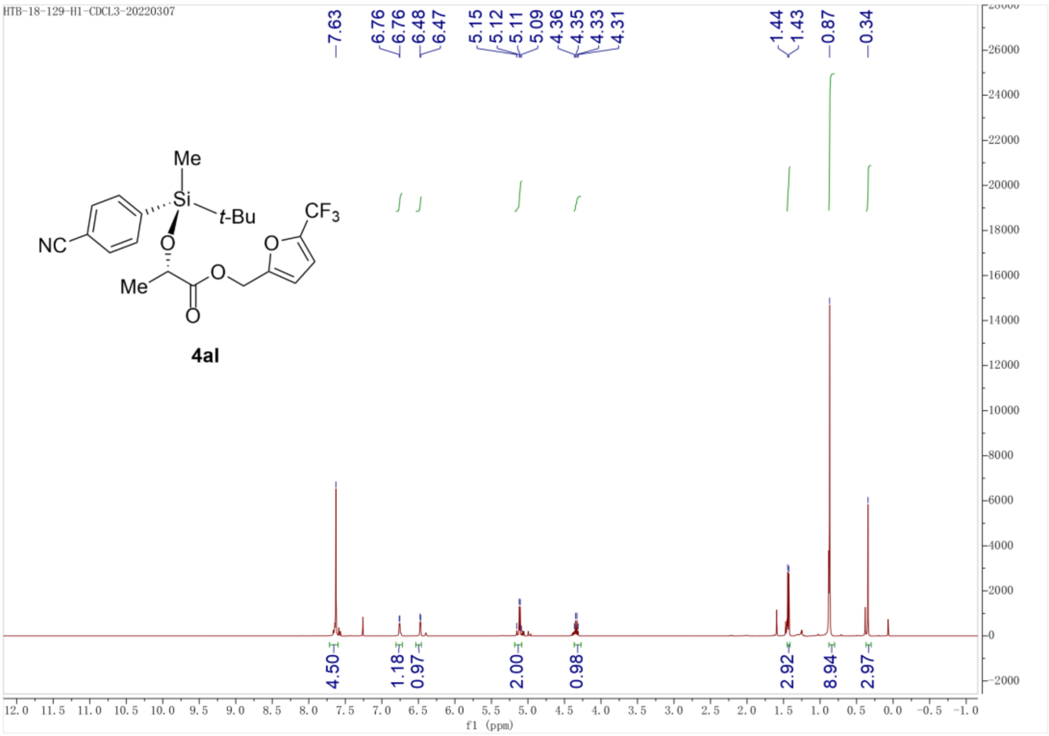

Supplementary Figure 151. ^{1}H NMR (400 M, $CDCl_3$, 25 ^{o}C) of compound **4al**

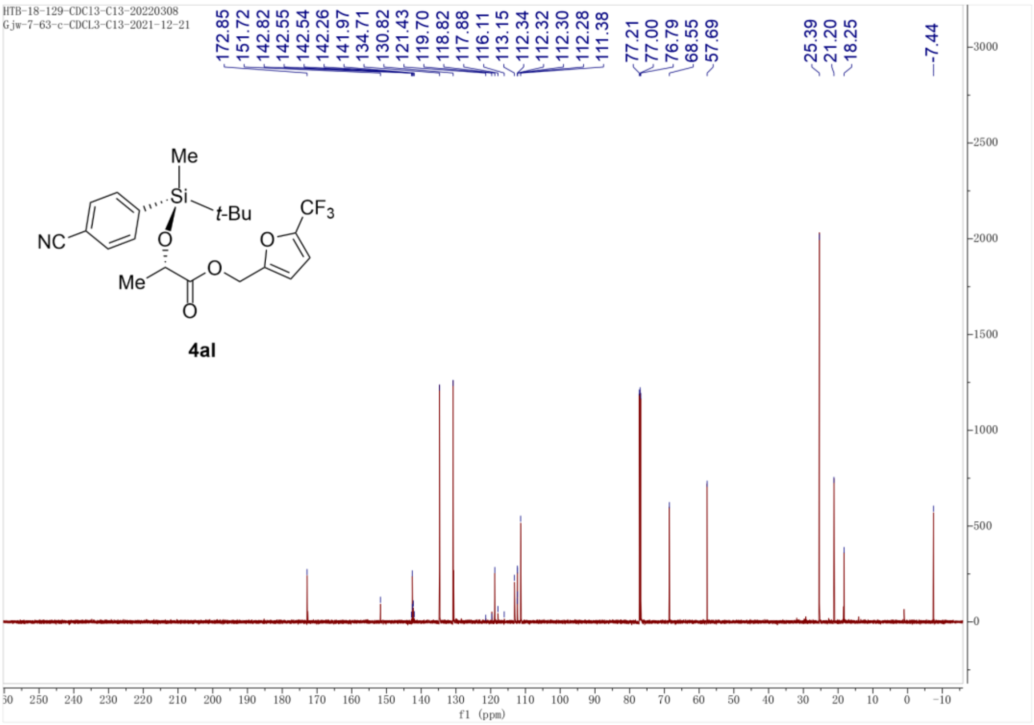

Supplementary Figure 152. ^{13}C NMR (150 M, $CDCl_3$, 25 ^{o}C) of compound **4al**

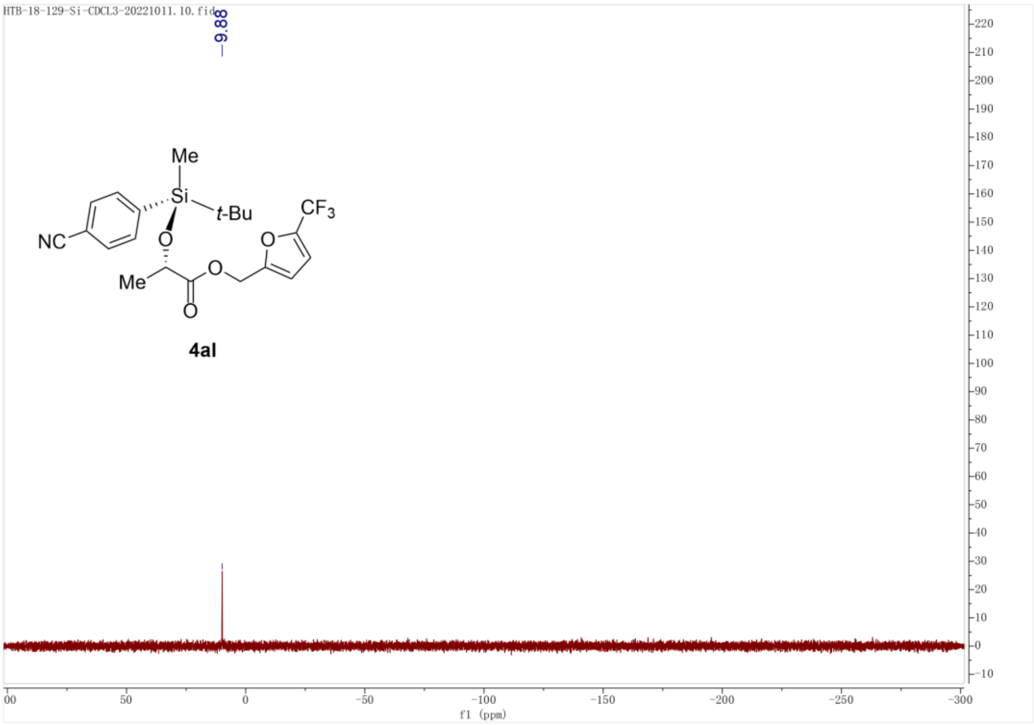

Supplementary Figure 153. ^{29}Si NMR (80 M, $CDCl_3$, 25 °C) of compound **4al**

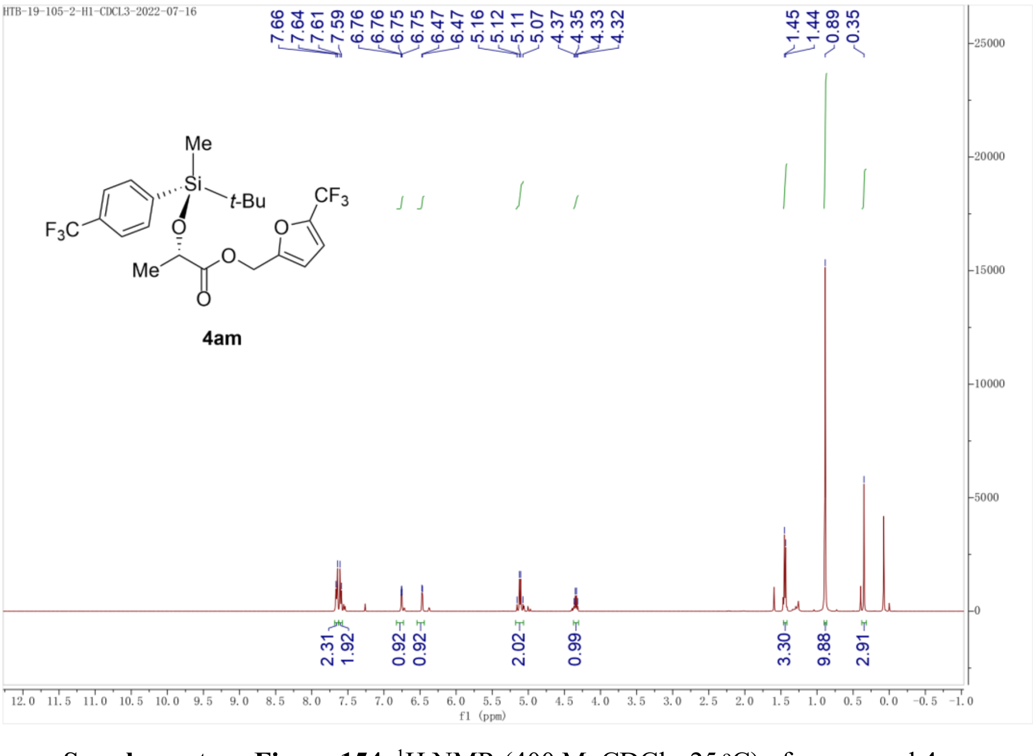

Supplementary Figure 154. ^{1}H NMR (400 M, $CDCl_3$, 25 °C) of compound **4am**

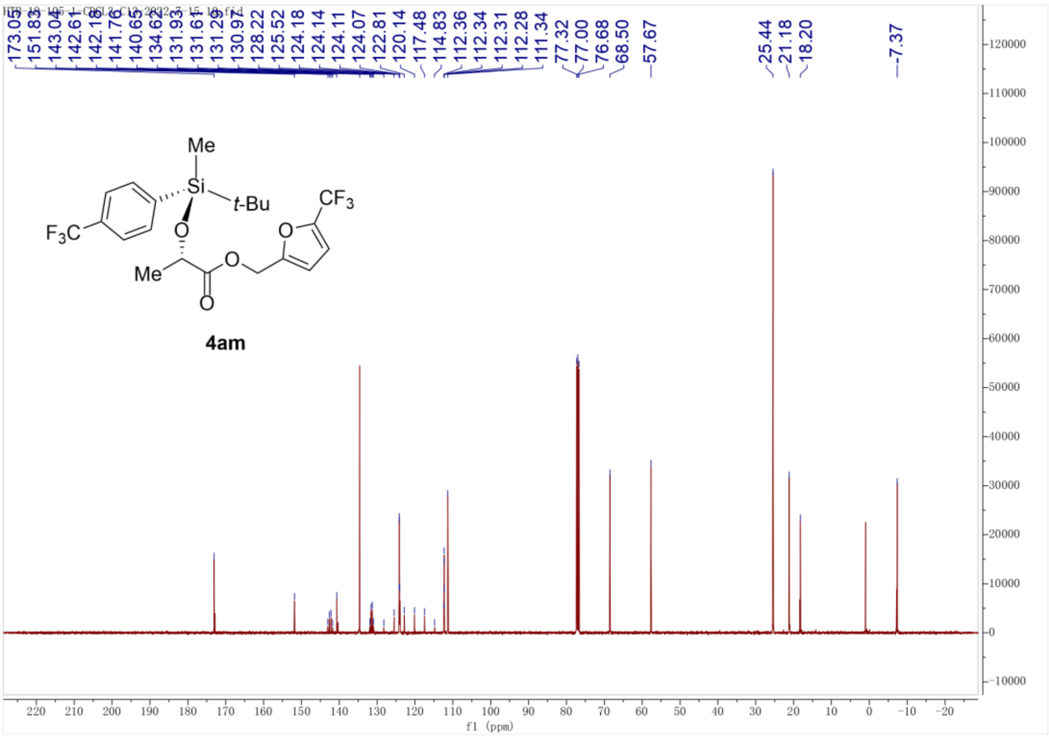

Supplementary Figure 155. ^{13}C NMR (100 M, $CDCl_3$, 25 °C) of compound **4am**

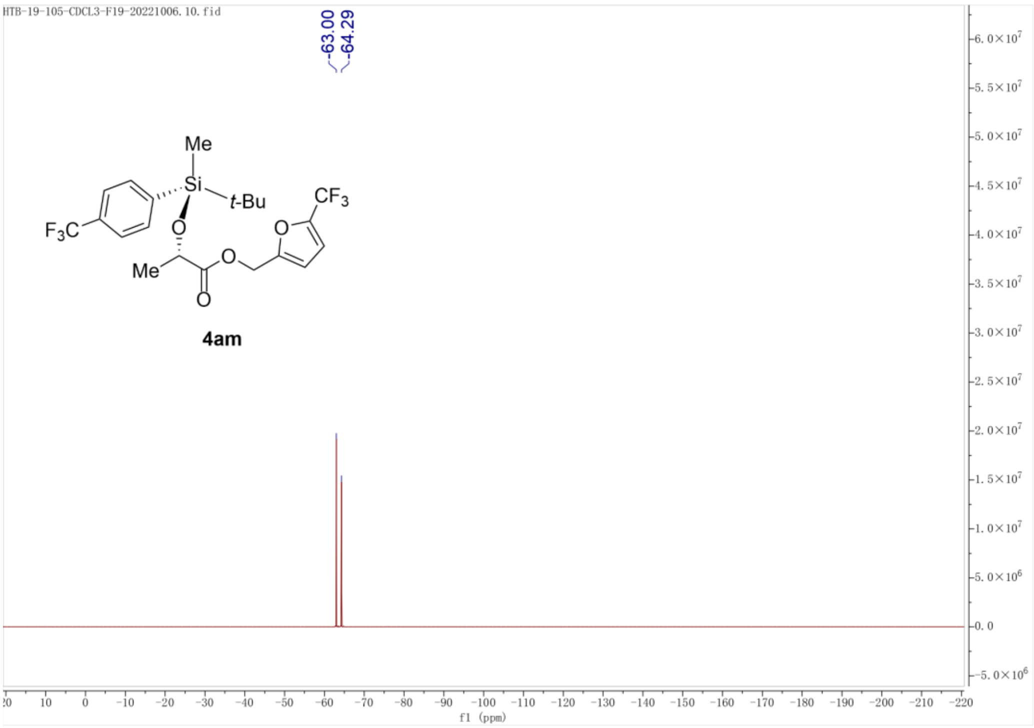

Supplementary Figure 156. ^{19}F NMR (376 M, $CDCl_3$, 25 °C) of compound **4am**

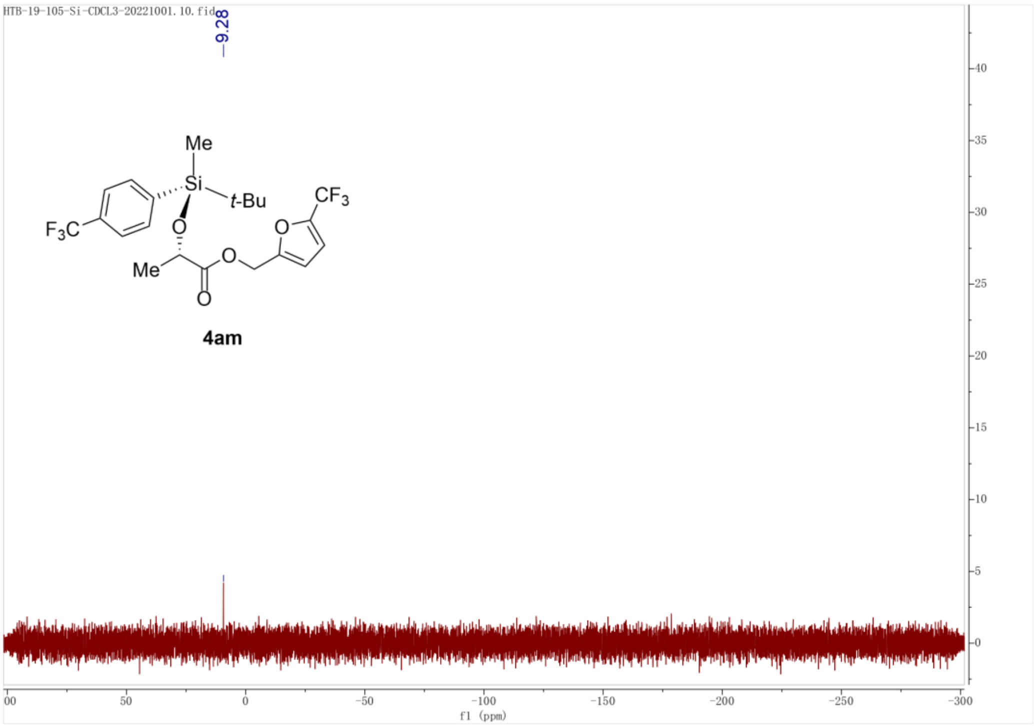

Supplementary Figure 157. ^{29}Si NMR (80 M, $CDCl_3$, 25 °C) of compound **4am**

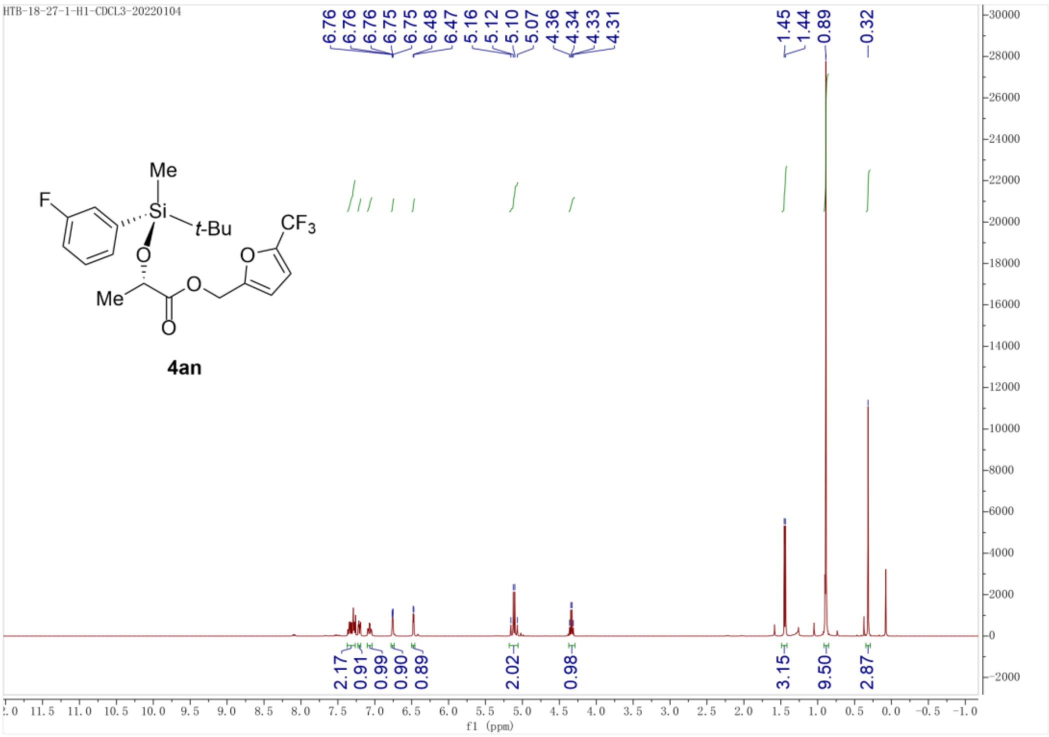

Supplementary Figure 158. ^{1}H NMR (400 M, $CDCl_3$, 25 °C) of compound **4an**

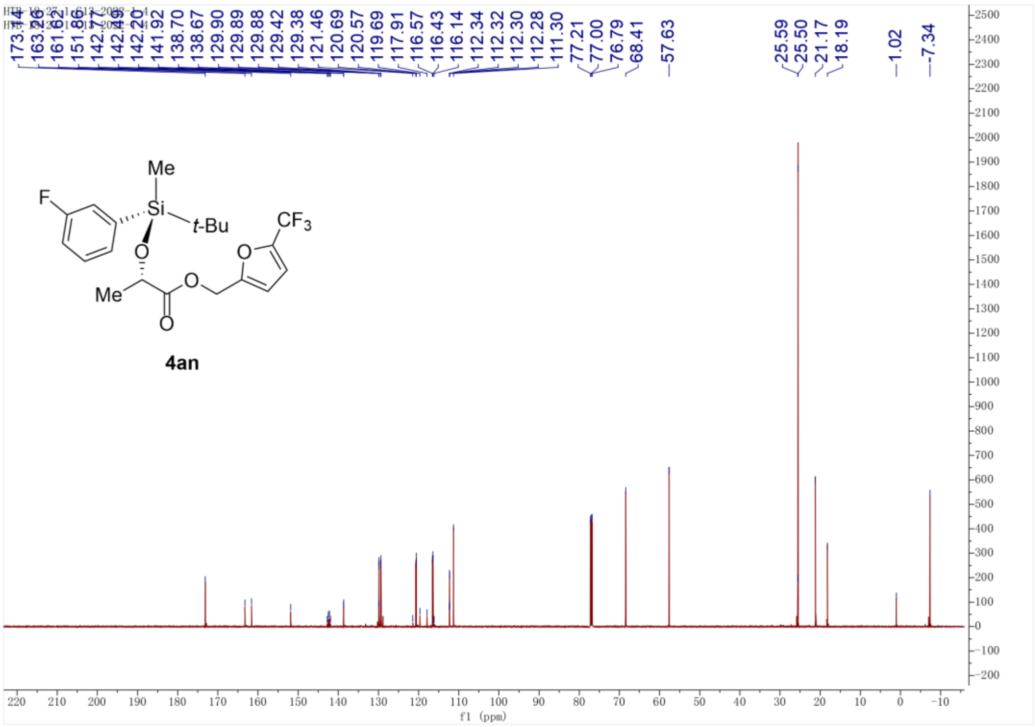

Supplementary Figure 159. ^{13}C NMR (150 M, $CDCl_3$, 25 °C) of compound **4an**

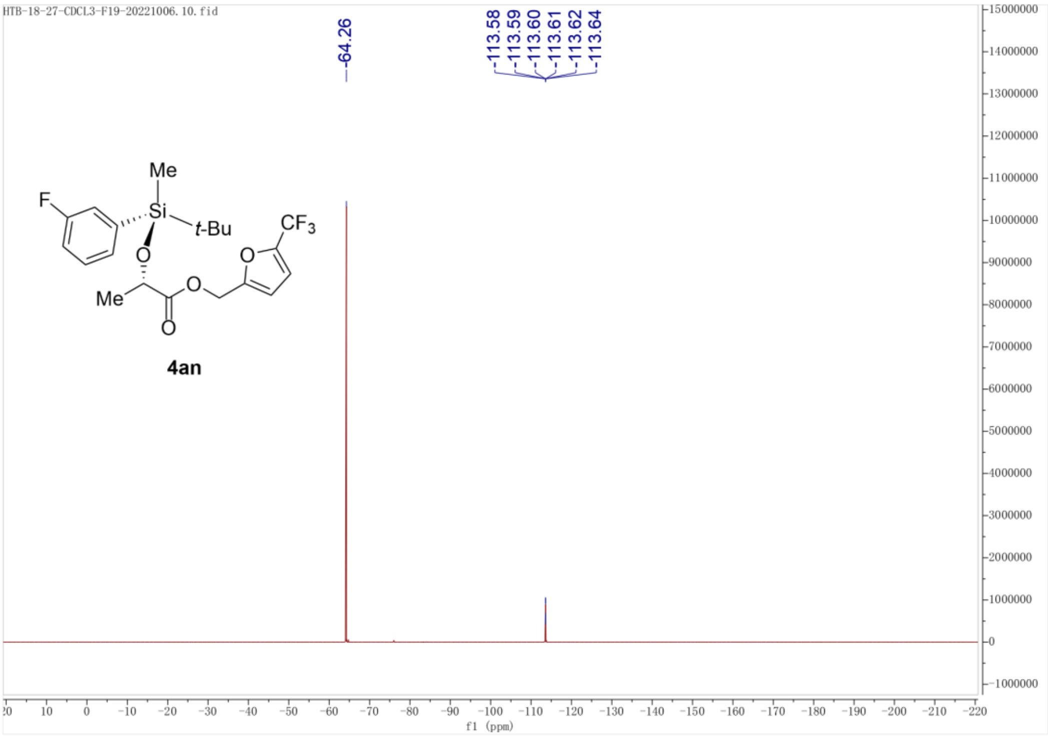

Supplementary Figure 160. ^{19}F NMR (376 M, $CDCl_3$, 25 °C) of compound **4an**

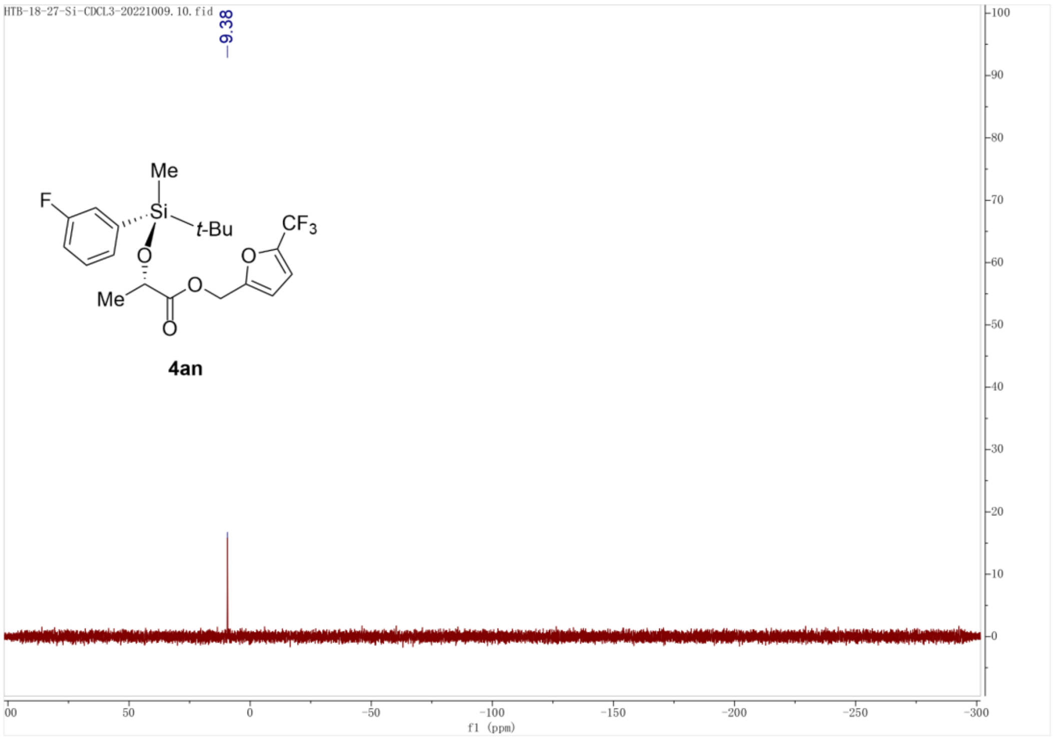

Supplementary Figure 161. ^{29}Si NMR (80 M, $CDCl_3$, 25 °C) of compound **4an**

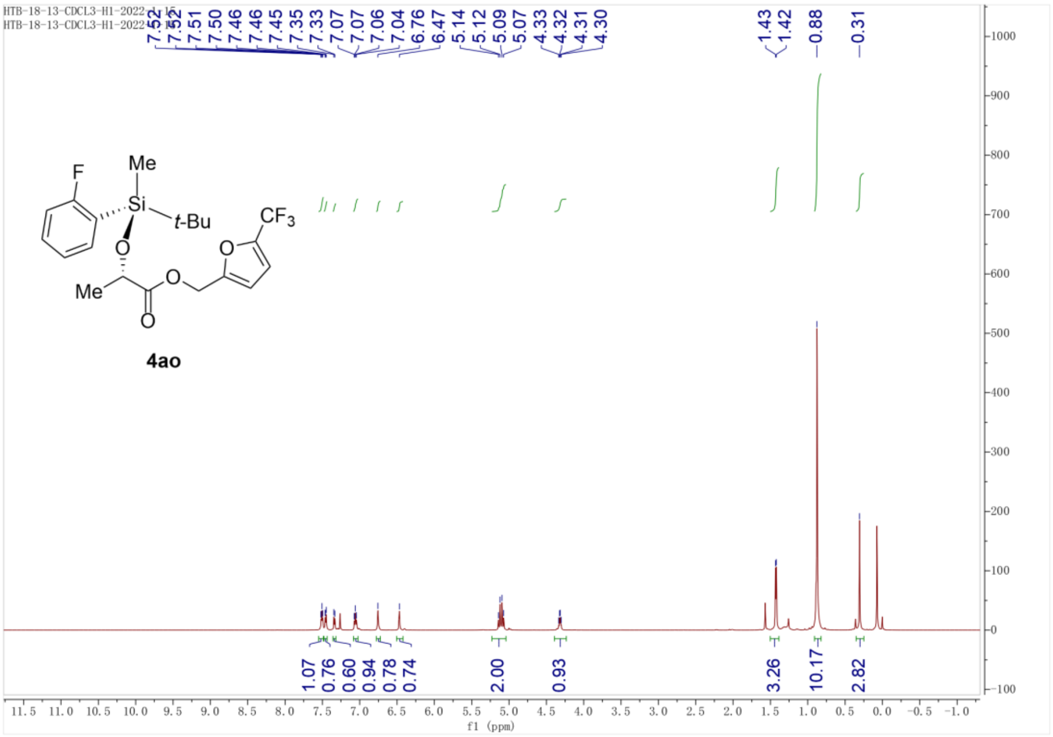

Supplementary Figure 162. ^{1}H NMR (400 M, $CDCl_3$, 25 °C) of compound **4ao**

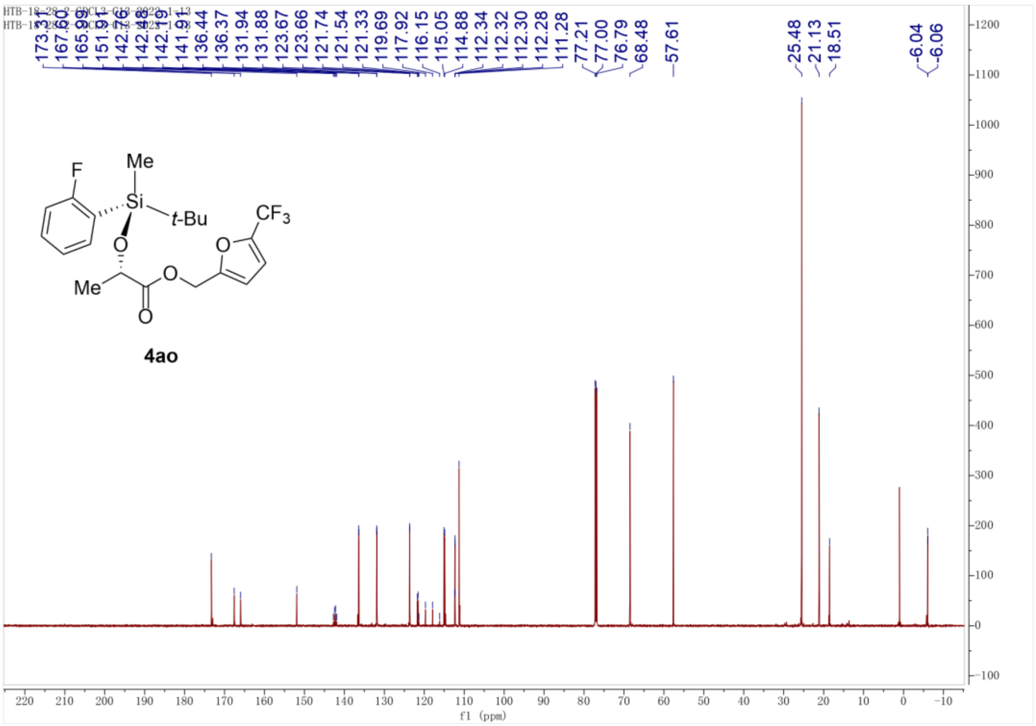

Supplementary Figure 163. ^{13}C NMR (150 M, $CDCl_3$, 25 ^oC) of compound **4ao**

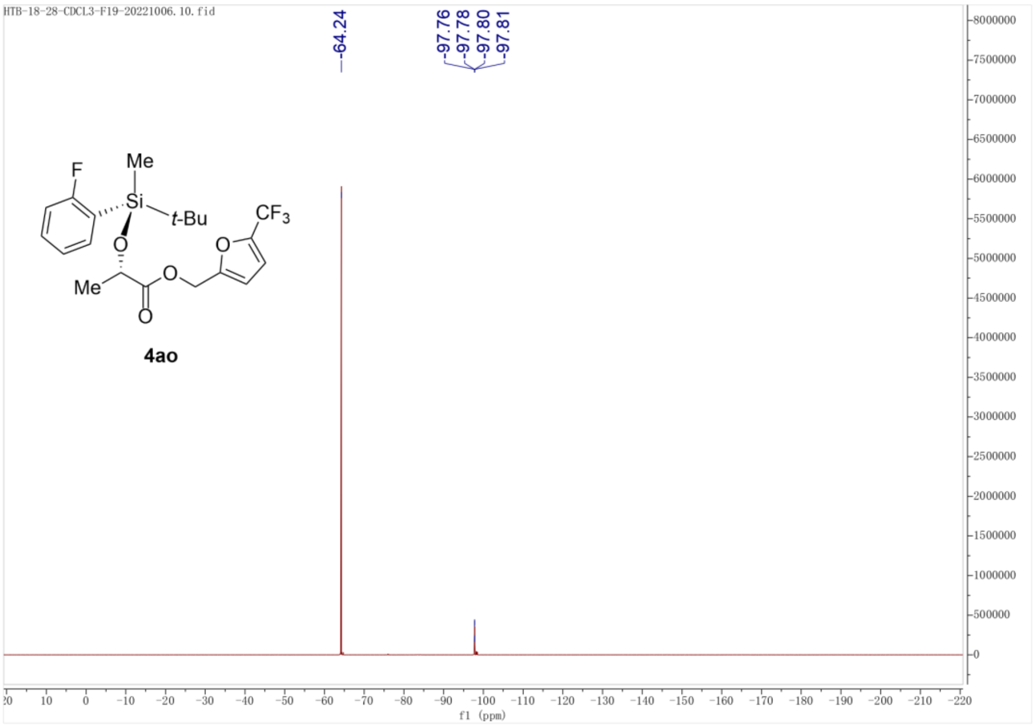

Supplementary Figure 164. ^{19}F NMR (376 M, $CDCl_3$, 25 ^oC) of compound **4ao**

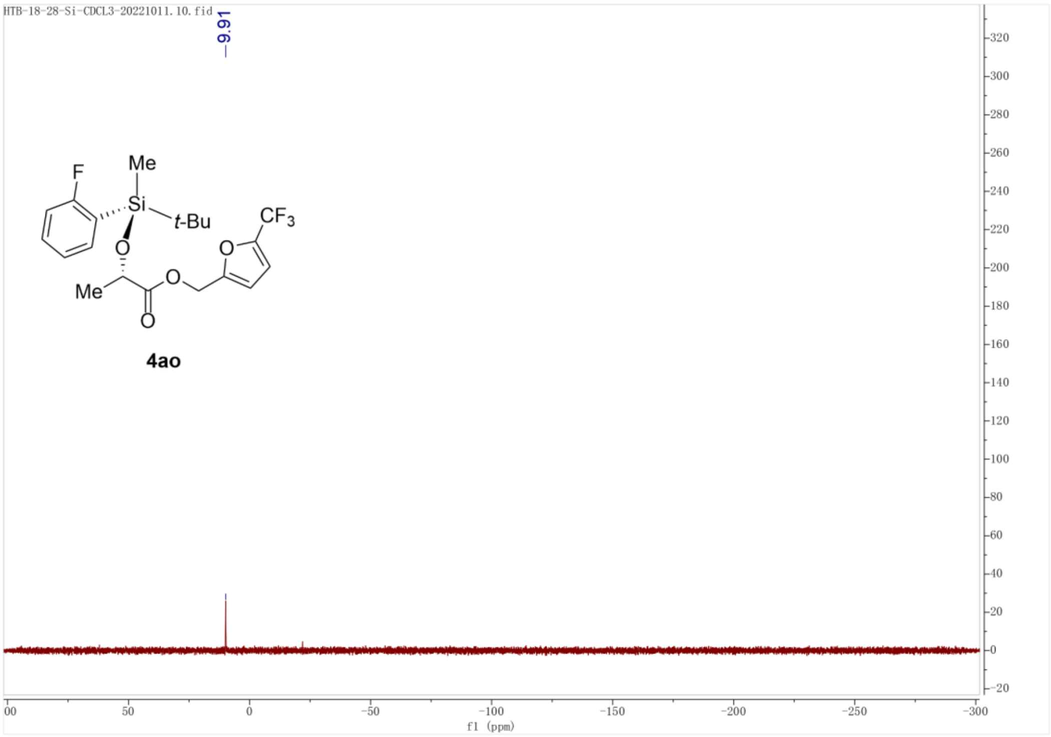

Supplementary Figure 165. ^{29}Si NMR (80 M, $CDCl_3$, 25 °C) of compound **4ao**

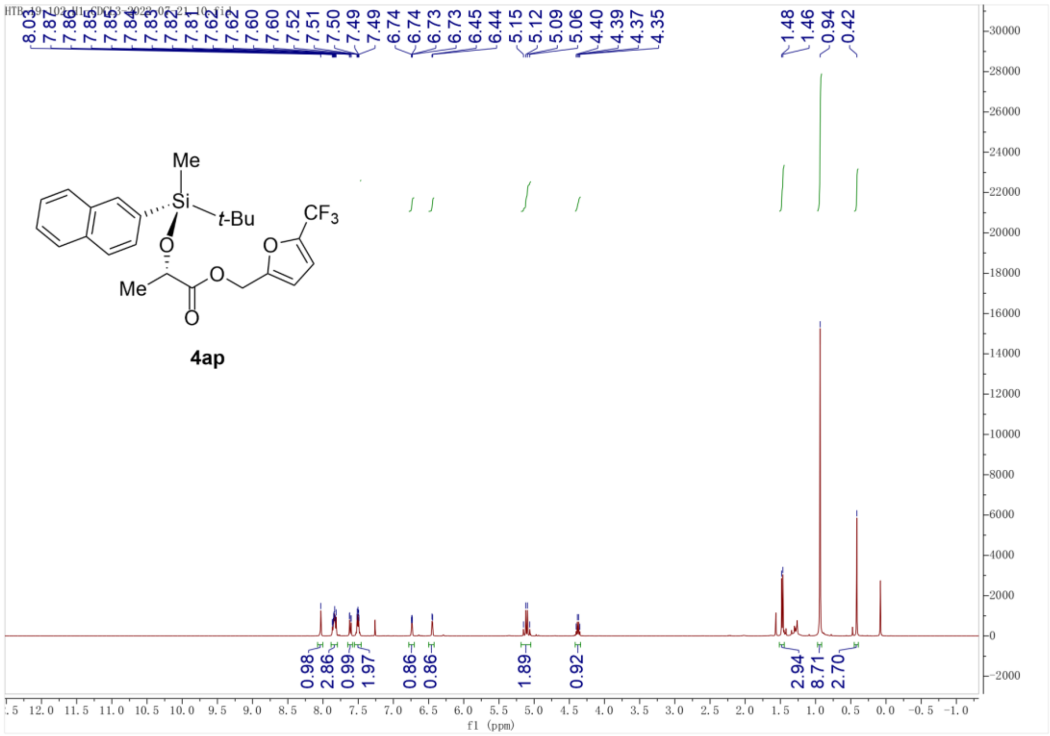

Supplementary Figure 166. ^{1}H NMR (400 M, $CDCl_3$, 25 °C) of compound **4ap**

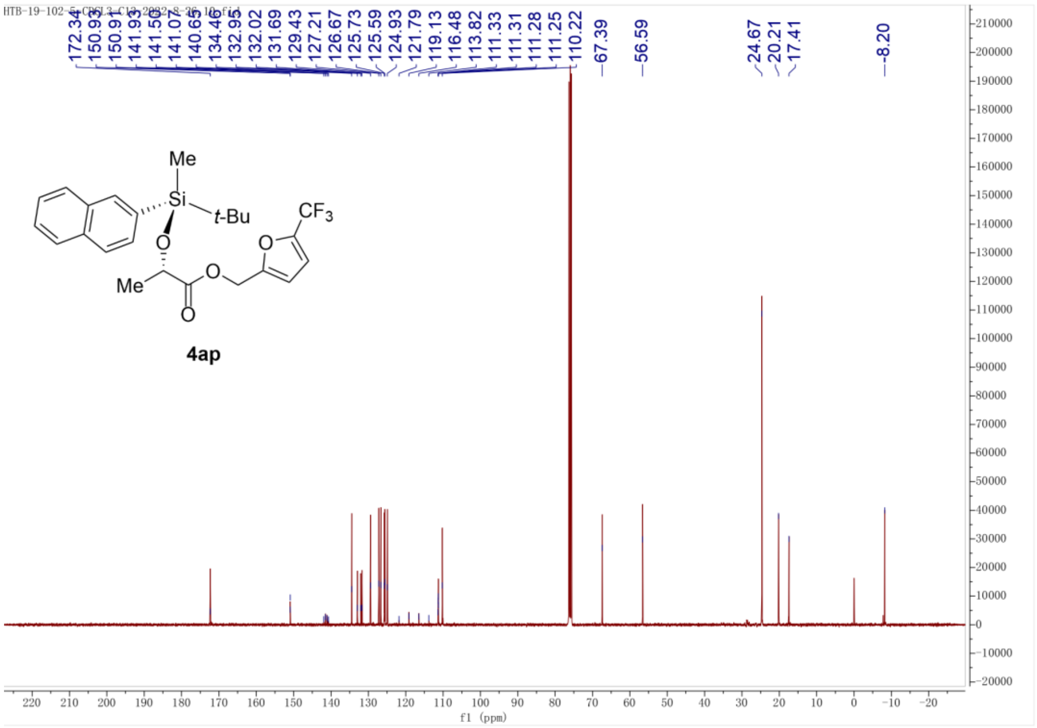

Supplementary Figure 167. ^{13}C NMR (100 M, $CDCl_3$, 25 °C) of compound **4ap**

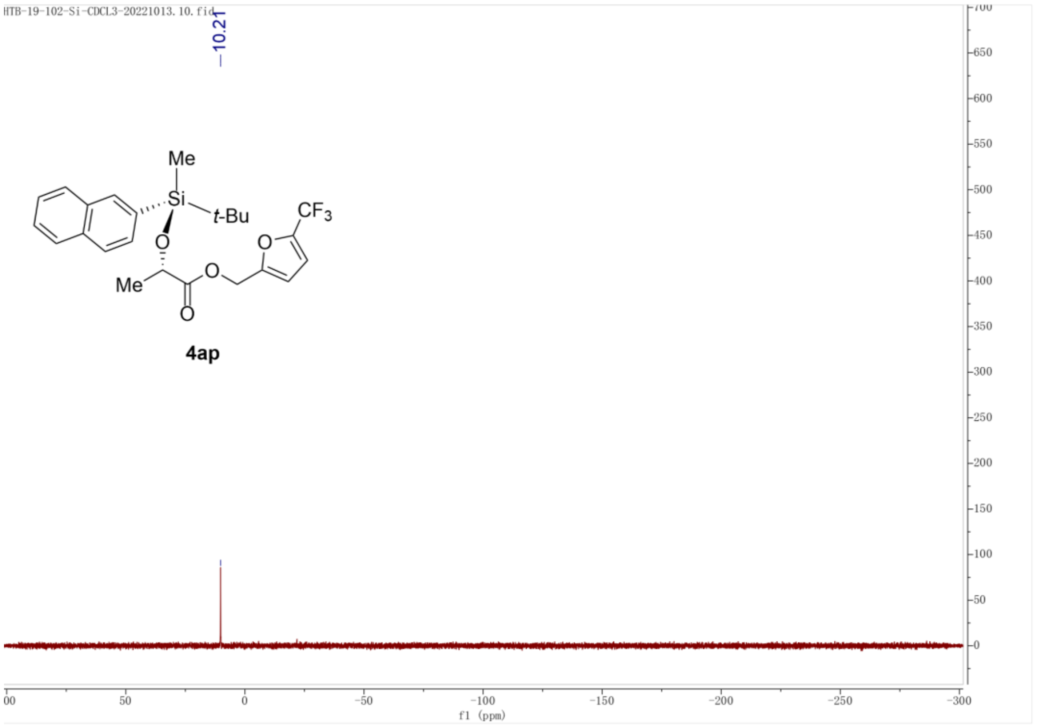

Supplementary Figure 168. ^{29}Si NMR (80 M, $CDCl_3$, 25 °C) of compound **4ap**

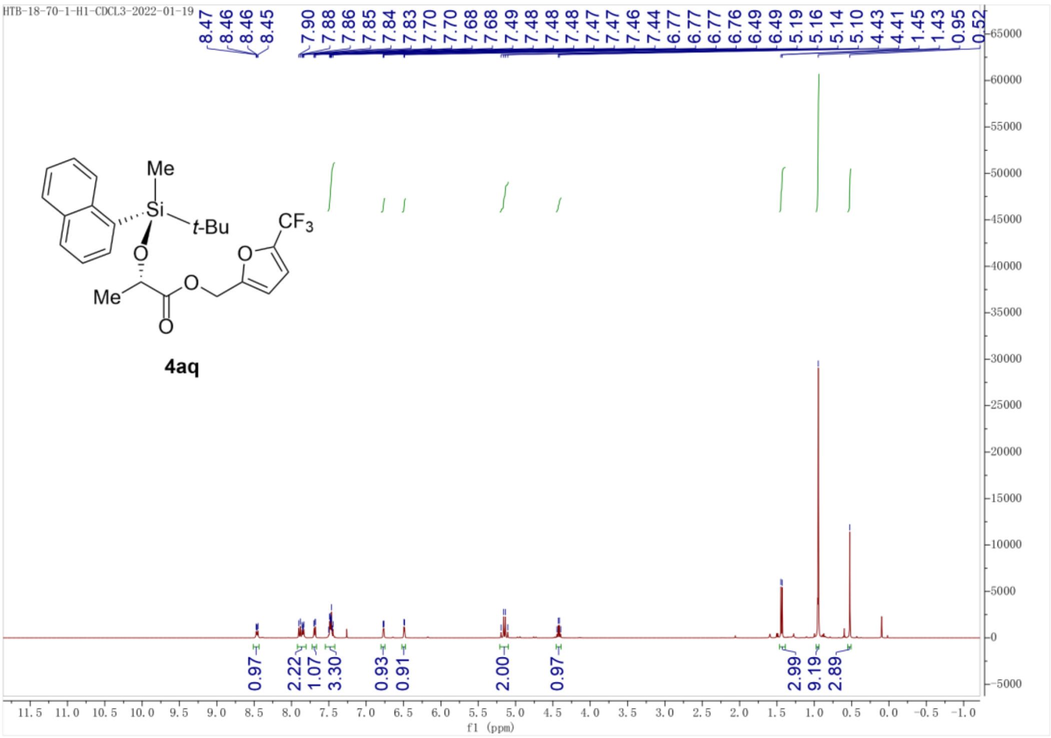

Supplementary Figure 169. ^{1}H NMR (400 M, $CDCl_3$, 25 °C) of compound **4aq**

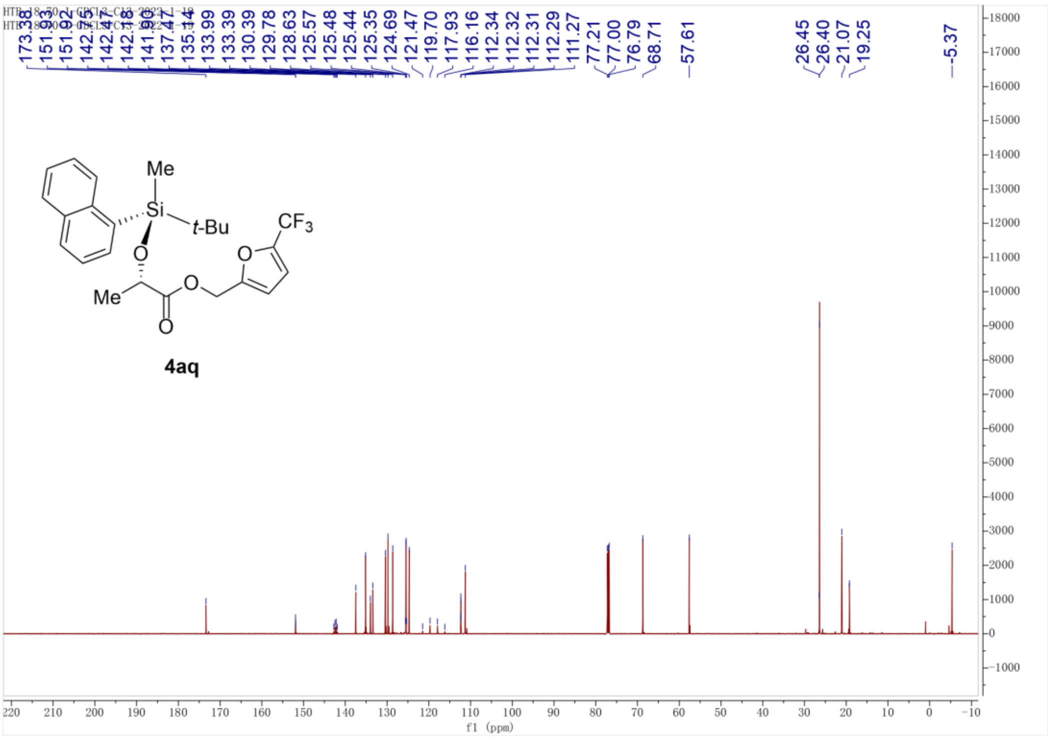

Supplementary Figure 170. ^{13}C NMR (150 M, $CDCl_3$, 25 °C) of compound **4aq**

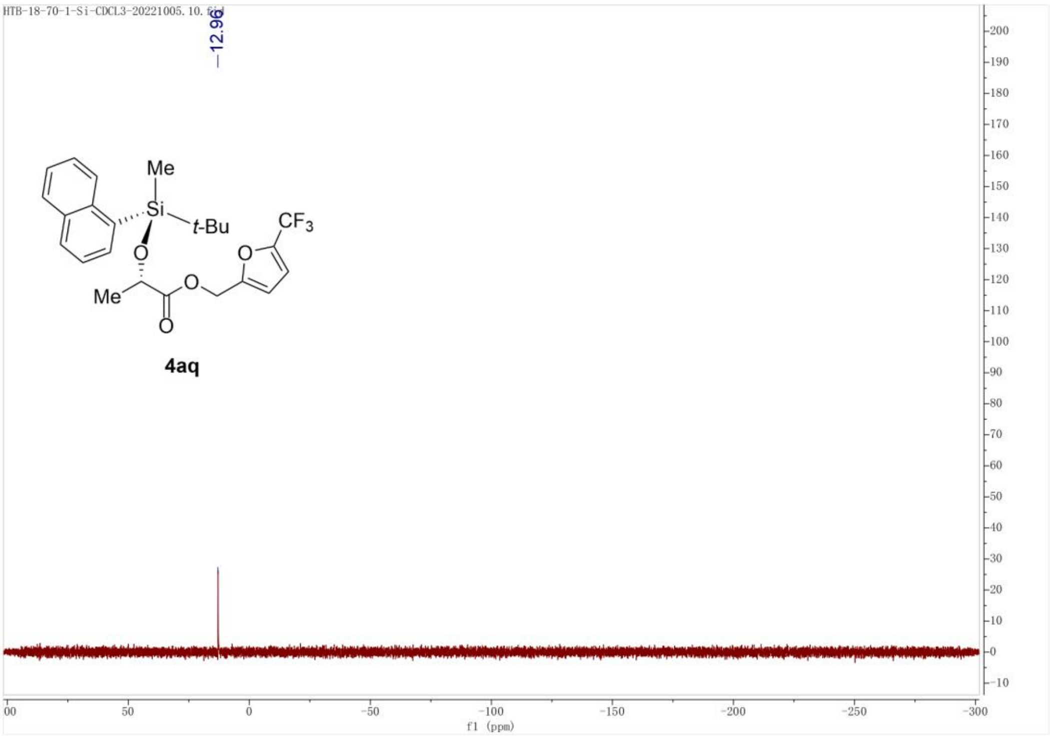

Supplementary Figure 171. ^{29}Si NMR (80 M, $CDCl_3$, 25 ^{o}C) of compound **4aq**

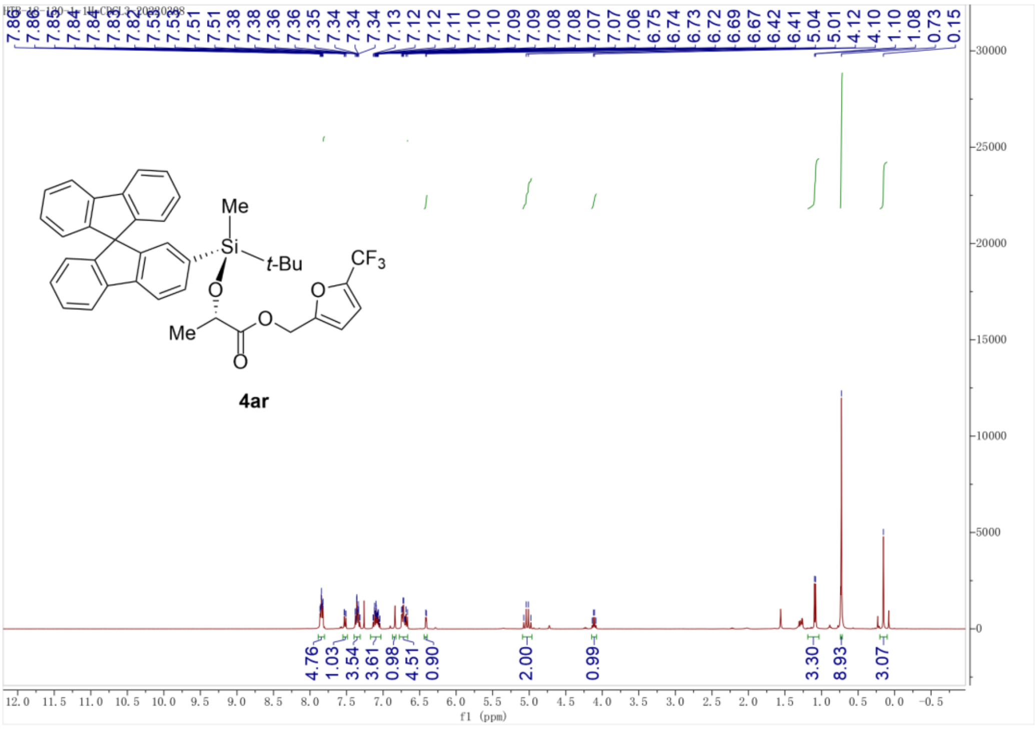

Supplementary Figure 172. ^{1}H NMR (400 M, $CDCl_3$, 25 ^{o}C) of compound **4ar**

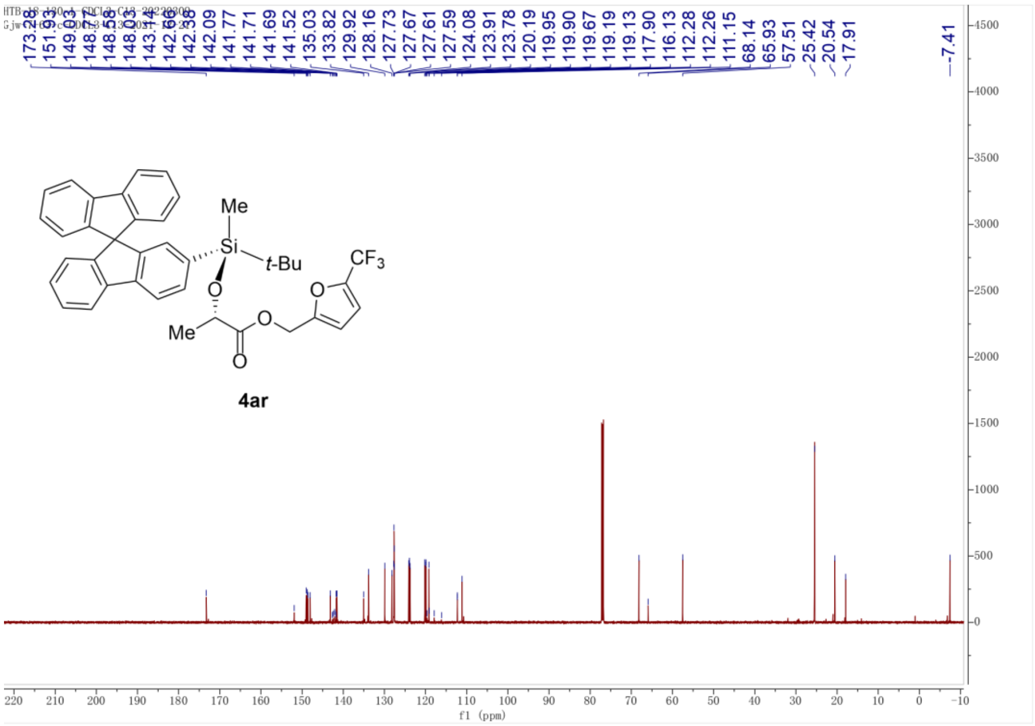

Supplementary Figure 173. ^{13}C NMR (150 M, $CDCl_3$, 25 °C) of compound **4ar**

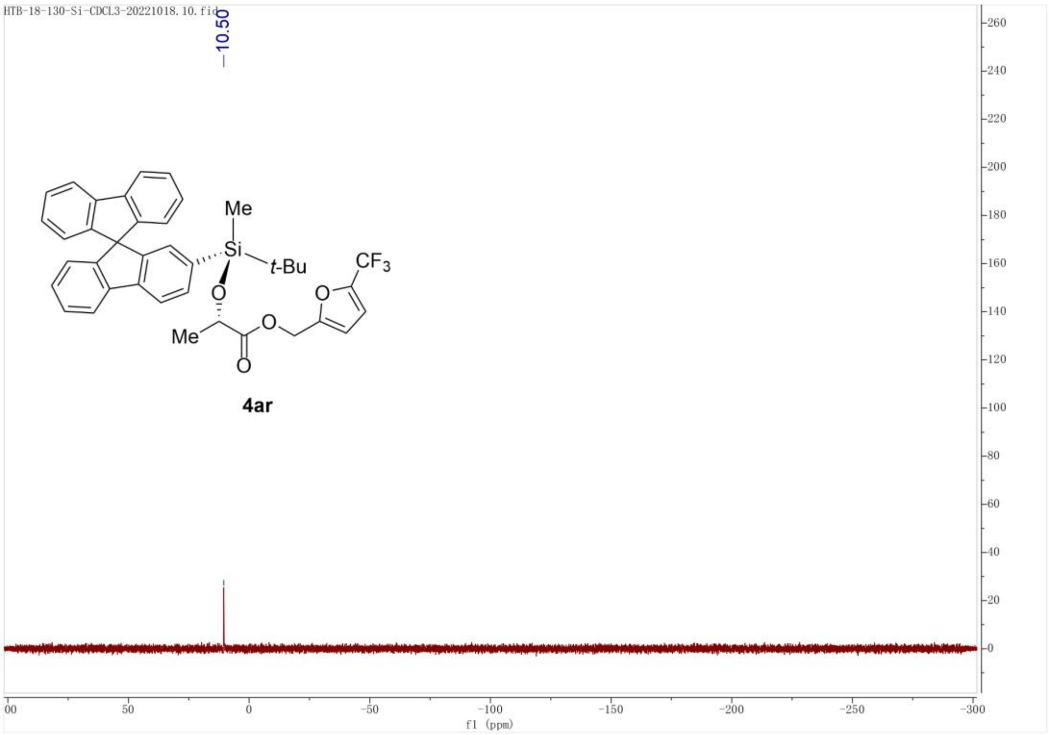

Supplementary Figure 174. ^{29}Si NMR (80 M, $CDCl_3$, 25 °C) of compound **4ar**

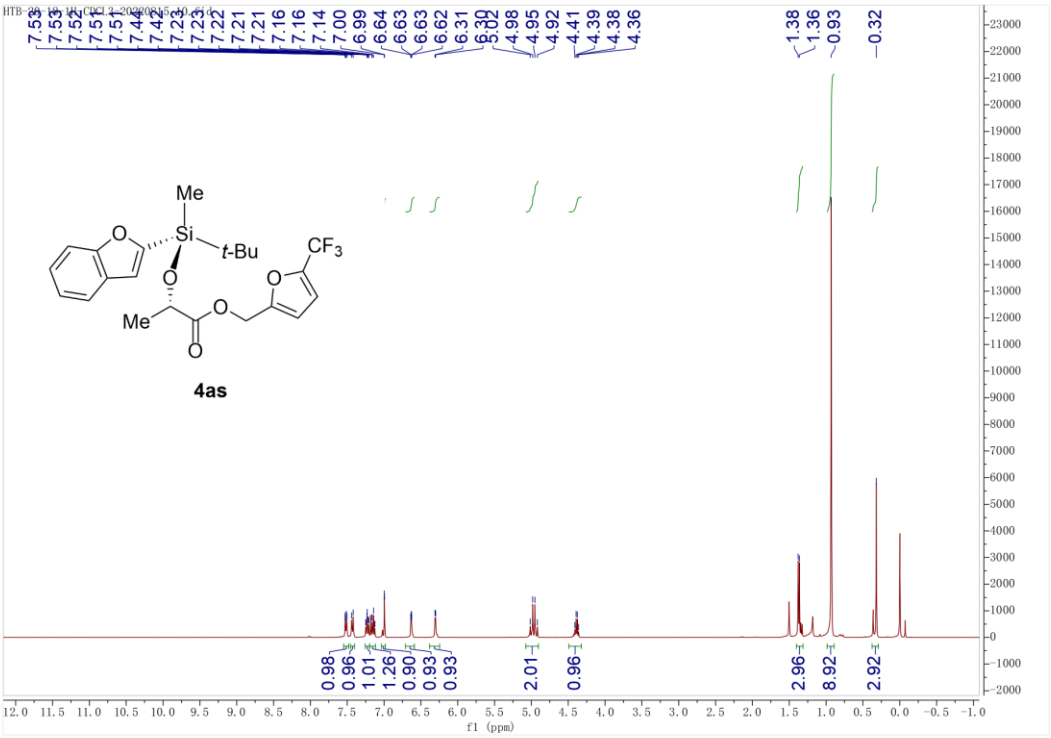

Supplementary Figure 175. ^{1}H NMR (400 M, $CDCl_3$, 25 ^{o}C) of compound **4as**

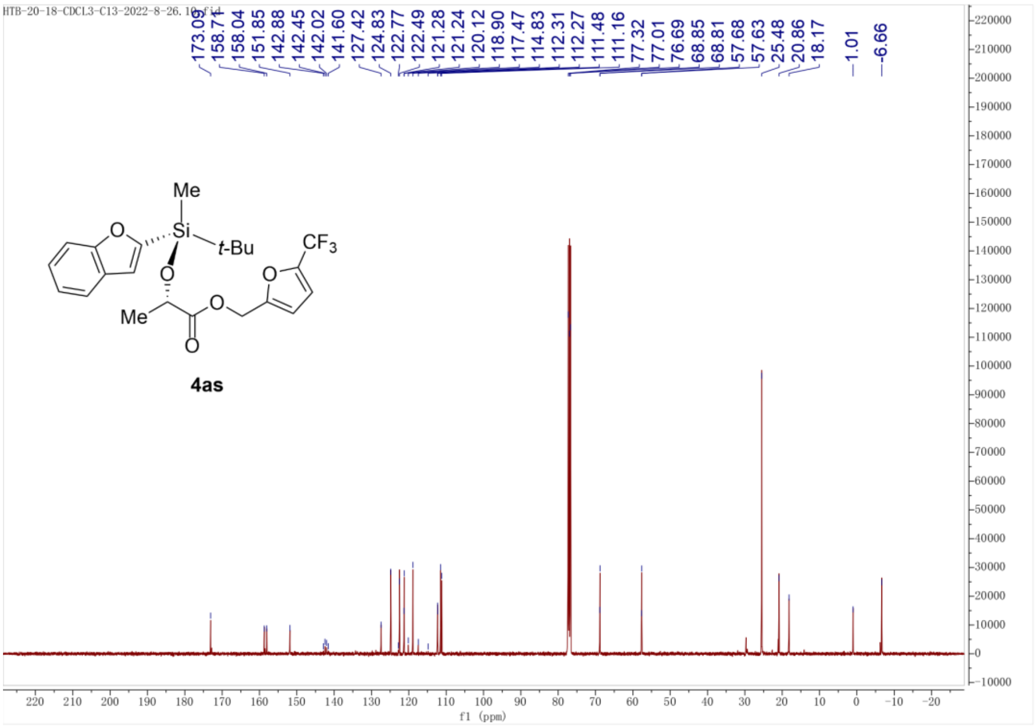

Supplementary Figure 176. ^{13}C NMR (100 M, $CDCl_3$, 25 ^{o}C) of compound **4as**

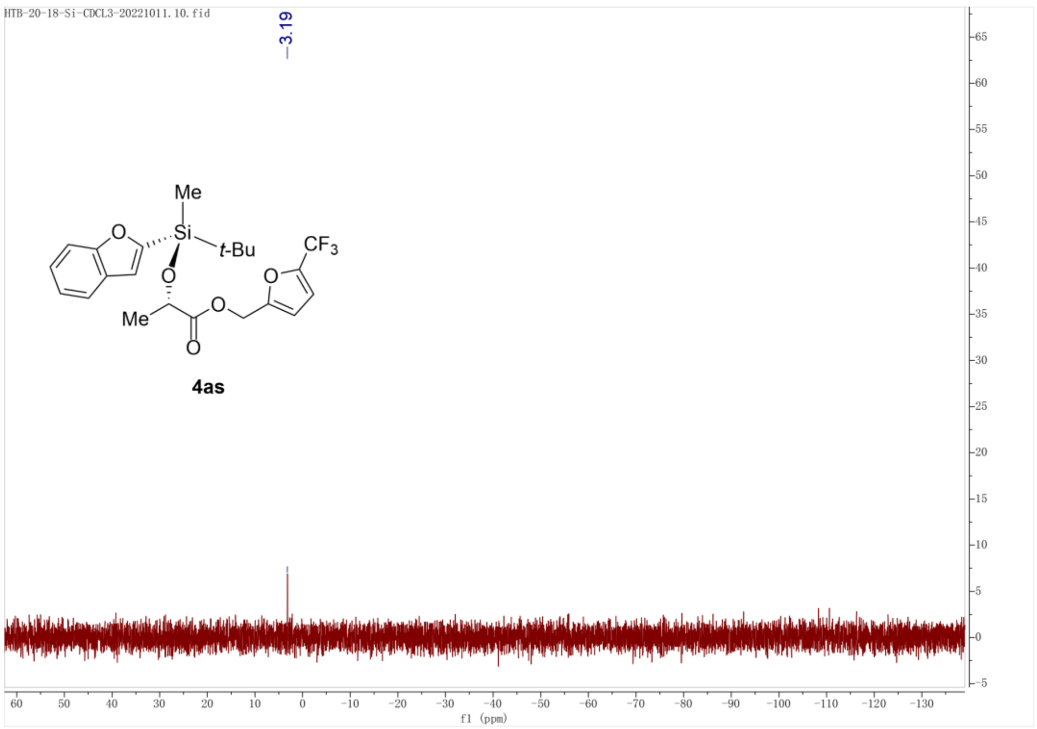

Supplementary Figure 177. ^{29}Si NMR (80 M, $CDCl_3$, 25 °C) of compound **4as**

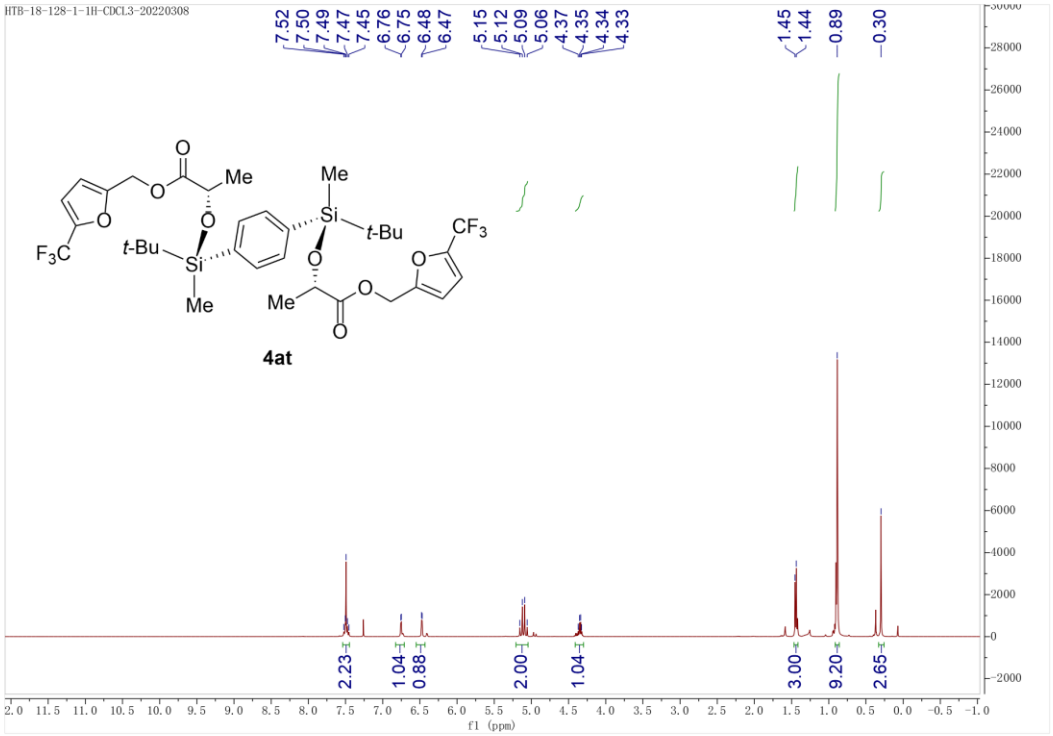

Supplementary Figure 178. ^{1}H NMR (400 M, $CDCl_3$, 25 °C) of compound **4at**

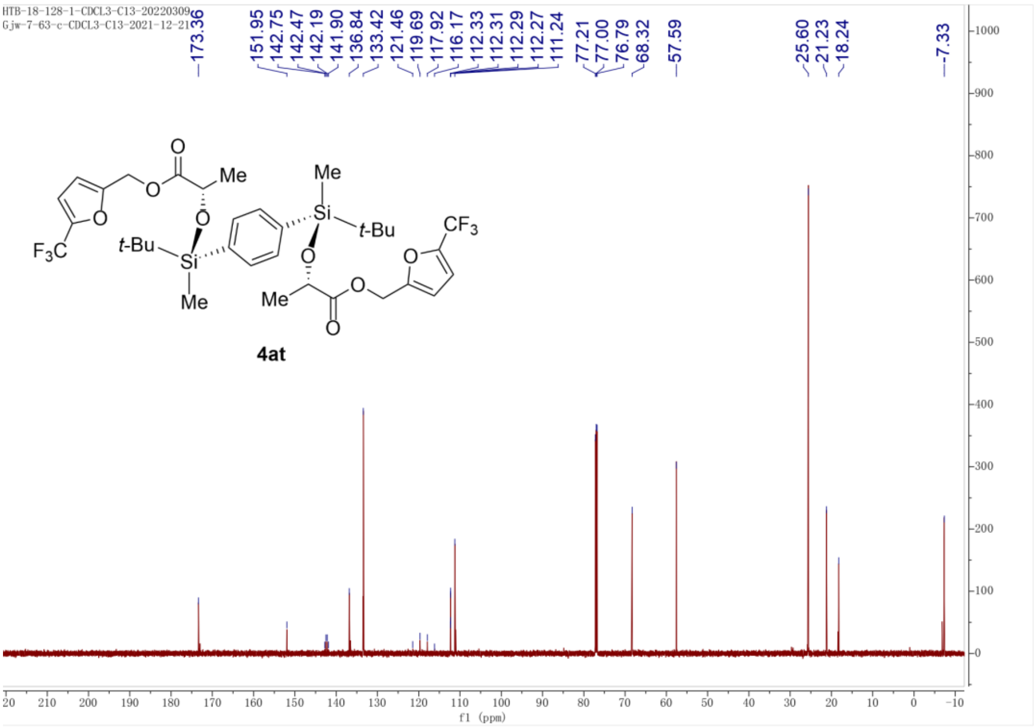

Supplementary Figure 179. ^{13}C NMR (100 M, $CDCl_3$, 25 °C) of compound **4at**

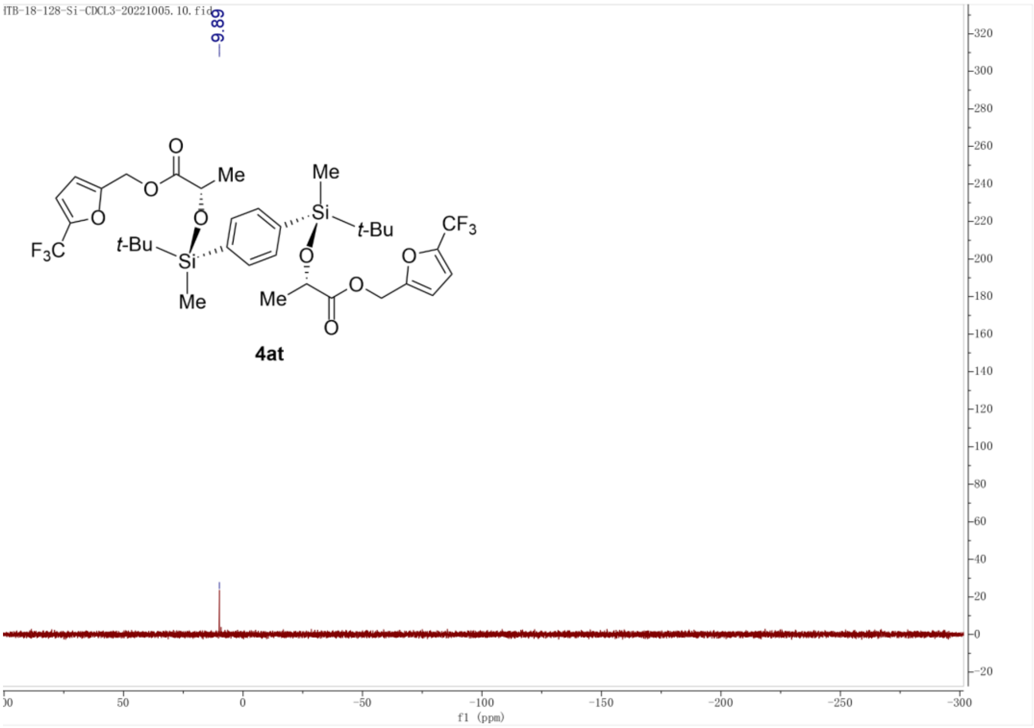

Supplementary Figure 180. ^{29}Si NMR (80 M, $CDCl_3$, 25 °C) of compound **4at**

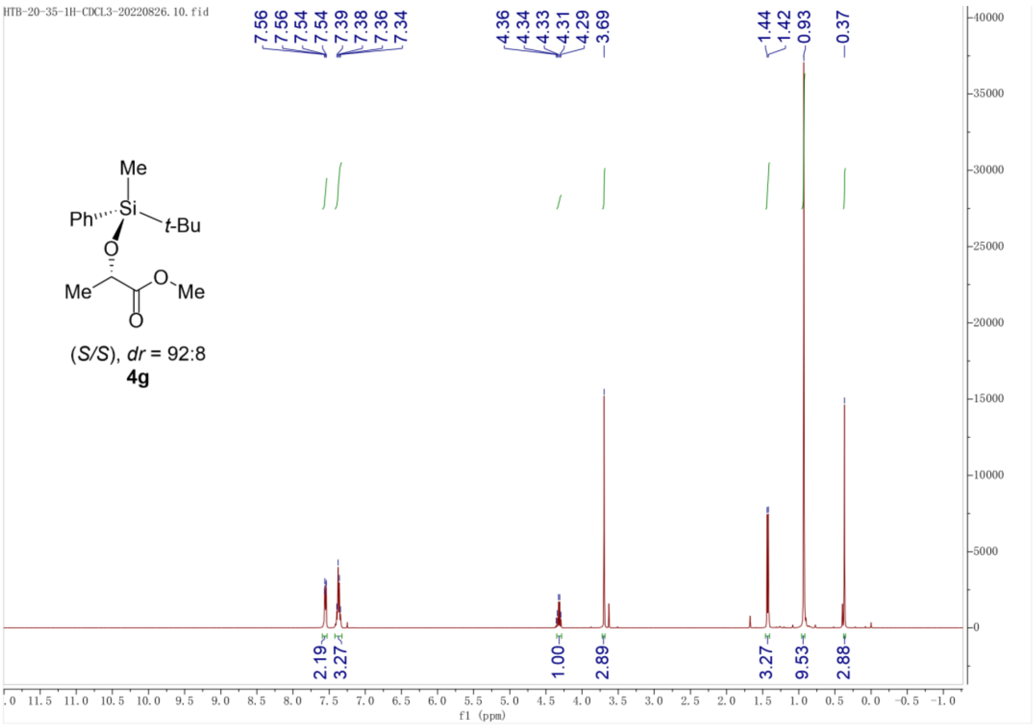

Supplementary Figure 181. ^{1}H NMR (400 M, $CDCl_3$, 25 °C) of compound **4g**

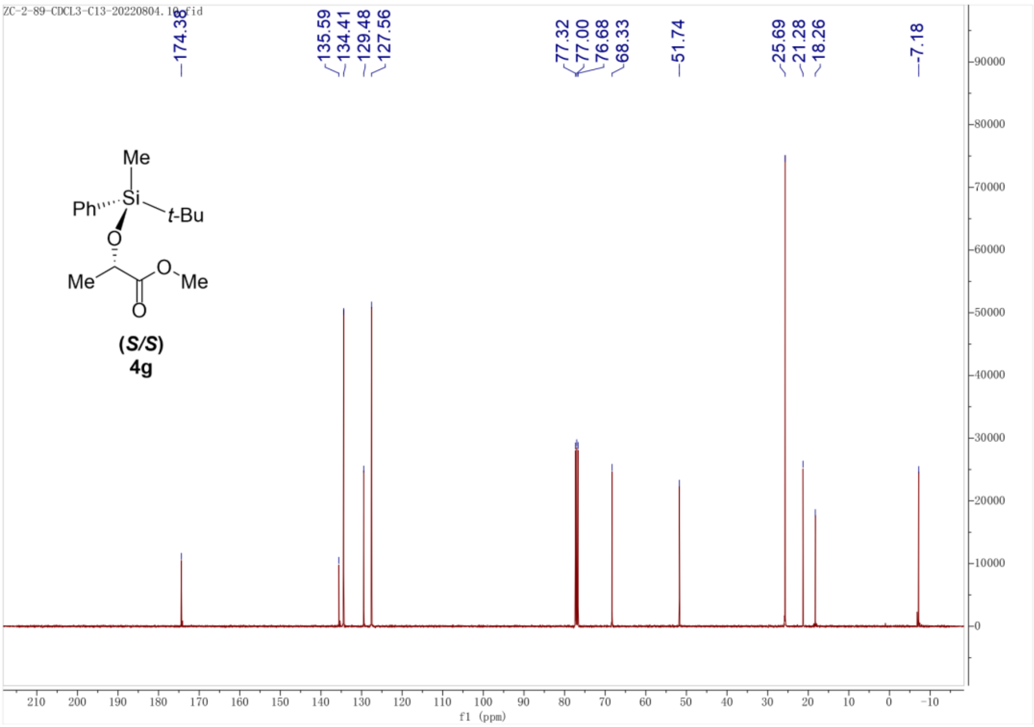

Supplementary Figure 182. ^{13}C NMR (100 M, $CDCl_3$, 25 °C) of compound **4g**

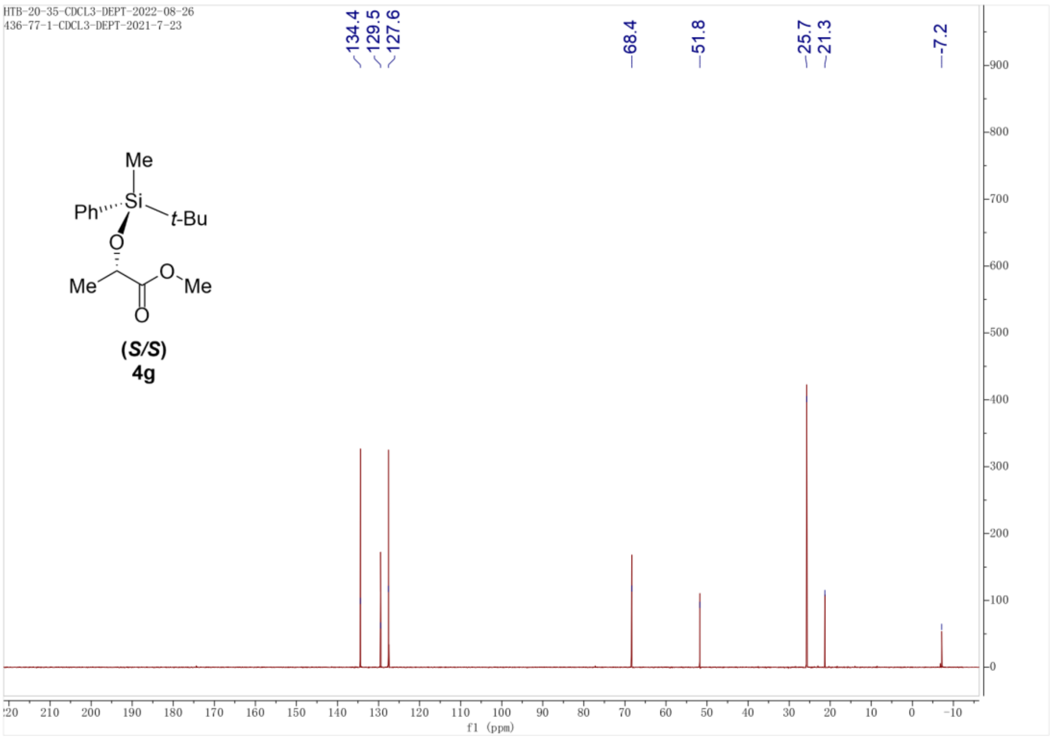

Supplementary Figure 183. DEPT NMR (100 M, $CDCl_3$, 25 °C) of compound **4g**

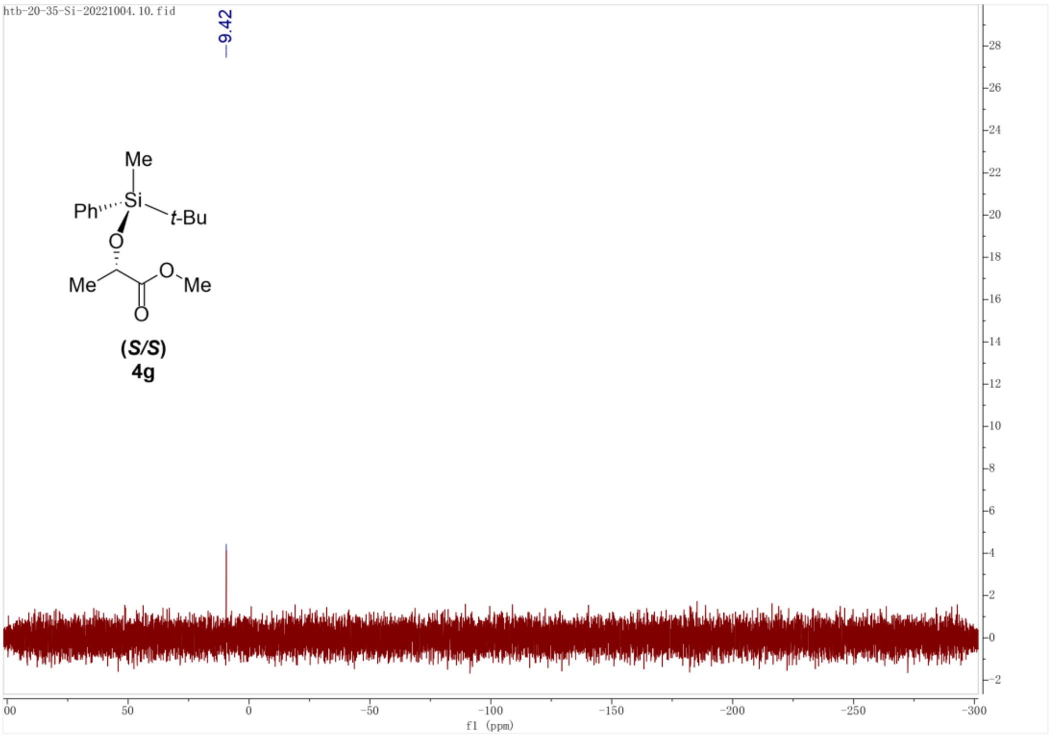

Supplementary Figure 184. ^{29}Si NMR (80 M, $CDCl_3$, 25 °C) of compound **4g**

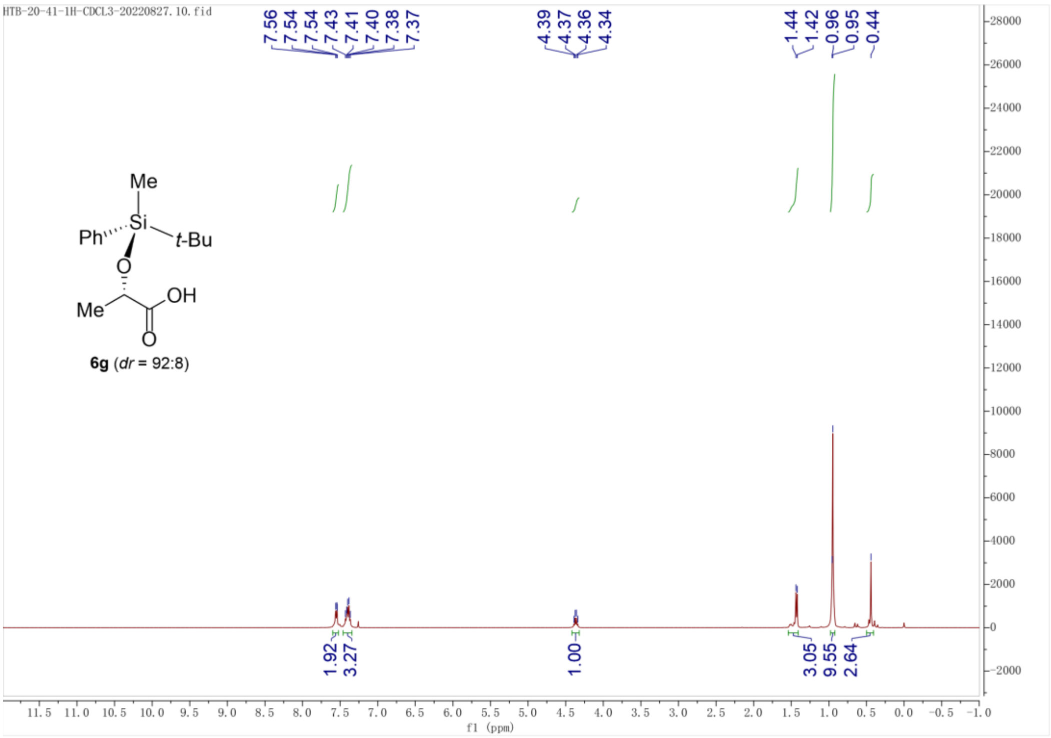

Supplementary Figure 185. ^{1}H NMR (400 M, $CDCl_3$, 25 ^{o}C) of compound **6g**

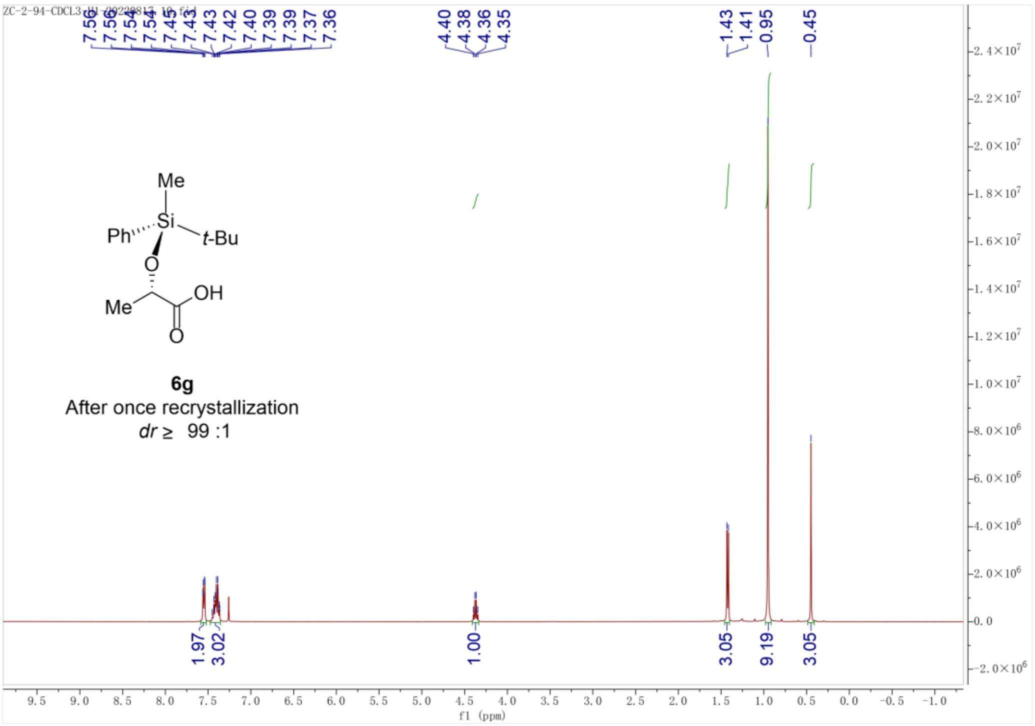

Supplementary Figure 186. ^{1}H NMR (400 M, $CDCl_3$, 25 ^{o}C) of compound **6g**

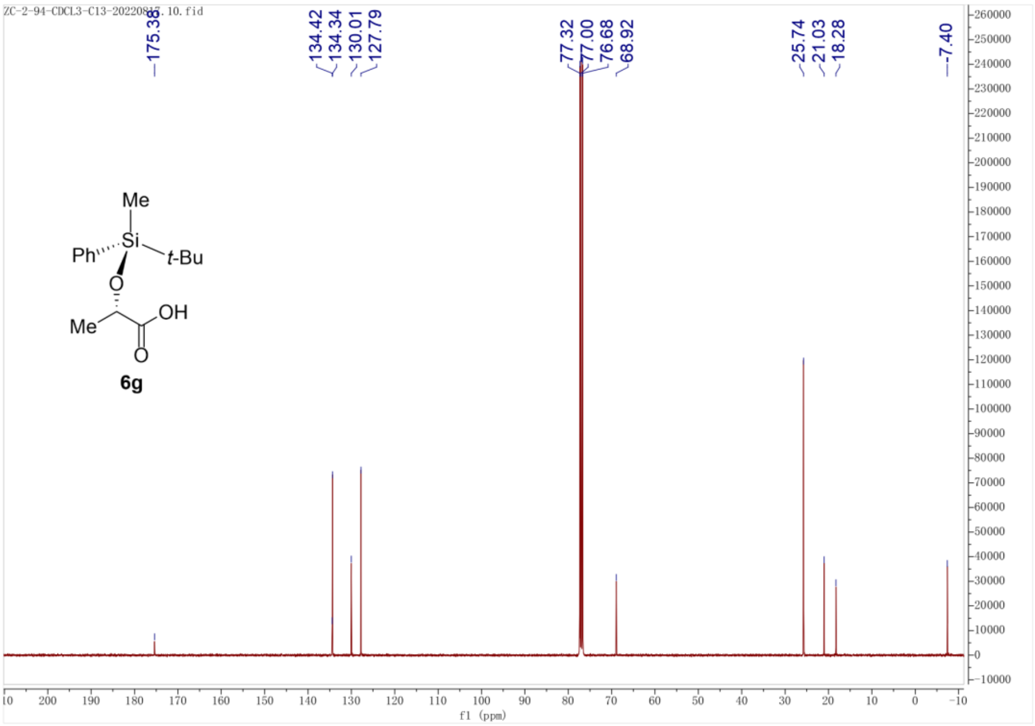

Supplementary Figure 187. ^{13}C NMR (100 M, $CDCl_3$, 25 °C) of compound **6g**

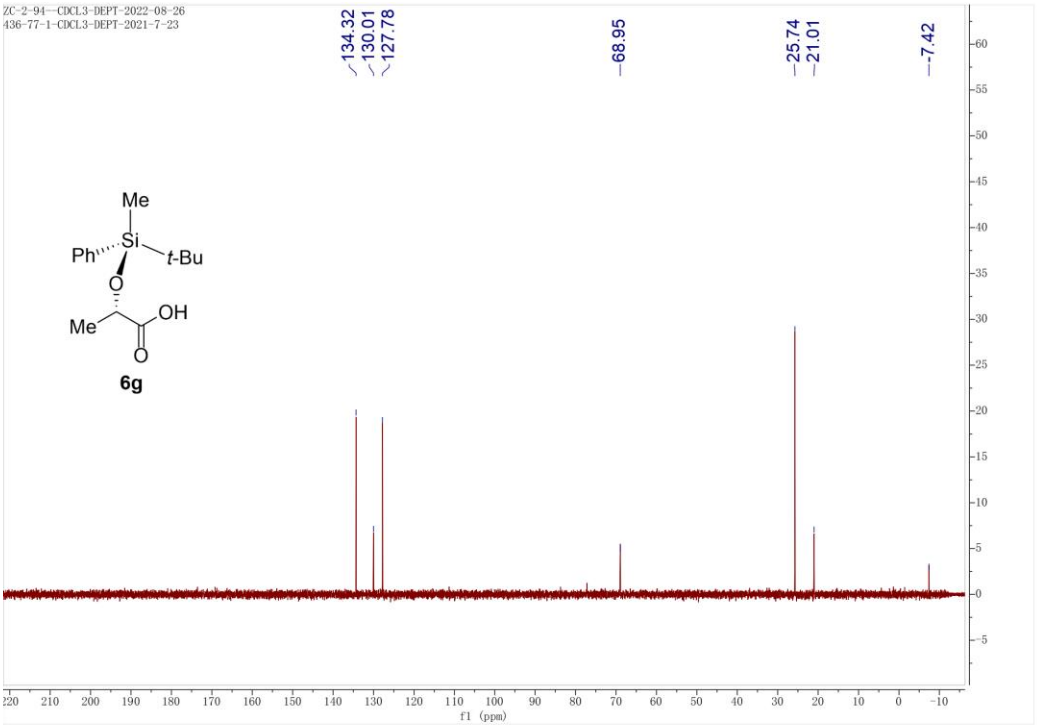

Supplementary Figure 188. DEPT NMR (100 M, $CDCl_3$, 25 °C) of compound **6g**

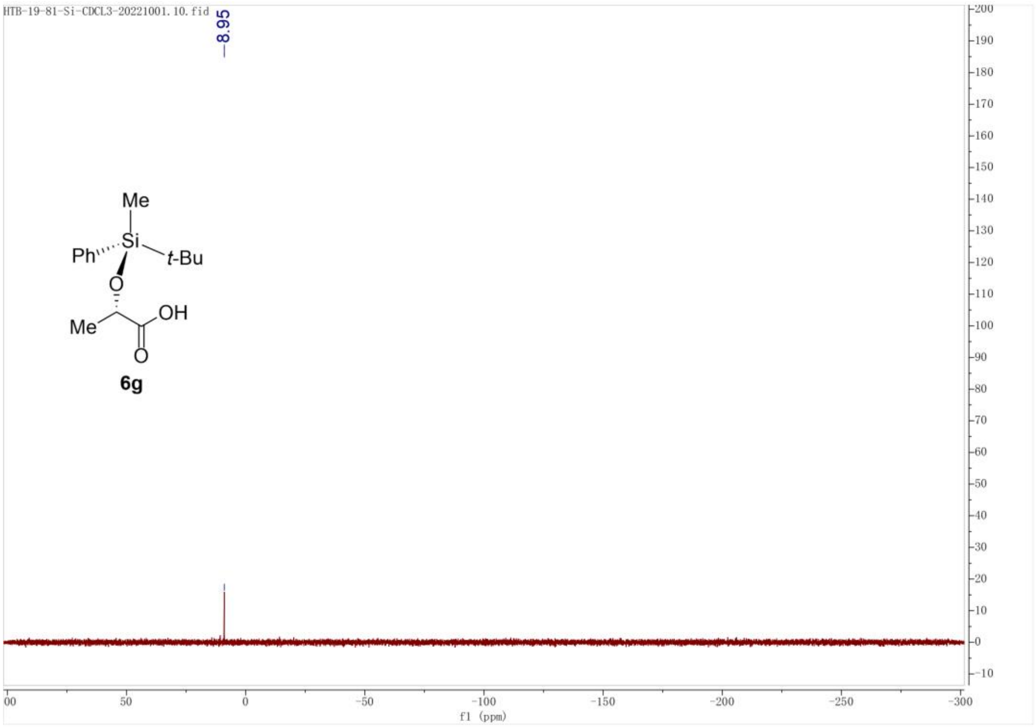

Supplementary Figure 189. ^{29}Si NMR (80 M, $CDCl_3$, 25 °C) of compound **6g**

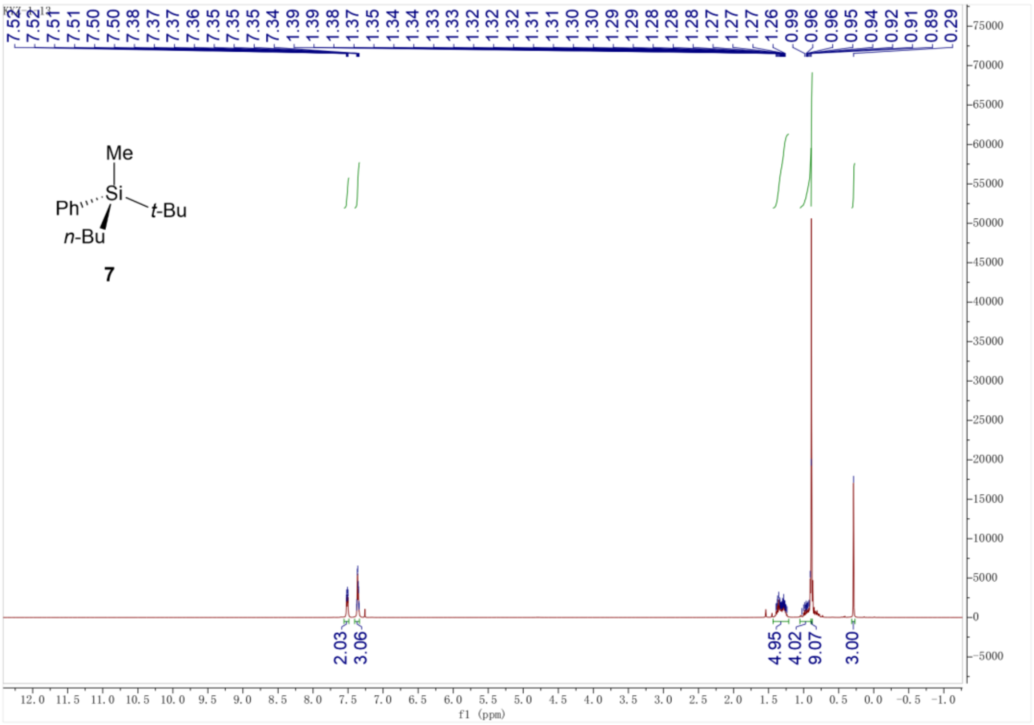

Supplementary Figure 190. ^{1}H NMR (400 M, $CDCl_3$, 25 °C) of compound **7**

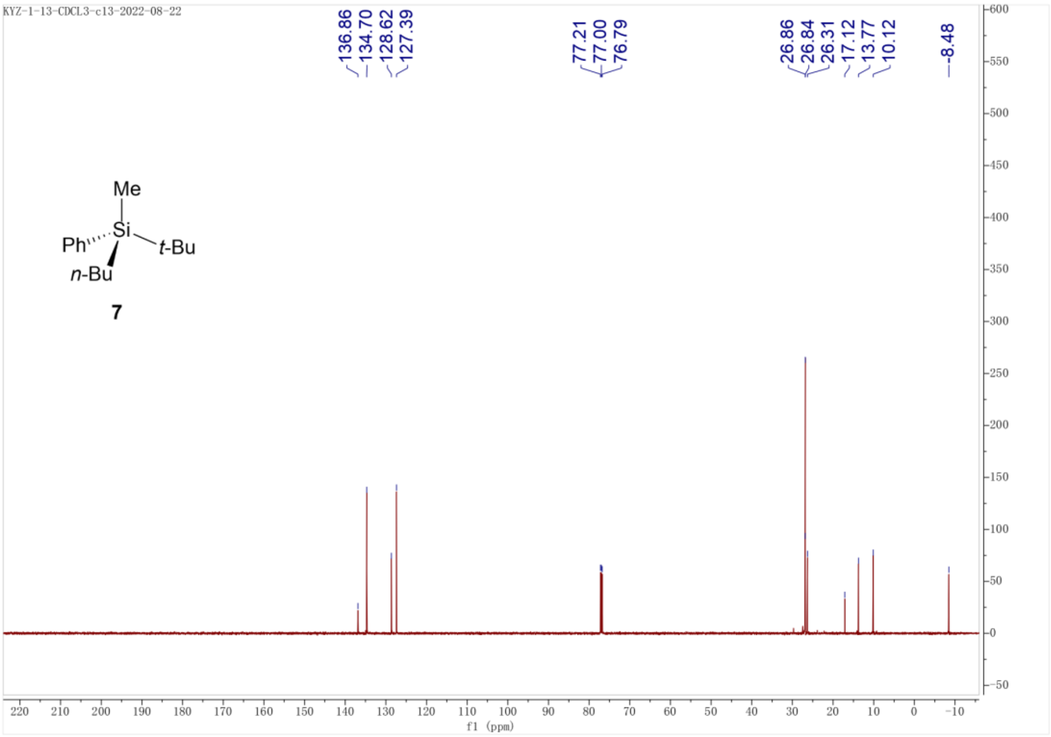

Supplementary Figure 191. ^{13}C NMR (150 M, $CDCl_3$, 25 ^{o}C) of compound **7**

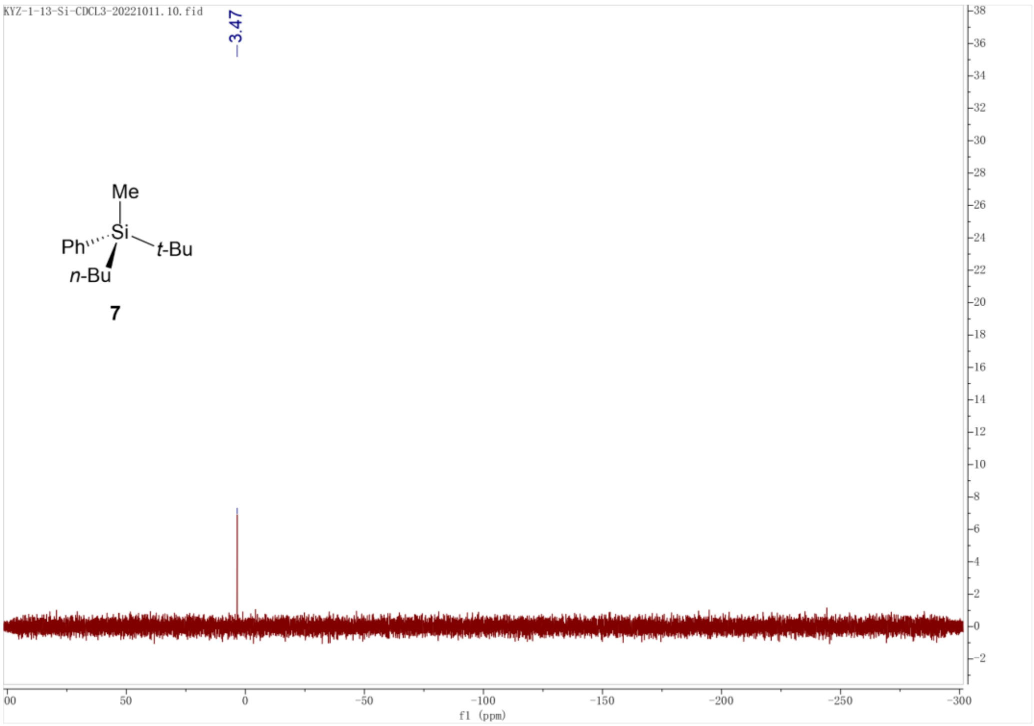

Supplementary Figure 192. ^{29}Si NMR (80 M, $CDCl_3$, 25 ^{o}C) of compound **7**

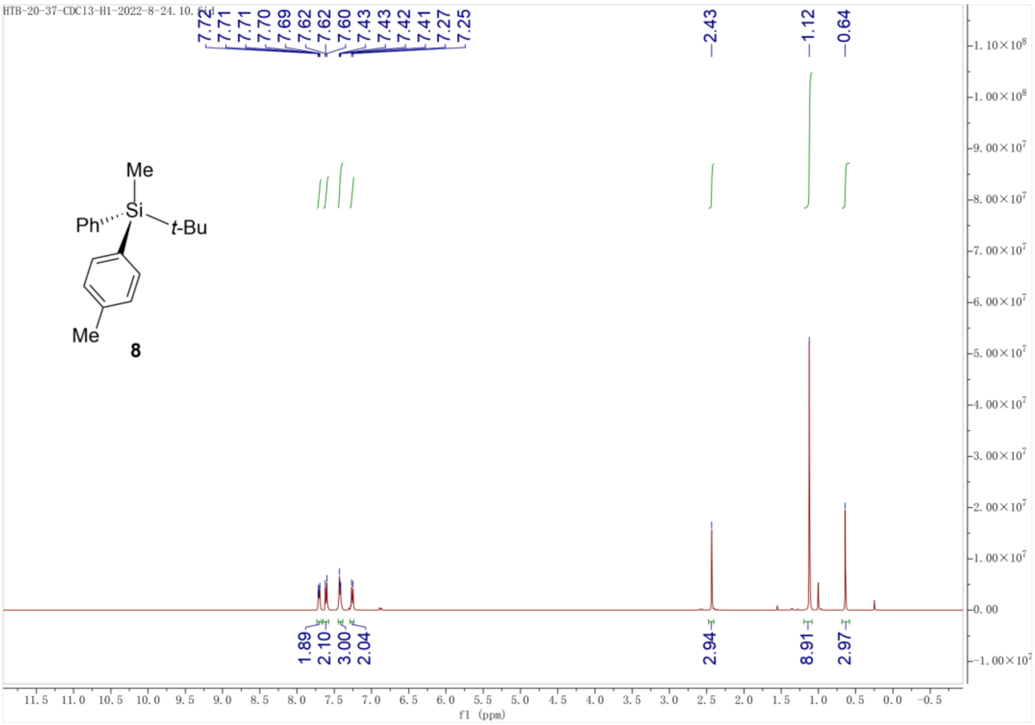

Supplementary Figure 193. ^{1}H NMR (400 M, $CDCl_3$, 25 °C) of compound **8**

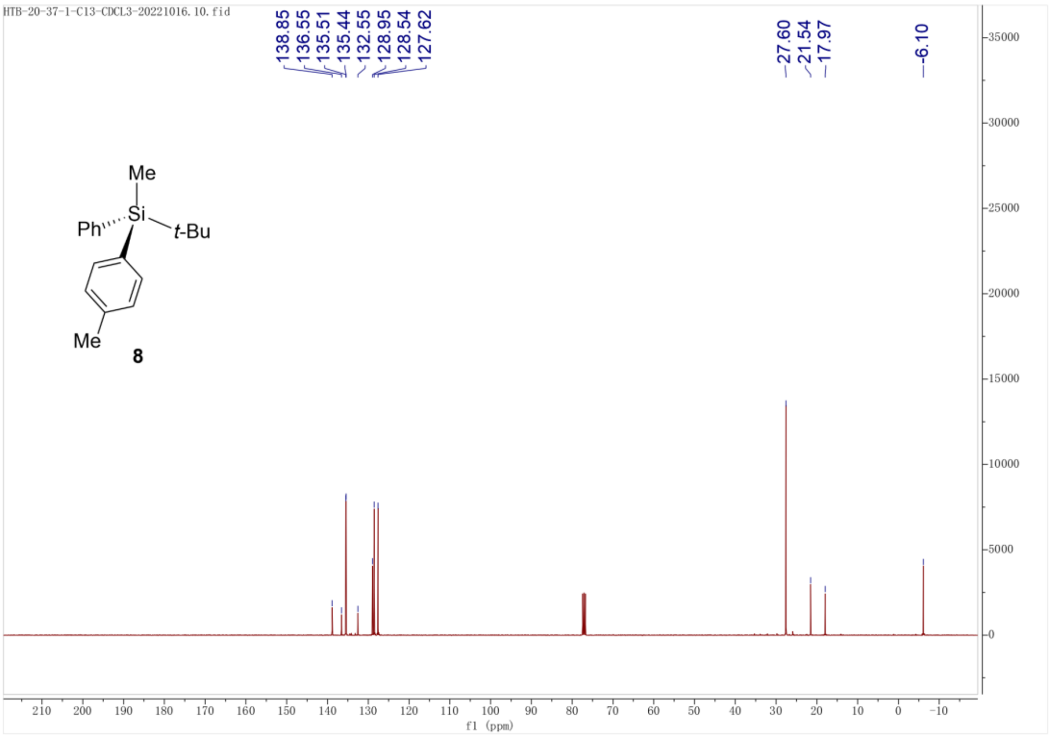

Supplementary Figure 194. ^{13}C NMR (100 M, $CDCl_3$, 25 °C) of compound **8**

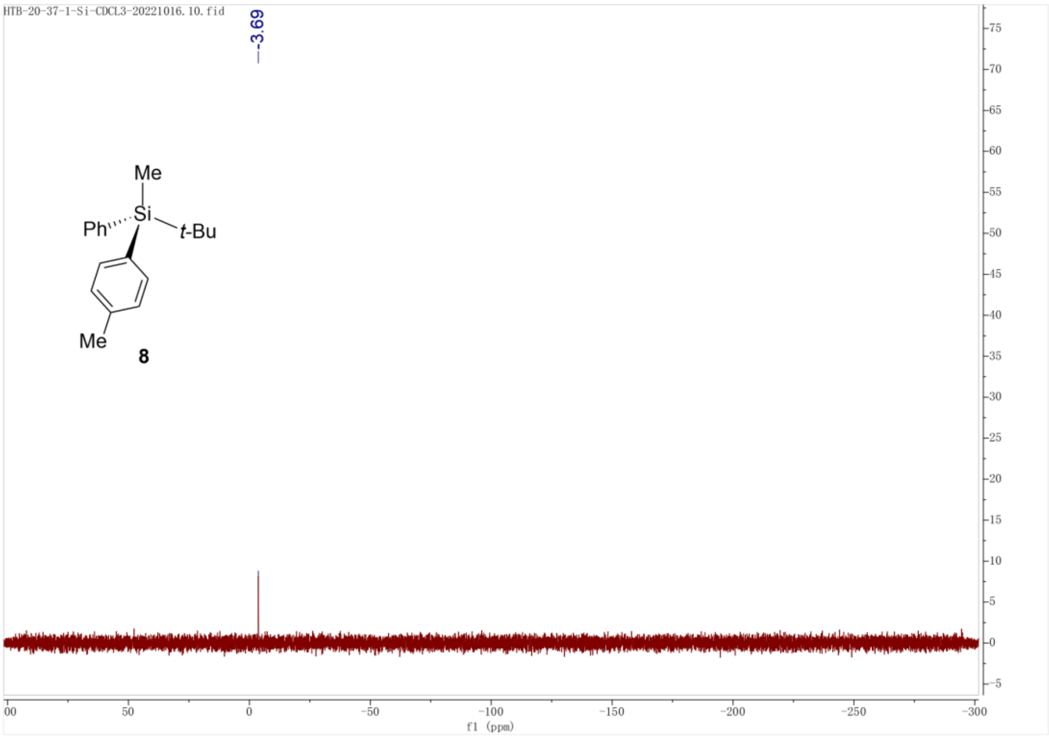

Supplementary Figure 195. ^{29}Si NMR (80 M, $CDCl_3$, 25 ^{o}C) of compound **8**

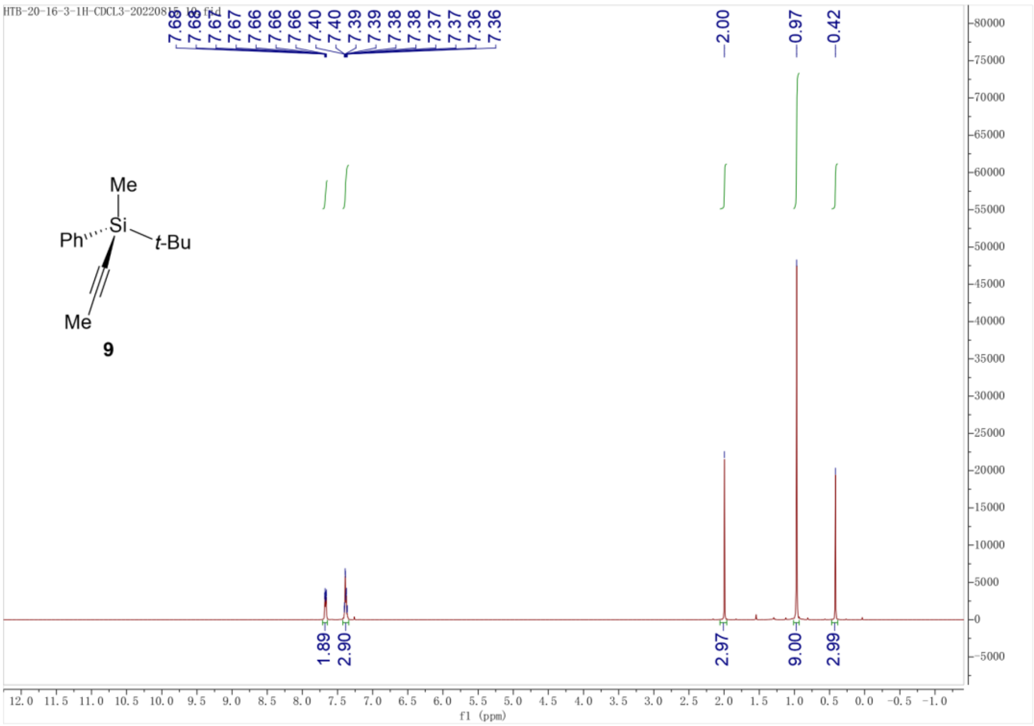

Supplementary Figure 196. ^{1}H NMR (400 M, $CDCl_3$, 25 ^{o}C) of compound **9**

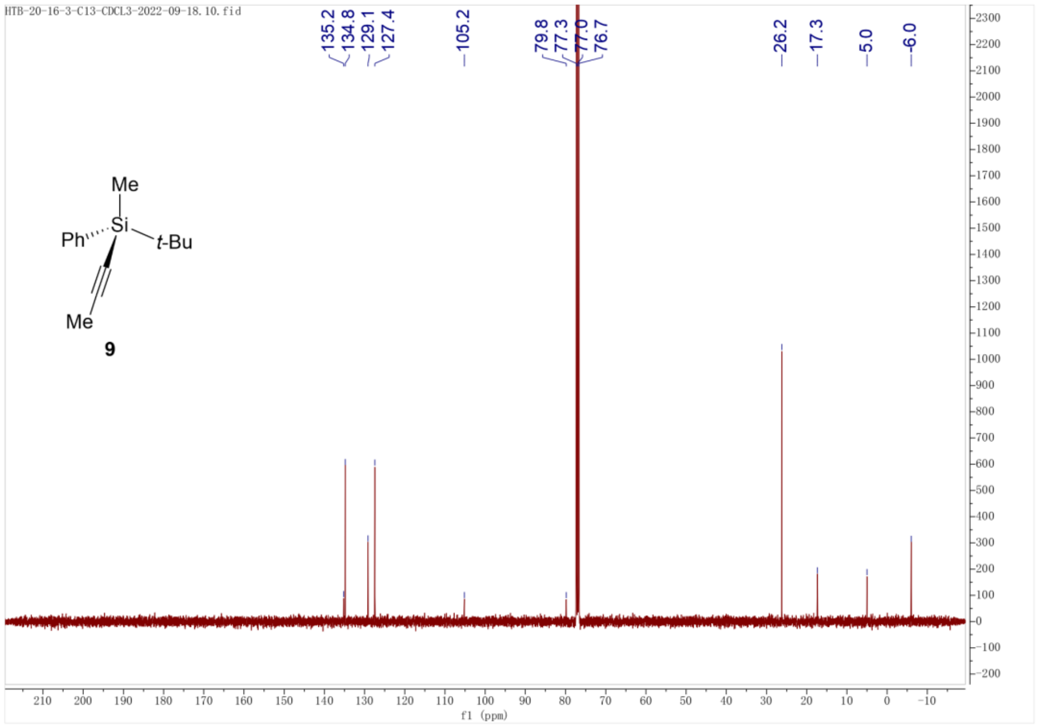

Supplementary Figure 197. ^{13}C NMR (100 M, $CDCl_3$, 25 ^{o}C) of compound **9**

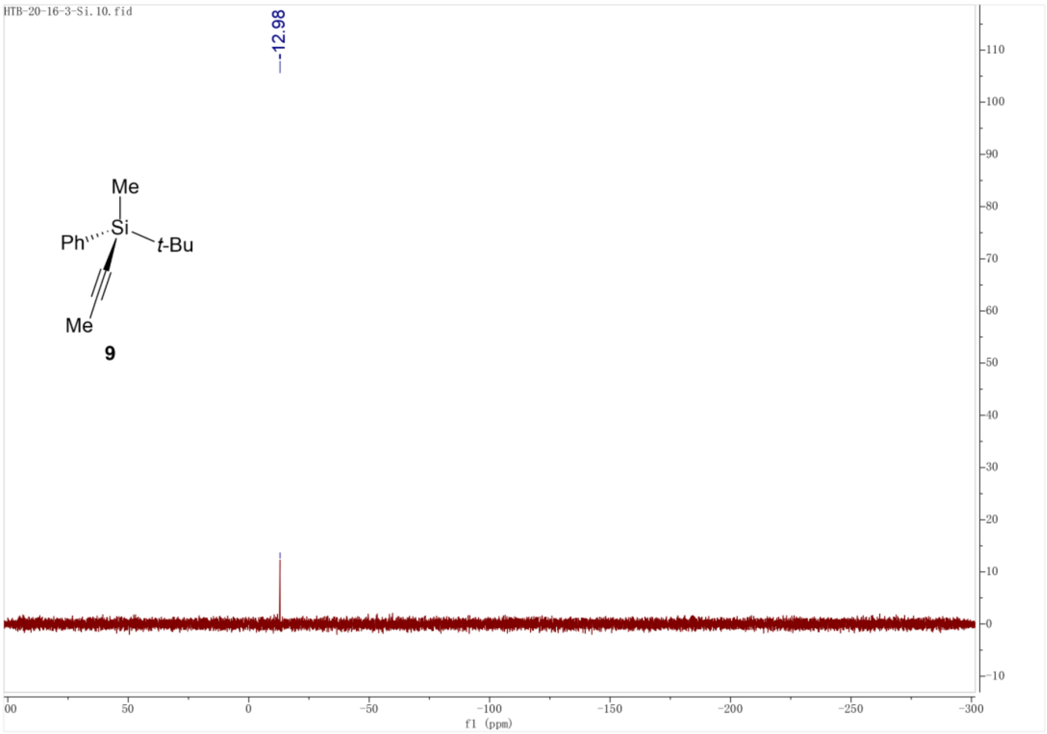

Supplementary Figure 198. ^{29}Si NMR (80 M, $CDCl_3$, 25 ^{o}C) of compound **9**

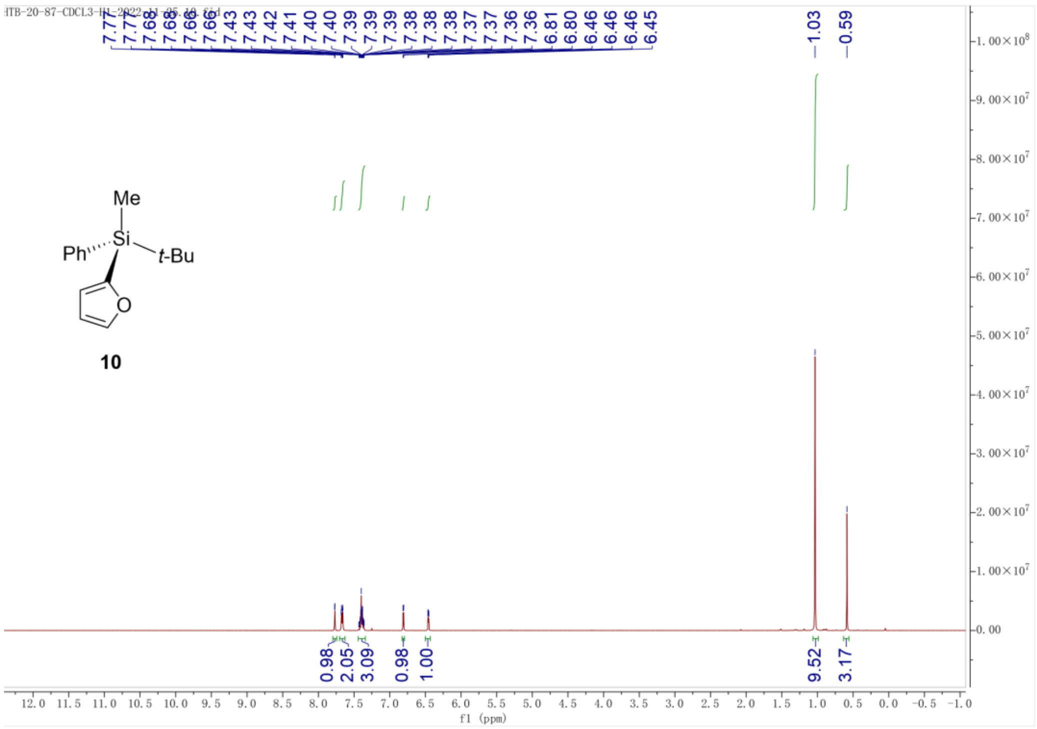

Supplementary Figure 199. ^{1}H NMR (400 M, $CDCl_3$, 25 °C) of compound **10**

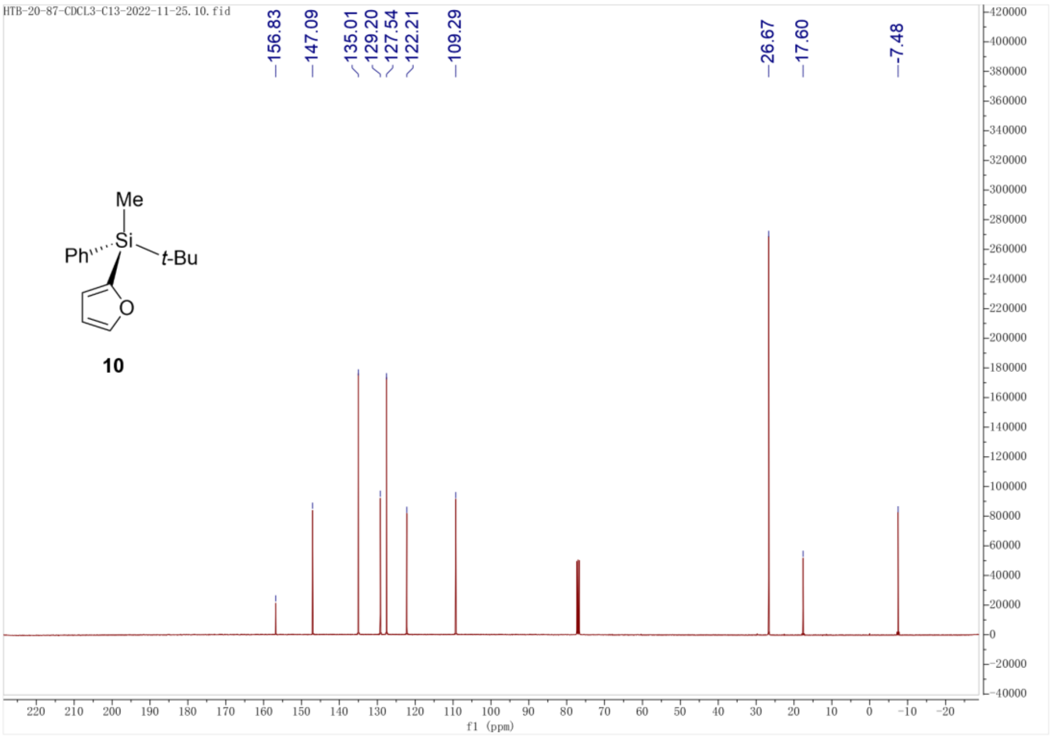

Supplementary Figure 200. ^{13}C NMR (100 M, $CDCl_3$, 25 °C) of compound **10**

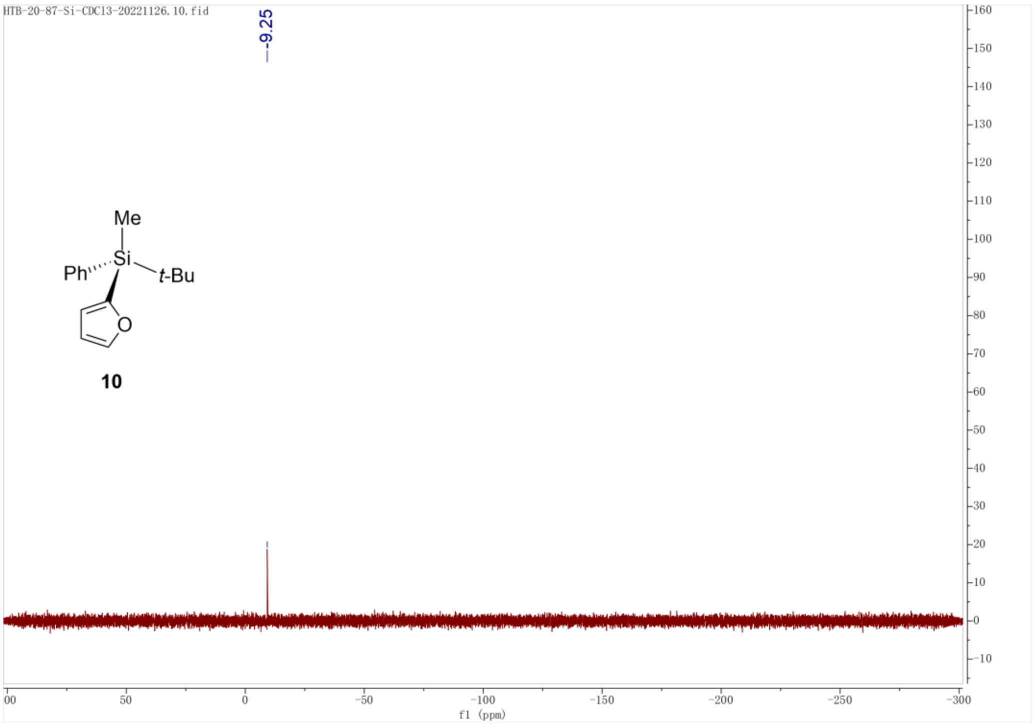

Supplementary Figure 201. ^{29}Si NMR (80 M, $CDCl_3$, 25 °C) of compound **10**

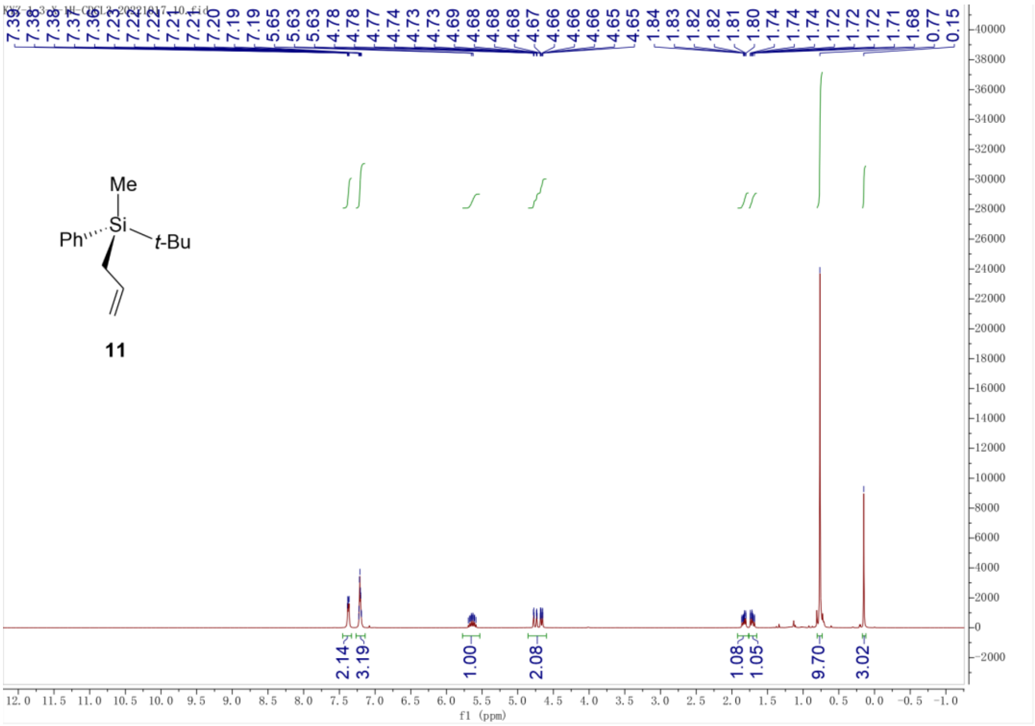

Supplementary Figure 202. ^{1}H NMR (400 M, $CDCl_3$, 25 °C) of compound **11**

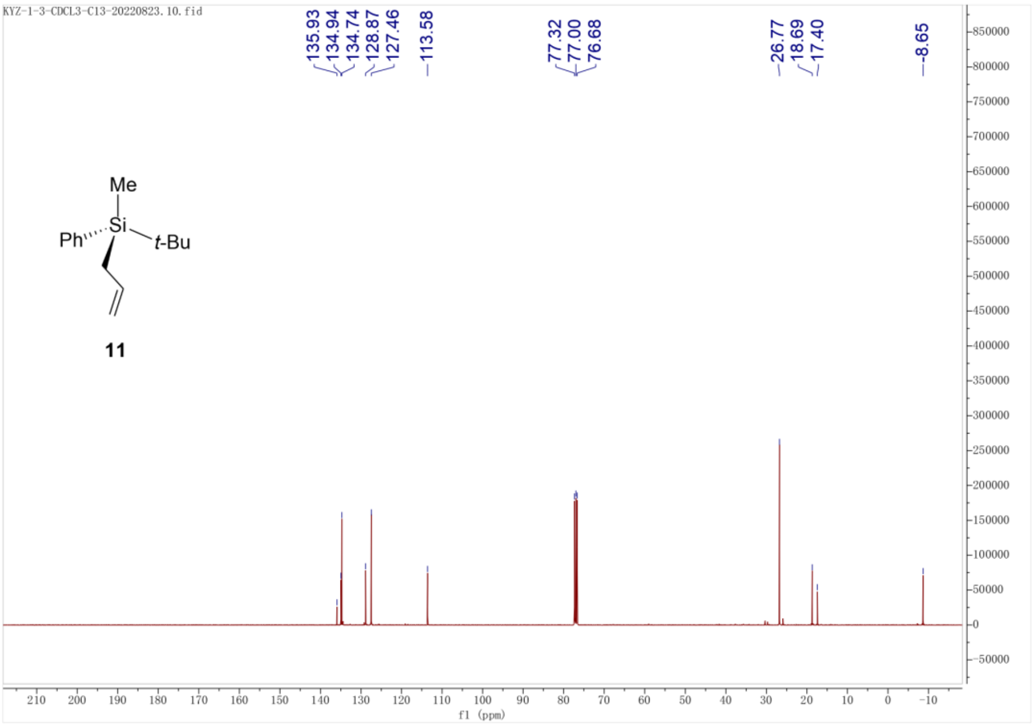

Supplementary Figure 203. ^{13}C NMR (100 M, $CDCl_3$, 25 ^{o}C) of compound **11**

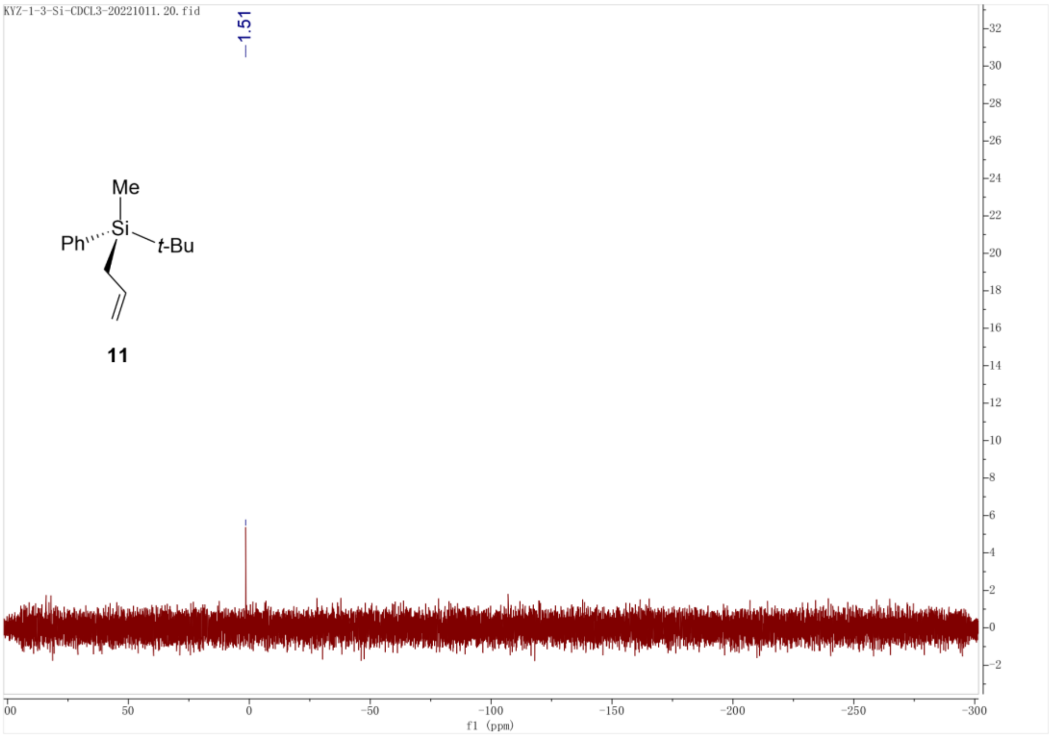

Supplementary Figure 204. ^{29}Si NMR (80 M, $CDCl_3$, 25 ^{o}C) of compound **11**

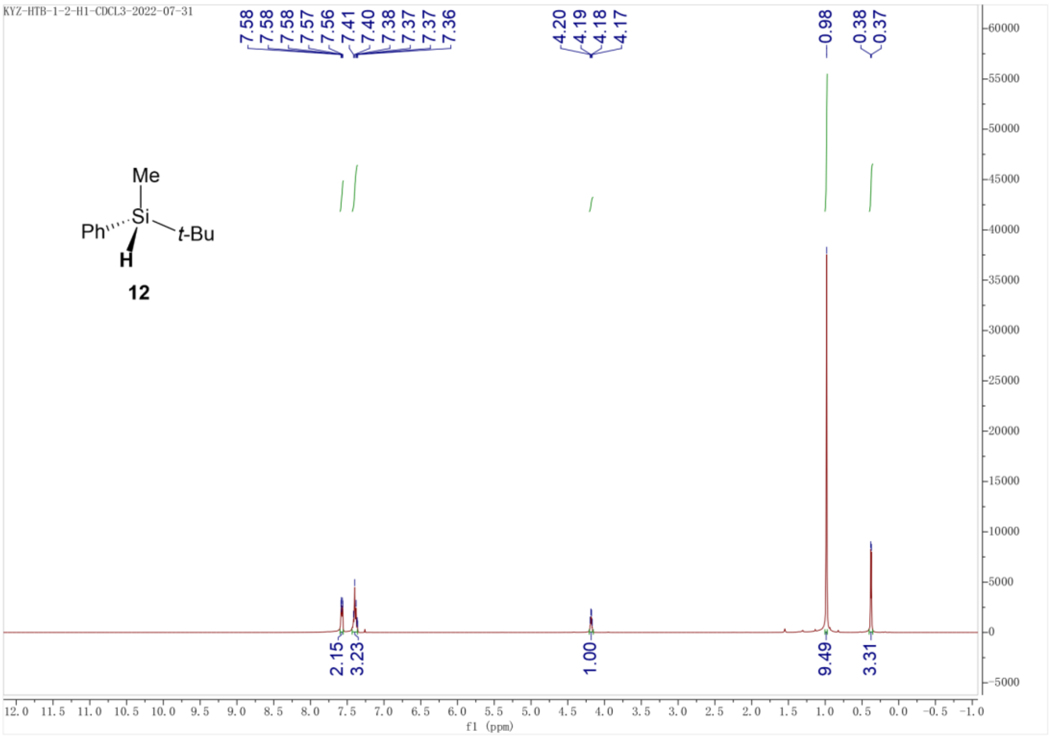

Supplementary Figure 205. ^{1}H NMR (400 M, $CDCl_3$, 25 °C) of compound **12**

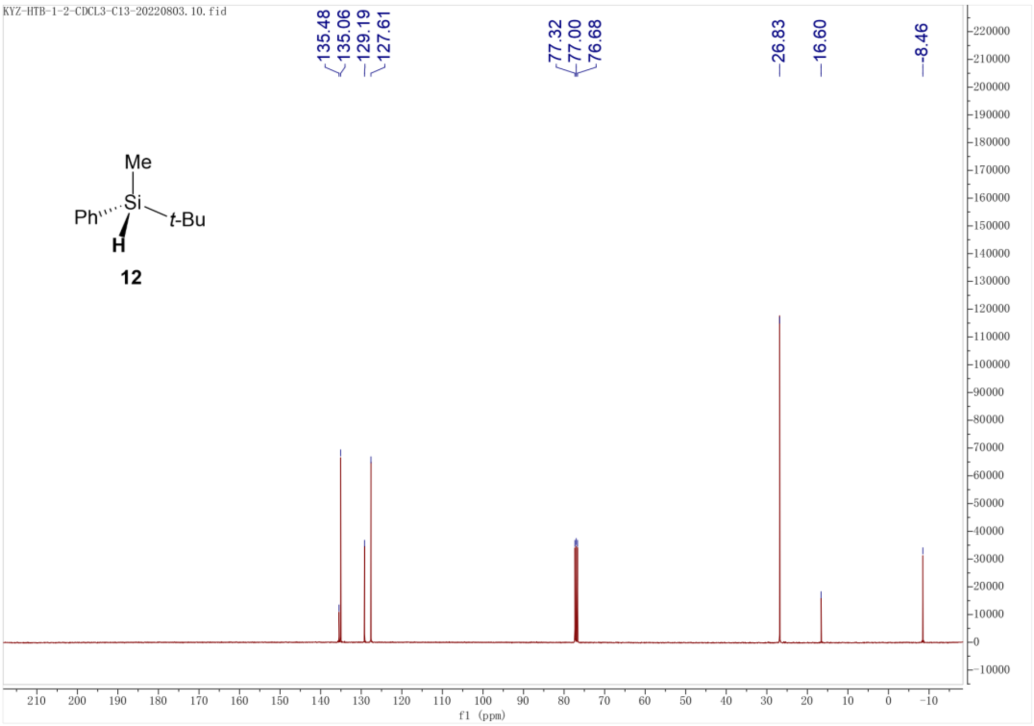

Supplementary Figure 206. ^{13}C NMR (100 M, $CDCl_3$, 25 °C) of compound **12**

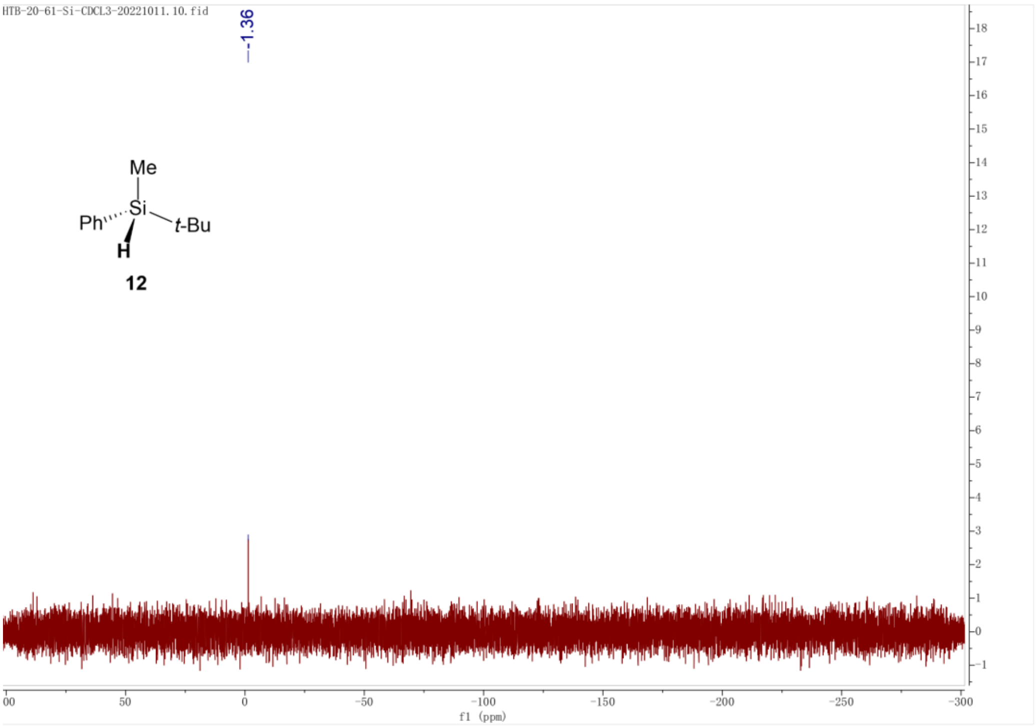

Supplementary Figure 207. ^{29}Si NMR (80 M, $CDCl_3$, 25 ^{o}C) of compound **12**

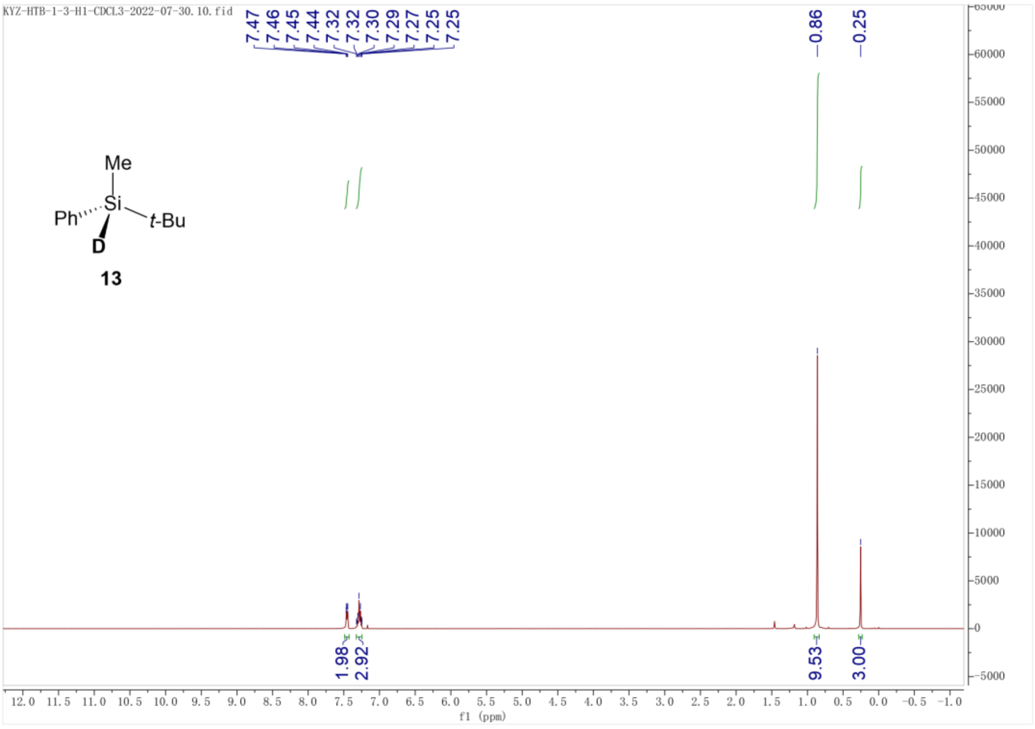

Supplementary Figure 208. ^{1}H NMR (400 M, $CDCl_3$, 25 ^{o}C) of compound **13**

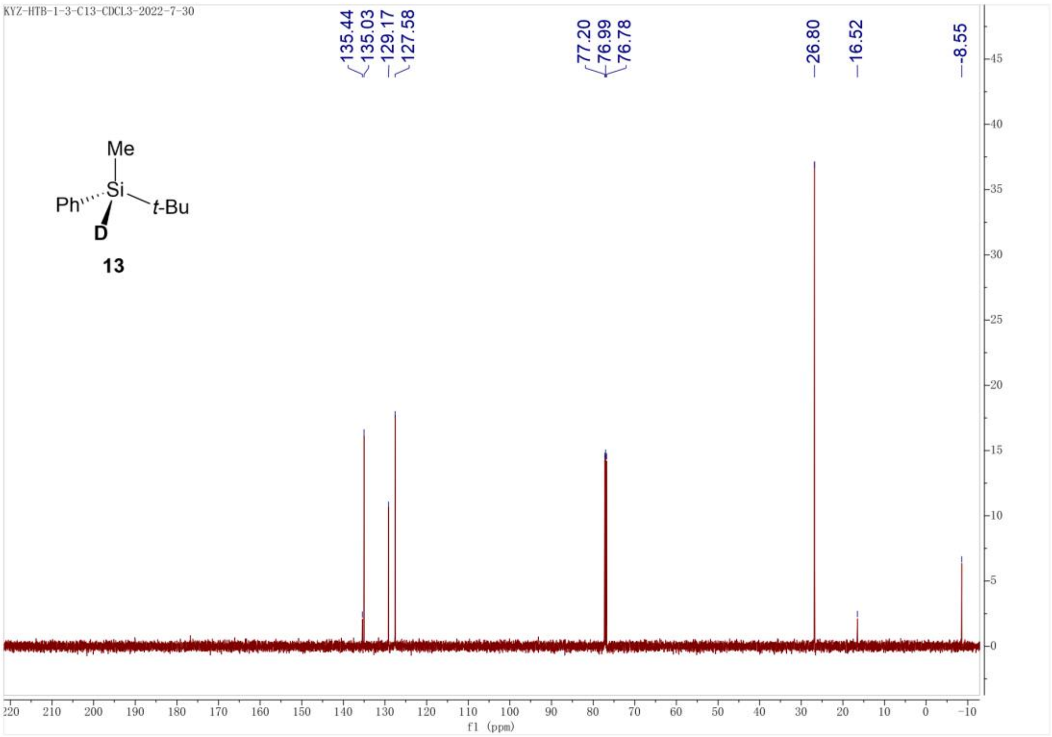

Supplementary Figure 209. ^{13}C NMR (150 M, $CDCl_3$, 25 ^{o}C) of compound **13**

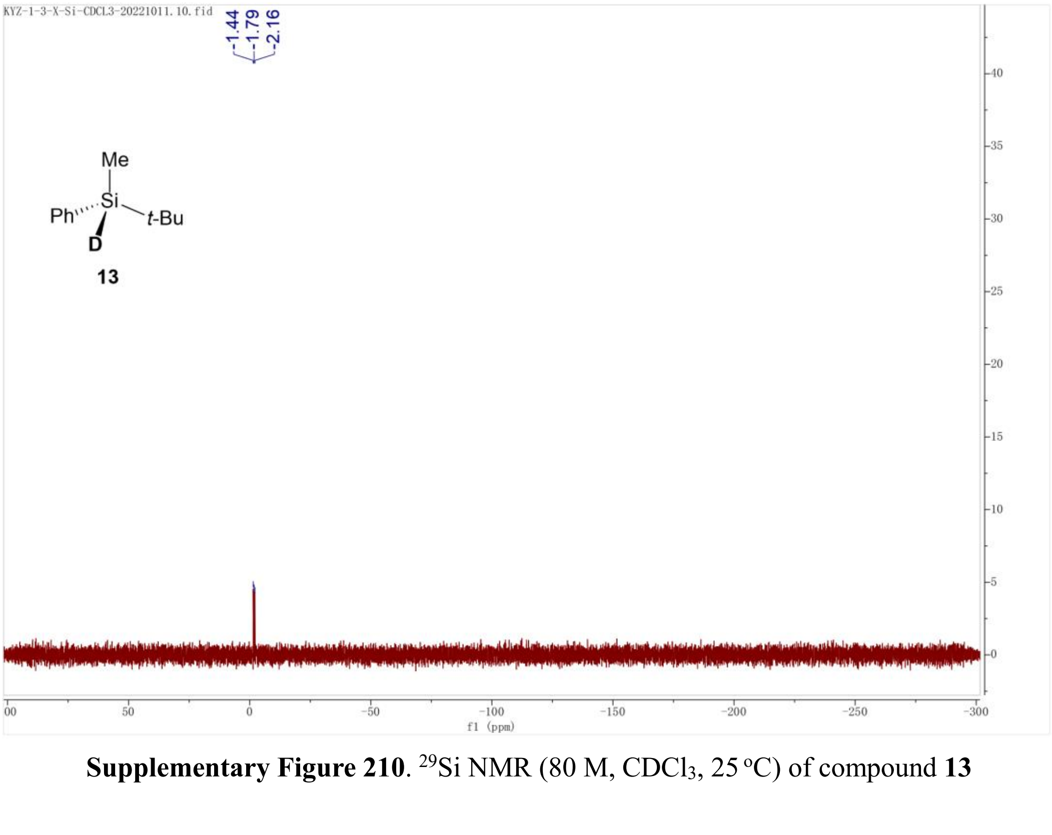

Supplementary Figure 210. ^{29}Si NMR (80 M, $CDCl_3$, 25 ^{o}C) of compound **13**

4. Supplementary Figures 211-217

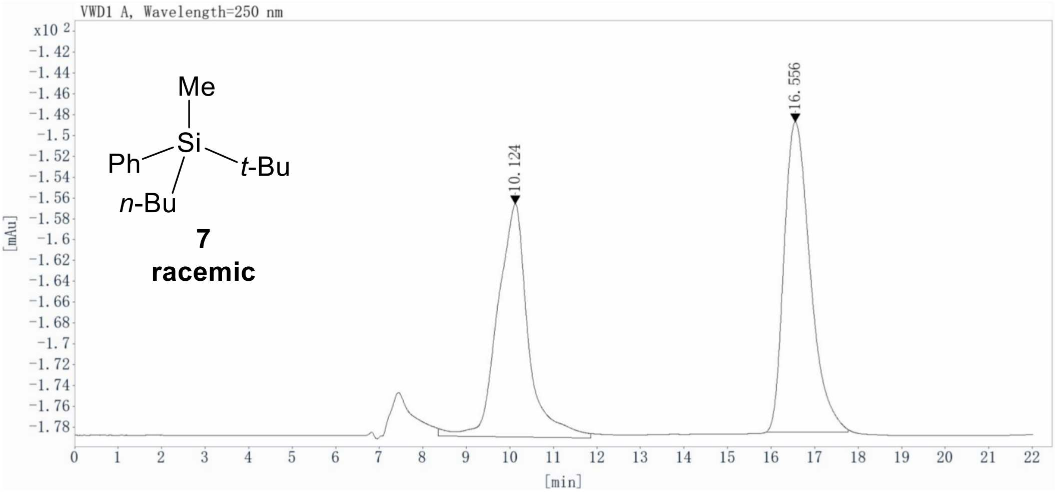

Ret Time [min]	Peak Type	Width [min]	Height [mAU]	Area [mAU*s]	Area [%]
10.124	MM T	1.16	22.4021	1095.8127	46.7777
16.556	PM	0.70	29.7923	1246.7821	53.2223
			Totals:	2342.5948	100.0000

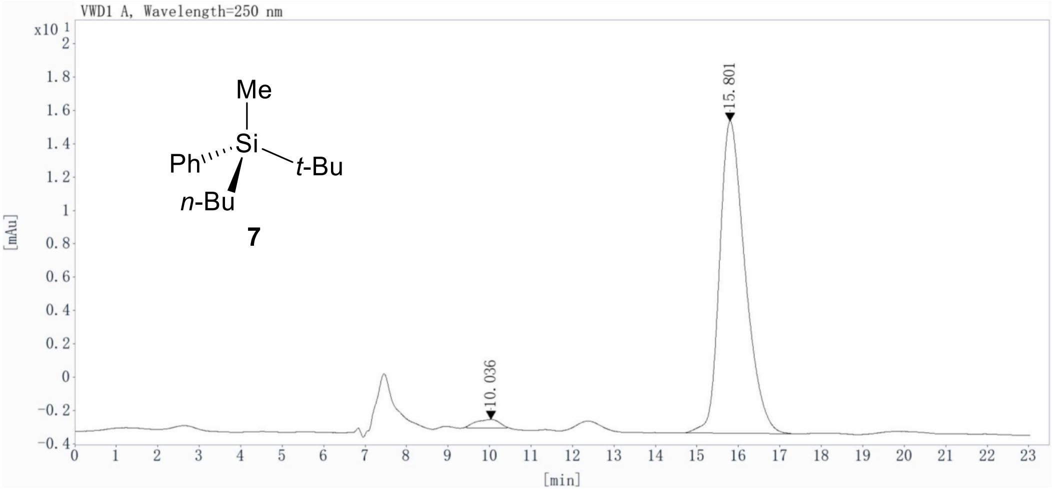

Ret Time [min]	Peak Type	Width [min]	Height [mAU]	Area [mAU*s]	Area [%]
10.036	MM	0.67	0.5257	21.1646	2.5733
15.801	BB	0.65	18.7441	801.3181	97.4267
			Totals:	822.4827	100.0000

Supplementary Figure 211. HPLC trace of **7**

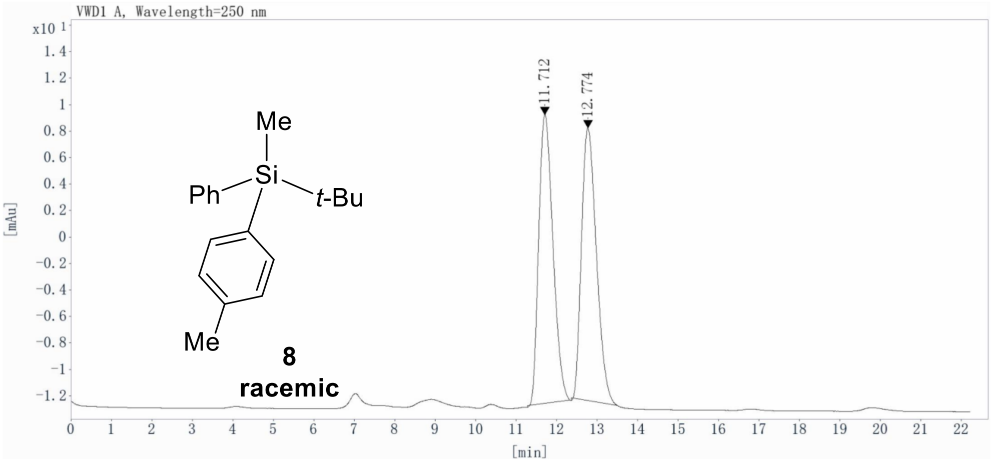

Ret Time [min]	Peak Type	Width [min]	Height [mAU]	Area [mAU*s]	Area [%]
11.712	MM T	0.41	21.8067	530.9917	50.2086
12.774	MM T	0.43	20.5841	526.5788	49.7914
			Totals:	1057.5705	100.0000

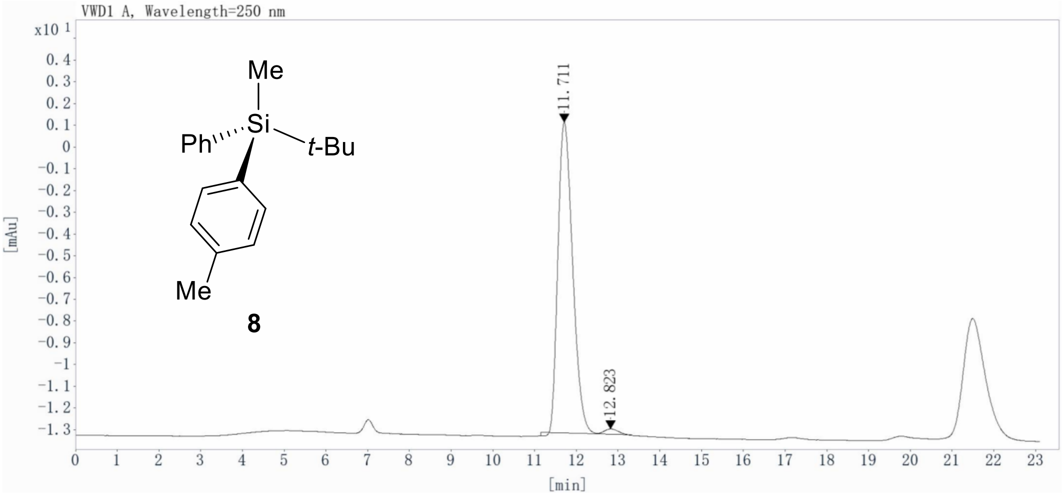

Ret Time [min]	Peak Type	Width [min]	Height [mAU]	Area [mAU*s]	Area [%]
11.711	MF	0.41	14.3001	350.6093	98.4923
12.823	FM	0.37	0.2399	5.3671	1.5077
			Totals:	355.9764	100.0000

Supplementary Figure 212. HPLC trace of **8**

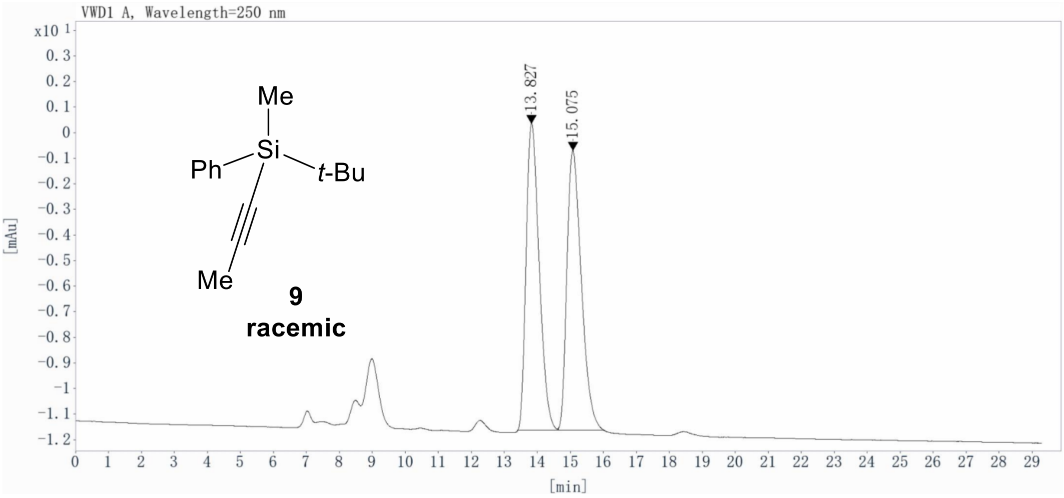

Ret Time [min]	Peak Type	Width [min]	Height [mAU]	Area [mAU*s]	Area [%]
13.827	MM T	0.46	11.9900	329.6114	49.8515
15.075	MM	0.51	10.9298	331.5756	50.1485
			Totals:	661.1870	100.0000

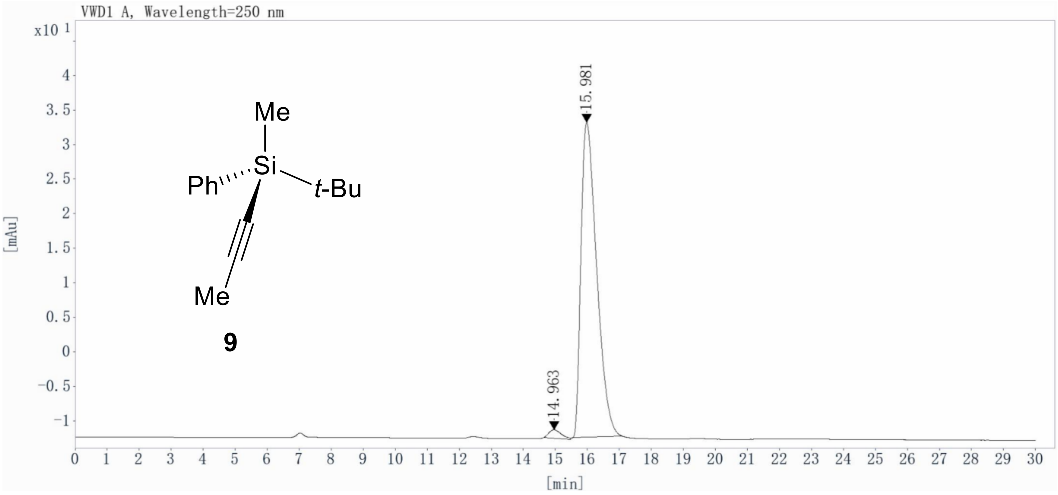

Ret Time [min]	Peak Type	Width [min]	Height [mAU]	Area [mAU*s]	Area [%]
14.963	MM T	0.46	1.2591	35.1024	2.2128
15.981	MM T	0.57	45.6043	1551.2526	97.7872
			Totals:	1586.3549	100.0000

Supplementary Figure 213. HPLC trace of **9**

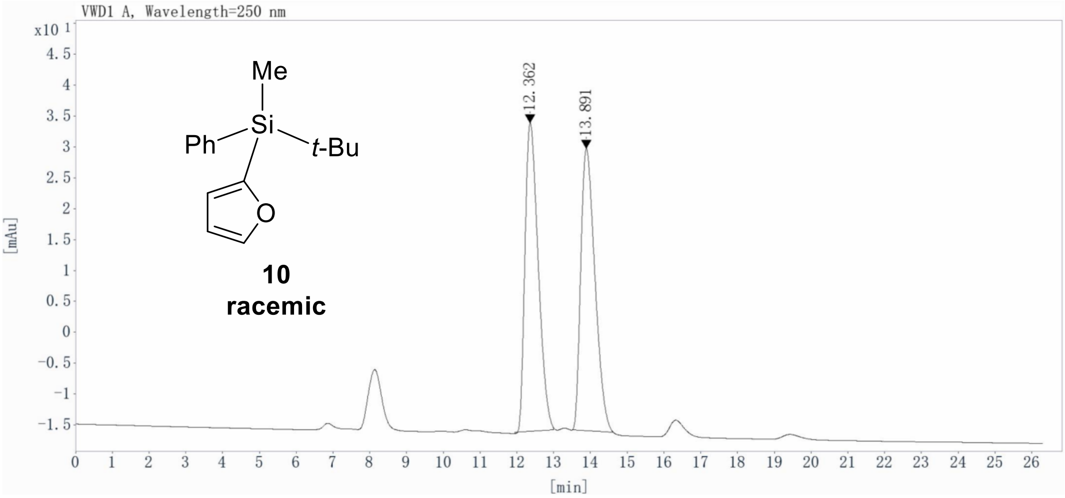

Ret Time [min]	Peak Type	Width [min]	Height [mAU]	Area [mAU*s]	Area [%]
12.362	MM T	0.47	49.9525	1241.6431	50.3553
13.891	MM T	0.45	45.7047	1224.1227	49.6447
			Totals:	2465.7657	100.0000

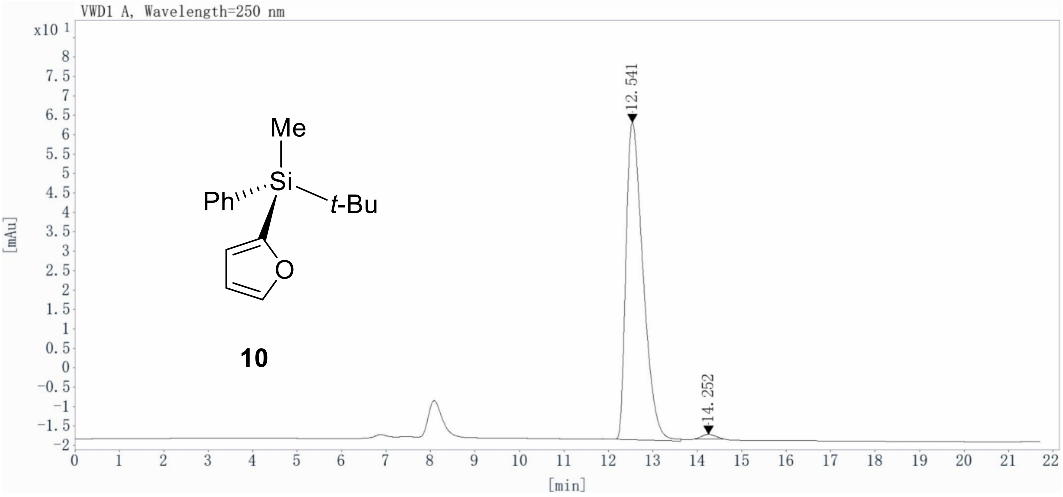

Ret Time [min]	Peak Type	Width [min]	Height [mAU]	Area [mAU*s]	Area [%]
12.541	MM T	0.44	81.7743	2180.3965	98.7486
14.252	PP	0.37	1.2545	27.6317	1.2514
			Totals:	2208.0282	100.0000

Supplementary Figure 214. HPLC trace of **10**

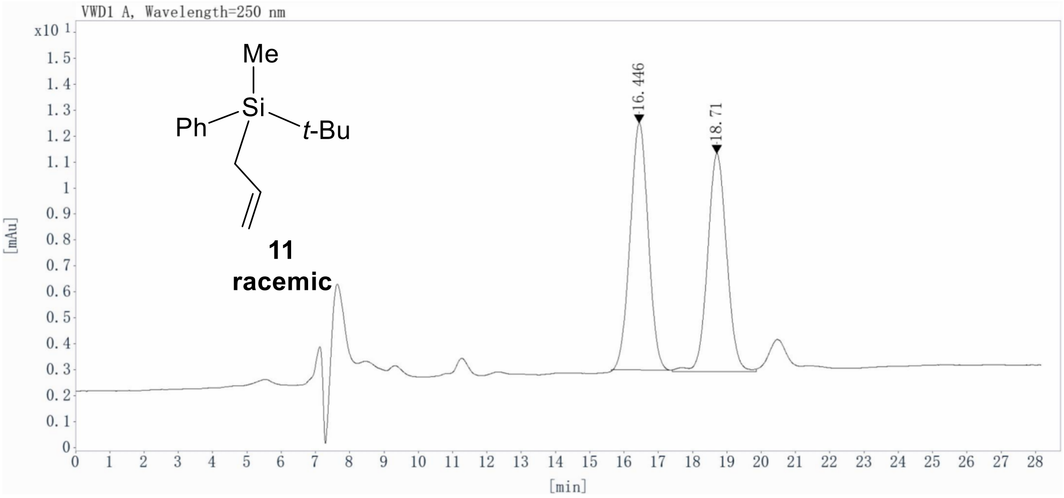

Ret Time [min]	Peak Type	Width [min]	Height [mAU]	Area [mAU*s]	Area [%]
16.446	MM T	0.61	9.5202	348.6889	51.6748
18.710	MM T	0.65	8.4066	326.0869	48.3252
			Totals:	674.7758	100.0000

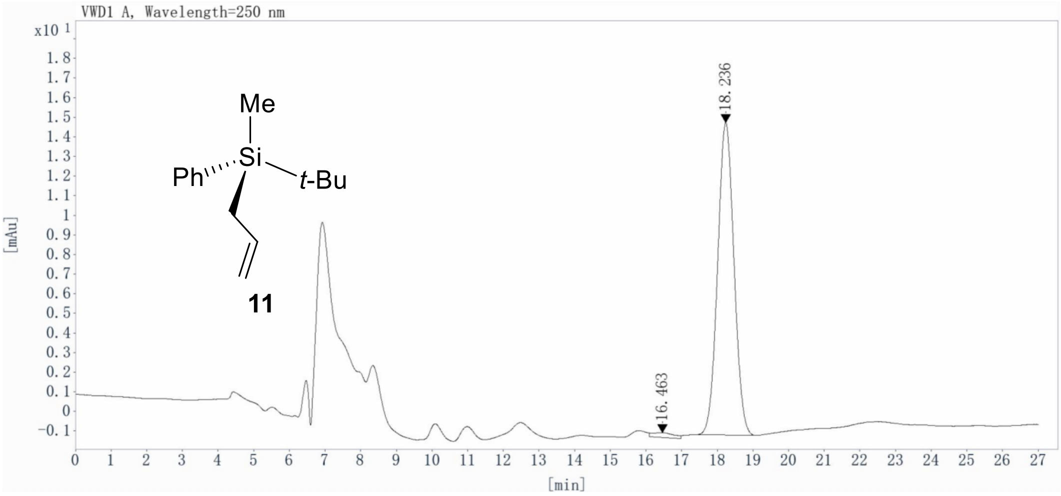

Ret Time [min]	Peak Type	Width [min]	Height [mAU]	Area [mAU*s]	Area [%]
16.463	MM	0.71	0.2490	10.6667	2.0539
18.236	BB	0.50	15.9414	508.6758	97.9461
			Totals:	519.3425	100.0000

Supplementary Figure 215. HPLC trace of **11**

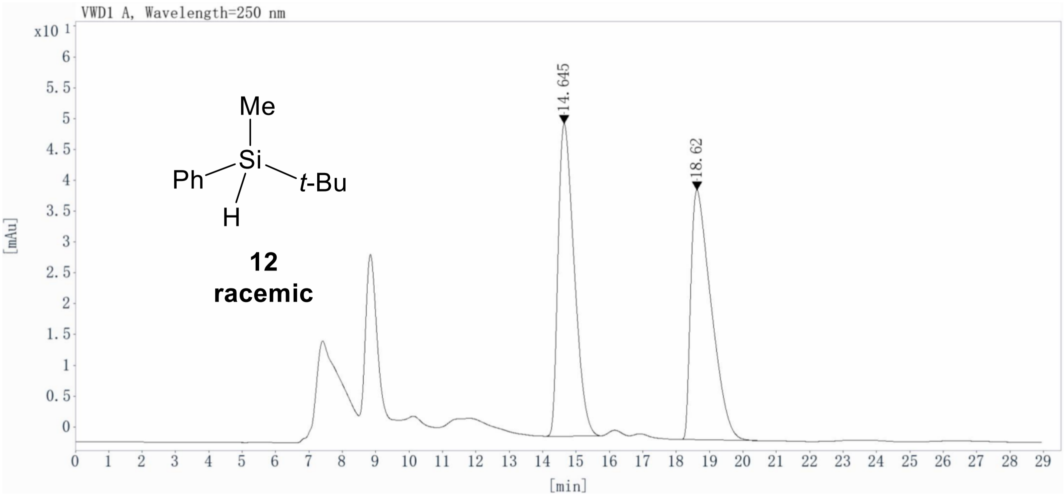

Ret Time [min]	Peak Type	Width [min]	Height [mAU]	Area [mAU*s]	Area [%]
14.645	BB	0.52	50.6970	1692.3638	49.6094
18.620	BB	0.63	40.5035	1719.0121	50.3906
			Totals:	3411.3759	100.0000

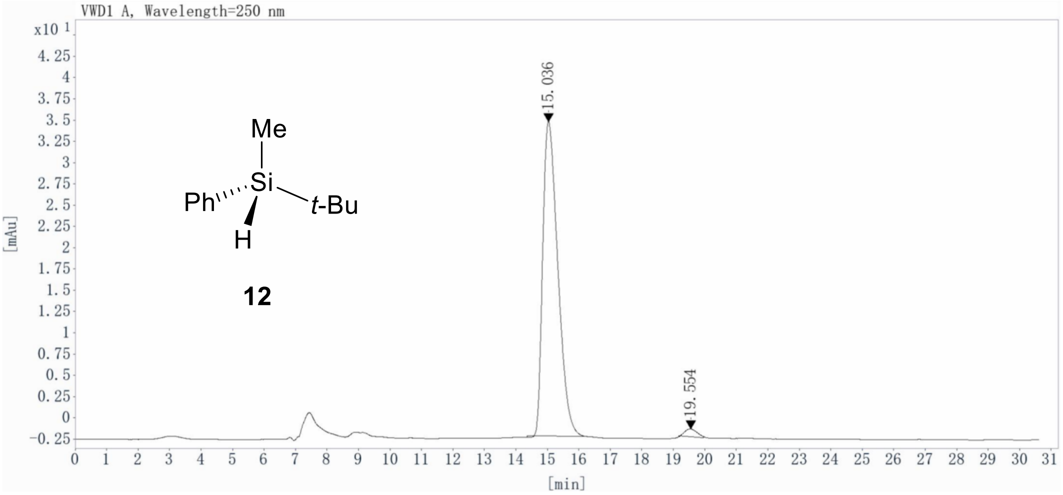

Ret Time [min]	Peak Type	Width [min]	Height [mAU]	Area [mAU*s]	Area [%]
15.036	MM T	0.55	36.9675	1227.9899	98.0358
19.554	MM T	0.45	0.9200	24.6032	1.9642
			Totals:	1252.5931	100.0000

Supplementary Figure 216. HPLC trace of **12**

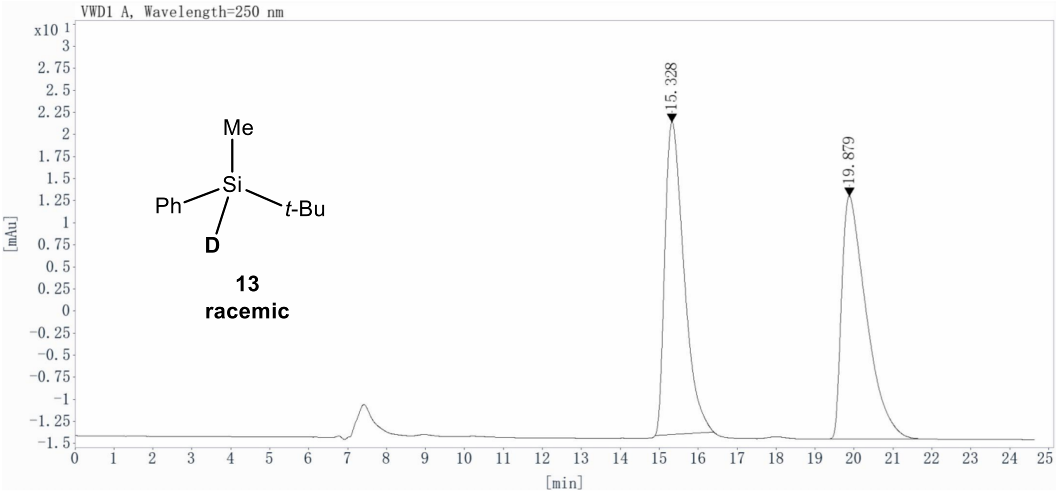

Ret Time [min]	Peak Type	Width [min]	Height [mAU]	Area [mAU*s]	Area [%]
15.328	MM T	0.57	35.4217	1206.0891	49.8971
19.879	BB	0.66	27.5063	1211.0649	50.1029
			Totals:	2417.1541	100.0000

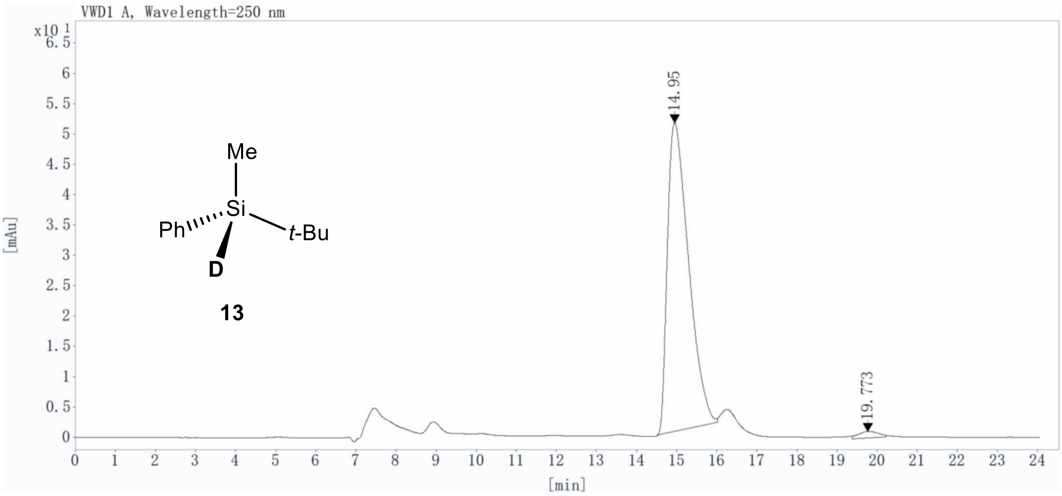

Ret Time [min]	Peak Type	Width [min]	Height [mAU]	Area [mAU*s]	Area [%]
14.950	MM T	0.61	50.8703	1866.1689	98.0025
19.773	MM T	0.58	1.1008	38.0367	1.9975
			Totals:	1904.2057	100.0000

Supplementary Figure 217. HPLC trace of **13**

5. Supplementary References

1. Trepohl, V. T.; Fröhlich, R.; Oestreich, M. Conjugate phosphination of cyclic and acyclic acceptors using Rh(I)-phosphineor Rh(I)-carbene complexes. Probing the mechanism with chirality at the silicon atom or the phosphorus atom of the Si-P reagent. *Tetrahedron*. **2009**, *65*, 6510-6518.
2. Zhang, G.; Li, Y, -F.;Wang, Y.; Zhang, Q.; Xiong, T.; Zhang, Q. Asymmetric synthesis of silicon-stereogenic silanes by copper-catalyzed desymmetrizing protoboration of vinylsilanes. *Angew. Chem. Int. Ed.* **2020**, *59*, 11927-11931.
3. Zhou, M. -L.; Liu, J. -J.; Deng, R.; Wang, Q. -Y.; Wu, S.-Q.; Zheng, P. -C.; Chi, Y. R. Construction of tetrasubstituted silicon-stereogenic silanes via conformational isomerization and N-heterocyclic carbene-catalyzed desymmetrization. *ACS Catal.* **2022**, *12*, 7781-7788.
4. DiBlasi, C. M.; Macks, D. E.; Tan, D. S. An Acid-Stable *tert*-butyldiarylsilyl (TBDAS) linker for solid-phase organic synthesis. *Org. Lett.* **2005**, *7*, 1777-1780.
5. Zhao, Z. -Z.; Pang, X. -B.; Wei, X. -X.; Liu, X.-Y.; Shu, X. -Z. Nickel-catalyzed reductive C(sp^2)-Si Coupling of Chlorohydrosilanes via Si-Cl Cleavage. *Angew. Chem. Int. Ed.* **2022**, *61*, e202200215.
6. Fernandes, A.; Laye, C.; Pramanik, S.; Palmeira, D.; Pekel, Ö. Ö.; Massip, S.; Schmidtmann, M.; Müller, T.; Robert, F.; Landais, Y. Chiral memory in silyl-pyridinium and quinolinium cations. *J. Am. Chem. Soc.* **2020**, *142*, 564-572 (2020).
7. Rohde, Volker H. G.; Pommerening, P.; Klare, Hendrik F. T.; Oestreich, M. *Organometallics.* **2014**, *33*, 3618-3628.
8. Fang, H. -Q.; Hou, W. -J.; Liu, G. -X.; Huang, Z. *J. Am. Chem. Soc*. **2017**, ***139***, 11601-11609.
9. Wang, X.; Long, C, -Y.; Su, M, -H.; Qu, Y, -X.; Li, S, -H.; Zhang, X. -J.; Huang, S, -J.; Wang, X. -Q. Rapid amination of methoxy pyridines with aliphatic amines. *Org. Process Res. Dev*. **2019**, *23*, 1587-1593.
10. Jeong, Y.; Lee, J.; Ryu, J. S. Design, synthesis, and evaluation of hinge-binder tethered 1,2,3-triazolylsalicylamide derivatives as Aurora kinase inhibitors. *Bioorganic & Medicinal Chemistry*. **2016**, *24*, 2114-2124.
11. Song, Z. -D.; Huang, Y. -Y.; Hou, K. -Q.; Liu, L.; Zhou, F.; Huang, Y.; Wan, G. -H.; Luo, H. -B.; Xiong, X. -F. Discovery and structural optimization of Toddacoumalone derivatives as novel PDE4 inhibitors for the topical treatment of psoriasis. *J. Med. Chem.* **2022**, *65*, 4238-4254.
12. Mayer, S. C.; Ramanjulu, J.; Vera, M. D.; Pfizenmayer, A. J.; Joullie, M. M. Synthesis of new Didemnin B analogs for investigations of structure/biological activity relationships. *J. Org. Chem.* **1994**, *59*, 5192-5205.
13. Qin, Y.; Li, L. -H.; Liang, J. -Y.; Li, K. -L.; Zhao, D. -B. *Chem. Sci.* **2021**, *12*, 14224-4229.

14. Igawa, K.; Kokan, N.; Tomooka, K. Asymmetric synthesis of chiral silacarboxylic acids and their ester derivatives. *Angew. Chem. Int. Ed.* **2010**, *49*, 728-731.

15. Jankowski, P.; Schaumann, E.; Wicha, J.; Zarecki, A.; Adiwidjaja, G. *Tetrahedron: Asymmetry*. **1999**, *10*, 519-526.

16. Wu, Y.; Chen, H.; Yang, W. -Y.; Fan, Y.; Gao, L.; Su, Z. -S & Hu, C. -W & Song, Z. -L. Asymmetric retro- [1, 4]-Brook rearrangement of 3-silyl allyloxysilanes via chirality transfer from silicon to carbon. *RSC Adv*. **2019**, *9*, 26209-26213.

17. Sommer, L. H.; Frye, C. L.; Parker. G. A. stereochemistry of asymmetric silicon. II. alkoxy and siloxy leaving groups. *J. Am. Chem. Soc*. **1964**, *86*, 3276-3279.

18. Frisch, M. J.; Trucks, G. W.; Schlegel, H. B.; Scuseria, G. E.; Robb, M. A.; Cheeseman, J. R.; Scalmani, G.; Barone, V.; Mennucci, B.; Petersson, G. A.; Nakatsuji, H.; Caricato, M.; Li, X.; Hratchian, H. P.; Izmaylov, A. F.; Bloino, J.; Zheng, G.; Sonnenberg, J. L.; Hada, M.; Ehara, M.; Toyota, K.; Fukuda, R.; Hasegawa, J.; Ishida, M.; Nakajima, T.; H *J. Am. Chem. Soc*. **1964**, *86*, 3276-3279.onda, Y.; Kitao, O.; Nakai, H.; Vreven, T.; Montgomery, J. A.; Peralta, J. J. E.; Ogliaro, F.; Bearpark, M.; Heyd, J. J.; Brothers, E.; Kudin, K. N.; taroverov, V. N.; Keith, T.; Kobayashi, R.; Normand, J.; Raghavachari, K.; Rendell, A.; Burant, J. C.; Iyengar, S. S.; Tomasi, J.; Cossi, M.; Rega, N.; Millam, J. M.; Klene, M.; Knox, J. E.; Cross, J. B.; Bakken, V.; Adamo, C.; Jaramillo, J.; Gomperts, R.; Stratmann, R. E.; Yazyev, O.; Austin, A. J.; Cammi, R.; Pomelli, C.; Ochterski, J. W.; Martin, R. L.; Morokuma, K.; Zakrzewski, V. G.; Voth, G. A.; Salvador, P.; Dannenberg, J. J.; Dapprich, S.; Daniels, A. D.; Farkas, O.; Foresman, J. B.; Ortiz, J. V.; Cioslowski, J.; Fox, D. J. Gaussian 09 (Revision D.01) I. Gaussian, Wallingford, CT, **2013**.

19. Zhao, Y. and Truhlar, D. G. “The M06 suite of density functionals for main group thermochemistry, thermochemical kinetics, noncovalent interactions, excited states, and transition elements: two new functionals and systematic testing of four M06-class functionals and 12 other functionals,” *Theor. Chem. Acc*. **2008**, *120*, 215-241.

20. Grimme, S.; Antony, J.; Ehrlich, S.; Krieg, H. A Consistent and accurate *ab initio* parametrization of density functional dispersion correction (DFT-D) for the 94 elements H-Pu. *J. Chem. Phys*. **2010**, *132*, 154104.

21. Marenich, A. V.; Cramer, C. J.; Truhlar, D. Universal solvation model based on solute electron density and on a continuum model of the solvent defined by the bulk dielectric constant and atomic surface tensions. *J. Phys. Chem. B*. **2009**, *113*, 6378-6396.

22. Gonzalez, C.; Schlegel, H. B. An improved algorithm for reaction path following. *J. Chem. Phys*. **1989**, *90*, 2154-2161.

23. CYLview, 1.0b; Legault, C. Y., Université de Sherbrooke, (2009) (http://www.cylview.org).

24. Lu, T.; Chen, F. -W. Multiwfn: a multifunctional wavefunction analyzer, *J. Comput. Chem*. **2012**, *33*, 580-592.
