# Supplementary material for: Enantioconvergent construction of stereogenic silicon via Lewis base-catalyzed dynamic kinetic silyletherification of racemic chlorosilanes
Source: Nat Commun. 2023 Aug 14;14:4900. doi: 10.1038/s41467-023-40558-6 (PMC10425371; doi:10.1038/s41467-023-40558-6)
Supplement: Supplementary file 3 — Description of Additional Supplementary Files [file 41467_2023_40558_MOESM3_ESM.pdf]

Description of Additional Supplementary Files

File Name: Supplementary Data 1
Description: cartesian coordinates of the optimized structures
