# Supplementary material for: Enantioconvergent construction of stereogenic silicon via Lewis base-catalyzed dynamic kinetic silyletherification of racemic chlorosilanes
Source: Nat Commun. 2023 Aug 14;14:4900. doi: 10.1038/s41467-023-40558-6 (PMC10425371; doi:10.1038/s41467-023-40558-6)
Supplement: Supplementary file 4 — Supplementary Data 1 [file 41467_2023_40558_MOESM4_ESM.pdf]

Cartesian coordinates of all stationary points

Cat (3i)

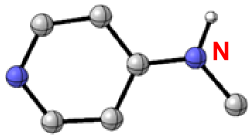

C	0.43932200	-0.28731300	-0.03589000
C	-0.00598700	1.04696500	-0.03252100
C	-0.55745100	-1.28379900	-0.00250200
C	-1.36972800	1.29730800	-0.00584200
H	0.69166200	1.87362000	-0.05065400
C	-1.88518100	-0.90941000	0.02617100
H	-0.28108700	-2.33245200	0.00108500
N	-2.31970800	0.35974400	0.02411300
H	-1.71499300	2.32782700	-0.00520100
H	-2.65338500	-1.67742900	0.05486800
N	1.75567700	-0.61625600	-0.08175500
H	1.98988900	-1.58484600	0.07414200
C	2.81417200	0.36175300	0.05996000
H	2.74732800	0.90595000	1.00902900
H	3.77007100	-0.15736900	0.02161800
H	2.78784800	1.08725600	-0.75764800

2g

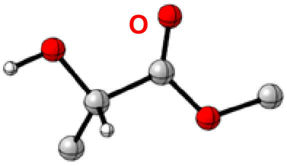

C	0.29015900	-0.33497300	-0.02491300
O	0.54288900	-1.37161400	0.52331600
O	1.21267500	0.57001700	-0.37032300
C	2.56371100	0.24278500	-0.02334500
H	3.16570800	1.08901300	-0.34481200
H	2.65643400	0.09953200	1.05380900

H	2.87882400	-0.66457100	-0.54003700
C	-1.09510800	0.15690600	-0.39992900
H	-1.05306200	0.46825100	-1.45097500
C	-1.47172400	1.35119400	0.47294200
H	-2.47621400	1.69217400	0.21190200
H	-1.46431200	1.05613500	1.52496200
H	-0.77713800	2.17907400	0.32642100
O	-1.97848600	-0.92586200	-0.22151100
H	-2.86908900	-0.59539700	-0.38165800

NEt_3

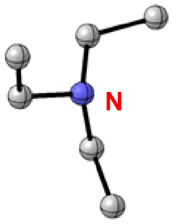

N	0.00042800	-0.00107300	-0.00798300
C	1.08341900	-0.88153900	0.44049100
C	2.32802000	-0.76526700	-0.42959200
H	0.72592900	-1.91272700	0.39575400
H	1.33244000	-0.68024300	1.49685100
H	3.10469500	-1.44955500	-0.07866200
H	2.08479200	-1.01751400	-1.46430700
H	2.74540700	0.24398800	-0.41648500
C	0.22068000	1.37634000	0.44349900
C	-0.49868100	2.39573500	-0.42982700
H	1.29252300	1.58234200	0.40596300
H	-0.08504000	1.49106700	1.49783000
H	-0.30233500	3.40995000	-0.07338700
H	-0.14906100	2.31687800	-1.46166400
H	-1.58066400	2.24616600	-0.42656300
C	-1.30117200	-0.49948700	0.44652100
C	-1.83237000	-1.62511000	-0.43190700
H	-2.01340400	0.32807500	0.41873500
H	-1.24223500	-0.82830100	1.49857000

H	-2.80928900	-1.96017300	-0.07479600
H	-1.93992300	-1.27544200	-1.46122000
H	-1.16621400	-2.49103300	-0.43585300

NEt$_3$·HCl

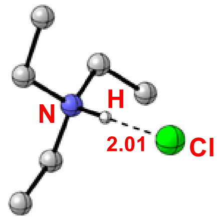

N	0.53375900	0.00785700	-0.01017600
C	0.96275300	1.28785000	0.64052100
C	0.61057300	1.36053000	2.11566200
H	0.45129800	2.08632500	0.10576900
H	2.03848900	1.38397300	0.47173700
H	0.76580700	2.38731900	2.45063500
H	-0.44055400	1.10955500	2.27798400
H	1.23296200	0.71061000	2.73064300
C	1.01513500	-1.19275300	0.74639400
C	0.63746500	-2.51004400	0.09201500
H	0.55844900	-1.13276000	1.73297200
H	2.09757900	-1.08591100	0.85354000
H	0.85115700	-3.31297900	0.79932100
H	-0.43027100	-2.53916400	-0.13834600
H	1.20508800	-2.70619900	-0.81747100
C	0.92569100	-0.04577600	-1.45621600
C	0.49421600	1.17884200	-2.24372500
H	0.44319000	-0.93063500	-1.86784600
H	2.00870800	-0.18979100	-1.48580700
H	0.63908000	0.96710400	-3.30416400
H	-0.56556500	1.39135400	-2.08385900
H	1.07932000	2.06486500	-1.99730200
H	-0.52118400	-0.01170200	0.01921500
Cl	-2.53087400	-0.04228600	0.05625300

Si^{S}-IM1

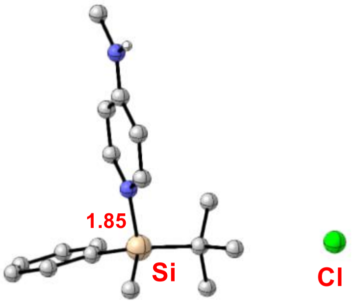

Si	-0.00008300	-0.30981100	-0.60902900
C	-0.00012700	-0.62557600	-2.47856300
H	0.88266500	-1.19343600	-2.77518800
H	-0.88278500	-1.19375400	-2.77500200
H	-0.00038700	0.30089300	-3.06151900
C	-4.91263100	-0.02416400	-0.70914600
C	-4.26071400	-0.79241400	0.27887800
C	-4.08614600	0.56038600	-1.69797700
C	-2.89017500	-0.91698400	0.24423200
H	-4.81355400	-1.28304000	1.06804300
C	-2.73121600	0.36928800	-1.64749100
H	-4.51966600	1.15593900	-2.49211400
N	-2.10627800	-0.35251100	-0.68983000
H	-2.38840700	-1.49627000	1.00864400
H	-2.10064400	0.81450800	-2.40640500
N	-6.24259900	0.14557800	-0.72921600
H	-6.63159000	0.72961000	-1.45442500
C	-7.14057700	-0.40676800	0.26606100
H	-8.15728400	-0.11847800	0.00792300
H	-7.08238800	-1.49864100	0.28530900
H	-6.91017700	-0.02371900	1.26449400
C	0.00003500	1.44285400	0.22985800
C	-0.00002500	2.53050800	-0.86065200
C	-1.23401200	1.68705900	1.12488200
C	1.23428600	1.68695900	1.12463000
H	0.88641900	2.47904400	-1.49968300
H	-0.88715100	2.47983900	-1.49880800

H	0.00065100	3.51372800	-0.37476500
H	-1.36293800	0.91110400	1.88630000
H	-1.09924600	2.64317700	1.64117800
H	-2.16217300	1.75967100	0.55565300
H	1.09948700	2.64287900	1.64126700
H	1.36354800	0.91078100	1.88576400
H	2.16228200	1.75990800	0.55516900
C	-0.00006800	-1.85530900	0.47017300
C	-0.00008900	-3.11379900	-0.14408900
C	-0.00001200	-1.80759600	1.87045800
C	-0.00006400	-4.28407900	0.61104500
H	-0.00012700	-3.19072600	-1.22786600
C	0.00000600	-2.97512500	2.63061500
H	-0.00000200	-0.85055600	2.38289400
C	-0.00001400	-4.21632800	2.00104900
H	-0.00008300	-5.24779600	0.11404100
H	0.00004000	-2.91435300	3.71317300
H	0.00000600	-5.12612200	2.59070000
C	4.91254700	-0.02439900	-0.70924700
C	4.26062200	-0.79272100	0.27871300
C	4.08607300	0.56019400	-1.69806200
C	2.89007800	-0.91726800	0.24405500
H	4.81345300	-1.28342000	1.06783900
C	2.73113900	0.36911800	-1.64758500
H	4.51960800	1.15575700	-2.49218400
N	2.10618300	-0.35269700	-0.68994600
H	2.38830400	-1.49660000	1.00842900
H	2.10059500	0.81432500	-2.40653100
N	6.24251900	0.14533500	-0.72930800
H	6.63148500	0.72958100	-1.45435800
C	7.14046100	-0.40682900	0.26610300
H	8.15716400	-0.11846400	0.00803100
H	6.90993100	-0.02372300	1.26448700
H	7.08238300	-1.49870500	0.28543100

Cl	0.00039400	5.57131900	1.75240800

Si^{S}-TS1

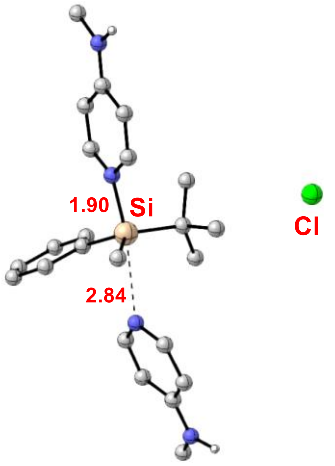

Si	0.44433200	-0.31585700	-0.64063700
C	0.17862500	-0.55900100	-2.47977600
H	0.98372300	-1.16646000	-2.90189200
H	-0.76651400	-1.06698200	-2.66120700
H	0.16156600	0.39159700	-3.01996900
C	-5.12148200	0.09038800	-0.65260100
C	-4.48670100	-0.77865400	0.25695600
C	-4.32214800	0.60873900	-1.69468100
C	-3.14062500	-1.05644800	0.09016100
H	-5.02779100	-1.22414000	1.08117900
C	-2.99209700	0.25634300	-1.76011200
H	-4.75093600	1.28000200	-2.42999700
N	-2.37838000	-0.55719400	-0.88610300
H	-2.64064200	-1.71648900	0.79548100
H	-2.37109700	0.65780600	-2.55711500
N	-6.43151200	0.40937700	-0.55605900
H	-6.78638500	1.11342900	-1.18480500
C	-7.27511500	-0.01264600	0.54220500
H	-8.26963200	0.40334800	0.39197700
H	-7.35964500	-1.10265200	0.57725400
H	-6.89237900	0.33859800	1.50641200
C	0.17205100	1.41045400	0.15256100

C	0.15380500	2.47241300	-0.96097000
C	-1.13160600	1.55234500	0.96046500
C	1.34056700	1.74181800	1.10635000
H	1.07886000	2.47947300	-1.54711200
H	-0.68518200	2.32199900	-1.64718900
H	0.04601500	3.46215300	-0.50236600
H	-1.26485300	0.75618600	1.69774500
H	-1.09438500	2.50666400	1.49715500
H	-2.01367500	1.56997000	0.32094300
H	1.14359400	2.71610700	1.56581300
H	1.44881300	1.00746200	1.91160000
H	2.29860000	1.81579600	0.58523300
C	0.04277500	-1.81986900	0.40424600
C	-0.24274700	-3.03546900	-0.23131000
C	-0.02365700	-1.77485100	1.80288100
C	-0.58587500	-4.16641700	0.50294000
H	-0.21268400	-3.10155600	-1.31485800
C	-0.36793500	-2.90460300	2.54236900
H	0.19119900	-0.85224900	2.33209600
C	-0.65211200	-4.10126900	1.89251600
H	-0.80629800	-5.09630000	-0.00905800
H	-0.41713800	-2.84697200	3.62386300
H	-0.92348700	-4.98048900	2.46597900
C	5.14733800	-0.13154000	-0.55609300
C	4.43280400	-0.84877100	0.43387700
C	4.38301800	0.43468000	-1.60870100
C	3.06923700	-0.94186900	0.34741100
H	4.93962500	-1.32456300	1.26173200
C	3.02757600	0.28909500	-1.61649600
H	4.87044900	0.98329200	-2.40492400
N	2.34766100	-0.38521800	-0.65122900
H	2.51073000	-1.48215300	1.10107800
H	2.43626500	0.72166300	-2.41355500
N	6.47260600	0.01039900	-0.51948700

H	6.90842800	0.54672200	-1.25634400
C	7.31784000	-0.52351000	0.53306200
H	8.34863600	-0.25799600	0.30998300
H	7.04708200	-0.10181800	1.50465400
H	7.23887400	-1.61249500	0.58280200
Cl	0.04681100	5.51372600	1.64428900

Si^{S}/C^{S}-TS2

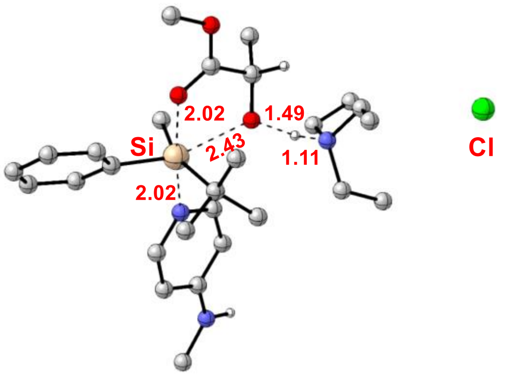

C	0.84803700	4.31075900	-0.30332400
C	-0.07449900	3.34217100	-0.76858200
C	2.07380500	3.82327300	0.20203900
C	0.25026100	2.01423900	-0.70829700
H	-1.03356500	3.65053900	-1.16799800
C	2.30715100	2.46715100	0.21913900
H	2.83894500	4.49308600	0.56907000
N	1.42482300	1.55326300	-0.22159800
H	-0.44697900	1.25638400	-1.04963300
H	3.24989500	2.08735100	0.59009200
N	0.55998300	5.61845000	-0.35059100
H	-0.33377600	5.89095600	-0.73237700
C	1.45461000	6.65952600	0.11708900
H	0.96482500	7.62043200	-0.02443100
H	2.39195000	6.65825600	-0.44625500
H	1.68103500	6.53516800	1.17972300
C	0.48197400	-3.09603700	-0.39318700
O	1.52279100	-2.47953900	-0.17089800
O	0.46910300	-4.39999200	-0.44966200

C	1.72295000	-5.08209800	-0.23388800
H	2.46410400	-4.73322600	-0.95184100
H	1.50583500	-6.13563000	-0.38256800
H	2.06827900	-4.89986500	0.78351900
C	-0.85401600	-2.41405300	-0.61981000
H	-1.54431500	-2.94254900	0.06520400
C	-1.30610800	-2.73711300	-2.05721600
H	-1.36230300	-3.81577900	-2.21710700
H	-0.61713000	-2.30425100	-2.78486500
H	-2.29891800	-2.31791300	-2.22707500
O	-0.73801200	-1.07639000	-0.32051100
H	-2.03091600	-0.34314600	-0.19950100
N	-3.05649900	0.07700900	-0.24565500
C	-3.21149100	0.47438900	-1.68660500
C	-4.60187800	0.30587400	-2.27980300
H	-2.49324700	-0.13113300	-2.24310700
H	-2.88572200	1.51472300	-1.75458500
H	-4.58326700	0.70796900	-3.29496500
H	-4.89049700	-0.74430200	-2.34446400
H	-5.36976300	0.83682000	-1.71711700
C	-3.91478400	-1.08524400	0.14363300
C	-3.58465900	-1.58428900	1.54188300
H	-3.71490400	-1.86447600	-0.59431400
H	-4.96738900	-0.80695300	0.07026200
H	-4.01427300	-2.57940700	1.66959500
H	-2.50274400	-1.65143800	1.69528800
H	-4.01184100	-0.94291500	2.31464200
C	-3.17630600	1.24245300	0.68151700
C	-4.51225300	1.96259100	0.61964200
H	-2.35687900	1.91706100	0.42506300
H	-2.98905800	0.87431100	1.68889500
H	-4.52468200	2.72429500	1.40191500
H	-4.65858400	2.46717100	-0.33728000
H	-5.35276800	1.28572100	0.79403400

Cl	-7.52041700	-0.59119500	0.65400000
H	0.35940600	-0.24493400	-2.36479100
C	1.37243400	-0.47132900	-2.03597800
H	2.05004900	0.26162300	-2.48360900
H	1.64801100	-1.45020800	-2.43831100
C	3.53346800	-0.58609800	-0.13117300
C	4.19354700	-1.61557500	0.55826400
C	4.33763700	0.25808100	-0.91044600
C	5.72183500	0.10689800	-0.97990300
C	6.34655600	-0.91500600	-0.27355900
C	5.57403600	-1.78234100	0.49423200
H	3.88218000	1.05782600	-1.48776100
H	6.30955700	0.78313100	-1.59150300
H	7.42237200	-1.04031600	-0.32605900
H	6.04792800	-2.59085300	1.04072000
H	3.61503600	-2.31287200	1.15665800
Si	1.60444100	-0.45925800	-0.12878000
C	1.12675100	-0.41817500	1.80152100
C	0.73996100	-1.78912500	2.38146800
C	-0.04805300	0.52874800	2.09915100
C	2.31107200	0.08979400	2.64828800
H	1.54093300	-2.52684500	2.28900300
H	-0.16211100	-2.19086600	1.91162100
H	0.52362600	-1.67438000	3.45048100
H	-0.34838900	0.41353700	3.14824200
H	-0.90438800	0.28424400	1.47690300
H	0.20920100	1.58174400	1.95671700
H	3.14936400	-0.60728100	2.66484600
H	1.97066200	0.21918000	3.68336500
H	2.68869700	1.05946600	2.31748700

Si^{R}-IM1

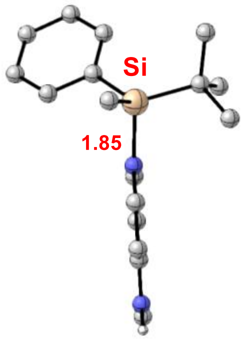

Si	0.53473500	0.65982300	0.95629500
C	0.54927500	0.96120200	2.79292400
H	1.37131600	1.64712000	3.01960600
H	-0.37577700	1.44095500	3.12471100
H	0.70511800	0.05251300	3.37857400
C	-3.34511500	-1.71774800	-0.01169900
C	-2.70342900	-0.90955800	-0.98529100
C	-2.77665800	-1.73028200	1.29029800
C	-1.59073300	-0.19788100	-0.64023400
H	-3.07641700	-0.84204600	-1.99736000
C	-1.66206700	-0.99313500	1.55135300
H	-3.22543500	-2.32478100	2.07591800
N	-1.05470400	-0.22211800	0.60733300
H	-1.08775100	0.42948700	-1.36711900
H	-1.21269500	-0.99312900	2.53691800
N	-4.43126700	-2.43344000	-0.29024800
H	-4.82996400	-2.99005800	0.45317600
C	-5.06126500	-2.48473000	-1.59848500
H	-5.91496700	-3.15530500	-1.53788800
H	-5.41028600	-1.49500900	-1.90295200
H	-4.36560400	-2.86627400	-2.34993900
C	1.89973400	-0.49973300	0.37774600
C	1.84673200	-0.74591500	-1.13760200
C	3.26422300	0.09422900	0.77166000
C	1.73039500	-1.85133800	1.09505400
H	0.91189000	-1.23317000	-1.43333700
H	1.95050200	0.17456900	-1.71772200

H	2.66695300	-1.41804500	-1.41417200
H	3.35196100	0.21682000	1.85599600
H	4.05627800	-0.58992900	0.44724800
H	3.45189700	1.06548400	0.30544400
H	2.56003900	-2.50759800	0.80919500
H	1.74566800	-1.74767700	2.18430900
H	0.79980300	-2.35147500	0.80771800
C	0.31217500	2.27896300	0.03798300
C	-0.85765200	3.01442600	0.28849500
C	1.25827600	2.82217400	-0.83992100
C	-1.07851600	4.24321800	-0.32229100
H	-1.61293100	2.62249500	0.96554700
C	1.04049900	4.05434900	-1.45244000
H	2.17672200	2.28921500	-1.05750400
C	-0.12688800	4.76435000	-1.19587000
H	-1.98936400	4.79387700	-0.11683400
H	1.78345100	4.45664300	-2.13151600
H	-0.29615800	5.72209800	-1.67496900
Cl	4.74043200	-3.39182100	-1.11769800

Si^{R}-TS1

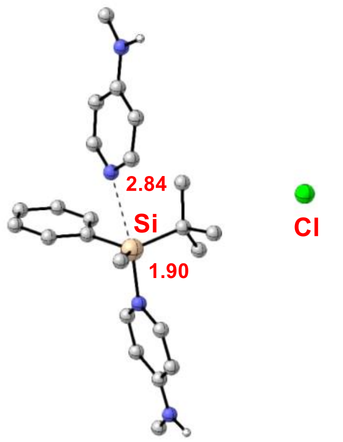

Si	-0.44444600	-0.31590700	-0.64063000
C	-0.17857400	-0.55889400	-2.47975900
H	0.76644000	-1.06715000	-2.66108000
H	-0.98378500	-1.16603400	-2.90211400
H	-0.16109000	0.39178200	-3.01980500

C	-5.14740200	-0.13161300	-0.55601500
C	-4.43285000	-0.84888900	0.43391200
C	-4.38310400	0.43462100	-1.60863500
C	-3.06928900	-0.94199700	0.34741200
H	-4.93965600	-1.32469900	1.26176600
C	-3.02766600	0.28902300	-1.61646900
H	-4.87055000	0.98325700	-2.40483000
N	-2.34773400	-0.38531900	-0.65123100
H	-2.51076400	-1.48230100	1.10105100
H	-2.43636300	0.72160100	-2.41353000
N	-6.47266200	0.01035700	-0.51935600
H	-6.90849900	0.54670500	-1.25618600
C	-7.31787500	-0.52356800	0.53320400
H	-8.34866700	-0.25799000	0.31018700
H	-7.23896000	-1.61255900	0.58288300
H	-7.04704600	-0.10194100	1.50480400
C	-0.17214800	1.41035700	0.15265600
C	-1.34065800	1.74164100	1.10647200
C	1.13153300	1.55217500	0.96052600
C	-0.15390700	2.47238100	-0.96081100
H	-2.29869500	1.81562900	0.58536200
H	-1.44888800	1.00723600	1.91167900
H	-1.14373200	2.71590200	1.56600100
H	2.01358200	1.56979000	0.32097600
H	1.09436700	2.50647200	1.49725600
H	1.26477500	0.75598800	1.69777300
H	-0.04615300	3.46210700	-0.50216300
H	0.68510600	2.32201600	-1.64701100
H	-1.07894200	2.47944800	-1.54698600
C	-0.04277500	-1.81994500	0.40417200
C	0.24281600	-3.03549600	-0.23145000
C	0.02366000	-1.77500000	1.80280900
C	0.58601900	-4.16646100	0.50273900
H	0.21274300	-3.10152900	-1.31500100

C	0.36801400	-2.90476800	2.54223600
H	-0.19125700	-0.85244300	2.33207800
C	0.65226300	-4.10138100	1.89231800
H	0.80649600	-5.09630400	-0.00930800
H	0.41721800	-2.84719100	3.62373300
H	0.92369700	-4.98061400	2.46573400
C	5.12161400	0.09043200	-0.65249800
C	4.48681600	-0.77864100	0.25701700
C	4.32232400	0.60875200	-1.69462600
C	3.14076600	-1.05650800	0.09012300
H	5.02787100	-1.22409800	1.08127800
C	2.99229500	0.25628100	-1.76015500
H	4.75112800	1.28004100	-2.42990900
N	2.37856600	-0.55729700	-0.88619500
H	2.64076400	-1.71657600	0.79540600
H	2.37132900	0.65770400	-2.55720600
N	6.43163400	0.40946800	-0.55588500
H	6.78646600	1.11364100	-1.18451700
C	7.27514900	-0.01243500	0.54249400
H	8.26967000	0.40356900	0.39231700
H	6.89232200	0.33887500	1.50664200
H	7.35970400	-1.10243400	0.57763600
Cl	-0.04700800	5.51434900	1.64391500

Si-IM2

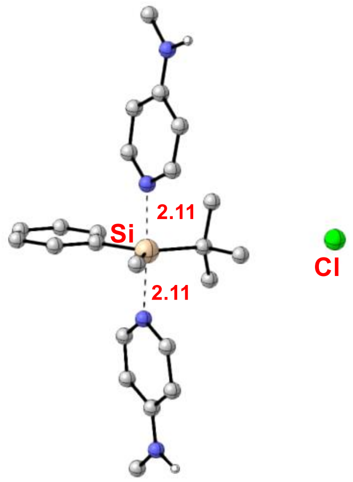

Si	-0.00008300	-0.30981100	-0.60902900
C	-0.00012700	-0.62557600	-2.47856300
H	0.88266500	-1.19343600	-2.77518800
H	-0.88278500	-1.19375400	-2.77500200
H	-0.00038700	0.30089300	-3.06151900
C	-4.91263100	-0.02416400	-0.70914600
C	-4.26071400	-0.79241400	0.27887800
C	-4.08614600	0.56038600	-1.69797700
C	-2.89017500	-0.91698400	0.24423200
H	-4.81355400	-1.28304000	1.06804300
C	-2.73121600	0.36928800	-1.64749100
H	-4.51966600	1.15593900	-2.49211400
N	-2.10627800	-0.35251100	-0.68983000
H	-2.38840700	-1.49627000	1.00864400
H	-2.10064400	0.81450800	-2.40640500
N	-6.24259900	0.14557800	-0.72921600
H	-6.63159000	0.72961000	-1.45442500
C	-7.14057700	-0.40676800	0.26606100
H	-8.15728400	-0.11847800	0.00792300
H	-7.08238800	-1.49864100	0.28530900
H	-6.91017700	-0.02371900	1.26449400
C	0.00003500	1.44285400	0.22985800
C	-0.00002500	2.53050800	-0.86065200
C	-1.23401200	1.68705900	1.12488200
C	1.23428600	1.68695900	1.12463000
H	0.88641900	2.47904400	-1.49968300
H	-0.88715100	2.47983900	-1.49880800
H	0.00065100	3.51372800	-0.37476500
H	-1.36293800	0.91110400	1.88630000
H	-1.09924600	2.64317700	1.64117800
H	-2.16217300	1.75967100	0.55565300
H	1.09948700	2.64287900	1.64126700
H	1.36354800	0.91078100	1.88576400
H	2.16228200	1.75990800	0.55516900

C	-0.00006800	-1.85530900	0.47017300
C	-0.00008900	-3.11379900	-0.14408900
C	-0.00001200	-1.80759600	1.87045800
C	-0.00006400	-4.28407900	0.61104500
H	-0.00012700	-3.19072600	-1.22786600
C	0.00000600	-2.97512500	2.63061500
H	-0.00000200	-0.85055600	2.38289400
C	-0.00001400	-4.21632800	2.00104900
H	-0.00008300	-5.24779600	0.11404100
H	0.00004000	-2.91435300	3.71317300
H	0.00000600	-5.12612200	2.59070000
C	4.91254700	-0.02439900	-0.70924700
C	4.26062200	-0.79272100	0.27871300
C	4.08607300	0.56019400	-1.69806200
C	2.89007800	-0.91726800	0.24405500
H	4.81345300	-1.28342000	1.06783900
C	2.73113900	0.36911800	-1.64758500
H	4.51960800	1.15575700	-2.49218400
N	2.10618300	-0.35269700	-0.68994600
H	2.38830400	-1.49660000	1.00842900
H	2.10059500	0.81432500	-2.40653100
N	6.24251900	0.14533500	-0.72930800
H	6.63148500	0.72958100	-1.45435800
C	7.14046100	-0.40682900	0.26610300
H	8.15716400	-0.11846400	0.00803100
H	6.90993100	-0.02372300	1.26448700
H	7.08238300	-1.49870500	0.28543100
Cl	0.00039400	5.57131900	1.75240800

Si^{R}/C^{S}-TS2

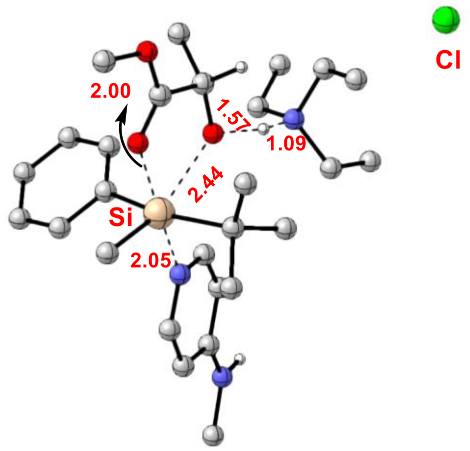

H	-3.84060400	-2.38359800	-0.31739800
C	-3.41857400	-1.79284000	-1.13290300
H	-4.18245100	-1.08636000	-1.46598600
H	-3.24330300	-2.48602900	-1.96020200
Si	-1.72507400	-1.06226200	-0.62163200
C	-0.92279300	-0.79920300	-2.41833700
C	-0.24836200	-2.07076300	-2.95550000
C	0.10852300	0.33172000	-2.51274600
C	-2.03447900	-0.44256200	-3.42714600
H	-0.91496000	-2.93780000	-2.94133900
H	0.66086000	-2.31875900	-2.40132800
H	0.05194200	-1.90653600	-3.99767000
H	0.53120300	0.35830600	-3.52571500
H	0.91991200	0.16371700	-1.81281100
H	-0.33030000	1.31621600	-2.32657400
H	-2.74412100	-1.25499400	-3.58915500
H	-1.56740100	-0.22446800	-4.39517300
H	-2.59601000	0.45009900	-3.14308900
C	-2.00686700	-0.94479200	1.31495500
C	-3.34028100	-0.74947100	1.72095700
C	-1.05663400	-0.96377300	2.34143900
C	-1.40735300	-0.84604600	3.68736900
C	-2.73657700	-0.68815000	4.05696300
C	-3.70477900	-0.62739600	3.05833200
H	-0.01482100	-1.04133900	2.07731900

H	-0.63109100	-0.87237100	4.44582700
H	-3.01437600	-0.59870500	5.10126400
H	-4.74785400	-0.48202100	3.31891200
H	-4.12712200	-0.67343700	0.97778000
C	-2.42668800	3.72024200	-0.16663900
C	-1.28659300	3.02882500	0.31267300
C	-3.40401900	2.94632400	-0.82829100
C	-1.18660100	1.67868100	0.11714800
H	-0.50481500	3.56244300	0.84066000
C	-3.20431000	1.59156700	-0.98106600
H	-4.31006300	3.39194800	-1.21457200
N	-2.11718900	0.94694800	-0.53391200
H	-0.33459600	1.12102700	0.49600400
H	-3.95427600	0.99108300	-1.47988400
N	-2.55919000	5.04194600	0.01797700
H	-1.81479100	5.52716000	0.49679200
C	-3.69909100	5.81120000	-0.44115500
H	-3.55310300	6.84833900	-0.14717700
H	-4.62701500	5.44751900	0.00907200
H	-3.79447000	5.76665300	-1.53000000
C	-0.00649700	-3.34809900	-0.18765600
O	-1.15470400	-2.97451100	-0.42714900
O	0.30543900	-4.61320500	-0.17823200
C	-0.75184600	-5.55830900	-0.44919300
H	-1.54276700	-5.44767800	0.29157400
H	-0.28765300	-6.53699300	-0.37280300
H	-1.14623300	-5.39418600	-1.45152700
C	1.11089200	-2.37975400	0.13223400
H	1.89614900	-2.59790700	-0.61536900
C	1.68736300	-2.80290100	1.50544700
H	2.24131100	-3.73778500	1.40428400
H	0.90576500	-2.94862800	2.25308100
H	2.37240000	-2.03638700	1.86960500
O	0.62484600	-1.09647100	0.02380300

H	1.74776500	-0.00096100	0.06511500
N	2.58621200	0.66506200	0.26904500
C	2.44308300	0.95741900	1.73922800
C	3.73856400	1.17680700	2.50446200
H	1.90394400	0.10819000	2.16147300
H	1.78816600	1.82824600	1.81495300
H	3.47754800	1.47304500	3.52249800
H	4.32960100	0.26181600	2.56643100
H	4.36258100	1.95756000	2.07089100
C	3.80495300	-0.14764900	-0.04928700
C	3.85022000	-0.57771100	-1.50607200
H	3.76257600	-1.02107900	0.60294800
H	4.69649100	0.42723300	0.20489400
H	4.61301000	-1.35111400	-1.61226600
H	2.89386100	-0.99222800	-1.83850400
H	4.12677400	0.24547400	-2.16676200
C	2.46097000	1.89823700	-0.57281000
C	3.49690600	2.96873200	-0.28150600
H	1.45179000	2.27863400	-0.40702200
H	2.52239900	1.58306200	-1.61249400
H	3.37876500	3.76155800	-1.02316600
H	3.35761400	3.41478400	0.70494600
H	4.51796900	2.58615800	-0.35806200
Cl	7.10215100	1.47097400	-0.11717400

Si^{S}-4g

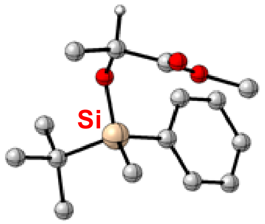

C	-1.82472900	-0.66025600	-1.25738400
C	-3.63852500	-0.81257100	0.84440200
C	-1.47519800	-0.10776100	-0.01875100
C	-3.05820800	-1.27609100	-1.44754200

C	-3.96764600	-1.35269800	-0.39490600
C	-2.40296100	-0.19450200	1.02717000
H	-3.30989700	-1.70010800	-2.41348900
H	-4.34207800	-0.87337200	1.66736200
H	-1.11265500	-0.62217200	-2.07632200
H	-4.92820900	-1.83451200	-0.54009900
H	-2.16210700	0.21717500	2.00419600
C	0.86294800	0.28005300	1.92324900
H	0.69620300	-0.77244900	2.16769800
H	1.93125400	0.48911500	2.02152700
H	0.35037600	0.87302900	2.68749300
Si	0.19203500	0.72338300	0.23017100
C	0.03518200	2.59550800	0.02067900
C	1.40400900	3.24075400	0.27736400
C	-0.41702100	2.90792200	-1.41232400
C	-0.99302400	3.16064800	1.00944300
H	1.75773200	3.05214700	1.29635900
H	2.15970800	2.86587200	-0.41982800
H	1.33940900	4.32784900	0.14545500
H	-0.49313300	3.99282500	-1.55650400
H	0.29254400	2.51982000	-2.14862100
H	-1.39984500	2.47509000	-1.62563600
H	-0.71520200	2.96507500	2.05031900
H	-1.06784600	4.24831900	0.88911900
H	-1.98934900	2.74052400	0.84020100
C	2.02863200	-1.86924100	-0.08433500
O	2.86449200	-2.44974100	0.55649700
O	0.74605000	-2.22692100	-0.13435100
C	0.35566100	-3.33211700	0.68665200
H	0.58391200	-3.12700800	1.73379400
H	-0.71759900	-3.43911800	0.54544500
H	0.87401900	-4.23882700	0.37173000
C	2.29449400	-0.66129500	-0.98253400
H	2.38103900	-1.07966100	-1.99303900

C	3.59948000	0.02496600	-0.62008300
H	3.78645600	0.83274100	-1.32887400
H	3.55780000	0.44159900	0.38849100
H	4.42659100	-0.68417200	-0.66831300
O	1.21368500	0.24453000	-1.00807700

Si^{R}-4g

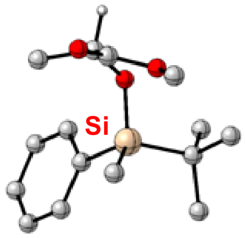

C	2.40044000	-0.79230000	-0.97090600
C	3.92146100	-0.73752900	1.35409700
C	1.83437800	-0.25895000	0.19606500
C	3.70013500	-1.28962900	-0.98020300
C	4.46279100	-1.26343700	0.18485000
C	2.62005900	-0.24104400	1.35682100
H	4.11767100	-1.69936200	-1.89333300
H	4.51076100	-0.71800900	2.26418400
H	1.81131400	-0.82295400	-1.88301300
H	5.47473300	-1.65249800	0.18137000
H	2.21640800	0.16129400	2.28163900
C	-0.73793300	0.17292200	1.81805500
H	-1.66944000	0.73927400	1.89320400
H	-0.95956500	-0.88075500	2.01516400
H	-0.07722900	0.51644900	2.61971100
Si	0.08341000	0.43418500	0.15482700
C	-2.71653200	-1.35248900	-0.12529500
O	-3.26161400	-2.25424900	0.45766800
O	-3.15505900	-0.09517300	-0.11807100
C	-4.30285500	0.15921400	0.69885200
H	-4.09543400	-0.10930700	1.73601100
H	-4.49617600	1.22602900	0.61438600
H	-5.16116000	-0.41023000	0.34022800

C	-1.47533800	-1.52676500	-1.00215000
H	-1.87057500	-1.72516100	-2.00584800
C	-0.65494000	-2.72555600	-0.55129500
H	0.17326500	-2.87946300	-1.24397700
H	-0.24777900	-2.57504500	0.45234500
H	-1.27649300	-3.62108000	-0.53845100
O	-0.72412300	-0.33933300	-1.09448600
C	0.09579000	2.25701100	-0.33227500
C	0.87373700	3.05909600	0.71918700
C	0.76667100	2.42233700	-1.70209700
C	-1.34970600	2.76906900	-0.40897300
H	0.42658200	2.96496600	1.71452100
H	1.91766700	2.73528500	0.78356200
H	0.87320100	4.12388800	0.45601600
H	0.76561200	3.48000200	-1.99337000
H	1.80710800	2.08310900	-1.68566800
H	0.23772100	1.86092100	-2.47789200
H	-1.84775900	2.72412100	0.56500400
H	-1.35715600	3.81571800	-0.73745300
H	-1.94358300	2.18254000	-1.11522200
